# Chemically Fueled Communication Along a Scaffolded Nanoscale Array of Squaramides

**DOI:** 10.1002/anie.202307841

**Published:** 2023-08-10

**Authors:** Luis Martínez‐Crespo, Iñigo J. Vitórica‐Yrezábal, George F. S. Whitehead, Simon J. Webb

**Affiliations:** ^1^ Department of Chemistry University of Manchester Oxford Road Manchester M13 9PL UK; ^2^ Manchester Institute of Biotechnology University of Manchester 131 Princess Street Manchester M1 7DN UK

**Keywords:** Hydrogen Bonding, Molecular Communication, NMR, Squaramides, Out-of-Equilibrium

## Abstract

Relaying conformational change over several nanometers is central to the function of allosterically regulated proteins. Replicating this mechanism artificially would provide important communication tools, but requires nanometer‐sized molecules that reversibly switch between defined shapes in response to signaling molecules. In this work, 1.8 nm long rigid rod oligo(phenylene‐ethynylene)s are scaffolds for switchable multi‐squaramide hydrogen‐bond relays. Each relay can adopt either a parallel or an antiparallel orientation relative to the scaffold; the preferred orientation is dictated by a director group at one end. An amine director responded to proton signals, with acid‐base cycles producing multiple reversible changes in relay orientation that were reported by a terminal NH, which is 1.8 nm distant. Moreover, a chemical fuel acted as a dissipative signal. As the fuel was consumed, the relay reverted to its original orientation, illustrating how information from out‐of‐equilibrium molecular signals can be communicated to a distant site.

## Graphical abstract

A two nanometer‐long molecular device has been synthesised that contains a rigid rod ‐ to provide structural definition ‐ attached to a molecular relay that switches direction in response to an incoming signal. Like natural systems, it responds to temporal changes in a signal, exemplified by the consumption of a molecular fuel, changes that are transmitted down the device.

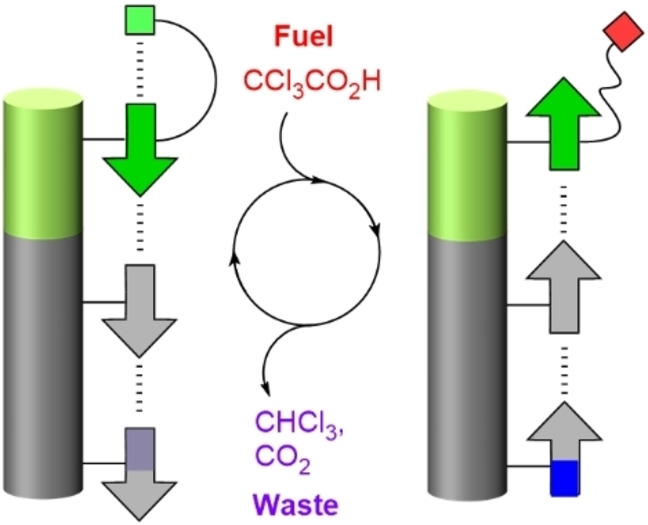

## Introduction

The cooperative assembly of hydrogen bond donors and acceptors produces nanometer‐sized domains of secondary structure in proteins, such as α‐helices and β‐sheets. The motion of domains relative to one another at room temperature produces a conformational landscape for each protein. For some proteins, incoming signals can perturb this conformational distribution, with the external signal causing domain reorientation that can extend over several nanometers. This process underpins the allosteric (“other shape”) regulation of enzymes, which involves intramolecular communication of a shape change that is initiated by signaling molecules binding to a specific region.[^1^, ^2^] For example, the binding of a ligand to the extracellular region of G‐protein coupled receptors (GPCRs) causes a change in the relative disposition of seven interconnected transmembrane α‐helices, with the resulting change in conformational population triggering a cascade of intracellular events.^[3]^ The rigidity and defined length of the α‐helices gives structural definition, allowing their relative motion to generate the transmembrane communication process.

The allosteric regulation of proteins has inspired the development of artificial oligomers with similar responsive properties, aiming to produce devices able to transmit molecular signals over nanometer distances without physical transport of a signaling molecule.^[4]^ Such communication tools could have widespread applications, for example transferring information between sites in molecular factories. Most of these biomimetic oligomers are semiflexible, allowing them to adopt multiple interconverting conformations. For some examples, their conformational landscape can be reversibly altered by external stimuli. The groups of Yashima, Clayden and Webb have used 3_10_‐helical peptide foldamers (“folded oligomers”) to develop systems with end‐to‐end communication properties.[^5^, ^6^] These systems have been thoroughly studied in organic solvents, with some also studied in bilayer membranes.[^7^, ^8^] Similarly, Clayden and co‐workers showed oligo‐urea foldamers in organic solvents form intramolecular head‐to‐tail hydrogen‐bonded arrays that can adopt opposite net dipole orientations.[^9^, ^10^] Rapid interconversion between conformations permits chemical inputs, such as anions, acids or bases, at one end to control the orientation of the urea chains and therefore alter binding and/or reactivity at distant sites.

Nonetheless, foldamer flexibility can lead to uncertainty about the dimensions of key conformations, which might be an issue when relaying information between sites that are separated by fixed distances. Adding a rigid scaffold can provide this structural definition. Rigid macrocycles have been used as scaffolds. Hou and co‐workers showed that pillar[*n*]arenes rim‐functionalized with peptides form unimolecular transmembrane channels defined by intramolecular hydrogen bonds.^[11]^ Rebek and co‐workers showed that tetra‐urea calix[4]arenes dimerize into capsules with cooperative hydrogen bonds between urea groups on the rims.[^12^, ^13^] A similar outcome was observed by Ballester and co‐workers with tetra‐urea calix[4]pyrrole capsules.[^14^, ^15^, ^16^] Different urea array directionalities – either clockwise or counter‐clockwise – were observed in both types of capsule, with the direction determined by chiral substituents on the hosts.[^17^, ^18^] Linear rigid scaffolds have also been used to pre‐organize hydrogen‐bonded peptide arrays; artificial β‐barrel ion channels developed by Matile and co‐workers used linear oligo‐phenylene rigid‐rods to prevent structural collapse of the peptide portions.[^19^, ^20^]

Scaffolded arrays able to relay information are rare, but an example employing supramolecular interactions other than hydrogen bonds has been reported. In organic solvent, oligo‐xanthenes functionalized with aromatic tertiary amides transferred chirality from one terminus to the other, using conformational constraints and dipole‐dipole interactions between bis(*iso*‐propyl) amides arrayed along the scaffold.^[21]^ The preorganization provided by the rigid linear skeleton was crucial for the efficient communication of chirality.

**Figure 1:**
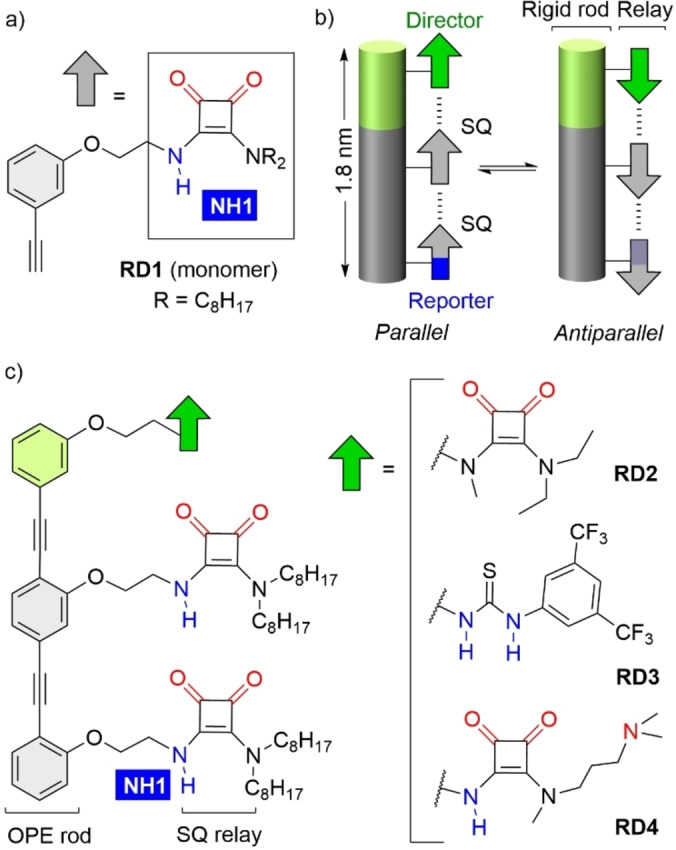
(a) Structure of mono‐squaramide **RD1**. Hydrogen bond donors are shown in blue, acceptor groups are shown in red. (b) Schematic representation of a scaffolded relay. A rigid rod is functionalized with directional units (grey arrows), which can adopt opposite and interconvertible orientations. The preferred orientation is induced by a “director” group (green arrow) and reported by the final unit (blue arrow). (c) Structures of **RD2‐4**. OPE=Oligo‐(phenylene‐ethynylene). SQ=Squaramide.

Combining a rigid scaffold and a conformationally switchable hydrogen bonded array is an attractive approach towards switchable information relays. Hydrogen bonds have predictable well‐defined geometries and are robust enough to persist in different environments. To explore this approach, we created a series of rigid‐rods functionalized with multiple squaramide (SQ, boxed in Figure 1a) units that form intramolecular hydrogen bonds to each other. The rigid scaffold prevents structural collapse and defines relay length (≈2 nm), whilst an array of hydrogen bonded SQ units was designed to form opposite yet interconvertible conformations relative to the rigid rod. Given SQ lengths are ca. 0.5 nm,[^22^, ^23^] only three SQ are needed to span 2 nm. With few components involved in the relay, there are fewer opportunities to form defects that lower the fidelity of information transfer (such as the “tendril perversions” in α‐amino‐*iso*‐butyric acid (Aib) foldamers).^[6b]^ A directing group (a “director”, shown in green in Figure 1b) at one end of each rigid scaffold dictates which orientation is preferred. Its preference will be relayed to the final SQ, 1.8 nm distant, which will report on array orientation (“reporter”, NH1, shown in blue in Figure 1b). Using a director responsive to an external stimulus, such as a chemical fuel, should allow this end‐to‐end molecular communication to reflect changes in signal strength over time, as experienced by enzymes under allosteric control.

## Results and Discussion

### Design and Synthesis of Intramolecular SQ Relays

Squaramides (SQs) are vinylogous amides with a rigid planar structure, in which each hydrogen bond donor NH is 4 Å from the adjacent, and 5 Å from the opposite, hydrogen bond accepting carbonyl oxygen.[^22^, ^23^] Much like amides, SQs can self‐assemble into head‐to‐tail aggregates.[^24^, ^25^, ^26^, ^27^] Also like amides, SQ‐SQ pairs can have opposite orientations, with the first SQ acting either as a hydrogen bond donor or as a hydrogen bond acceptor. Due to these properties, SQs have been widely used to create structures with well‐defined conformational properties.[^28^, ^29^, ^30^, ^31^, ^32^, ^33^, ^34^, ^35^] They were a natural choice for the construction of an intramolecularly hydrogen bonded information relay.

Oligo‐(phenylene‐ethynylene)s (OPEs) were chosen for the linear scaffold. These rigid‐rods have inherent directionality and can be prepared by well‐established synthetic methods. The rigidity and defined length of these rigid‐rods has led to their use in various supramolecular systems.^[36]^ OPEs can span the 2–3 nm width of a phospholipid bilayer. Sato, Kinbara and co‐workers developed self‐assembled transmembrane channels formed by multiple interconnected OPE rods,[^37^, ^38^, ^39^] whereas Zhu and co‐workers used a single rod to preorganize anion binding macrocycles into unimolecular ion channels.^[40]^ The OPE backbone is extendable^[40]^ and functional motifs can be added at either end. Thus, OPE termini can be elaborated with directing groups. Crucially for our design, the repeat unit in OPEs has a center‐to‐center distance between adjacent aromatic rings (7 Å) that is similar to the distance between the centroids of a reported hydrogen bonded SQ array.^[25]^

Secondary‐tertiary SQs, which have two carbonyl oxygens and one NH (Figure 1a), were appended to these OPE rigid rods. Each secondary‐tertiary SQ possesses a hydrogen bond donor and two acceptors, allowing successive hydrogen bonds between SQ units to give an array. Although the rigidity of both OPEs and SQs introduces a high degree of preorganization, flexible ethylene linkers between the OPE and each SQ provides enough freedom to achieve different SQ‐SQ hydrogen bond geometries, including opposite orientations with respect to the OPE backbone. If OPE directionality is arbitrarily defined as pointing from the *ortho* substituted aromatic ring at one end to the *meta* substituted ring at the other, the overall dipole of the SQ chain could be oriented either “parallel” or “antiparallel” (Figure 1b). A “director”, with either hydrogen bond acceptor, donor or switchable properties, will be added to one OPE terminus and a “reporter” added to the other OPE terminus (Figure 1c).

In brief, compounds **RD1** (Figure 1a) and **RD2‐4** (Figure 1c) were prepared after optimization of standard synthetic conditions (Schemes S1–S2).[^40^, ^41^] Compounds **RD1‐4** were fully characterized in CD_2_Cl_2_ at millimolar concentrations using NMR spectroscopy, correlation spectroscopy (COSY) and nuclear Overhauser effect spectroscopy (NOESY) data (Figures S1–S5). Conformational diversity in solution was assessed using variable temperature (VT) NMR experiments (Figures S8–S12). NMR titrations with DMSO‐*d*_6_ (Figures S13–S17) provided information on the solvent exposure of each NH. Moreover, solution studies were supported by several solid state structures, determined using single crystal X‐ray diffraction.

### Effect of Different Directing Groups on the SQ Relay

**Figure 2:**
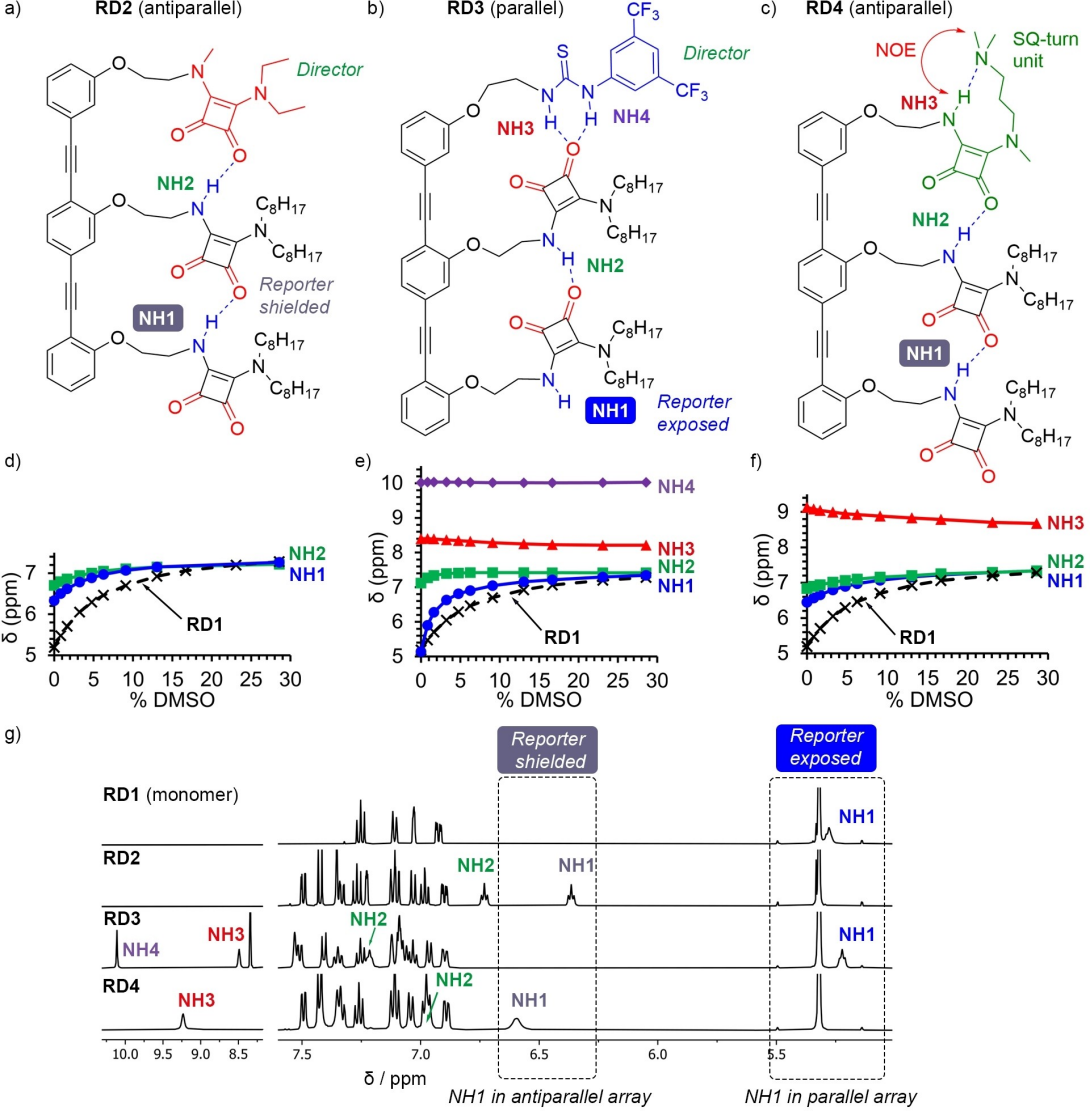
(a,b) Major conformers of **RD2** and **RD3** in solution, showing hydrogen bond donors (in blue) and hydrogen bond acceptors (in red). (c) Major conformer of **RD4** in solution, showing the SQ‐turn motif in the “director” group (in green). (d‐f) Variation of the chemical shift of the NH signals of compounds (d) **RD2**, (e) **RD3** and (f) **RD4** during DMSO‐*d_6_* titrations (circles: NH1; squares: NH2; triangles: NH3; diamonds: NH4). The exposed NH signal of monomer **RD1** (×, structure in Figure 1) is shown as a reference. (g) Partial ^1^H NMR (CD_2_Cl_2_, 500 MHz, 298 K) spectra of **RD1‐4** (2 mM). The NH1 chemical shift reports on SQ chain orientation: Parallel for **RD3** (5.2 ppm) and antiparallel for **RD2** and **RD4** (6.3–6.7 ppm).

The first of the trimers, **RD2**, has a di‐tertiary SQ as director. Without any NHs, this group can act only as a hydrogen bond acceptor and only participate in an antiparallel array (Figure 2a).[^42^, ^43^] The simple squaramide **RD1** (Figure 1a) provides a useful contrast, since this monomeric compound is unable to form intramolecular hydrogen bonds and the “reporter”, NH1, is always exposed to the solvent. The ^1^H NMR spectrum of **RD2** showed resonances at 6.36 and 6.73 ppm for “reporter” NH1 and NH2, respectively (Figure 2d and 2 g), which are both substantially downfield of NH1 of **RD1** (5.28 ppm, Figure 2d and 2 g). Moreover, DMSO‐*d_6_* titrations showed these two NHs in **RD2** were much less affected by the addition of this polar solvent than the NH of **RD1** (Figure 2d), in agreement with shielding of NH1 and NH2 from bulk solution.

**Figure 3:**
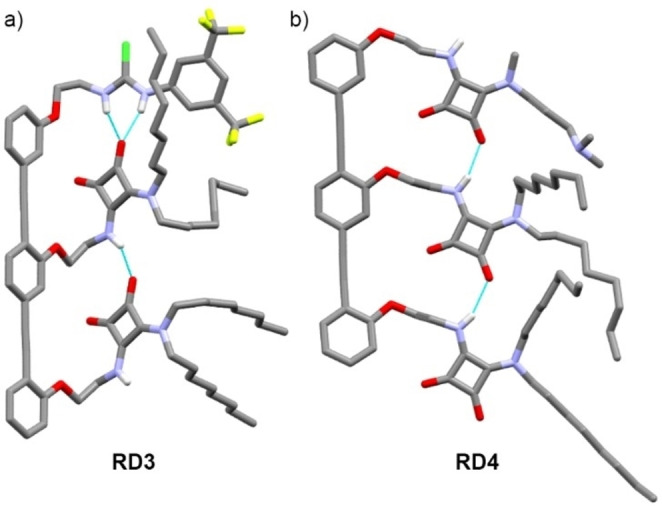
X‐ray crystal structures of (a) **RD3** (CCDC:2240192) and (b) **RD4** (CCDC: 2240193) showing either a parallel or an antiparallel orientation of the SQ chain, respectively. The intramolecular hydrogen bonds between SQ in each structure are shown (cyan). C atoms are shown in grey, H in white, N in light blue, O in red, F in yellow and S in green. H atoms not involved in hydrogen bonds have been removed for clarity.

To induce the reverse directionality in the hydrogen bonded array, a 3,5‐bis(trifluoromethyl)phenylthiourea (TFPT) was employed as the directing unit in **RD3** (Figure 2b).^[9]^ TFPTs are poor hydrogen bond acceptors but powerful hydrogen bond donors, which has led them to be applied as anion binders and organocatalysts.^[44]^ Comparison of the ^1^H NMR spectra of **RD2** and **RD3** showed a large difference in the chemical shift of “reporter” NH1 (Figure 2g), which is upfield shifted in **RD3** (5.22 ppm) to a position similar to that of the NH of **RD1** (5.28 ppm). This shift suggests that NH1 is not forming intramolecular hydrogen bonds and is exposed, consistent with a parallel orientation of the hydrogen bond array. A thiourea to SQ hydrogen bond is confirmed by the chemical shifts of NH3 and NH4, which are much higher than those of a reference mono‐thiourea (Figure S6). The presence of internal hydrogen bonds was confirmed with a DMSO‐*d_6_* titration, which produced a negligible effect on NH2‐NH4 but gave a strong change in the chemical shift of NH1 (Figure 2e). The crystal structure of **RD3** supports the spectroscopic data in solution, showing a bifurcated hydrogen bond from the thiourea unit to the C=O of the adjacent SQ (N⋅⋅⋅O 2.818(5) and 2.836(6) Å, Figure S19a) that produces a SQ chain in parallel orientation (Figure 3a).^[45]^

To incorporate a potentially switchable “director”, we designed **RD4**, which has a 3‐(*N*,*N*‐dimethylamino)propyl substituent on the director SQ (Figure 2c). This motif was reported by Rotger and co‐workers to form an SQ‐turn, involving a strong intramolecular hydrogen bond between the tertiary amine group and the SQ‐NH (Figure 2c, top).^[31]^ In **RD4**, this SQ‐turn would mask NH3 and force the director to only present hydrogen bond acceptors to the rest of the relay, inducing an antiparallel orientation. The presence of a SQ‐turn was confirmed in CD_2_Cl_2_ by the chemical shift of NH3, which is strongly downfield at 9.2 ppm (Figure 2g). The formation of this strong intramolecular hydrogen bond between NH3 and the *N*,*N*‐dimethylamino group is further confirmed by an NOE cross‐peak between the methyls and NH3 (Figure 2c and S5). The rest of the SQ chain has spectroscopic properties similar to those of **RD2**, in agreement with an antiparallel orientation of the hydrogen bond relay. The chemical shift of reporter NH1 (>6.5 ppm) suggests that it is shielded from solvent within an intramolecular hydrogen bonding array (Figure 2g). A DMSO‐*d_6_* titration produced a smaller effect on NH1, NH2 and NH3 of **RD4** than on the exposed NH1 of **RD1** (≈0.5 ppm and ≈2 ppm respectively, Figures 2f, S13 and S16), suggesting all three NHs are shielded in intramolecular hydrogen bonds. Lowering the temperature to 198 K still showed only a single set of signals (Figure S12), indicating no significant amounts of alternative conformers are present.

The crystal structure of **RD4** also shows an antiparallel conformation of the SQ relay, although the SQ‐turn is not observed in the solid state (Figure 3b). The 3‐(*N*,*N*‐dimethylamino)propyl group instead packs with the *n*‐octyl sidechains. The intermolecular hydrogen‐bond and the packing interactions are sufficient to overcome the strong intramolecular NH⋅⋅⋅N hydrogen bond in the SQ‐turn; similar cooperativity‐induced conformational changes have been reported for di‐secondary SQs.[^42^, ^43^]

These ^1^H NMR spectroscopic studies of **RD1**‐**RD4** confirm that the chemical shift of NH1 reports on the orientation of the SQ chain (Figure 2g), with a parallel orientation giving δ<5.6 ppm and an antiparallel orientation giving δ>6.3 ppm. The signals of some aromatic CHs also report on relay orientation, albeit more weakly, as they are more downfield shifted when the relays have an antiparallel orientation (Figures S7 and S20). To report on switches in array orientation caused by structural changes in the director, NH1 is a versatile spectroscopic handle that also shows an end‐to‐end conformational relay is in operation.

### Transfer of Conformational Information in RD4: Acid‐Base Induced Reversible Conformational Switch

**Figure 4:**
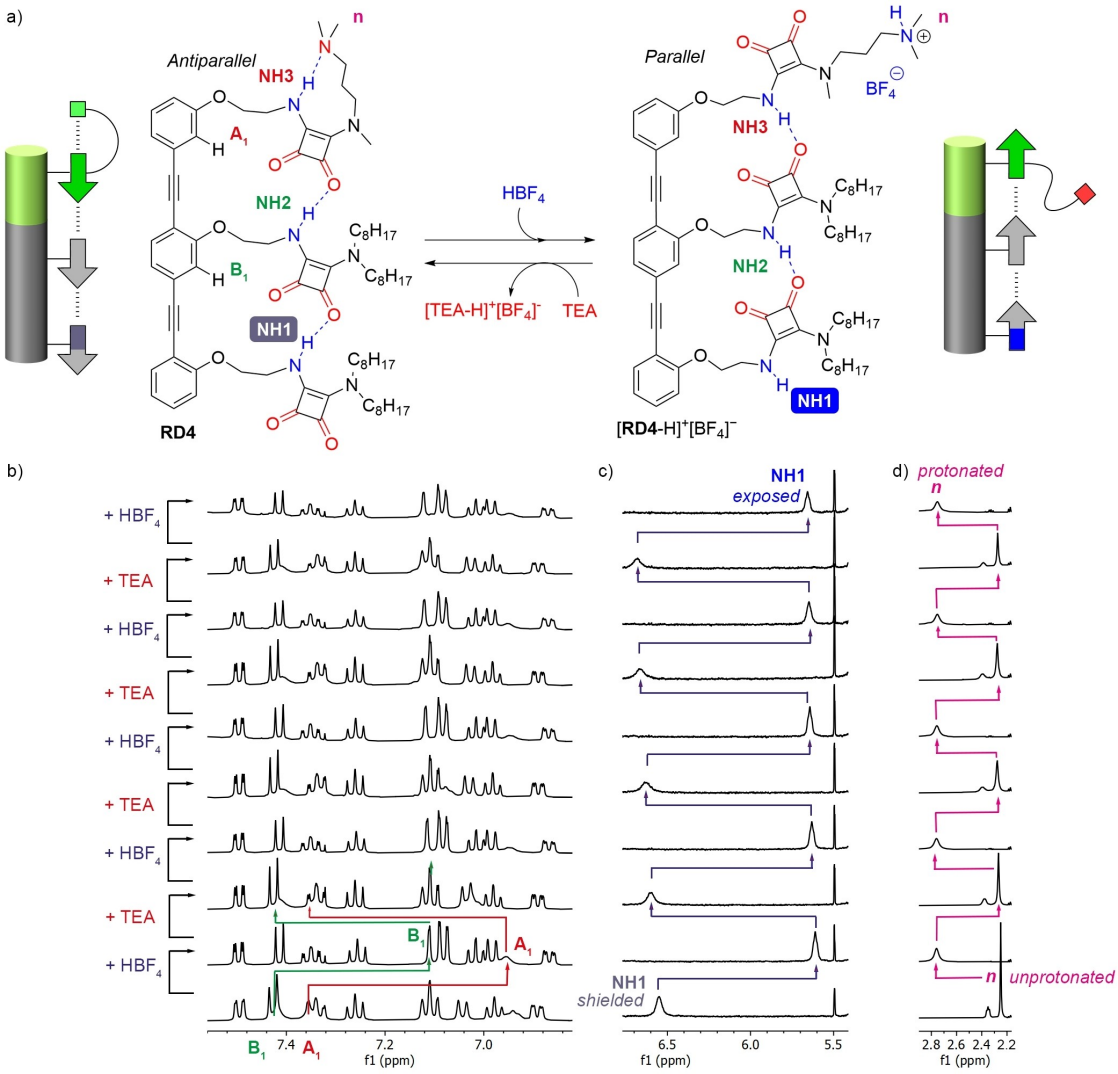
(a) Conformational change of **RD4** during protonation and deprotonation. (b‐d) Partial ^1^H NMR (CD_2_Cl_2_, 500 MHz, 298 K) spectra of **RD4** (1 mM), showing the effect of alternating additions of HBF_4_ and TEA. (b) The changes in chemical shift of the aromatic signals A_1_ and B_1_ allow interchange between antiparallel (downfield) and parallel (upfield) conformers to be monitored. (c) The signal of NH1 at the reporter terminus, 1.8 nm distant from the director, reflects concomitant inversions in the orientation of the relay. (d) Methyl signal *n* reports on protonation of the amine at the terminal director.

The SQ‐turn in the **RD4** director should be an allosteric binding site for protons, with protonation of the amine group removing the turn, releasing NH3 and producing a parallel orientation that leaves the remote NH1 of **RD4**‐H^+^ exposed (Figure 4a).

Titration of a solution of **RD4** in CD_2_Cl_2_ with HBF_4_ (Figure S21), a strong acid with a non‐coordinating anion, produced a downfield shift of the NMe_2_ resonance (signal *n*, Figure 4a) that indicated protonation. Crucially a commensurate upfield shift of the distant NH1 reporter signal was observed (see Figure S21 and first addition in Figure 4c), which is consistent with a conformational change from antiparallel to parallel and loss of the intramolecular hydrogen bond to this NH. The addition of 1.4 equivalents of HBF_4_ maximized the chemical shift change in NH1 (from 6.5 to 5.6 ppm); further acid addition caused negligible changes in the NMe_2_ and NH1 signals (see Figure S22). The conformational switch was also reported by aromatic signals A_1_ and B_1_ (Figure 4a), which switched from the region characteristic of antiparallel orientation (7.5–7.3 ppm) to that indicating parallel orientation (6.9–7.1 ppm) (Figure 4b). The addition of HBF_4_ had a substantial effect on the resonance of NH3, which moved upfield by approx. 1.5 ppm due to the loss of the strong hydrogen bond in the SQ‐turn and its replacement with a hydrogen bond to the SQ array. The effect on the NH2 resonance was smaller as this NH remains hydrogen bonded within the SQ array. However, these two NH signals broadened and overlapped with the aromatic signals, making them difficult to locate precisely after complete protonation.

Although no additional conformations were observed for unprotonated **RD4** at either 298 K or 258 K, the broadness of the NH2 and NH3 resonances at 298 K suggested that [**RD4**‐H]^+^[BF_4_]^−^ may adopt multiple conformations that interconvert at an intermediate timescale in the ^1^H NMR spectrum. Decreasing the temperature to 258 K revealed two species in solution, in a 7 : 3 ratio (Figure S26). The spectrum of the major species agreed with the **RD4**‐H^+^ structure proposed in Figure 4a, with NH2 and NH3 showing chemical shifts that are in the intramolecular hydrogen bond region and NH1 shifted much more upfield into the “exposed” NH region. The spectra of both **RD4**‐H^+^ species were similar, with the largest chemical shift differences around the protonated amine. Therefore, we speculate that the minor species could have an interaction with the anion BF_4_^−^ (e.g., Figure S26),[^46^, ^47^] but unambiguous structural identification was not possible. Nonetheless, comparison to the spectrum of **RD4** at 258 K showed both major and minor **RD4**‐H^+^ species lack an SQ‐turn (Figure S27) and have reporter NH1 exposed, i.e. the arrays are in parallel orientations.

The addition of the base triethylamine (TEA) to [**RD4**‐H]^+^[BF_4_]^−^ returned the relay to its original state, indicated by changes in signals *n*, NH1, A_1_ and B_1_ (Figure 4b‐d). A sequence of acid‐base cycles showed switching between parallel and antiparallel conformers was fully reversible, proving that the **RD4**/[**RD4**‐H]^+^[BF_4_]^−^ system is stable yet dynamic (Figures 4b‐d and S24). The net effect is to transfer chemical information ‐ a proton messenger binding to the allosteric site ‐ via a cooperative conformational change along the scaffolded relay, to produce a spectroscopic signal from a ≈2 nm distant reporter (NH1).

### Transfer of Conformational Information in RD4: Chemically Fueled Molecular Communication

**Figure 5:**
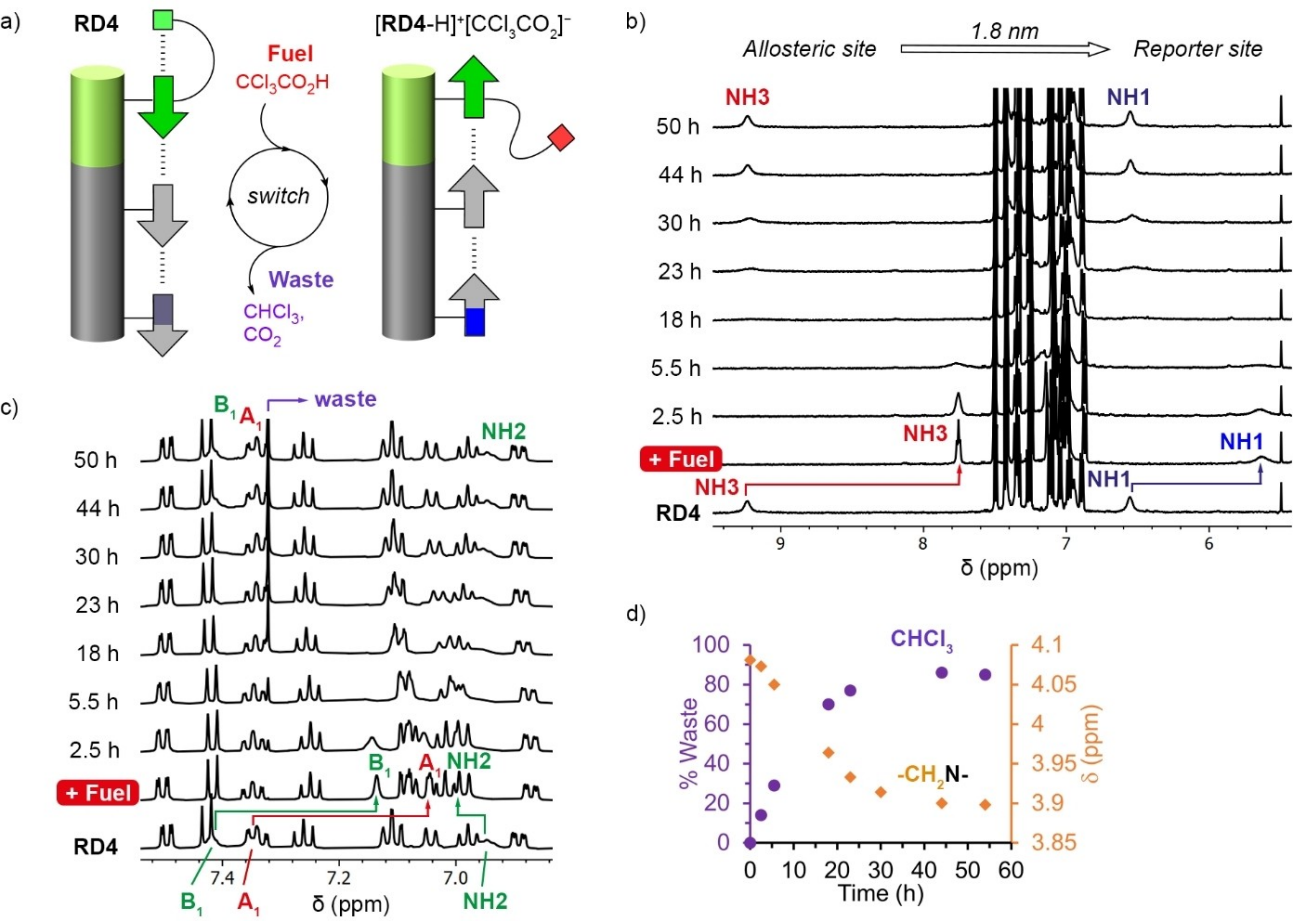
(a) Schematic representation of the conformational change induced in **RD4** by the chemical fuel CCl_3_COOH, and recovery of the original conformation upon fuel consumption and generation of CHCl_3_ waste. (b) Partial ^1^H NMR (CD_2_Cl_2_, 500 MHz, 298 K) spectra of **RD4** (1 mM), showing the effect of adding 1 equivalent of CCl_3_COOH and evolution of the mixture over time. Conformational changes at the allosteric site are shown by signal NH3, and the relay of these induced changes to the distant reporter site are reported by signal NH1. Both signals recover their original position after 44 h. (c) Aromatic regions of the ^1^H NMR (CD_2_Cl_2_, 500 MHz, 298 K) spectra of **RD4** (1 mM), showing the effect of adding 1 equivalent of CCl_3_COOH and evolution of the mixture over time. Aromatic signals A_1_ and B_1_ report on the conformational switching of **RD4**. All signals recover their original position after 44 h. The sharp singlet appearing at 7.32 ppm corresponds to generated CHCl_3_. (d) Rate of CHCl_3_ production (violet circles, determined by integration; see Figure S29 for details), and rate of change in the conformational state of **RD4** (orange diamonds), reported by the methylene signal chemical shift (next to NH3), after addition of 1 equivalent of CCl_3_COOH.

Molecular communication in cells often occurs in an out‐of‐equilibrium context, where each signaling molecule is generated and removed as part of interacting catalytic cycles. Chemical fuels can replicate this signal production/consumption. In conjunction with a functional partner, the fuel can place a molecular system in an out‐of‐equilibrium state, which then spontaneously relaxes via a dissipative process that consumes the fuel.[^48^, ^49^, ^50^] Some molecular machines have been designed to exploit these out‐of‐equilibrium conditions, using the fuel to perform a molecular motion.[^51^, ^52^, ^53^] A frequently utilized chemical fuel is trichloroacetic acid, which provides a self‐dissipative source of protons. In the presence of an amine, this acid is deprotonated to afford the corresponding ammonium and trichloroacetate. Trichloroacetate then decarboxylates over time to form the extremely basic trichloromethyl anion, which deprotonates the ammonium to regenerate the amine and produce chloroform as waste (Figures 5a and S30a).^[54]^

Given that **RD4** is acid‐base switchable, the addition of the molecular fuel CCl_3_COOH was expected to push the relay into an out‐of‐equilibrium state, which will then relax over time as the fuel is consumed (Figure 5a). The addition of 1 equivalent of the fuel to a solution of **RD4** opened the SQ‐turn module and gave [**RD4**‐H]^+^[CCl_3_CO_2_]^−^, shown by the NH3 resonance moving to a lower chemical shift that is distinct from the aromatic signals (Figure 5b and see Section 6 of the Supporting Information). Protonation of the amine group also generated a new signal NH4 (Figure S31) and shifted the NMe_2_ signal *n*. The array now switched from an antiparallel to a parallel conformation; the signal of the distant reporter NH1 underwent a clear upfield shift as it became exposed (Figure 5b), with aromatic signals A_1_ and B_1_ also shifting upfield (Figure 5c). We speculate that, as suggested for BF_4_^−^, the trichloroacetate counterion hydrogen bonds to NH3 and to NH4 of the ammonium counterion (Figure S30). The ^1^H NMR spectrum of this now out‐of‐equilibrium mixture evolves over time. First, signals for the reporter NH1 and director NH3, which are most affected by array orientation, broaden and become almost undetectable after 18 h. This suggests the system has formed a mixture of protonated and non‐protonated species that are in intermediate exchange on the ^1^H NMR spectroscopy timescale. After 23 h, the broad signals start to reappear at their original positions, indicating the mixture now has the non‐protonated **RD4** species predominating.

The spontaneous reversion of the SQ relay from parallel to antiparallel over time as the fuel is consumed is also reported by the diagnostic signals A_1_ and B_1_ (Figure 5c). After 44 h, the spectrum of **RD4** is identical to that of the initial state, and no further change is observed. However, a new resonance at 7.32 ppm has appeared, which is due to conversion of fuel into the waste, chloroform (Figure 5c and S28). Integration of this signal over time tracks the appearance of waste (Figure S29). The rate **RD4**‐H^+^ evolves into **RD4** can be monitored from the methylene protons adjacent to NH3, since this resonance conveniently remains detectable during the whole process (signal *h* in Figure S30). This reversion mirrors the rate at which the waste appears (Figure 5d), confirming that those two processes are coupled. After 50 h the system has completed a single molecular communication cycle triggered by the signaling molecule (i.e., the fuel). The “director” to “reporter” scaffolded information relay **RD4** is robust enough to respond to multiple sequential additions of fuel. The same solution of **RD4** was shown to respond in the same way to another influx of fuel without significant degradation of output signal (see Figure S33).

## Conclusion

Combining an OPE scaffold with a hydrogen bonded SQ array has provided a new method for relaying chemical information over multi‐nanometer distances. The rigid rod OPE scaffold is a key part of the design as it prevents structural collapse of the SQ information relay. A modular synthesis methodology gave access to three different trimeric OPEs of defined length, 1.8 nm for **RD2**‐**RD4**. The ethylene linkers provided sufficient conformational freedom for relay orientation to be switched between parallel and antiparallel. Spectroscopy and crystallography confirmed that the adopted orientation depended on the hydrogen bonding character of a director at one terminus.

The scaffolded relay **RD4** was shown to be responsive to a simple chemical messenger, the proton. The molecular change caused by protonation was relayed to the end of the scaffold, where it was revealed by the chemical shift of a reporter, NH1, at the far terminus. Allosteric control by pH is well‐known in proteins^[55]^ and usually leads to changes in binding or reactivity at a remote site. In contrast to Aib foldamer‐based communication systems,[^5^, ^6^, ^7^, ^8^, ^9^, ^10^] **RD4** only requires three repeating units (i.e., SQs) to cover a distance of ≈2 nm, offering fewer opportunities for defects to appear in the hydrogen‐bonded network.^[6b]^

The response of **RD4** to a chemical fuel mimics the way temporal fluctuations in signaling molecule concentration cause time‐dependent responses in an allosteric protein. In cells, signals for allosteric proteins arise and decay over time, as they are part of an out‐of‐equilibrium dissipative system. For example, Leu‐enkephalin is produced from preproenkephalin by endoproteases and degraded over time by peptidases like enkephalinase.^[56]^ The trichloroacetic acid fuel activates the **RD4** molecular communication device, both creating an out‐of‐equilibrium system and acting as an allosteric signal that is relayed down the 2 nm long **RD4** scaffold. The chemical information in the fuel is then autonomously consumed and **RD4** relaxes to its initial deactivated state. Activated and relaxed states each produce different spectroscopic outputs from the distant reporter NH1; the device then is ready for a new signal.

The versatility of SQs^[28]^ offers different approaches for the future exploitation of OPE‐SQs; for example, designing relays triggered by enzymatic reduction^[57]^ or remotely switching SQ catalysis on and off.^[58]^ Our device (**RD4**), which covers a distance similar to the lipophilic interior of a lipid bilayer,[^37^, ^38^, ^39^] shows how evolving concentrations of chemical signals can not only cause local changes but can also serve as information to be relayed to a remote site, perhaps at the far side of a membrane.^[4]^ Receipt of these signals at the remote site could in turn modulate a different type of chemical reaction at another location,[^10c^, ^59^] e.g. in a molecular factory or within a compartment,^[60]^ increasing the network complexity of the dissipative system.

## Conflict of interest

The authors declare no conflict of interest.

1

## Supporting information

As a service to our authors and readers, this journal provides supporting information supplied by the authors. Such materials are peer reviewed and may be re‐organized for online delivery, but are not copy‐edited or typeset. Technical support issues arising from supporting information (other than missing files) should be addressed to the authors.

Supporting Information

## Data Availability

The data that support the findings of this study are available in the supplementary material of this article.
