# Supplementary material for: Chemically Fueled Communication Along a Scaffolded Nanoscale Array of Squaramides
Source: Angew Chem Int Ed Engl. 2023 Aug 10;62(38):e202307841. doi: 10.1002/anie.202307841 (PMC10952809; doi:10.1002/anie.202307841)
Supplement: Supplementary file 1 — Supporting Information [file ANIE-62-0-s001.pdf]

Angewandte Chemie
Eine Zeitschrift der Gesellschaft Deutscher Chemiker

Supporting Information

Chemically Fueled Communication Along a Scaffolded Nanoscale Array of Squaramides

L. Martínez-Crespo, I. J. Vitórica-Yrezábal, G. F. S. Whitehead, S. J. Webb**

Table of contents

1. General experimental information 3

1.1. Abbreviations 4

2. Synthesis of compounds **1-19** and **RD1-4** 5

3. ^{1}H NMR experiments for compounds **16** and **RD1-4** 18

3.1. Assignment of the ^{1}H NMR spectra in CD_2Cl_2 18

3.2. VT-NMR experiments in CD_2Cl_2 25

3.3. Titrations with DMSO-d_6 in CD_2Cl_2 29

3.4. Spectra of of **RD1**-**RD4** in acetone-d_6 33

4. X-ray crystal structures 34

4.1. Crystallographic data 34

4.2. Structural representations 36

5. Addition of HBF_4 and TEA to **RD4** 38

6. Addition of CCl_3COOH to **RD4** 47

7. Dilution experiment with **RD4**. 53

8. ^{1}H, ^{13}C and selected 2D NMR spectra of **1**-**19** and **RD1**-**RD4**. 54

9. References 86

1. General experimental information

All reagents and solvents were obtained from Sigma Aldrich, Fluorochem, Alfa Aesar and VWR, and were used without further purification unless otherwise stated. Flash chromatography was performed on silica gel (Merck 60H, 40-60 nm, 230–300 mesh). Analytical thin layer chromatography (TLC) was performed on Macherey Nagel alugram SIL G/UV254 TLC sheets and TLC plates were visualized by UV irradiation (254 nm).

NMR spectra were recorded in deuterated solvents using either Brüker AVANCE 400 MHz or Brüker AVANCE 500 MHz spectrometers. Chemical shifts (δ) are quoted in parts per million (ppm) and coupling constants (J) are quoted in Hz to the nearest 0.5 Hz. ^{1}H NMR spectra were referenced to the residual deuterated solvent peak ($CHDCl_2$: 5.32, $CHCl_3$: 7.26; CHD_2CN: 1.94 ppm, DMSO-d_5: 2.50, acetone-d_5: 2.05), unless stated otherwise.[S1] ^{13}C NMR spectra were referenced to the resonance of the solvent (CD_2Cl_2: 53.8, $CDCl_3$: 77.2, CD_3CN: 118.3 ppm).[S1]

High-resolution mass spectra (HRMS) were recorded by staff at the University of Manchester, on a Thermo Q-Exactive and are accurate to ±0.001 Da.

1.1. Abbreviations

- AcOEt: ethyl acetate
- Boc: *tert*-butyloxycarbonyl
- COSY: Correlated Spectroscopy
- DCM: dichloromethane
- DMF: *N*,*N*-dimethylformamide
- DIPA: *N,N*-di(*iso*-propyl)amine
- DIPEA: *N,N*-di(*iso*-propyl)ethylamine
- DMSO: dimethylsulfoxide
- EtOH: ethanol
- Et_2O: diethyl ether
- EXSY: Exchange spectroscopy
- HMBC: Heteronuclear Multiple Bond Correlation
- HRMS: High-resolution mass spectrometry
- HSQC: Heteronuclear Single Quantum Coherence
- MeOH: methanol
- NMR: Nuclear magnetic resonance
- NOESY: Nuclear Overhauser Effect Spectroscopy
- ppm: parts per million
- RD: rod
- SQ: squaramide
- TEA: triethylamine
- VT: Variable temperature

2. Synthesis of compounds 1-19 and RD1-4

Scheme S1. Synthesis of precursors **1-15** and final compounds **16**, **RD1** and **RD2**.

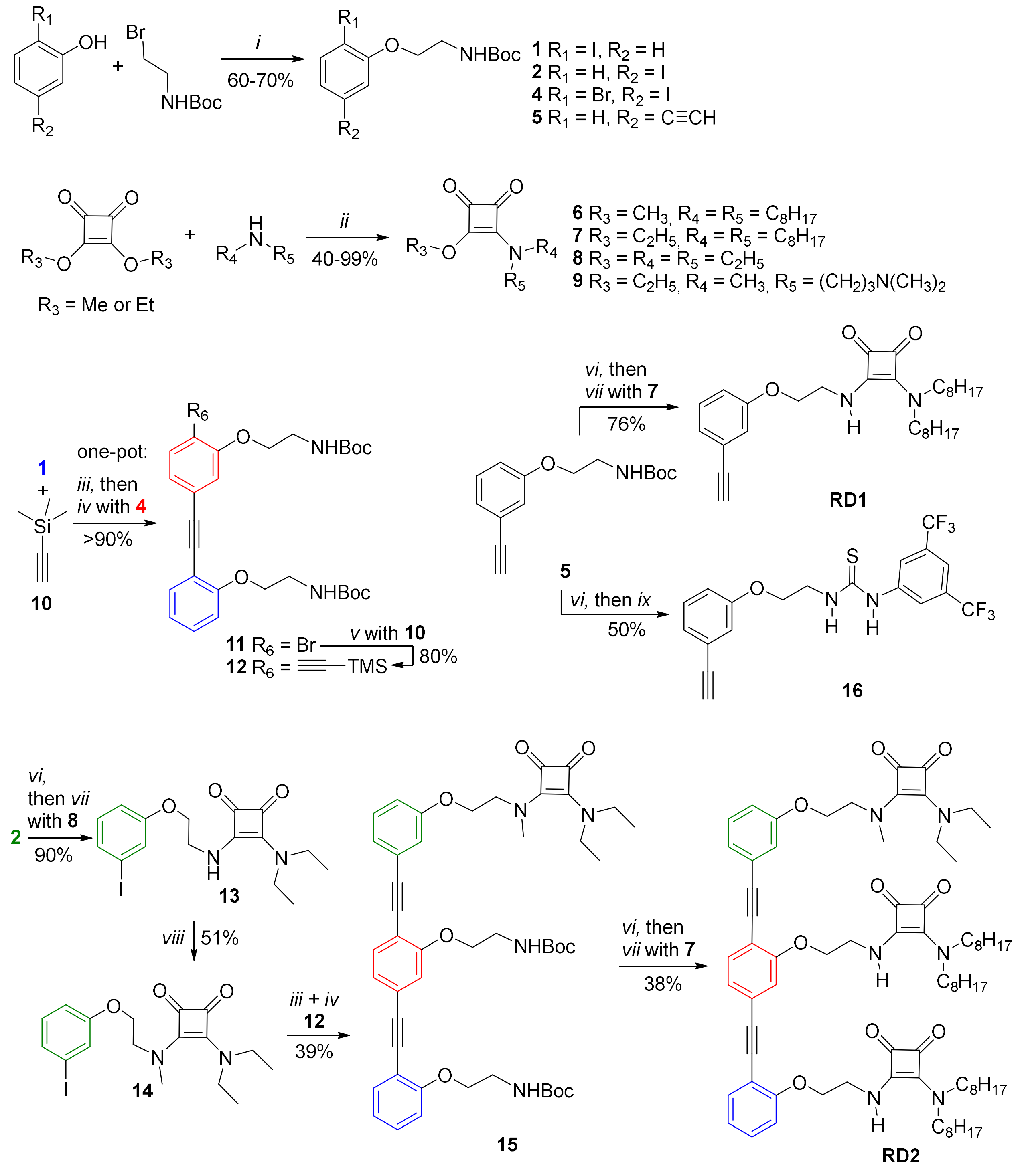

i. K_2CO_3 (1.5 eq.), DMF, 16 h; *ii.* MeOH or Et_2O, r.t., 1-16 h; *iii.* $Pd(PPh_3)_2Cl_2$, CuI, DIPA, r.t., 1.5 h; *iv.* add H_2SiF_6, H_2O, r.t., 16 h; *v.* $Pd(PPh_3)_2Cl_2$, CuI, Piperidine, 60°C, 16 h; *vi.* DCM-TFA 9-1, r.t., 1-2 h; *vii.* DIPEA or TEA, MeOH, r.t., 16-72 h; *viii.* MeI, NaH, DMF, 0°C to r.t., 0.5 h; *ix.* DCM, 3,5-$(CF_3)_2C_6H_3$-NCS, r.t., 16 h.

Scheme S2. Synthesis of precursors **17-19** and final compounds **RD3** and **RD4**.

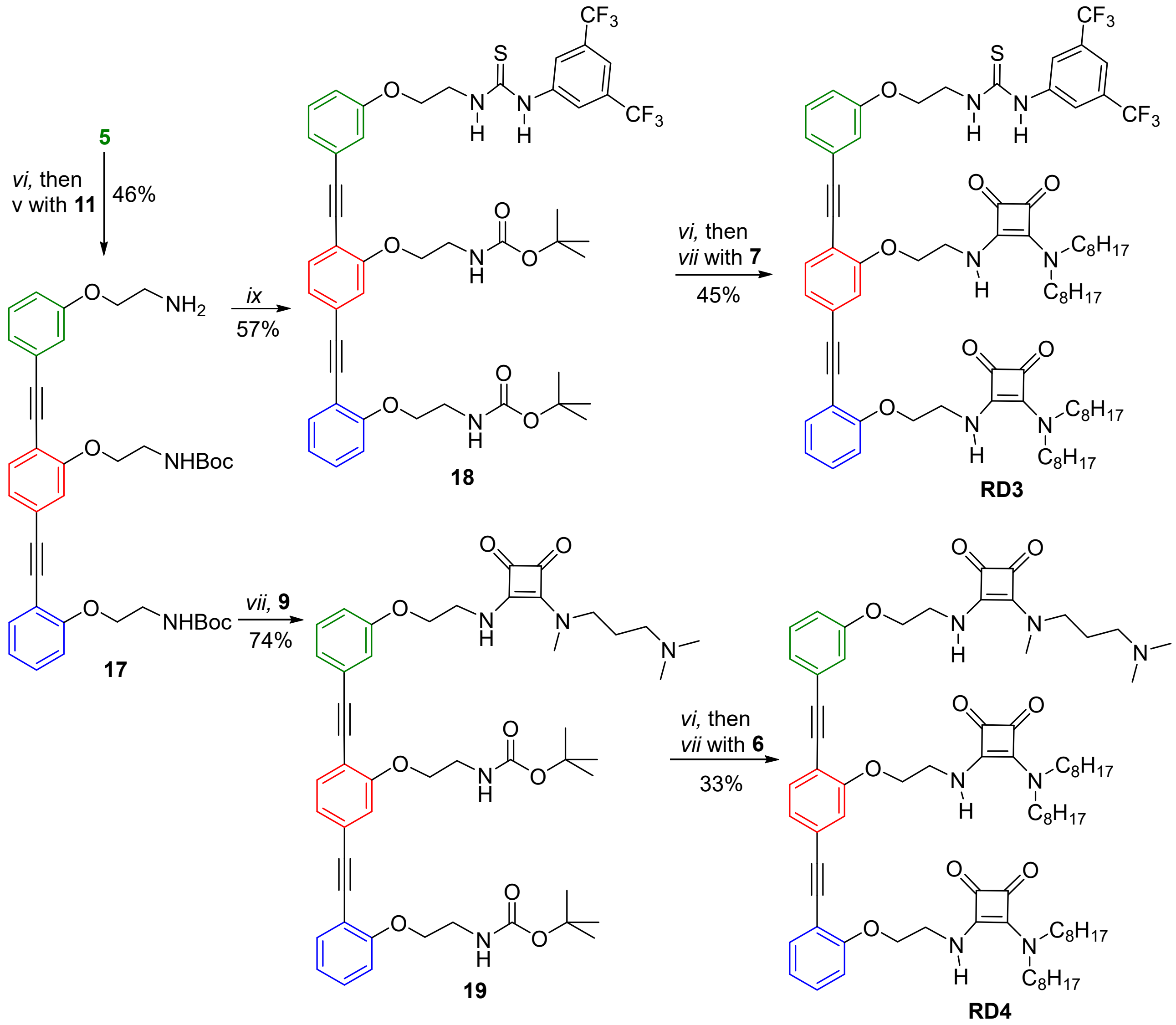

v. Pd(PPh$_3$)$_2$Cl$_2$, CuI, Piperidine, 60°C, 16 h; *vi.* DCM-TFA 9-1, r.t., 1-2 h; *vii.* DIPEA or TEA, MeOH, r.t., 16-72 h; *ix.* DCM, *3,5*-(CF$_3$)$_2$C$_6$H$_3$-NCS, r.t., 16 h.

***tert*-Butyl (2-(2-iodophenoxy)ethyl)carbamate (1)**

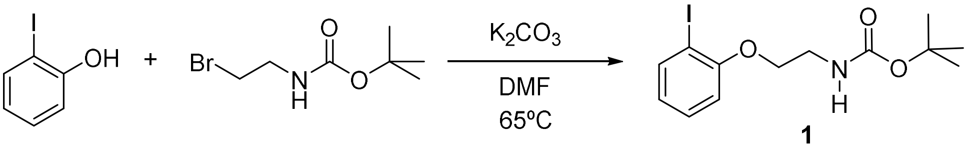

1

2-Iodophenol (2000 mg, 9.09 mmol) and 2-(Boc-amino)ethyl bromide (3055 mg, 13.64 mmol) were dissolved in anhydrous DMF (3 mL), K_2CO_3 (16.5 mg, 0.15 mmol) was added (in suspension) and the resulting mixture was stirred at 65°C and under an argon atmosphere for 16 h. DCM (50 mL) was added to the reaction mixture and the solution was filtered, washed with HCl (1 M, 2 × 35 mL) and brine (30 mL), and then dried with $MgSO_4$. The solvent was removed under reduced pressure and the crude was purified by column chromatography (SiO_2, DCM) to afford the product as a white solid (2220 mg, 67%). ***Rf***: 0.25 (DCM). **^{1}H NMR** (400 MHz, $CDCl_3$): δ = 7.77 (d, *J* = 7.7 Hz, 1H), 7.29 (t, *J* = 8.2 Hz, 1H), 6.81 (d, *J* = 8.2 Hz, 1H), 6.73 (t, *J* = 7.7 Hz, 1H), 5.12 (br, 1H), 4.07 (t, *J* = 5.0 Hz, 2H), 3.59 (q, *J* = 5.0 Hz, 2H), 1.46 (s, 9H). **^{13}C NMR** (100 MHz, $CDCl_3$): δ = 157.0, 155.9, 139.4, 129.6, 123.0, 112.5, 86.8, 79.6, 68.7, 40.0, 28.4. **HRMS** (ESI+): calcd for $C_{13}H_{18}O_3NINa$ $[M+Na]^+$: 386.0224, found: 386.0216.

***tert*-Butyl (2-(3-iodophenoxy)ethyl)carbamate (2)**

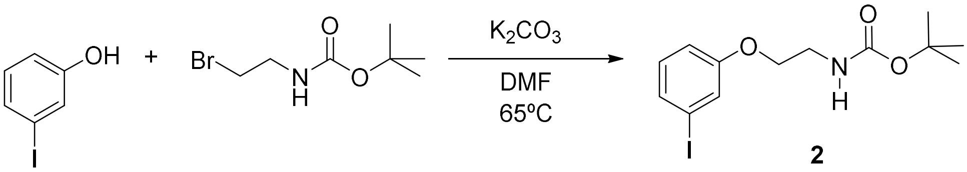

2

3-Iodophenol (3000 mg, 13.64 mmol) and 2-(Boc-amino)ethyl bromide (4580 mg, 20.45 mmol) were dissolved in anhydrous DMF (3.5 mL), K_2CO_3 (2800 mg, 20.45 mmol) was added (in suspension) and the resulting mixture was stirred at 65°C under an argon atmosphere for 16 h. DCM (50 mL) was added to the reaction mixture and the solution was filtered, washed with HCl (1 M, 2 × 35 mL) and brine (30 mL), and then dried with $MgSO_4$. The solvent was removed under reduced pressure and the crude was purified by column chromatography (SiO_2, DCM) to afford the product as a pale oil (3930 mg, 79%). ***Rf***: 0.5 (DCM). **^{1}H NMR** (400 MHz, $CDCl_3$): δ = 7.29 (d, *J* = 7.7 Hz, 1H), 7.24 (s, 1H), 6.99 (t, *J* = 8.3 Hz, 1H), 6.85 (d, *J* = 8.3 Hz, 1H), 4.98 (s, 1H), 3.98 (t, *J* = 5.0 Hz, 2H), 3.51 (q, *J* = 5.0 Hz, 2H), 1.45 (s, 9H). **^{13}C NMR** (100 MHz, $CDCl_3$): δ = 159.1, 155.9, 130.9, 130.2, 128, 114.0, 94.4, 79.6, 67.4, 40.0, 28.4. **HRMS** (ESI+): calcd for $C_{13}H_{18}O_3NINa$ $[M+Na]^+$: 386.0224, found: 386.0243.

2-Bromo-5-iodophenol (3)[S2]

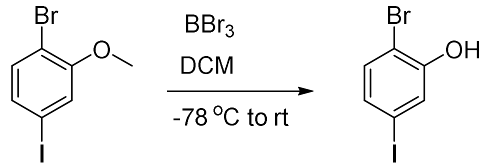

3

This compound was prepared as previously reported,[S2] with a modified purification method. Under an argon atmosphere, anhydrous DCM (30 mL) was added to 2-bromo-5-iodoanisol (2000 mg, 5.40 mmol, 87% purity) and the solution was cooled to −78°C. A solution of BBr_3 in DCM (1 M, 10 mL, 10 mmol) was added dropwise to the cold mixture and when the addition was completed the mixture was stirred for one more hour at −78°C. Water (40 mL) was added to quench the reaction. The product was extracted with diethyl ether (20 mL) and the organic phase was further washed with water (40 mL). The solvent was removed under reduced pressure and the crude was purified by column chromatography (SiO_2, DCM-hexane 1:1) to afford the product as a brown solid (1346 mg, 83%). ***Rf***: 0.4 (DCM-hexane 1:1). **^{1}H NMR** (400 MHz, $CDCl_3$): δ = 7.37 (d, *J* = 1.9 Hz, 1H), 7.18 (s, 0.2H), 7.16 (s, 0.8H), 7.14 (d, *J* = 1.9 Hz, 0.8H), 7.12 (d, *J* = 1.9 Hz, 0.2H), 5.49 (s, 1H). **HRMS** (ESI−): calcd for C_6H_3OBrI $[M-H]^-$: 296.8417, found: 296.8395.

tert-Butyl (2-(2-bromo-5-iodophenoxy)ethyl)carbamate (4)

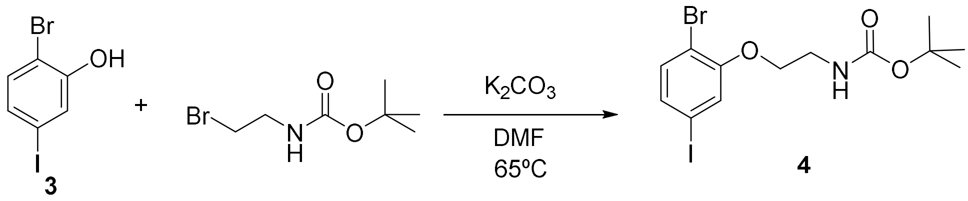

Compound **3** (1346 mg, 4.50 mmol) and 2-(Boc-amino)ethyl bromide (1600 mg, 7.20 mmol) were dissolved in anhydrous DMF (4 mL), K_2CO_3 (995 mg, 7.20 mmol) was added (in suspension) and the resulting mixture was stirred at 65°C under an argon atmosphere for 16 h. DCM (50 mL) was added to the reaction mixture and the solution was filtered, washed with HCl (0.3 M, 2 × 30 mL) and dried with $MgSO_4$. The solvent was removed under reduced pressure and the crude was purified by column chromatography (SiO_2, DCM) to afford the product as a white solid (1660 mg, 83%). $\boldsymbol{R_f}$: 0.6 (DCM). **^{1}H NMR** (400 MHz, $CDCl_3$): δ = 7.27 (mult., 1H), 7.17 (mult., 2H), 5.03 (br, 1H), 4.05 (t, J = 5.0 Hz, 2H), 3.58 (q, J = 5.0 Hz, 2H), 1.45 (s, 9H). **^{13}C NMR** (100 MHz, $CDCl_3$): δ = 155.8, 155.5, 134.5, 131.4, 122.7, 112.4, 92.4, 79.7, 69.0, 40.0, 28.3. **HRMS** (ESI+): calcd for $C_{13}H_{17}O_3NIBrNa$ $[M+Na]^+$: 463.9329, found: 463.9333.

tert-Butyl (2-(3-ethynylphenoxy)ethyl)carbamate (5)

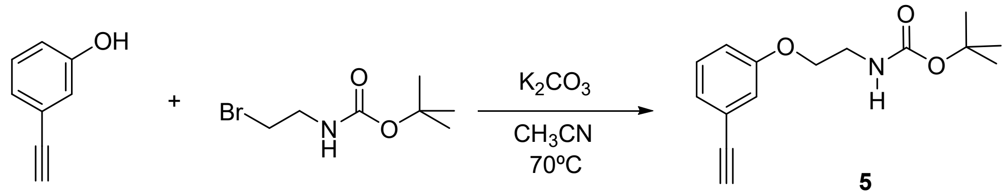

3-Ethynylphenol (1000 mg, 8.47 mmol) and 2-(Boc-amino)ethyl bromide (2845 mg, 12.70 mmol) were dissolved in anhydrous DMF (2 mL), K_2CO_3 (1755 mg, 12.70 mmol) was added (in suspension) and the resulting mixture was stirred at 65°C under an argon atmosphere for 16 h. DCM (50 mL) was added to the reaction mixture and the solution was filtered, washed with HCl (0.3 M, 2 × 30 mL) and brine (30 mL), and then dried with $MgSO_4$. The solvent was removed under reduced pressure and the crude was purified by column chromatography (SiO_2, DCM) to afford the product as a colourless oil (1600 mg, 72%). $\boldsymbol{R_f}$: 0.3 (DCM). **^{1}H NMR** (400 MHz, $CDCl_3$): δ = 7.23 (t, J = 8.0 Hz, 1H), 7.10 (d, J = 7.7 Hz, 1H), 7.01 (s, 1H), 6.89 (d, J = 8.3 Hz, 1H), 4.96 (br, 1H), 4.01 (t, J = 5.1 Hz, 2H), 3.53 (q, J = 5.1 Hz, 2H), 3.06 (s, 1H), 1.45 (s, 9H). **^{13}C NMR** (100 MHz, $CDCl_3$): δ = 158.3, 129.5, 125.0, 123.2, 117.7, 115.7, 83.4, 79.6, 77.2, 67.3, 40.1, 28.4. **HRMS** (ESI+): calcd for $C_{15}H_{19}O_3NNa$ $[M+Na]^+$: 284.1257, found: 284.1256.

3-(Di(_n_-octyl)amino)-4-methoxycyclobut-3-ene-1,2-dione (6)

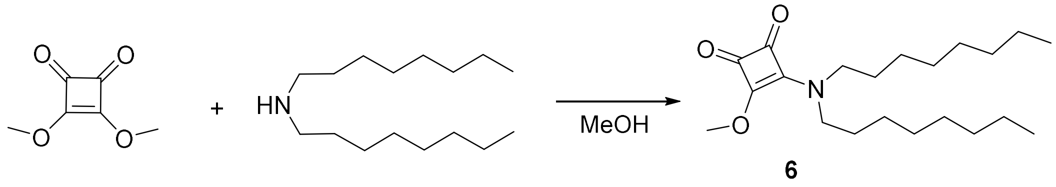

Dimethyl squarate (600 mg, 4.22 mmol) was partially dissolved in MeOH (5 mL) and a solution of _n_-octylamine (1.4 mL, 4.22 mmol) in MeOH (5 mL) was added dropwise. The mixture was stirred for 1 h and then filtered. The solvent was removed under reduced pressure and the crude was purified by column chromatography (SiO_2, DCM-AcOEt(5%)) to afford the product as a pale oil (610 mg, 40%). $\boldsymbol{R_f}$: 0.3 (DCM-AcOEt(5%)). **^{1}H NMR** (400 MHz, $CDCl_3$): δ = 4.39 (s, 3H), 3.65 (t, J = 7.4 Hz, 2H), 3.35 (t, J = 7.4 Hz, 2H), 1.59 (mult., 4H), 1.29 (mult., 20H), 0.89 (mult., 6H). **^{13}C NMR** (100 MHz, DMSO-d_6): δ = 188.8, 182.4, 176.1, 171.8, 60.2, 49.3, 48.7, 31.6, 29.1, 29.05, 29.0, 28.7, 28.3, 26.6, 26.2, 22.5, 13.9. **HRMS** (ESI+): calcd for $C_{21}H_{37}O_3NNa$ $[M+Na]^+$: 374.2666, found: 374.2656.

3-(Di(*n*-octyl)amino)-4-ethoxycyclobut-3-ene-1,2-dione (7)

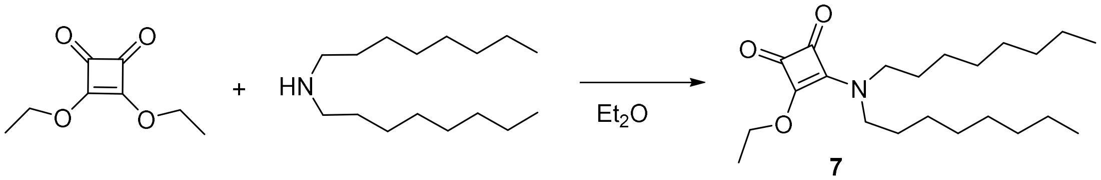

7

Diethyl squarate (300 mg, 1.71 mmol) was dissolved in Et_2O (3 mL) and a solution of *n*-octylamine (0.516 mL, 1.60 mmol) in Et_2O (3 mL) was added dropwise. The mixture was stirred for 16 h. The solvent was removed under reduced pressure and the crude was purified by column chromatography (SiO_2, DCM-AcOEt(2%)) to afford the product as a pale oil (580 mg, 99%). $\boldsymbol{R_f}$: 0.2 (DCM-AcOEt(3%)). **^{1}H NMR** (400 MHz, $CDCl_3$): δ = 4.76 (q, J = 7.1 Hz, 2H), 3.64 (t, J = 7.3 Hz, 2H), 3.36 (t, J = 7.3 Hz, 2H), 1.58 (mult., 4H), 1.43 (t, J = 7.1 Hz, 3H), 1.28 (mult., 20H), 0.88 (mult., 6H). **^{13}C NMR** (100 MHz, $CDCl_3$): δ = 188.8, 182.4, 175.9, 172.2, 69.4, 49.3, 48.9, 31.7, 29.2, 29.1, 28.8, 28.5, 26.4, 26.3, 22.6, 15.8, 14.1. **HRMS** (ESI+): calcd for $C_{22}H_{39}O_3NNa$ $[M+Na]^+$: 388.2822, found: 388.2813.

3-(Diethylamino)-4-ethoxycyclobut-3-ene-1,2-dione (8)[S3]

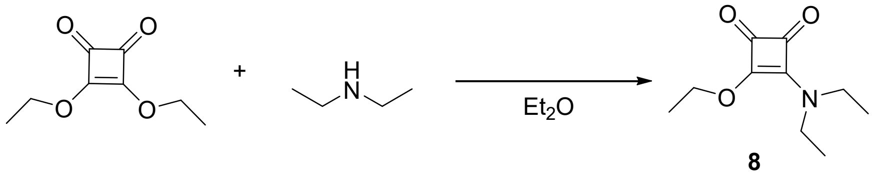

8

Diethyl squarate (300 mg, 1.71 mmol) was dissolved in Et_2O (5 mL) and a solution of diethylamine (0.180 mL, 1.71 mmol) in Et_2O (10 mL) was added dropwise. The mixture was stirred for 16 h. The solvent was removed under reduced pressure and the crude dried under high vacuum for 7 h to afford the product as a white solid (573 mg, 92%). $\boldsymbol{R_f}$: 0.2 (DCM-AcOEt(10%)). **^{1}H NMR** (400 MHz, $CDCl_3$): δ = 4.70 (q, J = 7.1 Hz, 2H), 3.67 (q, J = 7.2 Hz, 2H), 3.39 (q, J = 7.2 Hz, 2H), 1.38 (t, J = 7.1 Hz, 3H), 1.18 (t, J = 7.2 Hz, 6H). **^{13}C NMR** (100 MHz, $CDCl_3$): δ = 188.9, 182.3, 176.3, 171.5, 69.5, 44.1, 43.8, 15.9, 14.6, 14.3. The NMR characterization data are in agreement with those previously reported for this compound.[S3]

3-((3-(Dimethylamino)propyl)(methyl)amino)-4-ethoxycyclobut-3-ene-1,2-dione (9)[S4]

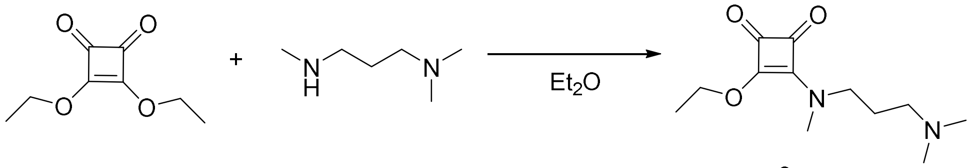

9

Diethyl squarate (300 mg, 1.71 mmol) was dissolved in Et_2O (2 mL) and a solution of *N*,*N*,*N*'-trimethylpropan-1,3-diamine (0.264 mL, 1.71 mmol) in Et_2O (3 mL) was added dropwise. The mixture was stirred for 16 h and then filtered. The solvent was removed under reduced pressure to afford the product as a pale oil (583 mg, 93%). **^{1}H NMR** (400 MHz, $CDCl_3$): δ = 4.76 (q, J = 7.1 Hz, 2H), 3.73 (t, J = 7.1 Hz, 1H), 3.45 (t, J = 7.1 Hz, 1H), 3.34 (s, 1H), 3.17 (s, 2H), 2.30 (br, 5H), 2.23 (s, 3H), 1.82 (mult., 2H), 1.46 (mult., 3H). The ^{1}H NMR characterization data are in agreement with those previously reported for this compound.[S4]

3-(Di(*n*-octyl)amino)-4-((2-(3-ethynylphenoxy)ethyl)amino)cyclobut-3-ene-1,2-dione (**RD1**)

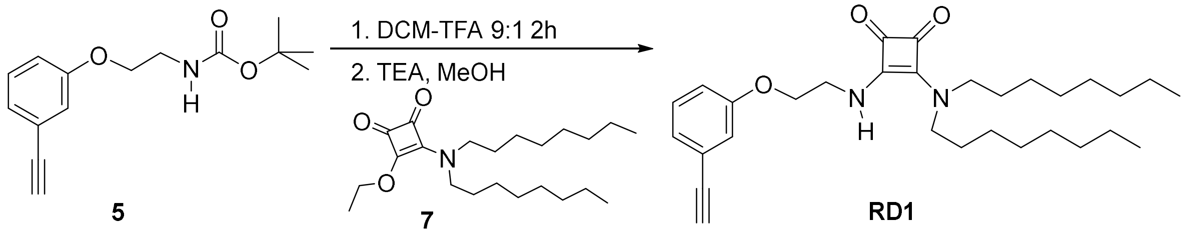

Compound **5** (100 mg, 0.38 mmol) was dissolved in a 9:1 mixture of DCM and TFA (5 mL) and the solution was stirred at room temperature for 2 h. The solvents were removed under reduced pressure. The crude was redissolved in MeOH (0.5 mL), TEA (270 µL, 1.92 mmol) and a solution of **7** (140 mg, 0.38 mmol) in MeOH (0.5 mL) were added and the mixture was stirred for 72 h. The solvent was removed under reduced pressure and the crude was purified by column chromatography (SiO_2, DCM-AcOEt(15%)) to afford the product as a white solid (135 mg, 73%). ***R_f***: 0.3 (DCM-AcOEt(15%)). **^{1}H NMR** (400 MHz, CD_2Cl_2): δ = 7.28 (t, *J* = 8.0 Hz, 1H), 7.14 (d, *J* = 7.6 Hz, 1H), 7.06 (s, 1H), 6.95 (d, *J* = 8.3 Hz, 1H), 5.68 (br, 1H), 4.17 (mult., 4H), 3.47 (br, 4H), 3.16 (s, 1H), 1.63 (mult., 4H), 1.28 (mult., 20H), 0.91 (mult., 6H). **^{13}C NMR** (101 MHz, CD_2Cl_2): δ = 183.7, 182.8, 168.2, 166.4, 158.2, 129.6, 125.1, 123.2, 117.8, 115.5, 83.1, 77.2, 67.9, 49.8, 43.6, 31.8, 29.6, 29.3, 29.2, 26.5, 22.6, 13.8. **HRMS** (ESI+): calcd for $C_{30}H_{44}O_3N_2Na$ $[M+Na]^+$: 503.3244, found: 503.3242.

***tert*-Butyl (2-(2-((4-bromo-3-(2-((*tert*-butoxycarbonyl)amino)ethoxy)phenyl)ethynyl)phenoxy)ethyl)-carbamate** (**11**)

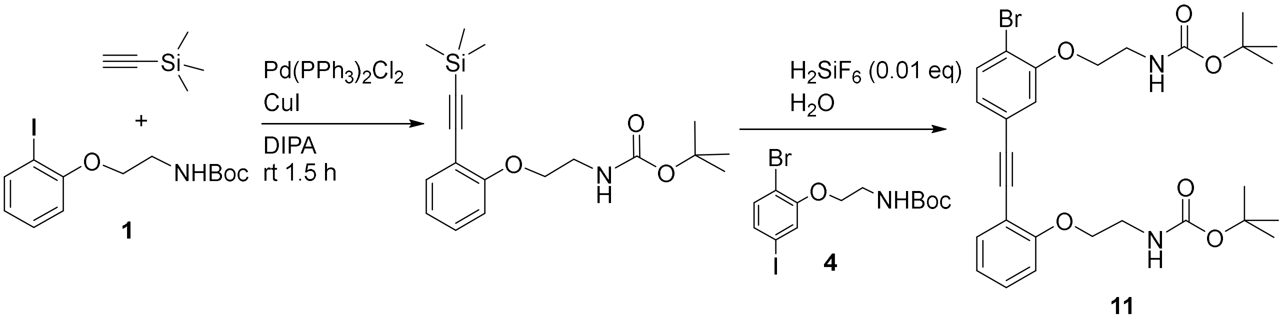

Compound **1** (820 mg, 2.26 mmol), bis(triphenylphosphine)palladium(II) dichloride (160 mg, 0.23 mmol) and copper iodide (43 mg, 0.23 mmol) were placed in a round bottom flask with an inert atmosphere. Anhydrous di(*iso*-propyl)amine (8 mL) and trimethylsilylacetylene **10** (0.344 mL, 2.48 mmol) were added and the mixture was stirred for 1.5 h at room temperature. Then, compound **4** (998 mg, 2.26 mmol), an aqueous solution of fluorosilicic acid (0.010 mL, 0.023 mmol, 34% w/w) and water (0.406 mL, 22.58 mmol) were added and the resulting mixture was stirred for 16 h at room temperature. DCM (5 mL) was added to the mixture and the resulting solution was filtered. The solvents were removed under reduced pressure and the crude was purified by column chromatography (SiO_2, DCM-AcOEt(3%)) to afford the product as a brown solid (1200 mg, 92%). ***R_f***: 0.4 (DCM-AcOEt(3%)). **^{1}H NMR** (400 MHz, CD_3CN): δ = 7.50 (d, *J* = 8.1 Hz, 1H), 7.42 (d, *J* = 7.5 Hz, 1H), 7.31 (t, *J* = 7.9 Hz, 1H), 7.12 (s, 1H), 7.00 (d, *J* = 8.1 Hz, 1H), 6.97 (d, *J* = 8.5 Hz, 1H), 6.93 (t, *J* = 7.5 Hz, 1H), 5.42 (s, 2H), 4.07 (mult., 4H), 3.40 (mult., 4H), 1.35 (s, 9H), 1.30 (s, 9H). **^{13}C NMR** (100 MHz, CD_2Cl_2): δ = 159.2, 155.8, 154.9, 133.3, 133.2, 130.2, 125.4, 123.9, 121.1, 116.2, 112.7, 112.6, 92.6, 86.7, 40.1, 39.9, 28.2, 28.1. **HRMS** (ESI+): calcd for $C_{28}H_{35}BrN_2O_6Na$ $[M+Na]^+$: 597.1571, found: 597.1569.

tert-Butyl (2-(2-((3-(2-((_tert_-butoxycarbonyl)amino)ethoxy)-4-((trimethylsilyl)ethynyl)phenyl)ethynyl)-phenoxy)ethyl)carbamate (12)

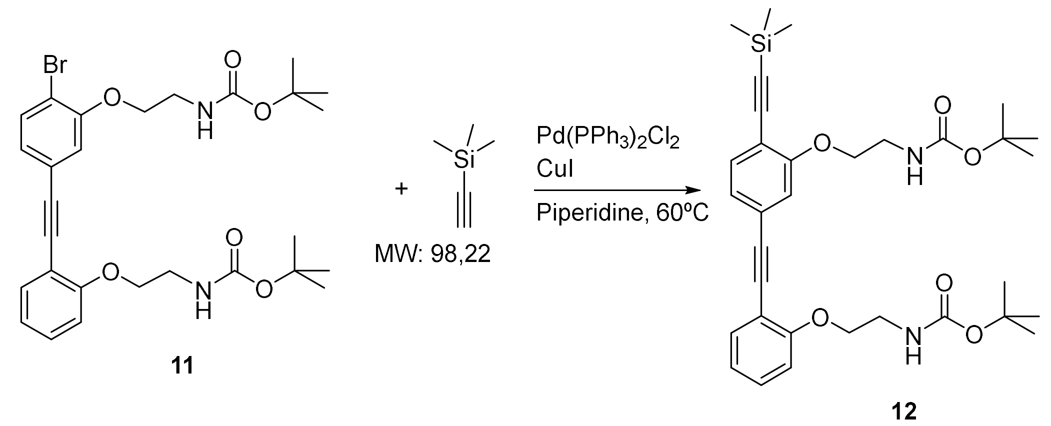

Compound **11** (400 mg, 0.70 mmol), bis(triphenylphosphine)palladium(II) dichloride (99 mg, 0.14 mmol) and copper iodide (20 mg, 0.10 mmol) were placed in a round bottom flask with an inert atmosphere. Anhydrous piperidine (5 mL) and trimethylsilylacetylene (0.240 mL, 1.74 mmol) were added and the mixture was stirred for 2 h at 60°C. The solvent was removed under reduced pressure and the crude was purified by column chromatography (SiO_2, DCM-AcOEt(3%)) to afford the product as a yellow solid (330 mg, 80%). ***R**$_f$*: 0.2 (DCM-AcOEt(3%)). **^{1}H NMR** (400 MHz, CD_3CN): δ = 7.47 (d, *J* = 7.2 Hz, 1H), 7.35 (mult., 2H), 7.12 (mult., 2H), 7.00 (mult., 2H), 5.50 (s, 1H), 5.39 (s, 1H), 4.12 (mult., 4H), 3.45 (br., 4H), 1.41 (s, 9H), 1.36 (s, 9H), 0.25 (s, 9H). **^{13}C NMR** (100 MHz, CD_3CN): δ = 156.9, 159.8, 156.4, 156.3, 133.9, 133.8, 131.1, 125.6, 124.7, 121.6, 116.0, 113.4, 113.3, 112.7, 101.4, 100.8, 93.2, 88.3, 79.2, 79.0, 68.4, 68.3, 40.3, 40.2, 28.2, 28.1, −0.39. **HRMS** (ESI+): calcd for $C_{33}H_{44}SiN_2O_6Na$ $[M+Na]^+$: 615.2861, found: 615.2857.

3-(Diethylamino)-4-((2-(3-iodophenoxy)ethyl)amino)cyclobut-3-ene-1,2-dione (13)

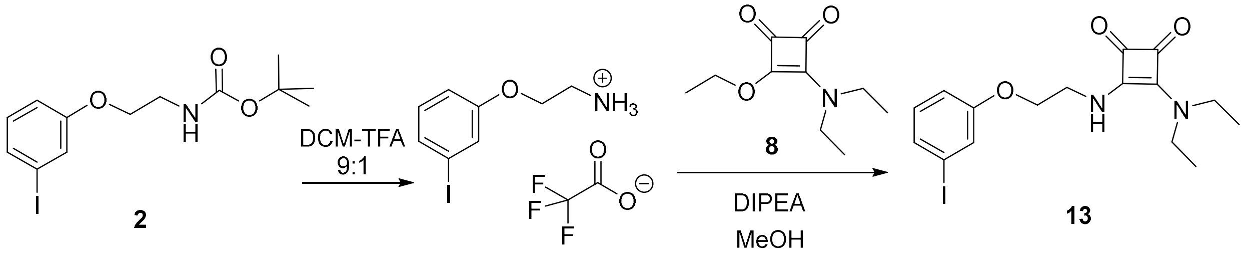

Compound **2** (600 mg, 1.65 mmol) was dissolved in a 9:1 mixture of DCM and TFA (5 mL) and the solution was stirred at room temperature for 2 h. The solvents were removed under reduced pressure. The crude was redissolved in MeOH (1.5 mL), then DIPEA (0.885 mL, 5.08 mmol) and a solution of **8** (250 mg, 1.27 mmol) in MeOH (0.5 mL) were added and the mixture was stirred for 16 h. A solid that appeared in suspension was separated from the solution by filtration, washed with MeOH and dried to afford the product as a white solid (472 mg, 90%). **^{1}H NMR** (400 MHz, DMSO-d_6): δ = 7.66 (br, 1H), 7.26 (mult., 2H), 7.03 (t, *J* = 8.2 Hz, 1H), 6.93 (d, *J* = 8.2 Hz, 1H), 4.08 (t, *J* = 5.6 Hz, 2H), 3.86 (q, *J* = 5.6 Hz, 2H), 3.47 (br, 4H), 1.08 (t, 6H). **^{13}C NMR** (100 MHz, DMSO-d_6): δ = 182.6, 181.7, 166.9, 166.8, 159.0, 131.3, 129.5, 123.0, 114.4, 94.9, 67.7, 43.4, 42.5, 14.9. **HRMS** (ESI+): calcd for $C_{16}H_{19}IN_2O_3Na$ $[M+Na]^+$: 437.0333, found: 437.0326.

3-(Diethylamino)-4-((2-(3-iodophenoxy)ethyl)(methyl)amino)cyclobut-3-ene-1,2-dione (14)

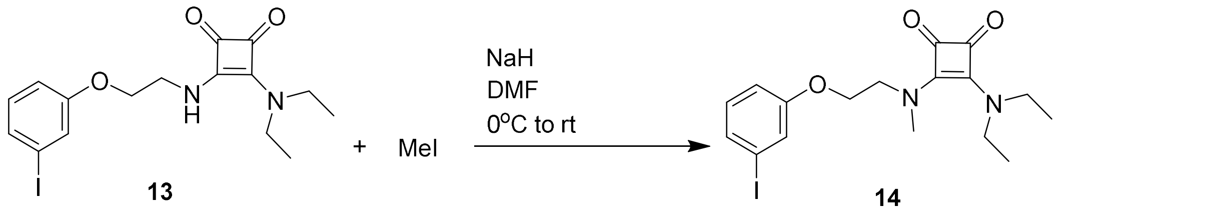

A solution of **13** (200 mg, 0.48 mmol) in anhydrous *N*,*N*-dimethylformamide (6 ml) was added to a stirred cold (0°C) suspension of 60% sodium hydride (23 mg, 0.58 mmol) in anhydrous *N*,*N*-dimethylformamide (1 mL) under argon. The mixture was stirred for an additional 30 min at 0°C. Then iodomethane (0.042 mL, 0.68 mmol) was added and the ice bath was removed. The reaction was led to stand for 30 min. After this period, the reaction mixture was poured into hydrochloric acid solution (1 M, 20 mL), extracted with DCM (2 × 20 mL), washed with HCl (1 M, 2 × 15 mL) and brine (30 mL), and dried with $MgSO_4$. The solvent was removed under reduced pressure and the crude was redissolved in Et_2O (15 mL) and stored at −18°C for 4 hours. The solid that precipitated (identified as residual **13**) was separated by decantation, then more Et_2O (10 mL) was added to the solution. The solution was washed with HCl (1 M, 2 × 20 mL) and brine (20 mL), and dried with $MgSO_4$. The solvent was removed under reduced pressure and the mixture was purified by column chromatography (SiO_2, DCM-AcOEt(30%)) to afford the product as a white solid (105 mg, 51%). ***R****f*: 0.3 (DCM-AcOEt(30%)). **^{1}H NMR** (400 MHz, $CDCl_3$): δ = 7.29 (d, *J* = 7.9 Hz, 1H), 7.23 (s, 1H), 6.99 (t, *J* = 7.9 Hz, 1H), 6.84 (d, *J* = 8.3 Hz, 1H), 4.17 (t, *J* = 4.5 Hz, 2H), 4.09 (t, *J* = 4.5 Hz, 2H), 3.58 (q, *J* = 7.1 Hz, 4H), 3.26 (s, 3H), 1.23 (t, *J* = 7.1 Hz, 6H). **^{13}C NMR** (100 MHz, $CDCl_3$): δ = 184.4, 183.9, 169.2, 169.1, 158.8, 130.9, 130.4, 123.7, 113.9, 94.4, 67.5, 51.8, 44.8, 41.5, 14.0. **HRMS** (ESI+): calcd for $C_{17}H_{21}O_3N_2INa$ $[M+Na]^+$: 451.0476, found: 451.0479.

***tert*-Butyl (2-(2-((3-(2-((*tert*-butoxycarbonyl)amino)ethoxy)-4-((3-(2-((2-(diethylamino)-3,4-dioxocyclobut-1-en-1-yl)(methyl)amino)ethoxy)phenyl)ethynyl)phenyl)ethynyl)phenoxy)ethyl)carbamate (15)**

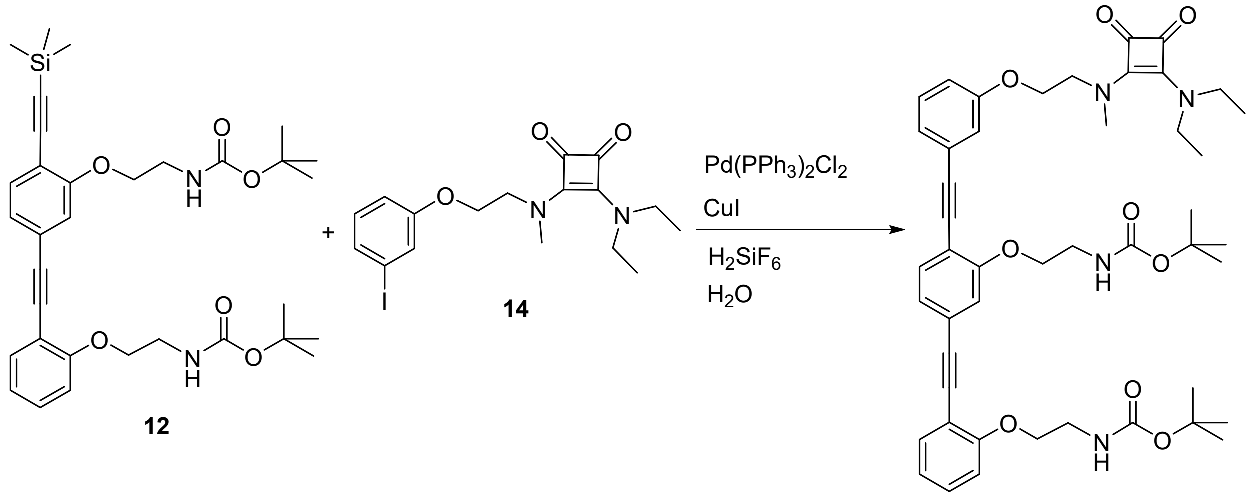

Compounds **12** (130 mg, 0.22 mmol), **14** (94 mg, 0.22 mmol), THF (0.1 mL) bis(triphenylphosphine)palladium(II) dichloride (16 mg, 0.02 mmol) and copper iodide (4 mg, 0.02 mmol) were placed in a round bottom flask with an inert atmosphere. Di(*iso*-propyl)amine (8 mL), an aqueous solution of fluorosilicic acid (0.001 mL, 0.002 mmol, 34% w/w) and water (0.039 mL, 2.193 mmol) were added and the resulting mixture was stirred for 16 h at room temperature. The solvents were removed under reduced pressure and the crude was purified by column chromatography (SiO_2, DCM-AcOEt(15%)) to afford the product as a brown solid (70 mg, 39%). ***R****f*: 0.3 (DCM-AcOEt(40%)). **^{1}H NMR** (400 MHz, acetone-*d*6): δ = 7.52 (d, *J* = 7.7 Hz, 1H), 7.50 (d, *J* = 7.8 Hz, 1H), 7.40 (t, *J* = 7.7 Hz, 1H), 7.34 (t, *J* = 7.8 Hz, 1H), 7.27 (s, 1H), 7.21 (mult., 3H), 7.13 (d, *J* = 8.3 Hz, 1H), 7.02 (mult., 2H), 6.16 (br, 2H), 4.37 (t, *J* = 5.3 Hz, 2H), 4.27 (t, *J* = 5.7 Hz, 2H), 4.20 (t, *J* = 5.7 Hz, 2H), 4.16 (t, *J* = 5.3 Hz, 2H), 3.65 (q, *J* = 7.1 Hz, 4H), 3.58 (mult., 4H), 3.37 (s, 3H), 1.41 (s, 18H), 1.25 (t, *J* = 7.1 Hz, 6H). **^{13}C NMR** (100 MHz, acetone-*d*6): δ = 184.5, 184.0, 170.0, 169.1, 159.6, 159.2,

158.6, 155.8, 133.2, 133.1, 130.4, 129.7, 125.1, 124.6, 124.4, 124.1, 120.8, 117.1, 115.4, 115.3, 113.0, 112.7, 112.5, 94.7, 93.0, 87.8, 85.6, 78.0, 67.9, 67.7, 66.3, 51.4, 44.5, 40.0, 39.9, 31.4, 29.0, 27.8, 22.4, 13.5, 13.5. **HRMS** (ESI+): calcd for $C_{47}H_{56}O_9N_4Na$ [M+Na]$^+$: 843.3945, found: 843.3932.

3-(Diethylamino)-4-((2-(3-((2-(2-((2-(di(*n*-octyl)amino)-3,4-dioxocyclobut-1-en-1-yl)amino)ethoxy)-4-((2-(2-((2-(di(*n*-octyl)amino)-3,4-dioxocyclobut-1-en-1-yl)amino)ethoxy)phenyl)ethynyl)phenyl)ethynyl)phenoxy)-ethyl)(methyl)amino)cyclobut-3-ene-1,2-dione (RD2)

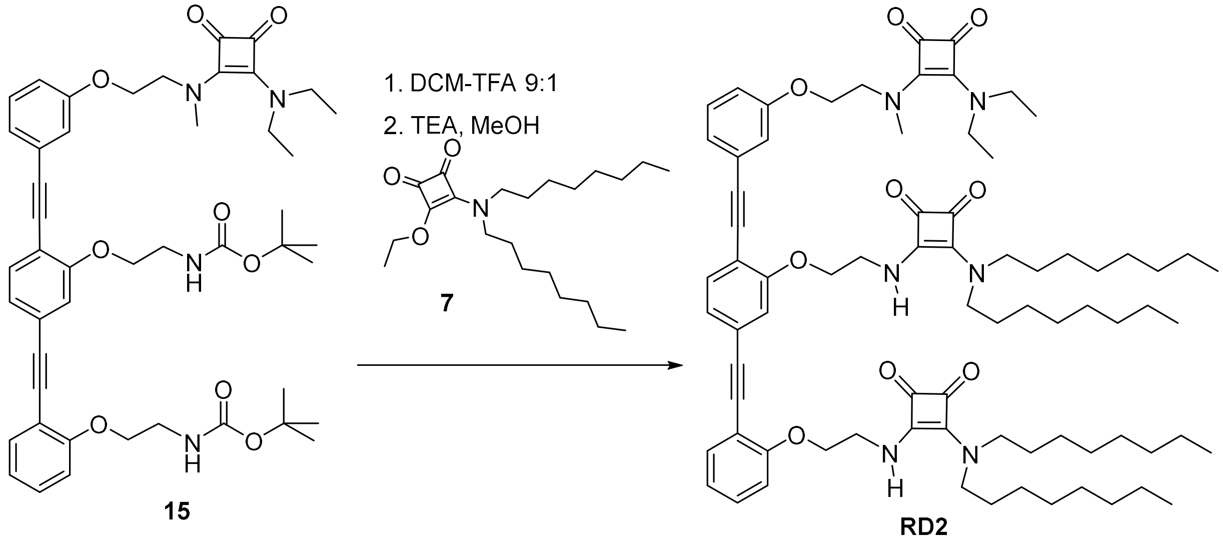

Compound **15** (60 mg, 0.07 mmol) was dissolved in a 9:1 mixture of DCM and TFA (5 mL) and the solution was stirred at room temperature for 1.5 h. The solvents were removed under reduced pressure. The crude residue was redissolved in MeOH (0.5 mL), then TEA (65 µL, 0.48 mmol) and a solution of **7** (56 mg, 0.15 mmol) in MeOH (0.5 mL) were added and the mixture was stirred for 72 h. The solvent was removed under reduced pressure to afford a mixture where the main components were monosubstituted intermediates. The crude was redissolved in MeOH (2 mL), further compound **7** (0.08 mg, 0.15 mmol) and TEA (12 µL, 0.09 mmol) were added and the mixture was stirred again at 60°C for 16 h. The solvent was removed under reduced pressure and the crude was purified by column chromatography (SiO_2, AcOEt-DCM-MeOH 70:28:2) to afford the product as a yellow solid (35 mg, 38%). ***R**$_f$*: 0.2 (AcOEt-MeOH(2%)). **^{1}H NMR** (500 MHz, CD_2Cl_2): δ = 7.49 (d, *J* = 7.6 Hz, 1H), 7.42 (d, *J* = 7.9 Hz, 1H), 7.35 (s, 1H), 7.32 (t, *J* = 7.9 Hz, 1H), 7.26 (t, *J* = 7.9 Hz, 1H), 7.72 (s, 1H), 7.12 (d, *J* = 7.7 Hz, 1H), 7.09 (d, *J* = 7.9 Hz, 1H), 7.02 (d, *J* = 8.3 Hz, 1H), 6.97 (t, *J* = 7.5 Hz, 1H), 6.89 (d, *J* = 8.4 Hz, 1H), 6.68 (t, *J* = 6.1 Hz, 1H), 6.57 (t, *J* = 5.9 Hz, 1H), 4.43 (t, *J* = 6.3 Hz, 2H), 4.31 (mult., 4H), 4.26 (mult., 2H), 4.18 (q, *J* = 6.3 Hz, 2H), 4.07 (t, *J* = 5.7 Hz, 2H), 3.56 (q, *J* = 7.2 Hz, 4H), 3.42 (br, 8H), 3.26 (s, 3H), 1.51 (br, 8H), 1.23 (br, 46H), 0.84 (mult., 12H). **^{13}C NMR** (125 MHz, CD_2Cl_2): δ = 184.6, 183.4, 183.4, 183.2, 182.6, 182.3, 169.8, 168.6, 168.2, 168.0, 167.0, 166.8, 159.1, 158.3, 133.6, 132.9, 130.2, 129.6, 124.9, 124.5, 124.3, 124.2, 121.1, 116.9, 115.9, 115.4, 112.9, 112.6, 112.5, 94.9, 93.2, 87.8, 85.9, 68.6, 68.4, 66.2, 51.7, 49.5, 49.4, 44.9, 43.5, 43.1, 40.8, 31.8, 31.8, 29.4, 29.4, 29.2, 29.2, 26.4, 26.4, 22.6, 22.6, 13.9, 13.8. **HRMS** (ESI+): calcd for $C_{77}H_{106}O_9N_6Na$ [M+Na]$^+$: 1281.7913, found: 1281.7917.

1-(3,5-Bis(trifluoromethyl)phenyl)-3-(2-(3-ethynylphenoxy)ethyl)thiourea (16)

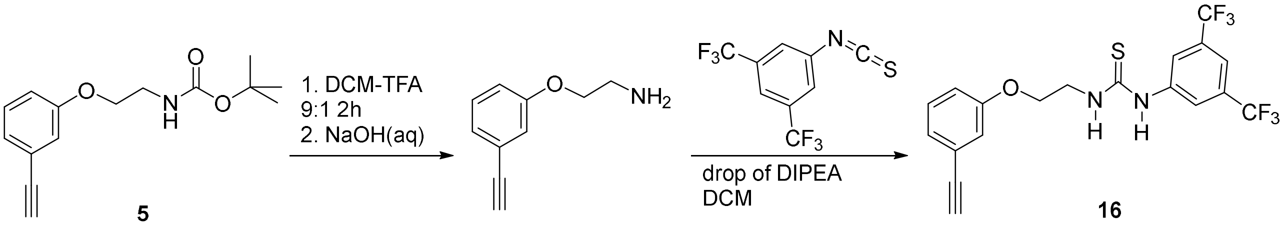

Compound **5** (170 mg, 0.65 mmol) was dissolved in a 9:1 mixture of DCM and TFA (5 mL) and the solution was stirred at room temperature for 2 h. The solvents were removed under reduced pressure, the crude was redissolved in DCM (15 mL) and washed with NaOH (1 M, 10 mL). The aqueous phase was extracted with DCM (2 × 10 mL) and all the organic fractions were combined and dried with $MgSO_4$. The solvent was removed under reduced pressure to afford the deprotected neutral amine. The amine (60 mg, 0.37 mmol) was dissolved in anhydrous DCM (0.7 mL), 3,5-bis(trifluoromethyl)phenyl isothiocyanate (68 µL, 0.37 mmol) and a drop of DIPEA were added, then the mixture stirred for 16 h. The solvent was removed under reduced pressure and the crude was purified by column chromatography (SiO_2, DCM) to afford the product as a white solid (140 mg, 50%). ***R****f*: 0.2 (DCM). **^{1}H NMR** (400 MHz, $CDCl_3$): δ = 7.95 (br, 1H), 7.78 (s, 2H), 7.74 (s, 1H), 7.24 (t, J = 8.1 Hz, 1H), 7.13 (d, J = 7.6 Hz, 1H), 6.99 (s, 1H), 6.86 (d, J = 8.3 Hz, 1H), 6.55 (br, 1H), 4.21 (t, J = 5.0 Hz, 2H), 4.09 (br, 2H), 3.07 (s, 1H). **^{13}C NMR** (100 MHz, $CDCl_3$): δ = 181.0, 157.7, 138.7, 133.3 (q, J = 34.2 Hz), 129.7, 125.6, 124.0, 123.9, 123.5, 122.4 (q, J = 273.3 Hz), 119.8, 117.6, 115.6, 83.0, 66.3, 44.7. **HRMS** (ESI+): calcd for $C_{19}H_{14}ON_2F_6SNa$ $[M+Na]^+$: 455.0623, found: 455.0615.

***tert*-Butyl (2-(2-((4-((3-(2-aminoethoxy)phenyl)ethynyl)-3-(2-((*tert*-butoxycarbonyl)amino)ethoxy)-phenyl)ethynyl)phenoxy)ethyl)carbamate (17)**

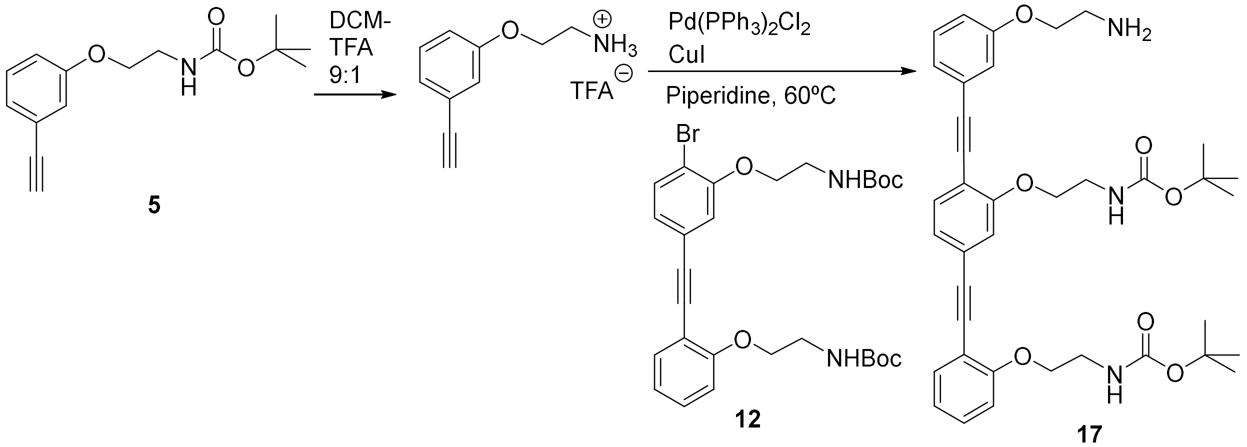

Compound **5** (200 mg, 0.77 mmol) was dissolved in a 9:1 mixture of DCM and TFA (5 mL) and the solution was stirred at room temperature for 2 h. The solvents were removed under reduced pressure. The crude was dissolved in anhydrous piperidine (5 mL) and transferred to a round bottom flask containing compound **12** (220 mg, 0.38 mmol), bis(triphenylphosphine)palladium(II) dichloride (54 mg, 0.08 mmol) and copper iodide (11 mg, 0.06 mmol), and the mixture was stirred for 20 h at 60°C. The solvent was removed under reduced pressure and the crude was purified by column chromatography (SiO_2, DCM-MeOH(8%)) to afford the product as a brown solid (115 mg, 46%). ***R****f*: 0.6 (DCM-MeOH(10%)). **^{1}H NMR** (400 MHz, CD_3CN): δ = 7.48 (mult., 2H), 7.37 (t, J = 7.9 Hz, 1H), 7.33 (t, J = 7.6 Hz, 1H), 7.18 (mult., 4H), 7.03 (mult., 3H), 5.51 (br, 2H), 4.17 (mult., 6H), 3.48 (br, 4H), 1.37 (s, 18H). **^{13}C NMR** (126 MHz, CD_3CN): δ = 159.2, 158.9, 155.8, 133.3, 133.1, 130.2, 129.6, 124.8, 124.4, 124.4, 124.3, 121.1, 117.3, 115.4, 115.3, 113.1, 112.6, 112.6, 94.9, 93.1, 87.5, 85.3, 79.1, 68.3, 68.2, 40.1, 30.6, 28.1. **HRMS** (ESI+): calcd for $C_{38}H_{47}O_7N_3$ $[M+H]^+$: 656.3330, found: 656.3324.

***tert*-Butyl (2-(2-((4-((3-(2-(3-(3,5-bis(trifluoromethyl)phenyl)thioureido)ethoxy)phenyl)ethynyl)-3-(2-((*tert*-butoxycarbonyl)amino)ethoxy)phenyl)ethynyl)phenoxy)ethyl)carbamate** (**18**)

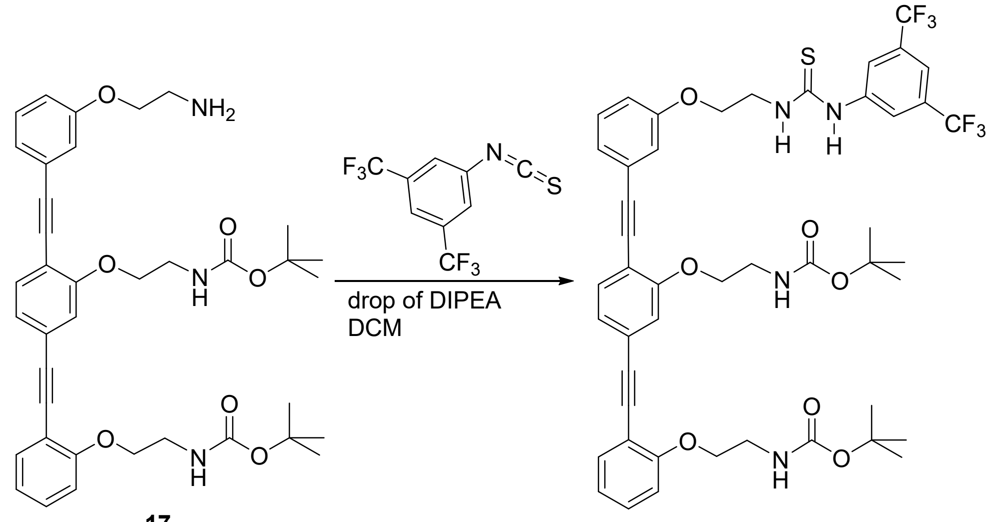

17 **18**

Compound **17** (100 mg, 0.15 mmol) was dissolved in anhydrous DCM (1 mL), 3,5-bis(trifluoromethyl)phenyl isothiocyanate (31 µL, 0.17 mmol) and a drop of TEA were added, then the mixture was stirred for 16 h. The solvent was removed under reduced pressure and the crude product was purified by column chromatography (SiO_2, DCM-AcOEt(8%)) to afford the product as a yellow solid (80 mg, 57%). ***R**$_f$*: 0.6 (DCM-AcOEt(10%)). **^{1}H NMR** (500 MHz, acetone-d_6): δ = 10.28 (s, 1H), 10.20 (s, 1H), 8.21 (s, 2H), 7.75 (s, 1H), 7.46 (s, 1H), 7.45 (d, J = 7.0 Hz, 1H), 7.36 (t, J = 7.5 Hz, 1H), 7.22 (d, J = 7.5 Hz, 1H), 5.70 (s, 1H), 3.95 (mult., 4H). **^{13}C NMR** (126 MHz, CD_2Cl_2): δ = 181.9, 159.6, 158.6, 156.8, 156.2, 140.6, 133.7, 133.0, 132.3, 130.7, 130.2, 125.2, 124.9, 123.7, 123.3, 122.5, 121.5, 118.8, 115.7, 115.5, 113.1, 95.6, 93.4, 88.0, 86.1, 80.2, 79.6, 68.6, 68.2, 67.7, 67.2, 60.6, 44.6, 40.8, 40.5, 28.5, 28.5. **HRMS** (ESI+): calcd for $C_{47}H_{48}O_7N_4F_6NaS$ $[M+Na]^+$: 949.3046, found: 949.3041.

1-(3,5-Bis(trifluoromethyl)phenyl)-3-(2-(3-((2-(2-((2-(di(*n*-octyl)amino)-3,4-dioxocyclobut-1-en-1-yl)amino)-ethoxy)-4-((2-(2-((2-(di(*n*-octyl)amino)-3,4-dioxocyclobut-1-en-1-yl)amino)ethoxy)phenyl)ethynyl)phenyl)-ethynyl)phenoxy)ethyl)thiourea (**RD3**)

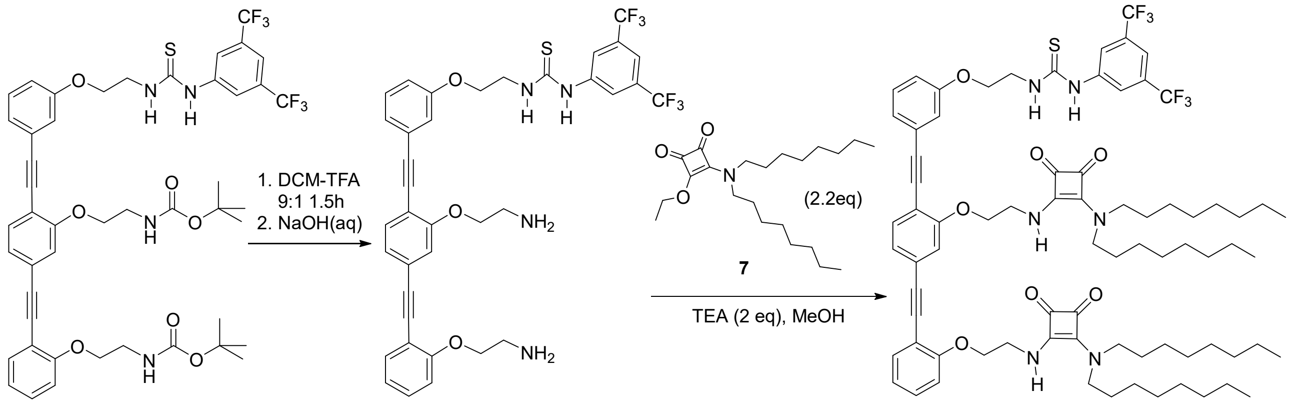

18 **RD3**

Compound **18** (75 mg, 0.08 mmol) was dissolved in a 9:1 mixture of DCM and TFA (5 mL) and the solution was stirred at room temperature for 1.5 h. The solvents were removed under reduced pressure, the crude was redissolved in DCM (10 mL), washed with NaOH (1 M, 5 mL), then dried with $MgSO_4$. After filtration the solvent was removed from the filtrate under reduced pressure to afford the intermediate deprotected diamine. This diamine (45 mg, 0.06 mmol) and compound **7** (54 mg, 0.15 mmol) were dissolved in MeOH (0.5 mL), TEA (21 µL, 0.15 mmol) was added and the mixture was stirred for 72 h. A precipitate appeared after this time, which was separated by filtration, washed with cold MeOH (1 mL) then purified by column chromatography (SiO_2, DCM-AcOEt(15%)) to afford the product as a slightly yellow solid (34 mg, 45%). ***R**$_f$*: 0.5

(DCM-AcOEt(20%)). **^{1}H NMR** (500 MHz, CD_2Cl_2): δ = 10.11 (s, 1H), 8.49 (s, 1H), 8.34 (s, 2H), 7.53 (s, 1H), 7.51 (d, J = 7.6 Hz, 1H), 7.41 (d, J = 8.1 Hz, 1H), 7.35 (t, J = 8.0 Hz, 1H), 7.25 (t, J = 8.0 Hz, 1H), 7.21 (br, 1H), 7.12 (s, 1H), 7.09 (mult., 3H), 7.03 (t, J = 7.6 Hz, 1H), 6.96 (d, J = 8.3 Hz, 1H), 6.90 (d, J = 8.3 Hz, 1H), 5.22 (br, 1H), 4.44 (br, 2H), 4.40 (br, 2H), 4.33 (br, 2H), 4.27 (br, 4H), 4.08 (q, J = 5.2 Hz, 2H), 3.35 (br, 8H), 1.52 (br, overlaped), 1.19 (br, 40H), 0.84 (t, J = 7.2 Hz, 12H). **^{13}C NMR** (101 MHz, CD_2Cl_2): δ = 183.7, 183.1, 181.5, 181.2, 167.4, 167.1, 166.8, 159.4, 158.9, 158.7, 142.1, 133.6, 132.5, 130.8 (q, J_{C-F} = 33 Hz), 130.1, 129.4, 124.3, 124.2, 124.1, 123.6, 123.4 (q, J_{C-F} = 272 Hz), 122.6, 121.6, 119.5, 116.3, 115.8, 114.2, 113.6, 113.2, 112.9, 95.4, 93.4, 87.1, 85.3, 68.3, 67.4, 66.1, 49.9, 44.3, 44.2, 43.8, 31.7, 29.2, 29.1, 26.5, 26.3, 22.5, 13.8. **HRMS** (ESI+): calcd for $C_{77}H_{98}O_7N_6F_6NaS$ $[M+Na]^+$: 1387.7014, found: 1387.7009.

***tert*-Butyl (2-(2-((3-(2-((*tert*-butoxycarbonyl)amino)ethoxy)-4-((3-(2-((2-((3-(dimethylamino)propyl)-(methyl)amino)-3,4-dioxocyclobut-1-en-1-yl)amino)ethoxy)phenyl)ethynyl)phenyl)ethynyl)phenoxy)-ethyl)carbamate (19)**

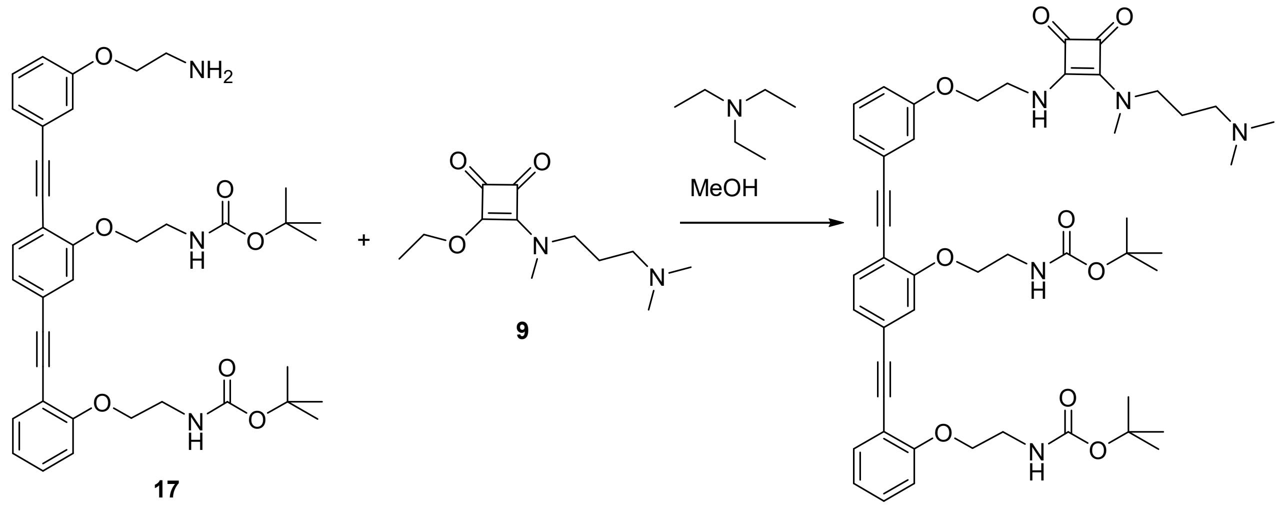

Compounds **17** (50 mg, 0.08 mmol) and **9** (18 mg, 0.08 mmol) were dissolved in MeOH (0.5 mL). TEA (20 µL, 1.92 mmol) was added and the mixture was stirred for 16 h. The solvent was removed under reduced pressure and the crude was purified by column chromatography (SiO_2, DCM-MeOH(10%)) to afford the product as a yellow solid (48 mg, 74%). ***R$_f$***: 0.3 (DCM-MeOH(10%)-TEA(0.5%)). **^{1}H NMR** (400 MHz, CD_3CN): δ = 8.79 (br, 1H), 7.49 (d, J = 7.6 Hz, 1H), 7.46 (d, J = 8.0 Hz, 1H), 7.36 (t, J = 8.0 Hz, 1H), 7.31 (t, J = 8.0 Hz, 1H), 7.17 (mult., 4H), 7.00 (mult., 3H), 5.33 (br, 2H), 4.18 (mult., 4H), 4.11 (t, J = 5.5 Hz, 2H), 4.02 (br, 2H), 3.48 (q, J = 5.5 Hz, 4H), 3.32 (br, 2H), 3.22 (s, 3H), 2.38 (br, 2H), 2.25 (s, 6H), 1.75 (br, 2H), 1.36 (s, 18H). **^{13}C NMR** (101 MHz, CD_3CN): δ = 188.4, 183.8, 170.1, 169.1, 160.3, 160.0, 159.4, 156.9, 134.2, 134.2, 131.5, 130.8, 125.8, 125.3, 125.2, 125.2, 122.0, 118.1, 116.6, 116.3, 113.8, 113.8, 113.2, 95.6, 93.8, 88.7, 86.4, 79.4, 69.2, 68.9, 68.7, 54.9, 49.3, 44.9, 44.5, 40.7, 40.7, 36.2, 28.6, 24.3. **HRMS** (ESI+): calcd for $C_{48}H_{59}O_9N_5Na$ $[M+Na]^+$: 872.4205, found: 872.4196.

3-((3-(Dimethylamino)propyl)(methyl)amino)-4-((2-(3-((2-(2-((2-(di(*n*-octyl)amino)-3,4-dioxocyclobut-1-en-1-yl)amino)ethoxy)-4-((2-(2-((2-(di(*n*-octyl)amino)-3,4-dioxocyclobut-1-en-1-yl)amino)ethoxy)phenyl)ethynyl)-phenyl)ethynyl)phenoxy)ethyl)amino)cyclobut-3-ene-1,2-dione (**RD4**)

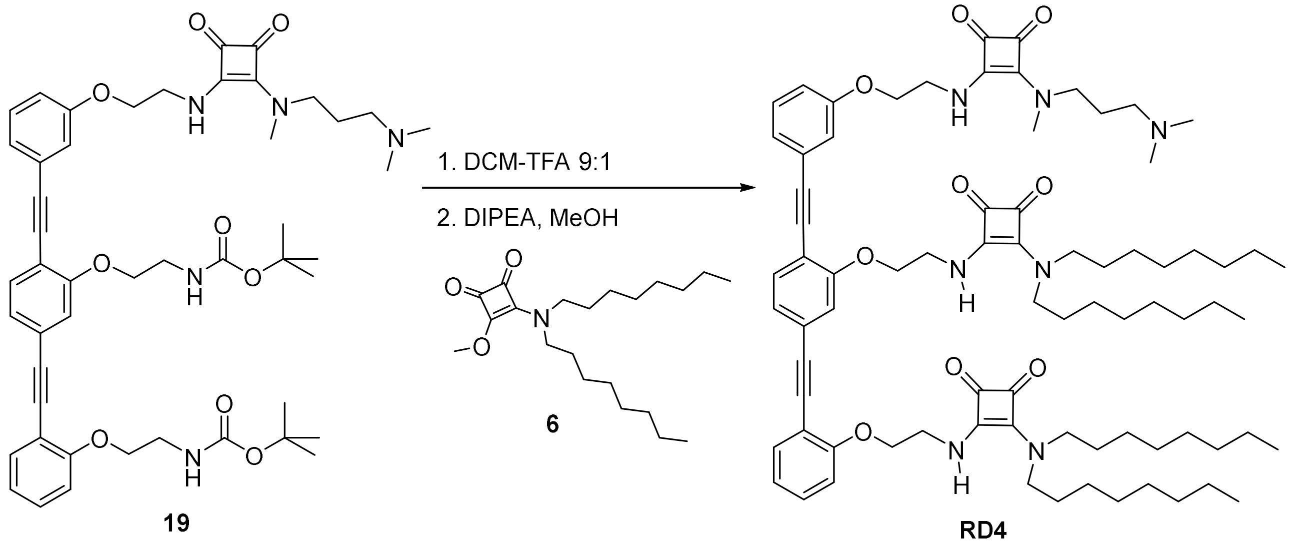

Compound **19** (46 mg, 0.05 mmol) was dissolved in a 9:1 mixture of DCM and TFA (5 mL) and the solution was stirred at room temperature for 1.5 h. The solvents were removed under reduced pressure. The crude was redissolved in MeOH (0.3 mL), then DIPEA (60 µL, 0.33 mmol) and a solution of **6** (46 mg, 0.13 mmol) in MeOH (0.3 mL) were added. The resulting mixture was stirred for 16 h. The solvent was removed under reduced pressure and the crude was purified by column chromatography (SiO_2, DCM-AcOEt-MeOH 85:5:10) to afford a mixture of salts derived from the product and DIPEA. This mixture was further purified by column chromatography (basic Al_2O_3, DCM-MeOH (from 0% to 5% MeOH)) to afford the product as a slightly yellow solid (23 mg, 33%). ***R**$_f$*: 0.4 (DCM-AcOEt(5%)-MeOH(15%)). **^{1}H NMR** (500 MHz, CD_2Cl_2): δ = 9.23 (br, 1H), 7.49 (d, *J* = 7.7 Hz, 1H), 7.42 (d+s, 2H), 7.34 (t+s, 2H), 7.26 (t, *J* = 8.0 Hz, 1H), 7.11 (d+d, 2H), 7.04 (d, *J* = 8.4 Hz, 1H), 6.98 (br, 2H), 6.89 (d, *J* = 8.4 Hz, 1H), 6.59 (br, 1H), 4.44 (t, *J* = 6.5 Hz, 2H), 4.28 (mult., 6H), 4.17 (br, 2H), 3.98 (br, 2H), 3.45 (br, 13H), 2.35 (br, 2H), 2.25 (s, 6H), 1.75 (br, 2H), 1.55 (br, overlapped), 1.49 (br, 4H), 1.22 (br, 40H), 0.85 (mult., 12H). **^{13}C NMR** (126 MHz, CD_2Cl_2): δ = 184.0, 183.8, 183.5, 183.1, 183.0, 182.7, 169.4, 169.0, 168.6, 168.4, 167.4, 167.2, 159.5, 158.9, 134.0, 133.2, 130.6, 130.0, 125.4, 124.9, 124.6, 124.6, 121.4, 116.8, 116.7, 115.6, 113.2, 113.0, 112.9, 95.4, 93.6, 88.2, 86.3, 69.0, 68.6, 68.4, 49.8, 48.8, 46.5, 45.0, 43.9, 43.3, 36.2, 32.2, 29.8, 29.8, 29.6, 29.6, 26.8, 26.8, 23.9, 23.0, 23.0, 20.6, 14.3. **HRMS** (ESI+): calcd for $C_{78}H_{109}O_9N_7Na$ $[M+Na]^+$: 1310.8179, found: 1310.8190.

3. ^{1}H NMR experiments for compounds 16 and RD1-4

The conformational properties of compounds **RD2-4** dissolved in CD_2Cl_2 were studied by ^{1}H NMR spectroscopy. Compounds **RD1** and **16** were also studied as reference compounds.

The spectra recorded in pure CD_2Cl_2 were referenced to the signal of $CDHCl_2$ at 5.32 ppm.

The spectra recorded in the presence of DMSO-d_6 were referenced to a TMS signal at 0 ppm, because the signal of $CDHCl_2$ was significantly affected during the DMSO-d_6 titrations (see Section 3.3).

3.1. Assignment of the ^{1}H NMR spectra in CD_2Cl_2

The ^{1}H NMR spectra of compounds **RD1** and **16** were assigned based on the multiplicity and chemical shifts of the signals observed (see Figures S1 and S6 respectively).

The ^{1}H NMR spectra of compounds **RD2-4** were assigned based on the multiplicity and chemical shifts of the signals observed, as well as the COSY and NOESY spectra (see Figure S1 to Figure S4).

- Each aromatic ring from the *oligo*-phenylene ethynylene rigid-rods showed a characteristic pattern around the diagonal in the aromatic region of the COSY spectra, which permitted the assignment of the corresponding signals.
- The signals from each CH_2CH_2NH system were connected using COSY cross-peaks.
- Each CH_2CH_2NH system was connected to the appropriate aromatic ring of the *oligo*-phenylene ethynylene rigid-rods based on NOESY cross-peaks, which permitted the unequivocal assignment of all the CH_2CH_2NH signals.
- The signals of the C_2H_5, C_8H_{17} and $C_3H_6NH(CH_3)_2$ substituents on the squaramides all showed characteristic multiplicities and chemical shifts, with expected COSY cross-peaks.
- The signals of the CH_3 squaramide substituents and the 3,5-$C_6H_3(CF_3)_2$ thiourea substituents appeared as singlets and showed characteristic chemical shifts.

The Supplemental Information spectra were assigned using numbers for the NH signals (1, 2, 3 or 4; corresponding to NH1, NH2, NH3 or NH4 in the manuscript), capital letters with a subscript for the aromatic signals from the *oligo*-phenylene ethynylene rigid-rods (A-C_{1-4}) and lowercase letters for the rest of CH signals (a-n).

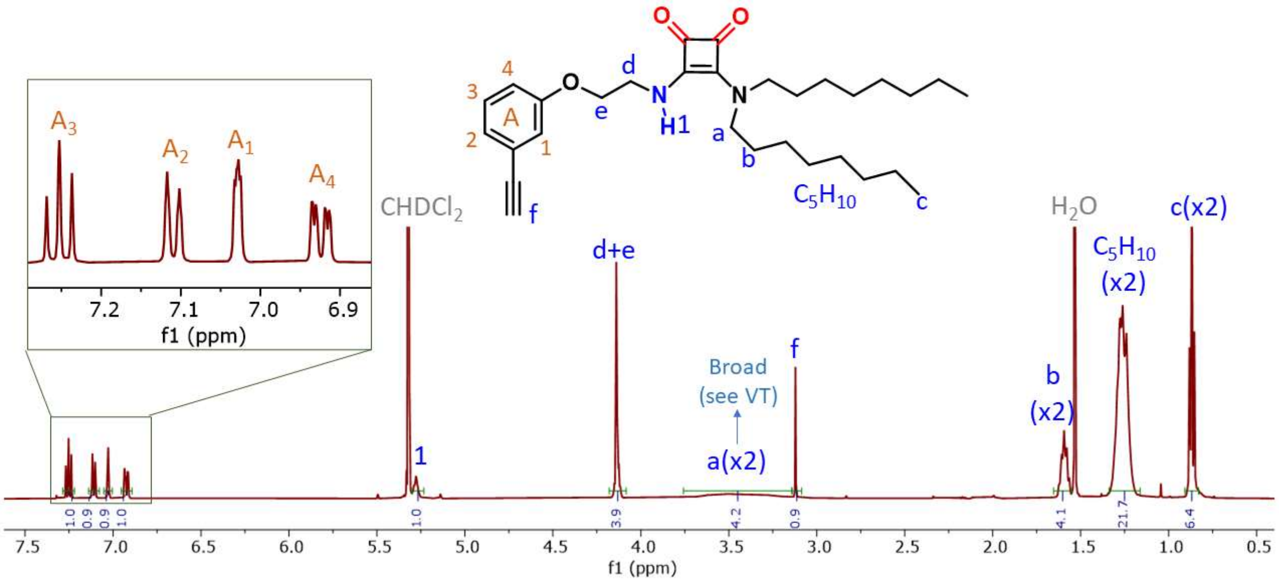

Figure S1. Assigned ^1H NMR spectrum (500 MHz, 298 K, CD_2Cl_2) of compound **RD1** (2.5 mM).

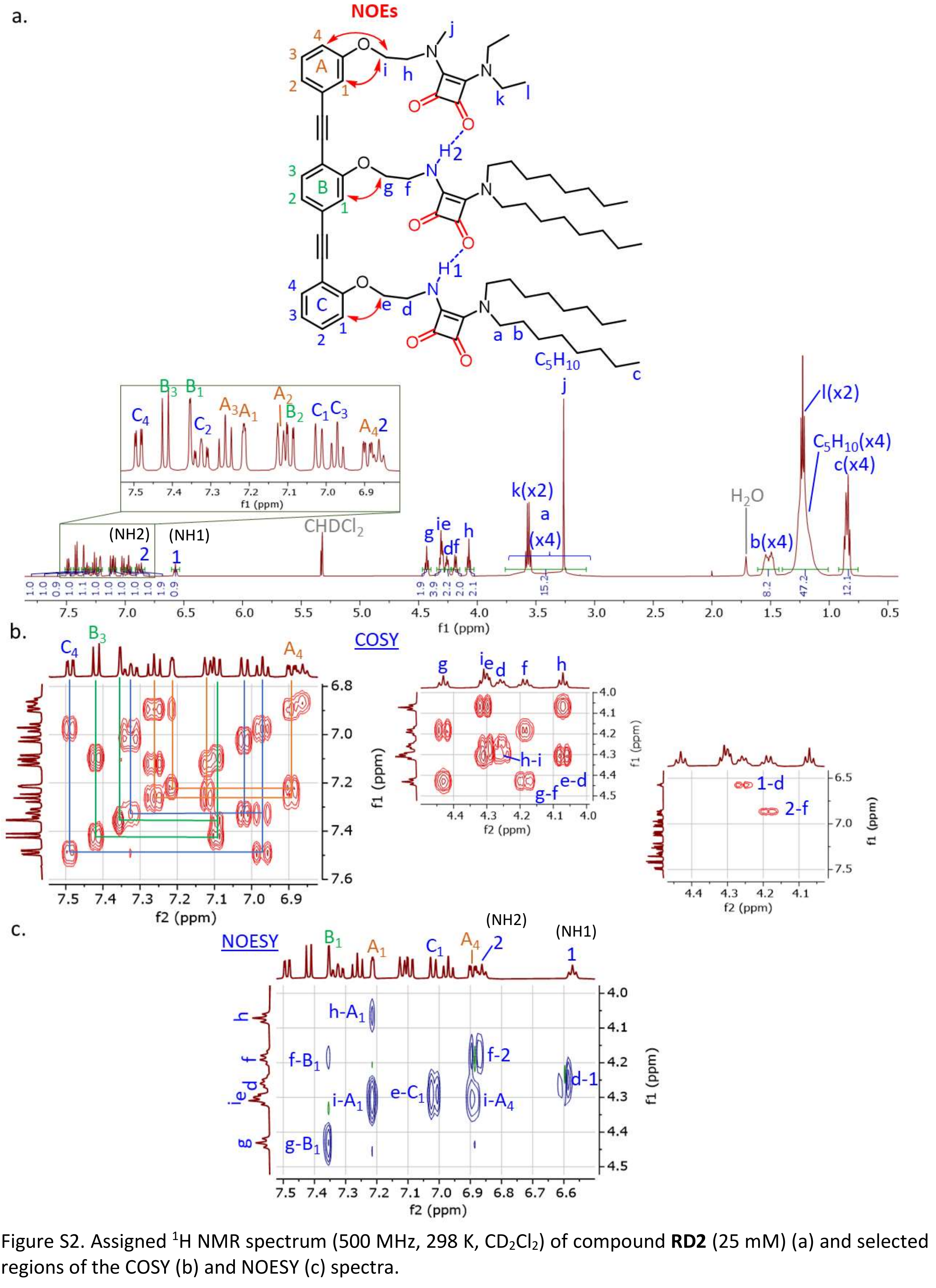

Figure S2. Assigned ^{1}H NMR spectrum (500 MHz, 298 K, CD_2Cl_2) of compound **RD2** (25 mM) (a) and selected regions of the COSY (b) and NOESY (c) spectra.

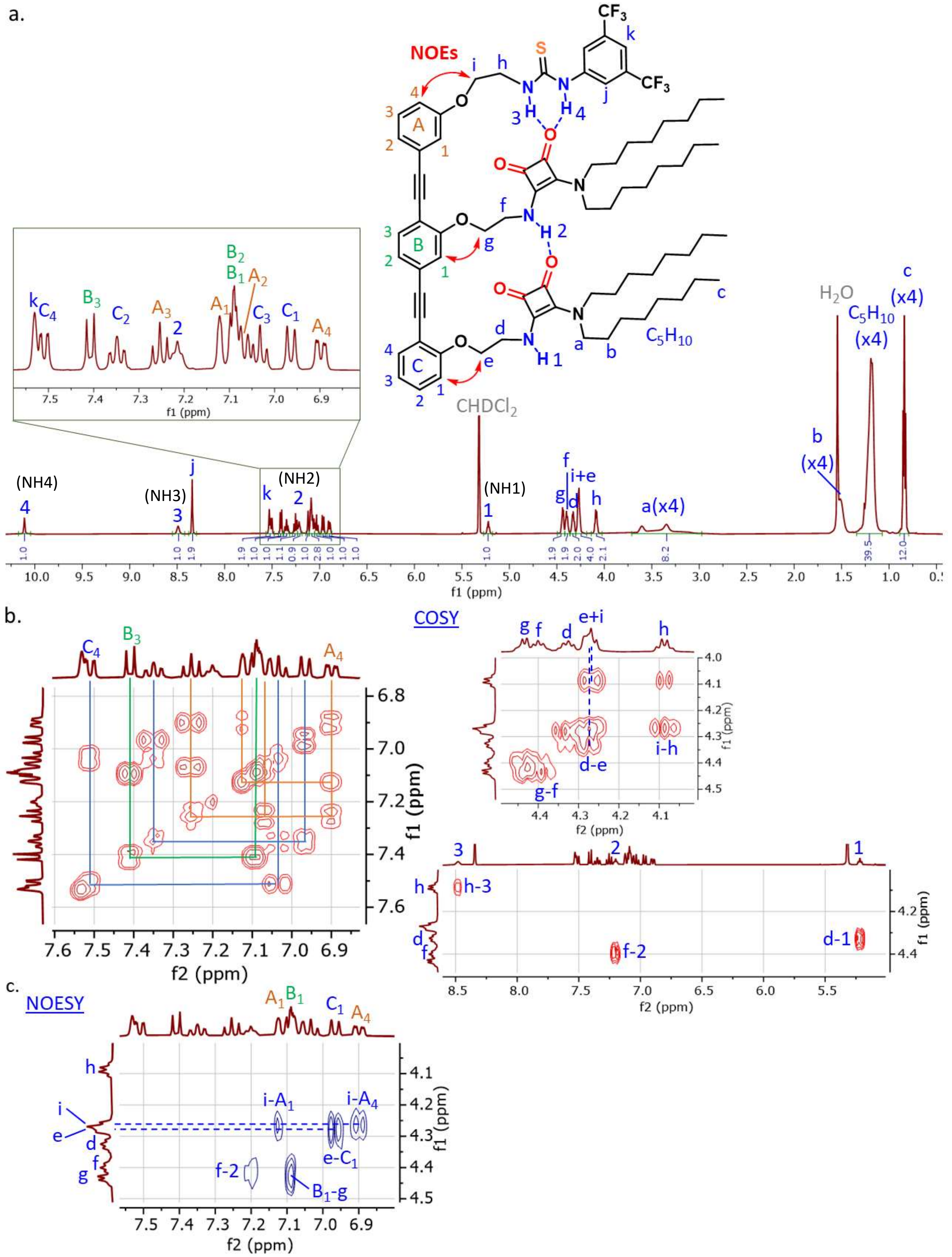

Figure S3. Assigned ^1H NMR spectrum (500 MHz, 298 K, CD_2Cl_2) of compound **RD3** (2.5 mM) (a) and selected regions of the COSY (b) and NOESY (c) spectra.

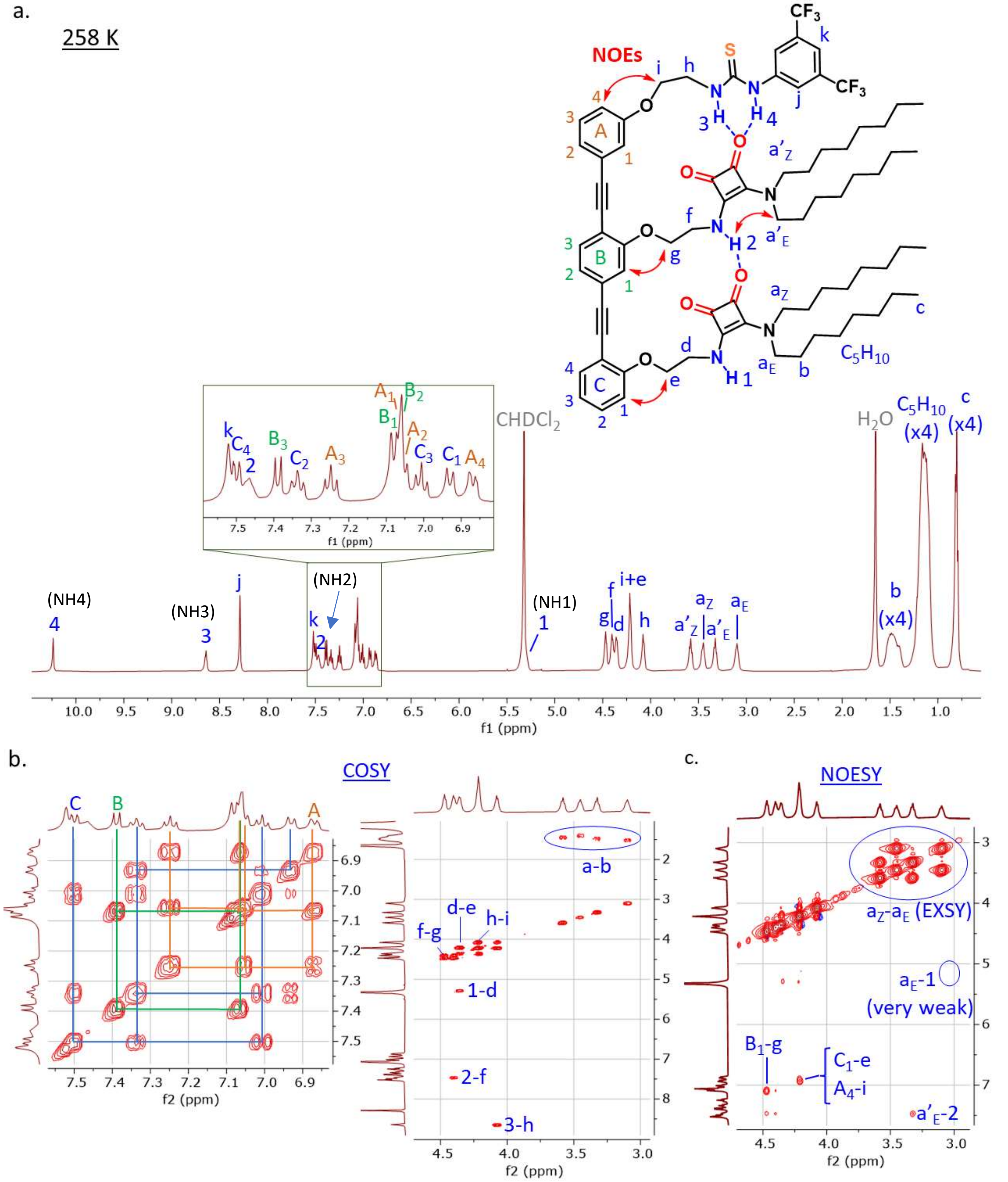

Figure S4. Assigned ^{1}H NMR spectrum (500 MHz, 258 K, CD_2Cl_2) of compound **RD3** (2 mM) (a) and selected regions of the COSY (b) and NOESY (c) spectra.

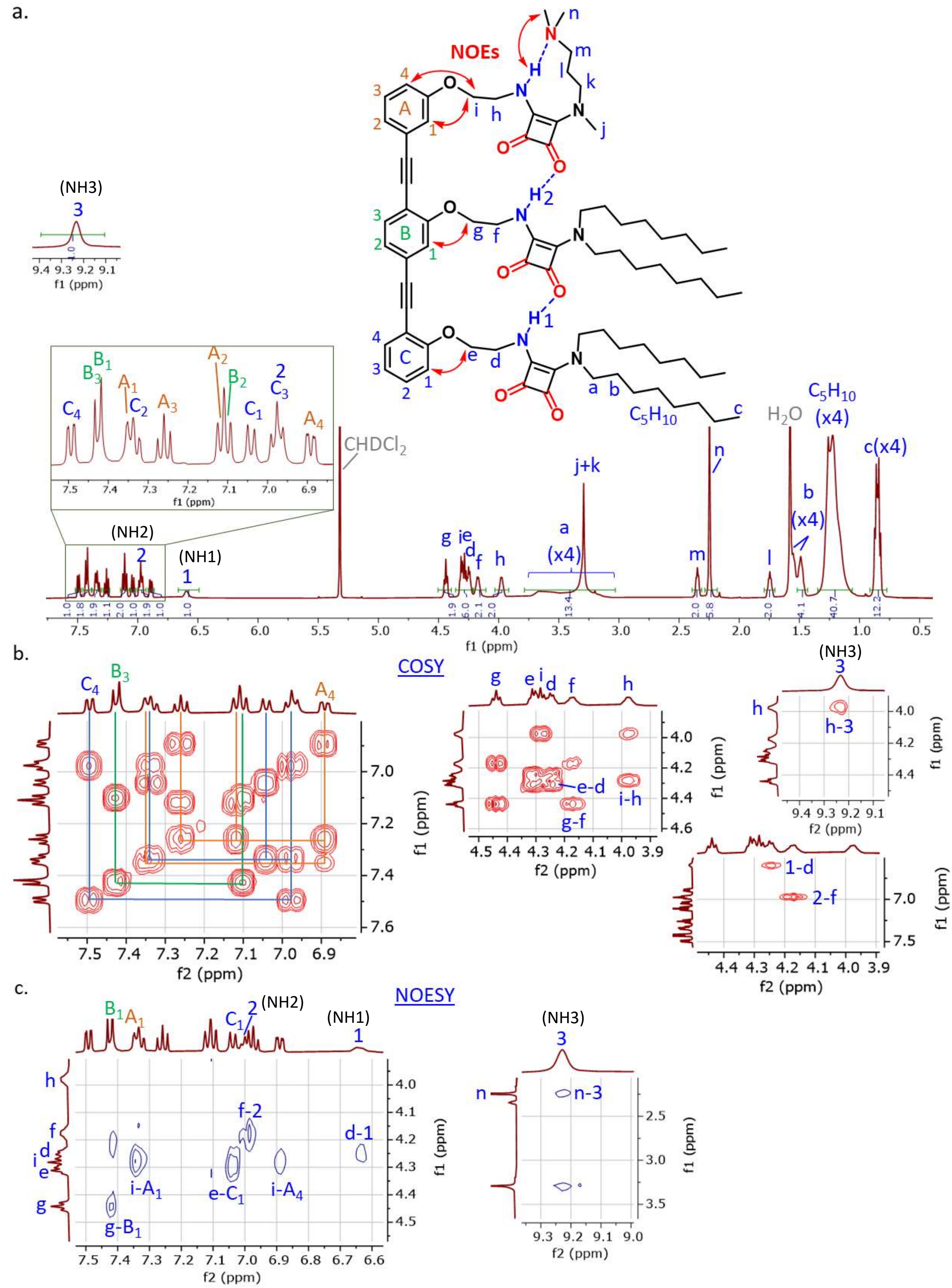

Figure S5. Assigned ^1H NMR spectrum (500 MHz, 298 K, CD_2Cl_2) of compound **RD4** (2.5 mM) (a) and selected regions of the COSY (b) and NOESY (c) spectra.

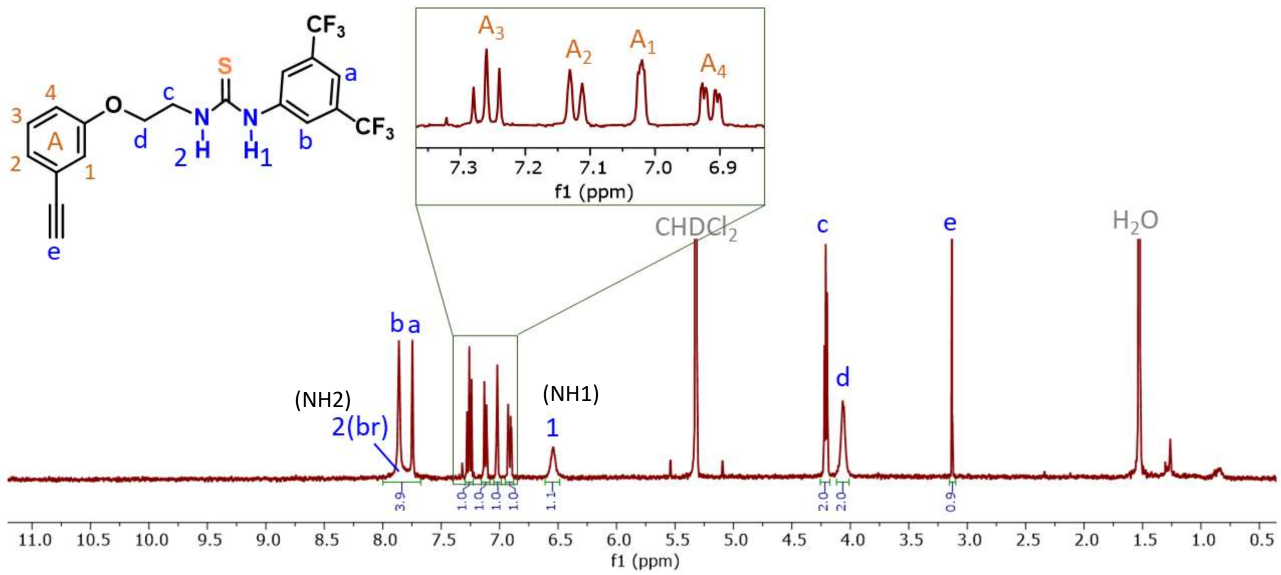

Figure S6. Assigned ^{1}H NMR spectrum (400 MHz, 298 K, CD_2Cl_2) of compound **16** (2.5 mM). The resonance for NH2 is broad (br) and under the resonance for “b”.

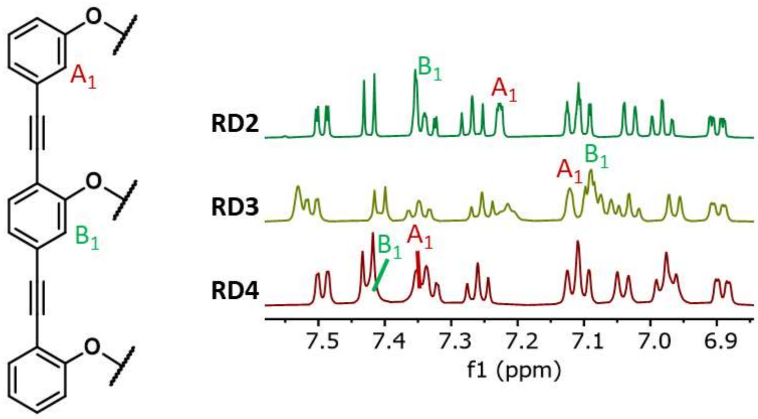

Figure S7. Aromatic regions of the ^{1}H NMR spectra (500 MHz, 298 K, CD_2Cl_2) of compounds **RD2-4** (2.5 mM).

3.2. VT-NMR experiments in CD_2Cl_2

For the VT-NMR experiments (from Figure S8 to Figure S12), solutions of pure compounds (2.5 mM, 600 µL) were transferred into NMR tubes, and the ^{1}H NMR spectra were recorded at different temperatures (298-250 K). In certain cases, the chemical shifts of the NH signals at low temperature were confirmed with COSY experiments.

VT-NMR experiments did not show any broadening or splitting of the NH signals that would indicate the presence of minor conformers in solution (Figures S8-12)

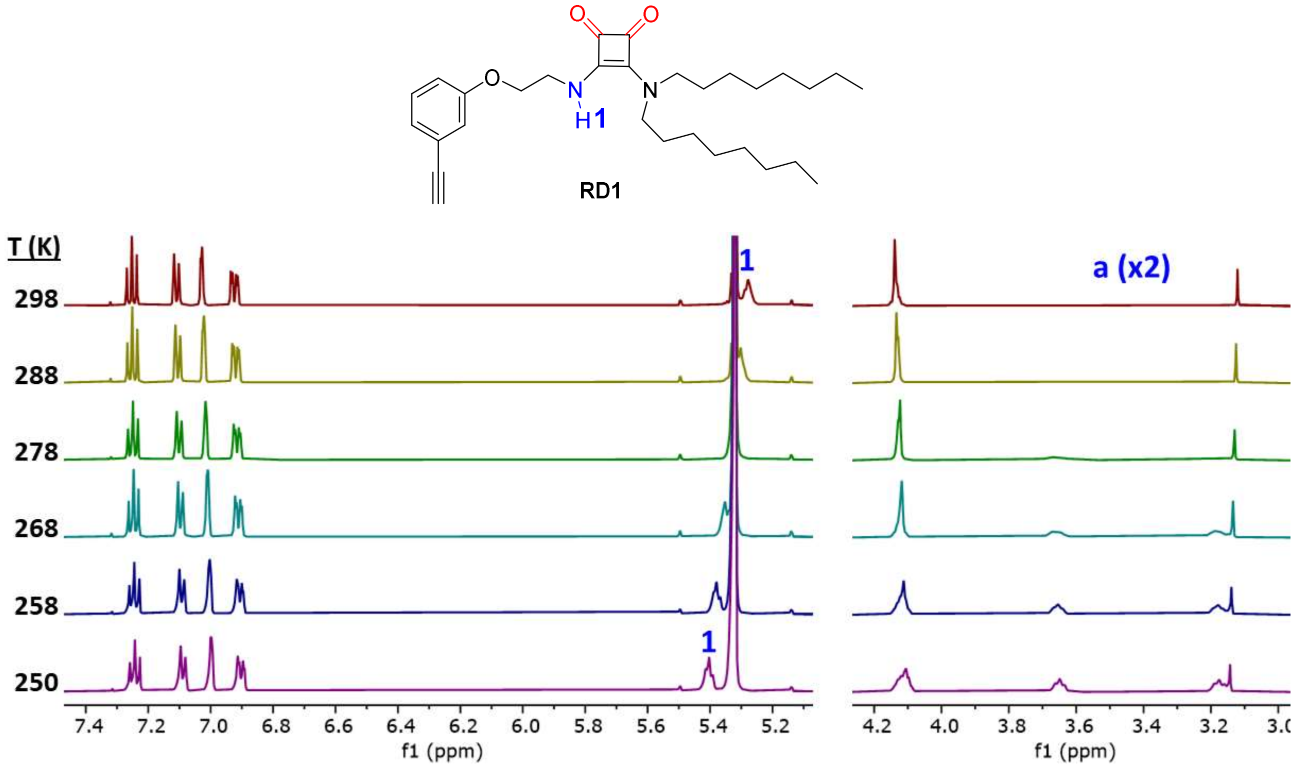

Figure S8. Selected regions of the ^{1}H NMR spectra (500 MHz, CD_2Cl_2) of compound **RD1** (2.5 mM) at different temperatures.

a.

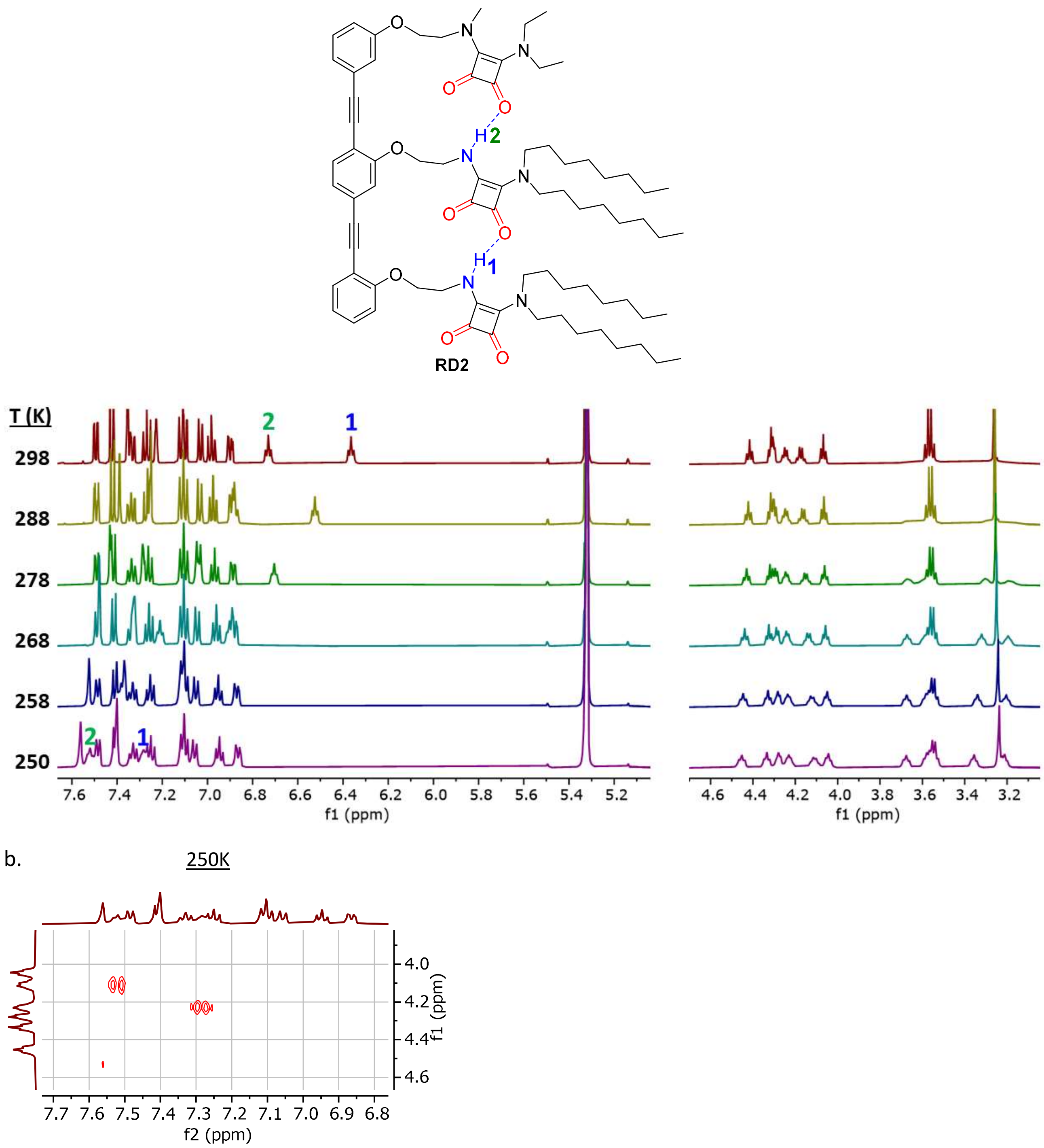

Figure S9. a. Selected regions of the ^{1}H NMR spectra (500 MHz, CD_2Cl_2) of compound **RD2** (2.5 mM) at different temperatures. b. Selected region of the COSY spectrum at 250 K (the CH_2-NH cross-peaks shown reveal the chemical shifts of the NH signals).

a.

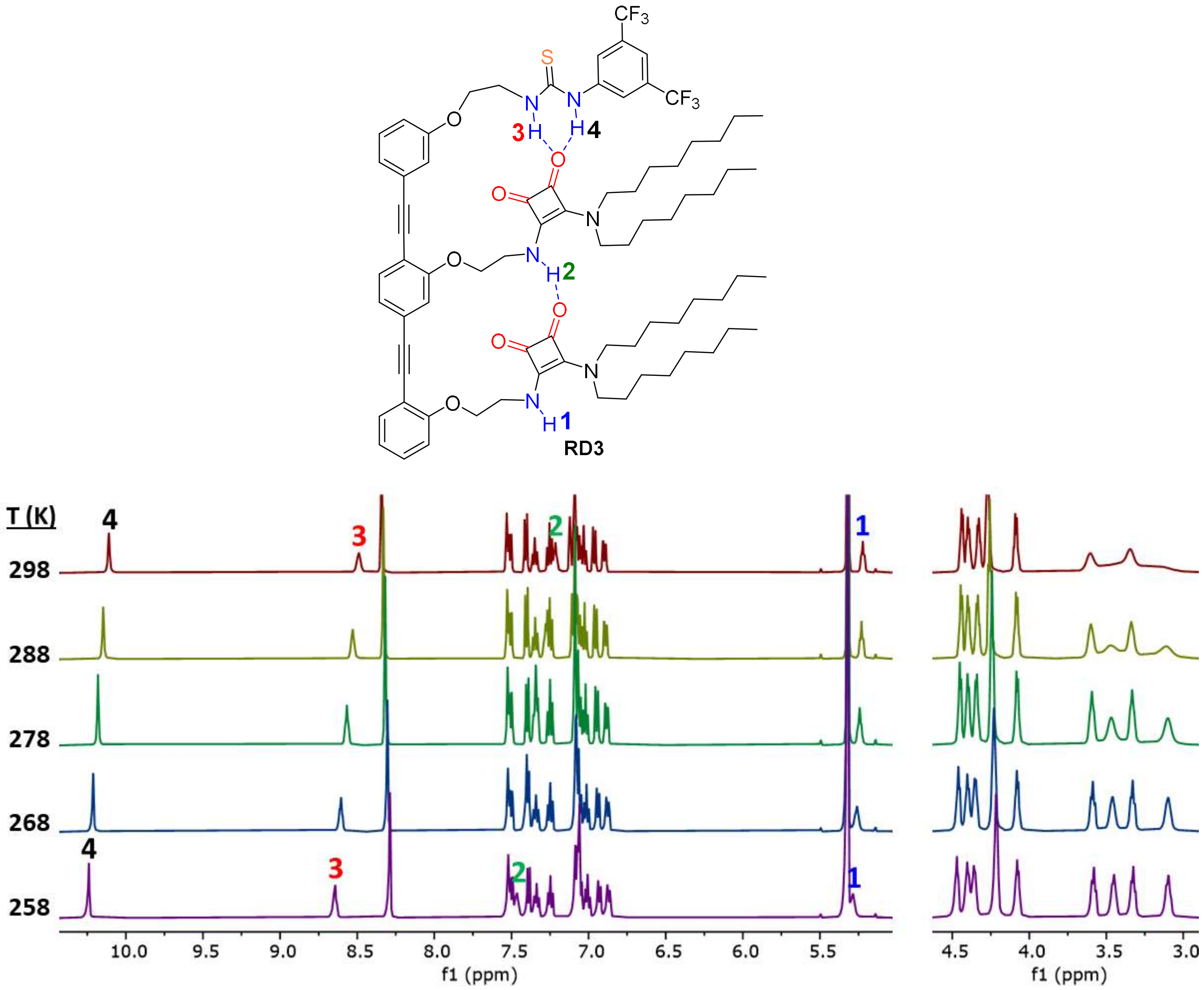

b.

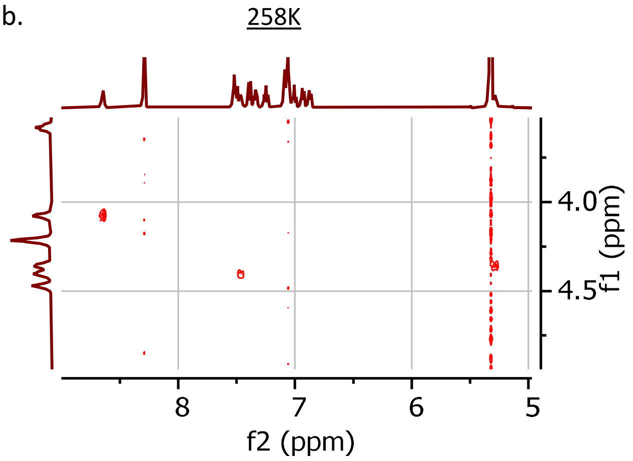

Figure S10. a. Selected regions of the ^{1}H NMR spectra (500 MHz, CD_2Cl_2) of compound **RD3** (2.5 mM) at different temperatures. b. Selected region of the COSY spectrum at 258 K (the CH_2-NH cross-peaks shown reveal the chemical shifts of the NH signals).

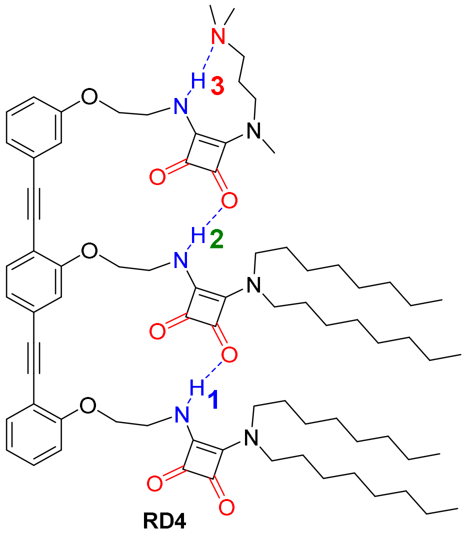

RD4

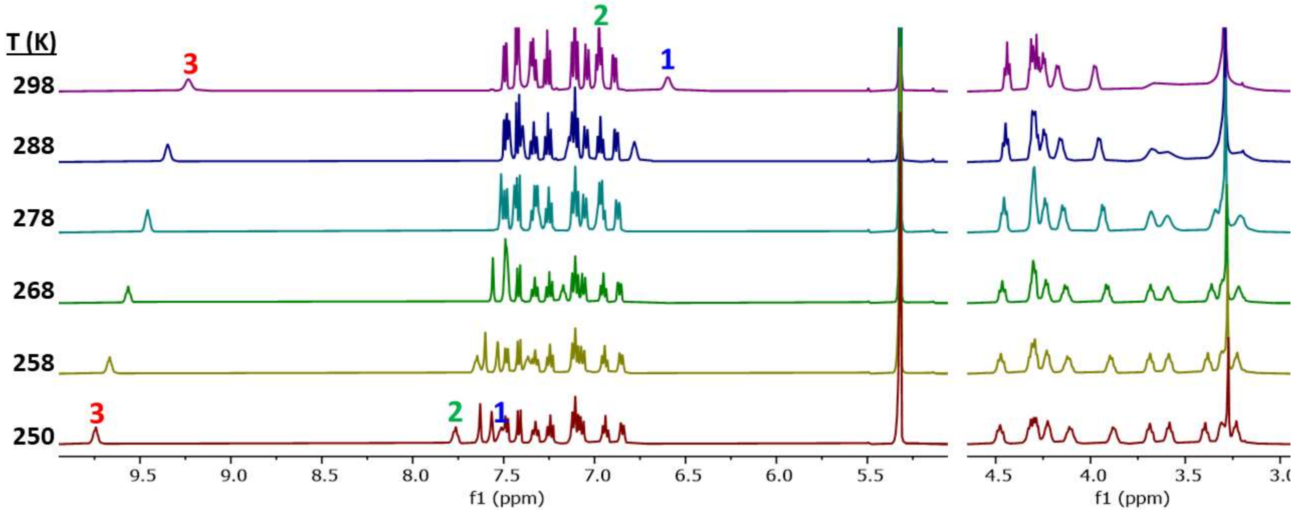

Figure S11. Selected regions of the ^{1}H NMR spectra (500 MHz, CD_2Cl_2) of compound **RD4** (2.5 mM) at different temperatures.

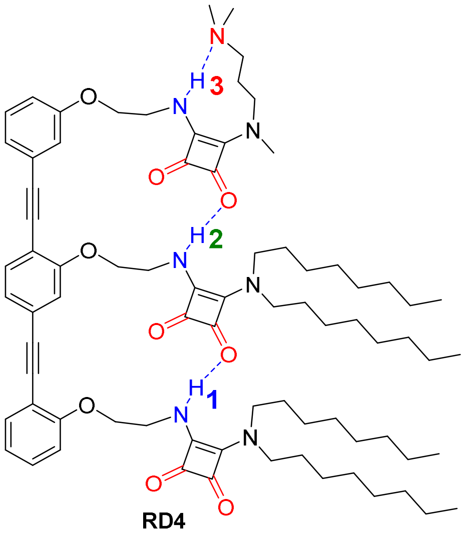

RD4

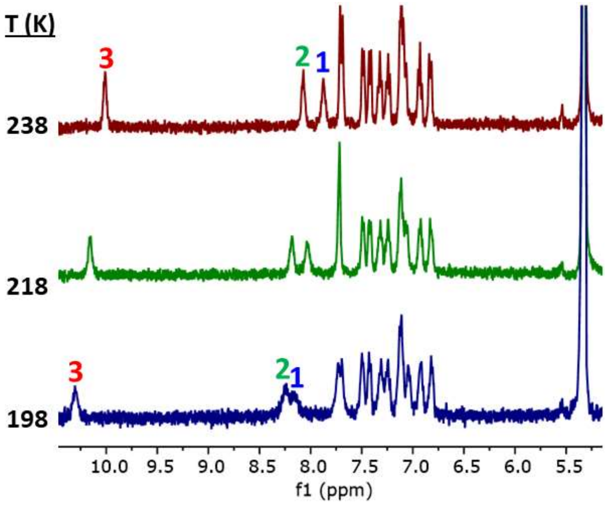

Figure S12. Selected regions of the ^{1}H NMR spectra (500 MHz, CD_2Cl_2) of compounds **RD4** (2 mM) at different temperatures below 250 K.

3.3. Titrations with DMSO-d_6 in CD_2Cl_2

The effect of DMSO-d_6 on the different NH signals of **RD1-4** was studied by adding increasing amounts of DMSO-d_6 into solutions of the different compounds in CD_2Cl_2 at 298 K. Solutions of compounds **RD1-4** (each 2.5 mM, 0.6 mL) were prepared in CD_2Cl_2 and the ^1H NMR spectra were recorded. Then aliquots of DMSO-d_6 were added to each NMR tube and ^1H NMR spectra were recorded after each addition (from Figure S13 to Figure S17).

Sequential additions of DMSO-d_6 : 5, 5, 10, 10, 10, 20, 30, 30, 60 and 60 µL.

In some cases, the chemical shifts of the NH signals in presence of different amounts of DMSO-d_6 were confirmed with COSY experiments.

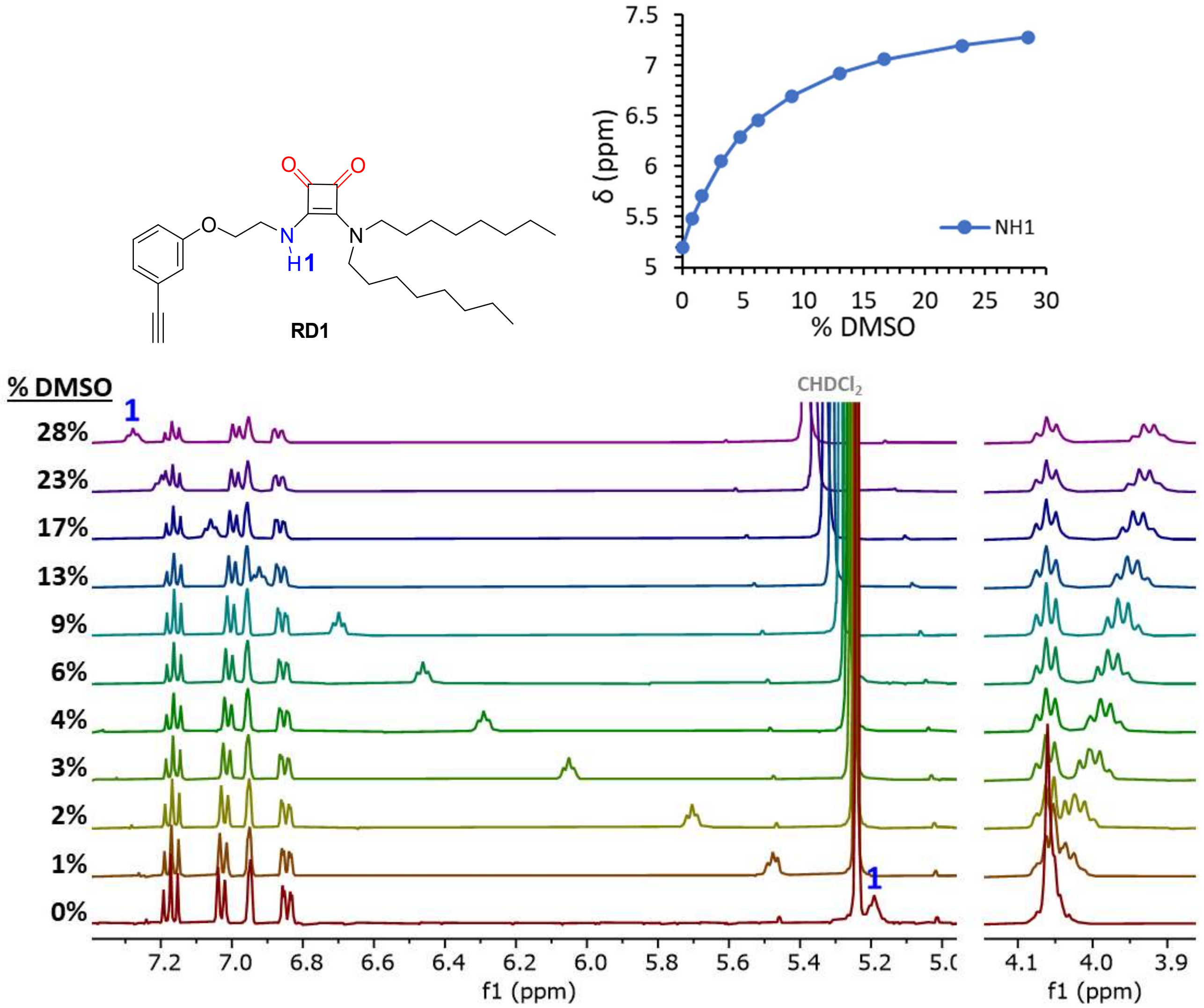

Figure S13. Selected regions of ^1H NMR spectra (400 MHz, 298K, CD_2Cl_2) from the titration of compound **RD1** (initially 2.5 mM) with DMSO-d_6. The proportion of DMSO-d_6 is indicated as % v/v.

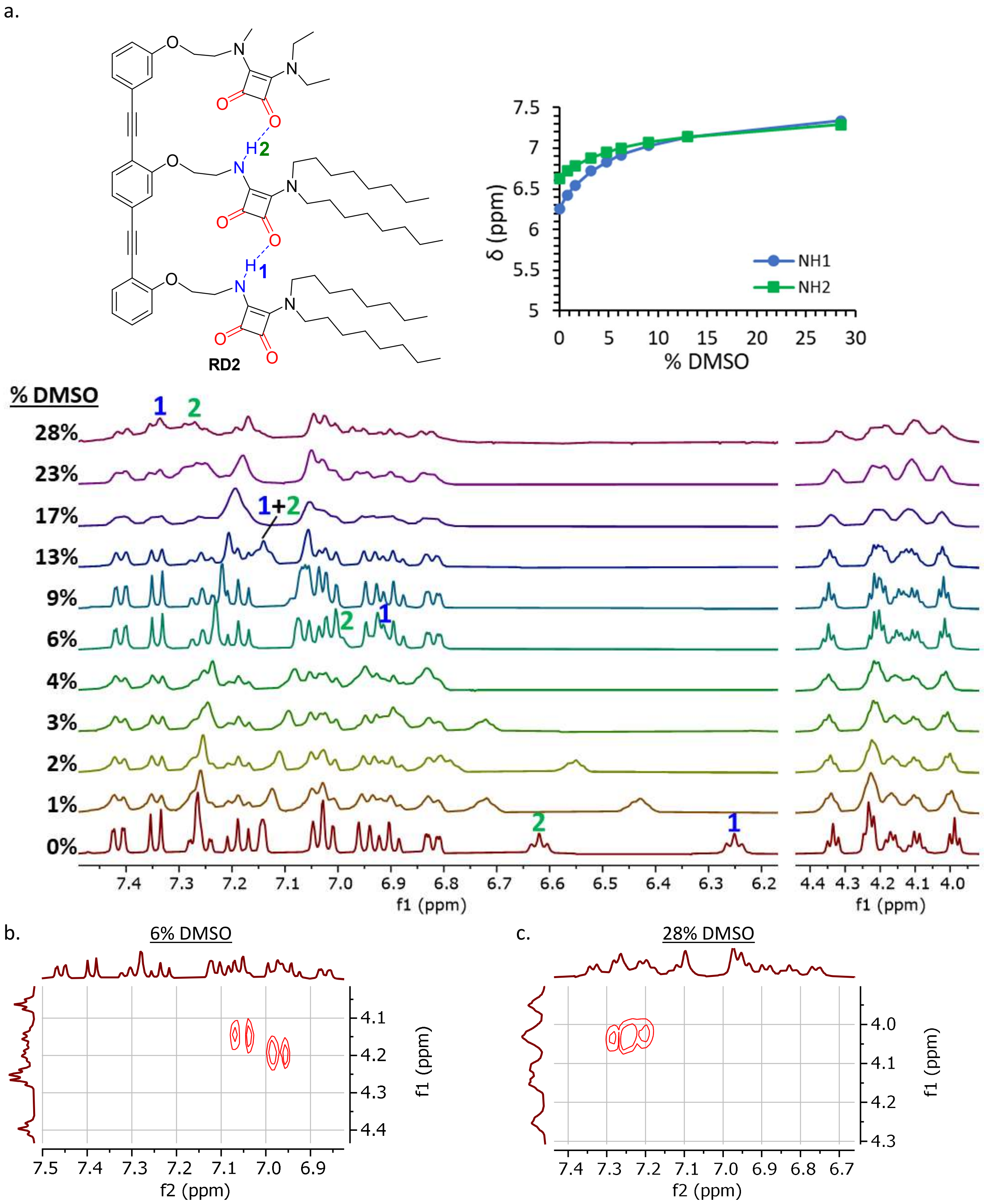

Figure S14. a. Selected regions of ^{1}H NMR spectra (400 MHz, 298K, CD_2Cl_2) from the titration of compound **RD2** (initially 2.5 mM) with DMSO-d_6. The proportion of DMSO-d_6 is indicated as % v/v. b-c. Selected regions of the COSY spectrum in presence of 6% DMSO-d_6 (b) and 28% DMSO-d_6 (c) (the CH_2-NH cross-peaks shown reveal the chemical shifts of the NH signals).

a.

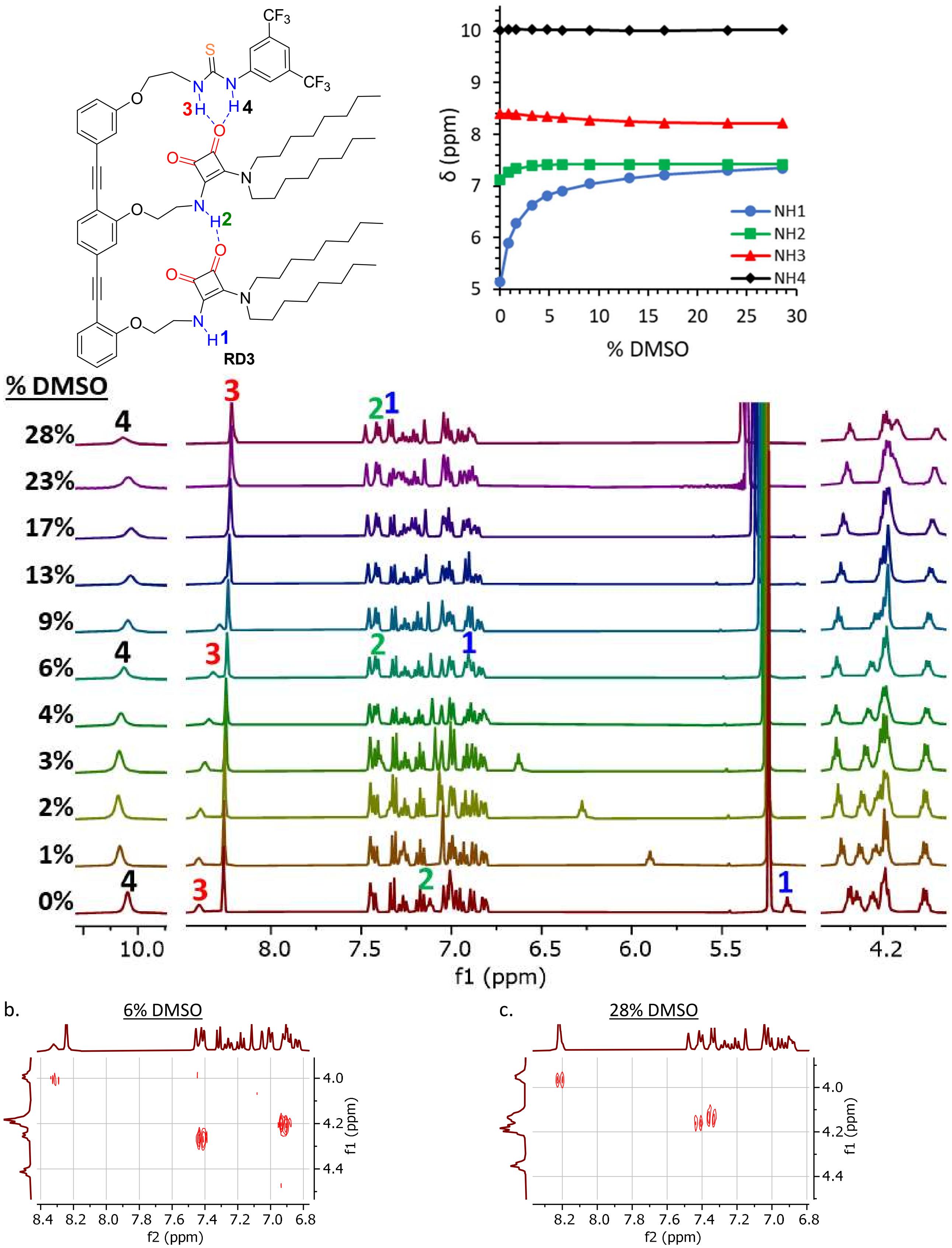

Figure S15. a. Selected regions of ^1H NMR spectra (400 MHz, 298K, CD_2Cl_2) from the titration of compound **RD3** (initially 2.5 mM) with DMSO-d_6. The proportion of DMSO-d_6 is indicated as % v/v. b-c. Selected regions of the COSY spectrum in presence of 6% DMSO-d_6 (b) and 28% DMSO-d_6 (c) (the CH_2-NH cross-peaks shown reveal the chemical shifts of the NH signals).

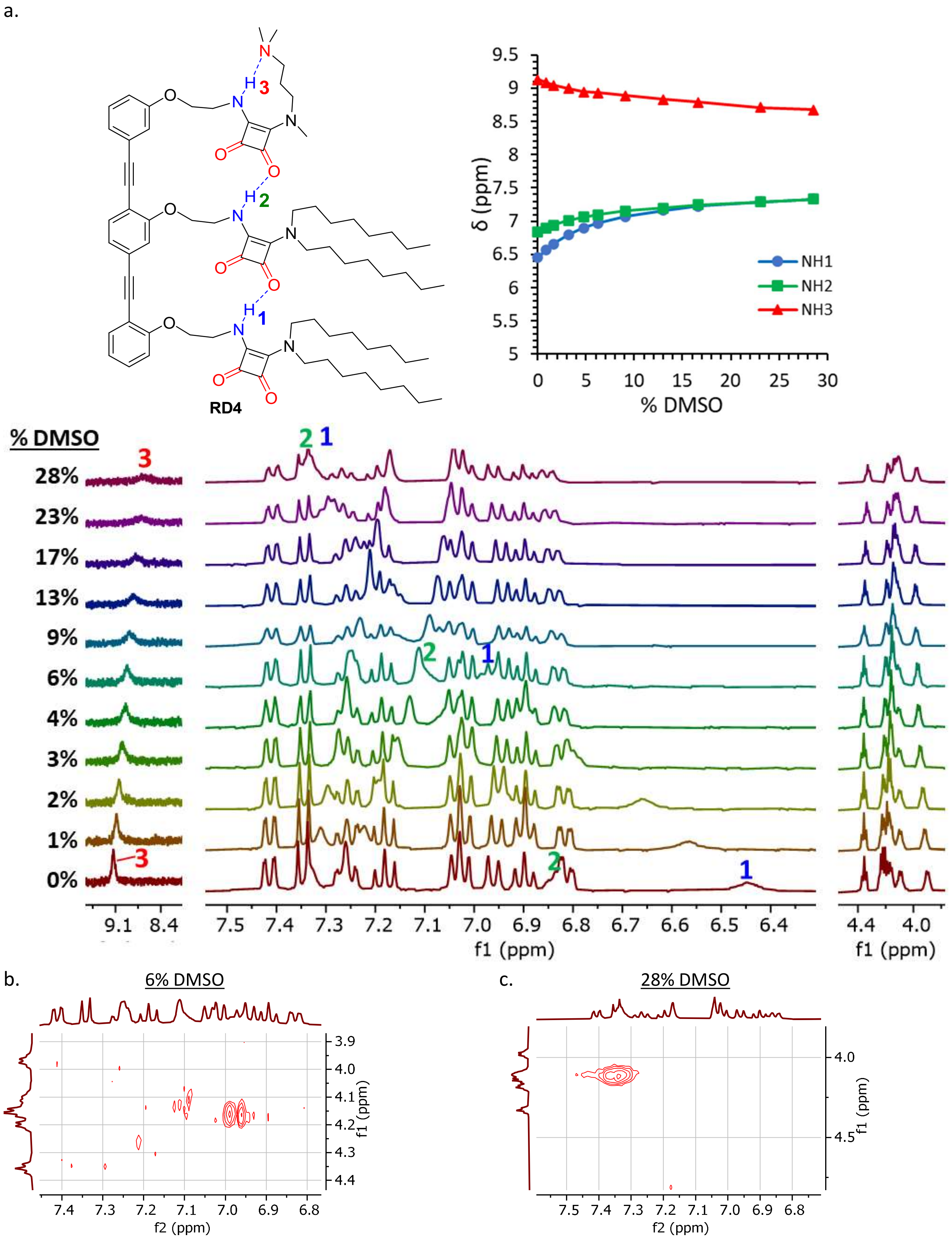

Figure S16. Selected regions of ^{1}H NMR spectra (400 MHz, 298K, CD_2Cl_2) from the titration of compound **RD4** (initially 2 mM) with DMSO-d_6. The proportion of DMSO-d_6 is indicated as % v/v. b-c. Selected regions of the COSY spectrum in presence of 6% DMSO-d_6 (b) and 28% DMSO-d_6 (c) (the CH_2-NH cross-peaks shown reveal the chemical shifts of the NH signals).

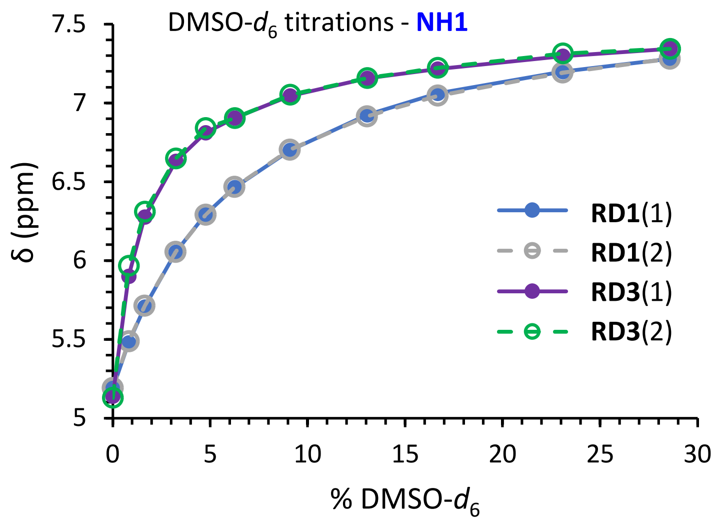

Figure S17. Variation of the chemical shift of the respective NH1 during the titrations of **RD1** and **RD3** (2.5 mM) with DMSO-d_6 at 298K in CD_2Cl_2. Each titration was performed twice.

3.4. Spectra of of RD1-RD4 in acetone-d_6

The ^{1}H NMR spectroscopic properties of **RD1**-**RD4** in acetone-d_6 have also studied. However, this hydrogen bond accepting solvent makes interpretation of the chemical shifts of the different NHs more difficult, specifically because the diagnostic signal, the resonance of NH1, is now much more downfield compared to its position in dichloromethane-d_2. The differences between the position of the NH1 resonance in **RD2-4** with the NH of **RD1** are now smaller. Although the difference between NH2 and NH1 is largest for **RD3**, consistent with a parallel orientation for this array in acetone-d_6, we cannot draw unequivocal conclusions about relay orientation for these compounds in acetone-d_6.

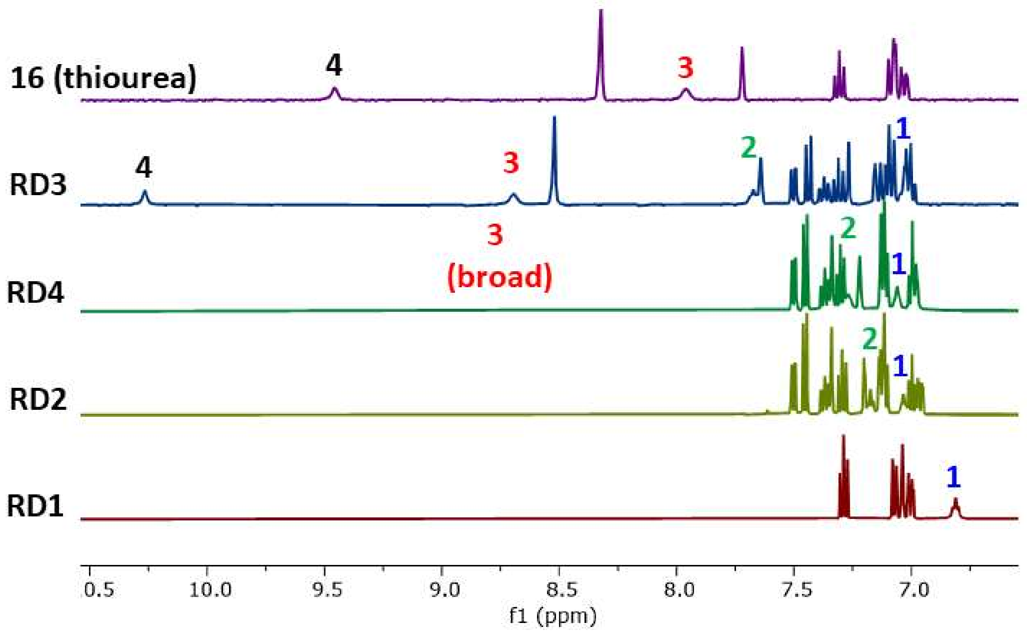

Figure S18. Key NH resonances of **RD1**-**RD4** and thiourea **16** at 298 K in acetone-d_6.

4. X-ray crystal structures

4.1. Crystallographic data

Data collection: X-ray diffraction data were collected for compounds **RD3** and **RD4** on a dual source Rigaku FR-X rotating anode at 100 K with Cu-Kα (1.54184 Å) radiation, equipped with a Hypix000HE detector and Oxford cryosystem. X-ray data were collected using CrysAlisPro software.

Crystal structure determination and refinements: X-ray data were processed and reduced using CrysAlisPro. Absorption correction was performed using empirical methods (SCALE3 ABSPACK) based upon symmetry-equivalent reflections combined with measurements at different azimuthal angles. The crystal structure was solved and refined against all F^2 values using the SHELX and Olex2 suite of programmes.[S6,S7] All atoms were refined anisotropically. Hydrogen atoms were placed in calculated positions and refined using idealised geometries and assigned fixed isotropic displacement parameters. Atomic displacement parameters were restrained using a rigid body approach by applying SHELX RIGU commands and to be similar using SHELX SIMU commands. Disordered moieties in **RD3** were modelled and refined over two positions. Bond distances of pairs of disordered atoms were restrained to be similar using SHELX SADI command. Despite the usage of a highly intense X-ray source, crystals of **RD4** diffracted to 1 Å of resolution, so the data was trimmed accordingly.

Crystallographic data have been deposited with the CCDC (CCDC 2240192 and 2240193).

Table S1 Crystallographic data

Identification code	**RD3**	**RD4**
Empirical formula	$C_{77}H_{98}ClF_6N_6O_7$	$C_{78}H_{109}N_7O_9$
Formula weight	1369.06	1288.72
Temperature/K	99.98(11)	100.00(13)
Crystal system	monoclinic	triclinic
Space group	$P2_1/c$	P-1
a/Å	26.3399(13)	9.6910(6)
b/Å	13.9293(4)	19.3092(14)
c/Å	21.5773(8)	20.3985(11)
α/°	90	77.720(5)
β/°	113.588(5)	78.019(5)
γ/°	90	77.309(6)
Volume/Å^3	7255.2(6)	3586.4(4)
Z	4	2
ρ_{calc}g/cm^3	1.253	1.193
μ/mm^{-1}	1.069	0.615
F(000)	2916.0	1396.0
Crystal size/mm^3	0.24 × 0.16 × 0.02	0.045 × 0.023 × 0.023
Radiation	Cu Kα (λ = 1.54184)	Cu Kα (λ = 1.54184)
2Θ range for data collection/°	3.66 to 151.712	4.498 to 100.872
Index ranges	-33 ≤ h ≤ 32, −17 ≤ k ≤ 6, −27 ≤ l ≤ 27	-9 ≤ h ≤ 9, −19 ≤ k ≤ 11, −20 ≤ l ≤ 20
Reflections collected	32006	23339
Independent reflections	14115 [R_{int} = 0.0544, R_{sigma} = 0.0800]	7515 [R_{int} = 0.1067, R_{sigma} = 0.1189]
Data/restraints/parameters	14115/363/1025	7515/660/854
Goodness-of-fit on F^2	1.010	0.970
Final R indexes [I>=2σ (I)]	R_1 = 0.0973, wR_2 = 0.2820	R_1 = 0.0683, wR_2 = 0.1653
Final R indexes [all data]	R_1 = 0.1477, wR_2 = 0.3205	R_1 = 0.1259, wR_2 = 0.1972
Largest diff. peak/hole / e Å^{-3}	0.57/-0.56	0.35/-0.23
CCDC number	2240192	2240193

4.2. Structural representations

a) b)

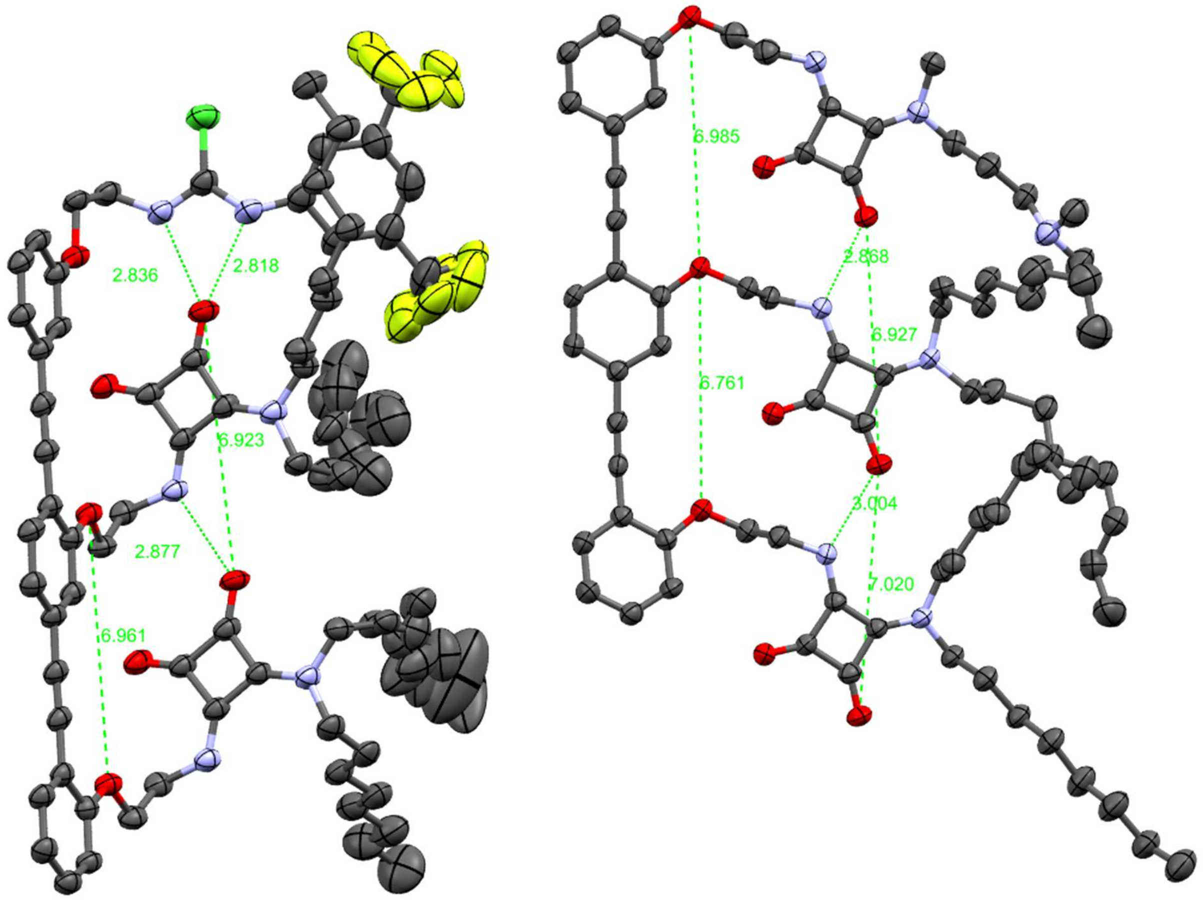

Figure S19. a) ORTEP representation of the asymmetric unit of compound **RD3** (thermal ellipsoids set at 50% probability). b) ORTEP representation of the asymmetric unit of compound **RD4** (thermal ellipsoids set at 50% probability).

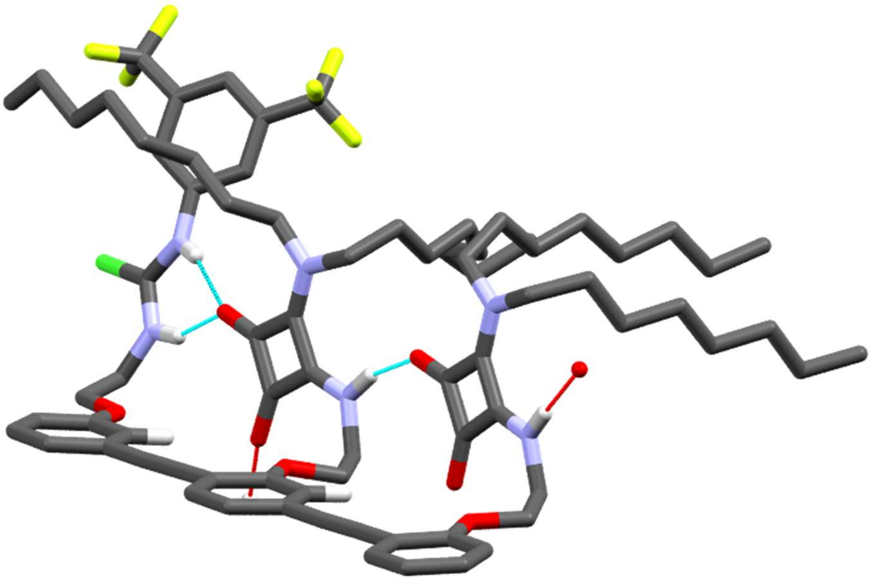

RD3

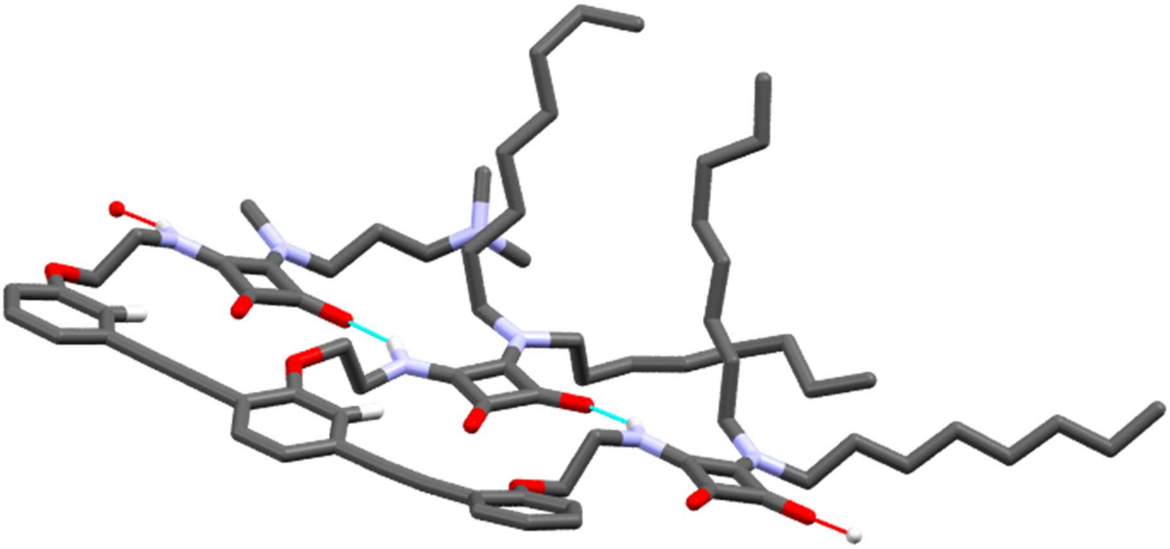

RD4

Figure S20. Stick representations of the crystal structures of compounds **RD3** and **RD4** highlighting the different chemical environment of the aromatic CHs (A_1 and B_1) when the SQ relay is in parallel (**RD3**) and in antiparallel (**RD4**) conformation. Hydrogens shown for polar NHs and for the aromatic CHs of interest.

5. Addition of HBF_4 and TEA to RD4

The effect of HBF_4 on compound **RD4** (in CD_2Cl_2) was monitored by ^1H NMR spectroscopy upon addition of increasing amounts of a stock solution of $HBF_4·Et_2O$ (in CD_2Cl_2) at 298 K (Figures S21 and S22). 1.4 equivalents of HBF_4 were required to complete protonation; further addition of acid did not cause significant changes in the relevant signals.

Protonation-deprotonation cycles of **RD4** were studied by addition of either $HBF_4·Et_2O$ or triethylamine (TEA) stock solutions (see details below). To ensure the final protonation or deprotonation states were reached, successive additions of the corresponding solution were required each time (Figure S23). The volume of $HBF_4·Et_2O$ stock solution required was different for each cycle, probably due to its degradation over time (this solution was freshly prepared at the beginning of the experiment and after the second cycle). The complete ^1H NMR spectra of the fully protonated and deprotonated states are shown in Figure S24. The final solution was concentrated to the original volume to prove that there is no significant effect due to dilution caused by the aliquots of stock solutions (Figure S25). This solution was then used to characterize the protonated state **RD4-H$^+$** at 258 K (Figure S26 and S27).

Stock solutions:

- 20 mM $HBF_4·Et_2O$ in CD_2Cl_2: $HBF_4·Et_2O$ (1.90 mg) was dissolved in CD_2Cl_2 (586 µL). All the material used was plastic, to avoid glass etching, and the stock solution was vigorously agitated before each addition, as indicated elsewhere.[S5] The solution was always used within 5 h of preparation.
- 20 mM TEA in CD_2Cl_2: TEA (1.25 mg) was dissolved in of CD_2Cl_2 (619 µL). The solution was always used during the day of preparation.

a.

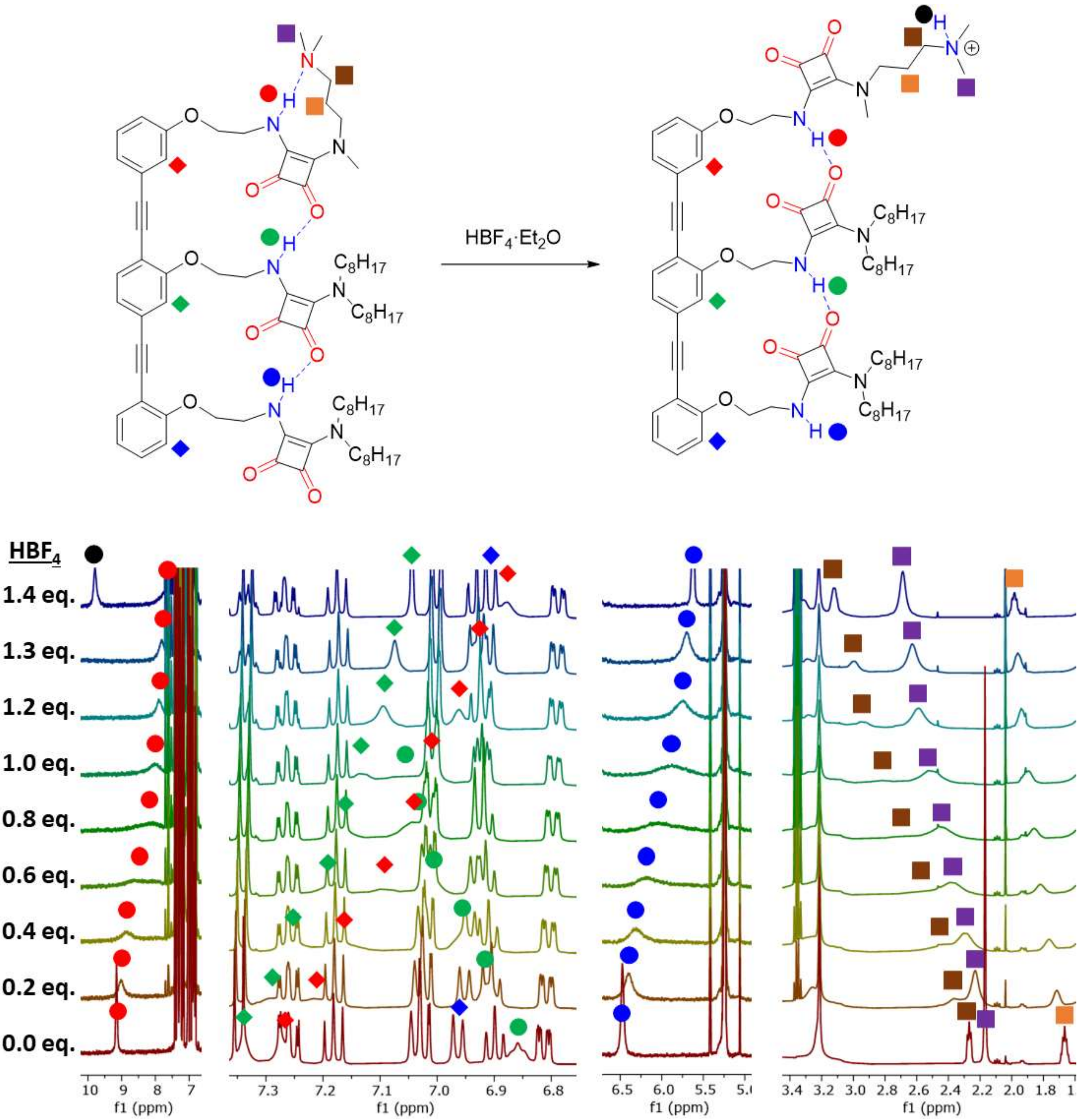

b.

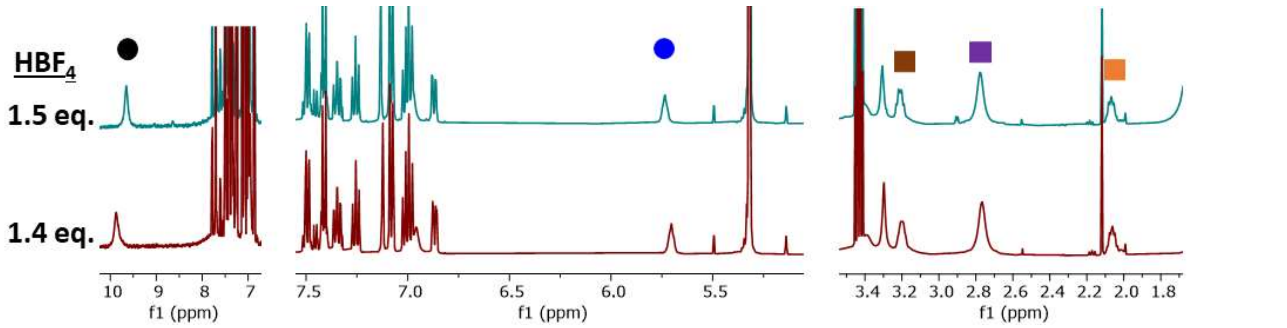

Figure S21. Selected regions of the ^1H NMR spectra (500 MHz, 298 K, CD_2Cl_2) from the titration of compound **RD4** (2 mM) with $HBF_4·Et_2O$ (0 – 3 mM). The number of equivalents of HBF_4 relative to **RD4** is shown. (a) 1.4 equivalents were required for complete protonation. (b) Further addition of HBF_4 caused negligible changes (see also Figure S22b).

a)

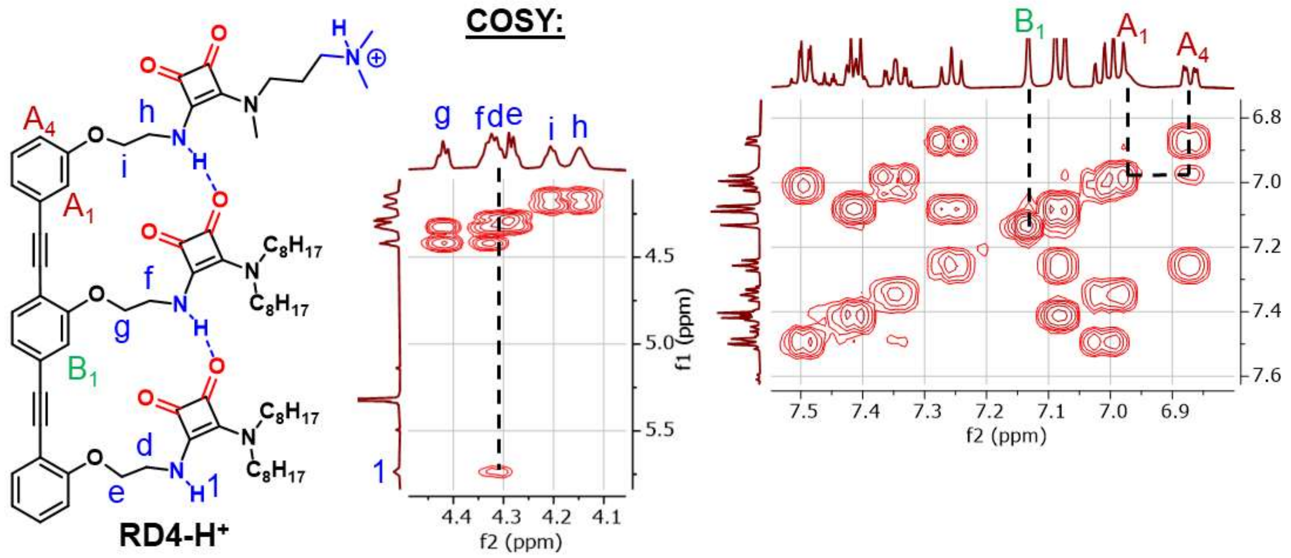

b)

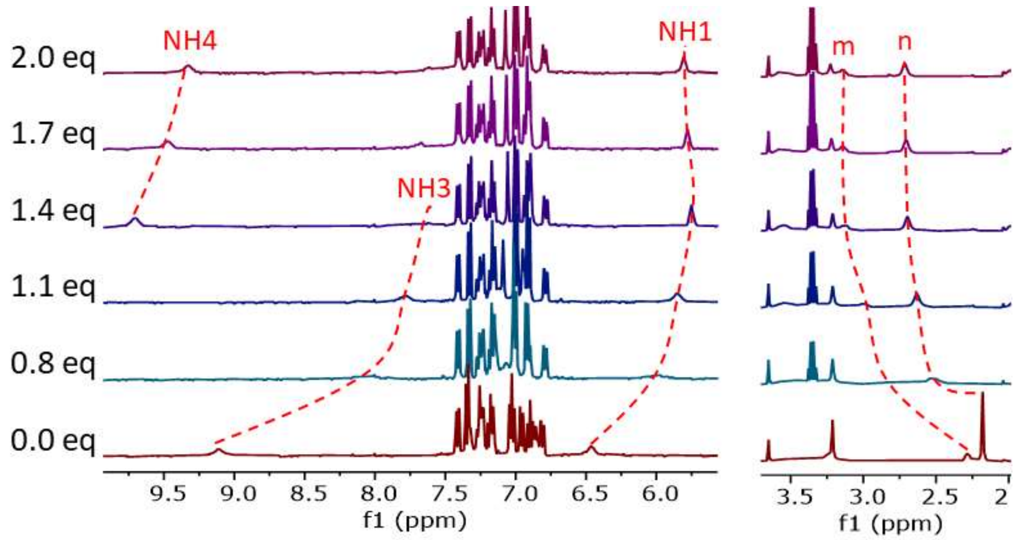

Figure S22. (a) Selected regions of the COSY spectrum (500 MHz, 298 K, CD_2Cl_2) of compound **RD4-H⁺** (final solution from Figure S21). Characterization of **RD4-H⁺** at low temperature is provided in Figure S26. (b) Selected regions of the ^1H NMR spectra (400 MHz, 298 K, CD_2Cl_2) from a titration of compound **RD4** (4 mM) with $HBF_4{\cdot}Et_2O$ (0 – 8 mM). This experiment confirms that protonation is complete after 1.4 equivalents, as reported by signals "n" and "m" adjacent to the amino group. Signal NH1 starts to move downfield upon addition of excess of acid (after 1.4 equivalents), but the change is very subtle compared to the effect observed from protonation, which suggests that the orientation of the squaramide array is not affected after complete protonation. Signals NH2 and NH3 become broad and overlap with the aromatic signals after complete protonation of the molecule. The signal most affected by the addition of excess of acid is NH4 (the NH from the ammonium), probably due to H exchange processes involving the ammonium and the excess of free acid.

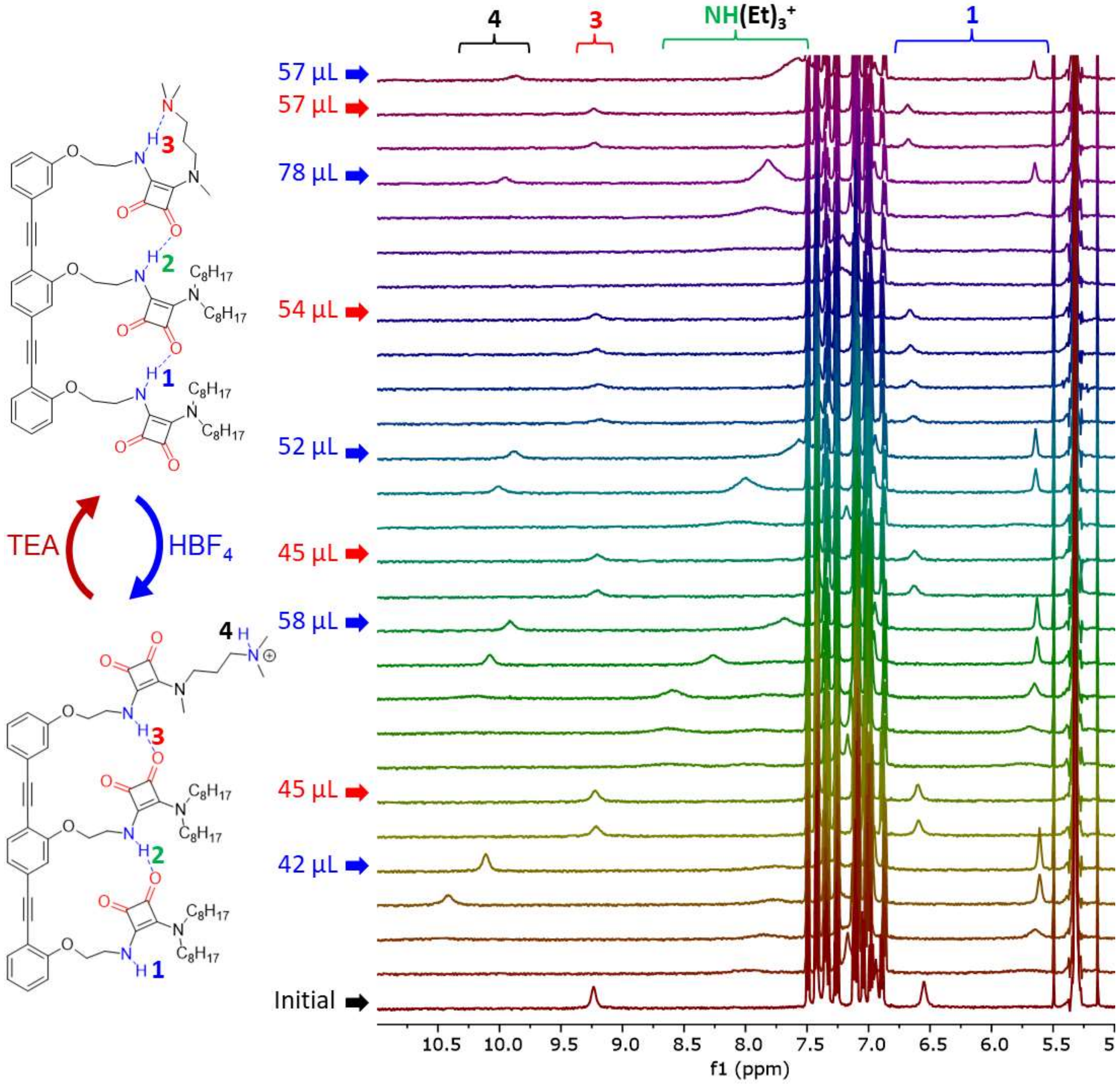

Figure S23. Complete sequence of additions corresponding to the experiment shown in Figure 4 of the main manuscript. Selected region of the ^{1}H NMR spectra (500 MHz, 298 K, CD_2Cl_2) from 5 to 11 ppm. The volumes of $HBF_4{\cdot}Et_2O$ stock solution required to effect complete protonation are indicated in blue, and the volumes of TEA stock solution required to effect complete deprotonation are indicated in red.

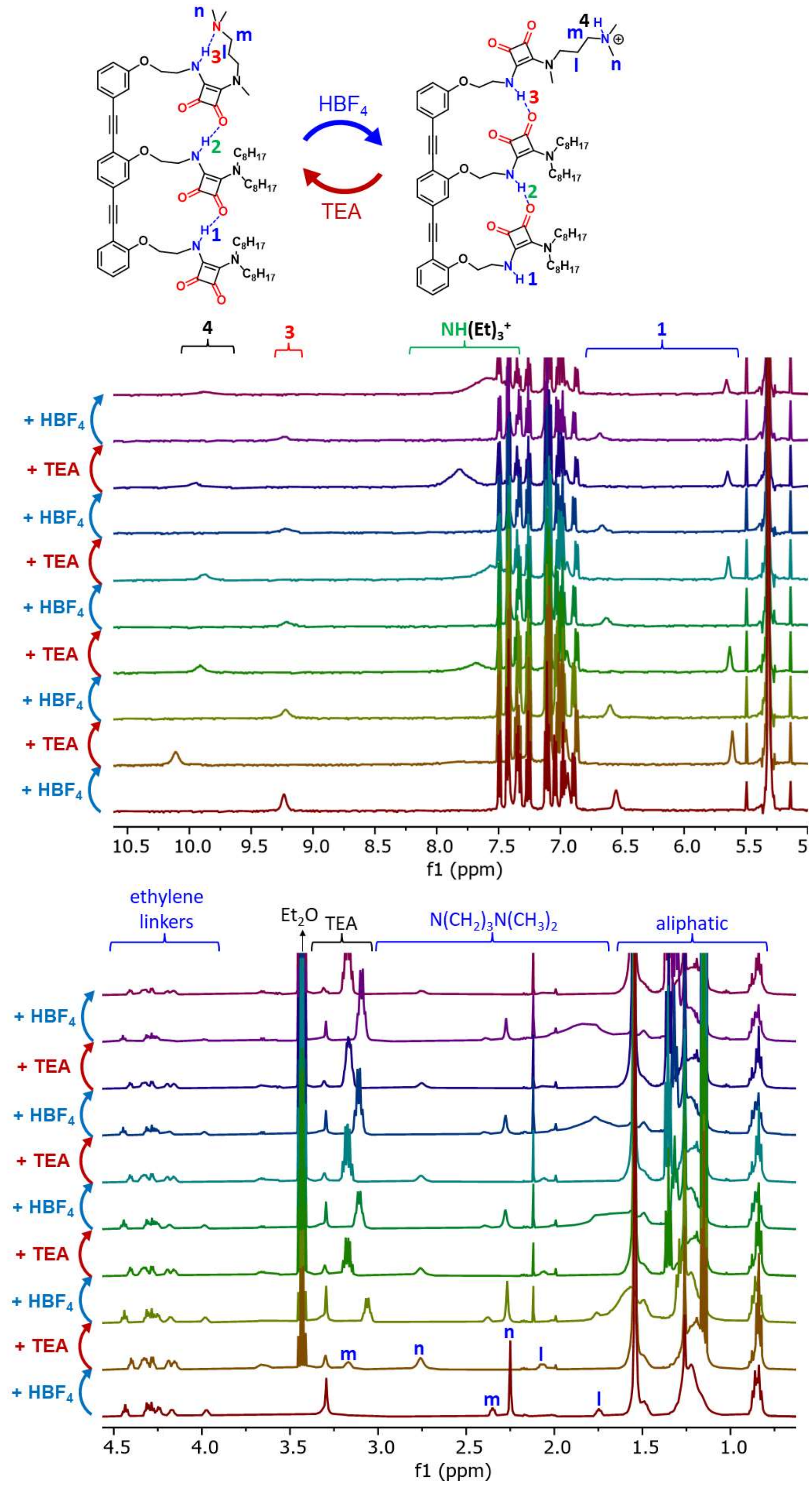

Figure S24. Downfield *(top)* and upfield *(bottom)* ^{1}H NMR spectral regions that contain the expanded regions shown in Figure 5 of the manuscript. The spectra shown here are the $HBF_4 \cdot Et_2O$ or TEA additions labelled with arrows in Figure S23.

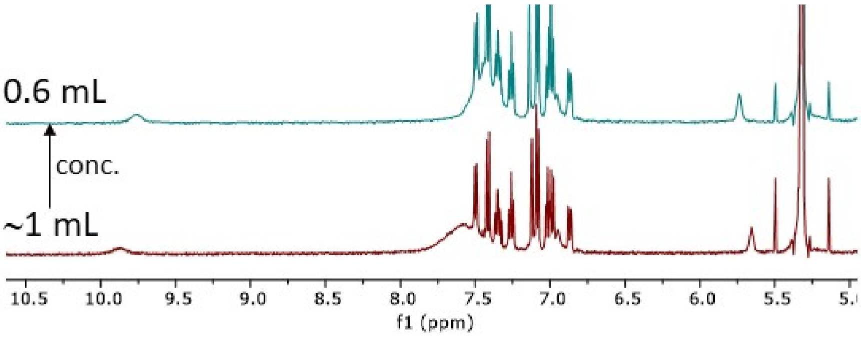

Figure S25. Effect of concentration on the final solution from the sequential additions shown in Figure S23. The sample was concentrated with a flow of N_2 to regain its initial volume.

a.

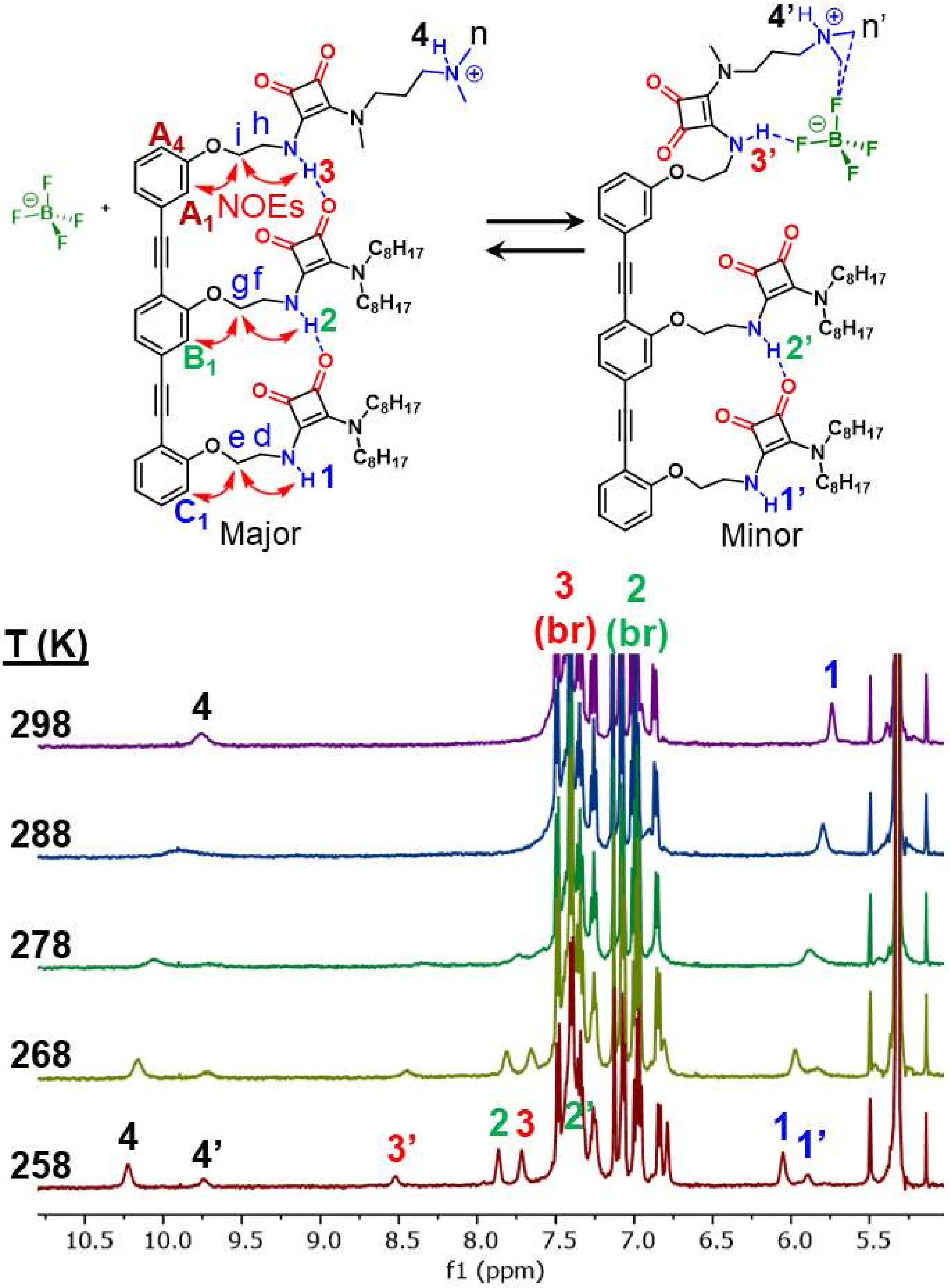

(See legend in next page)

b.

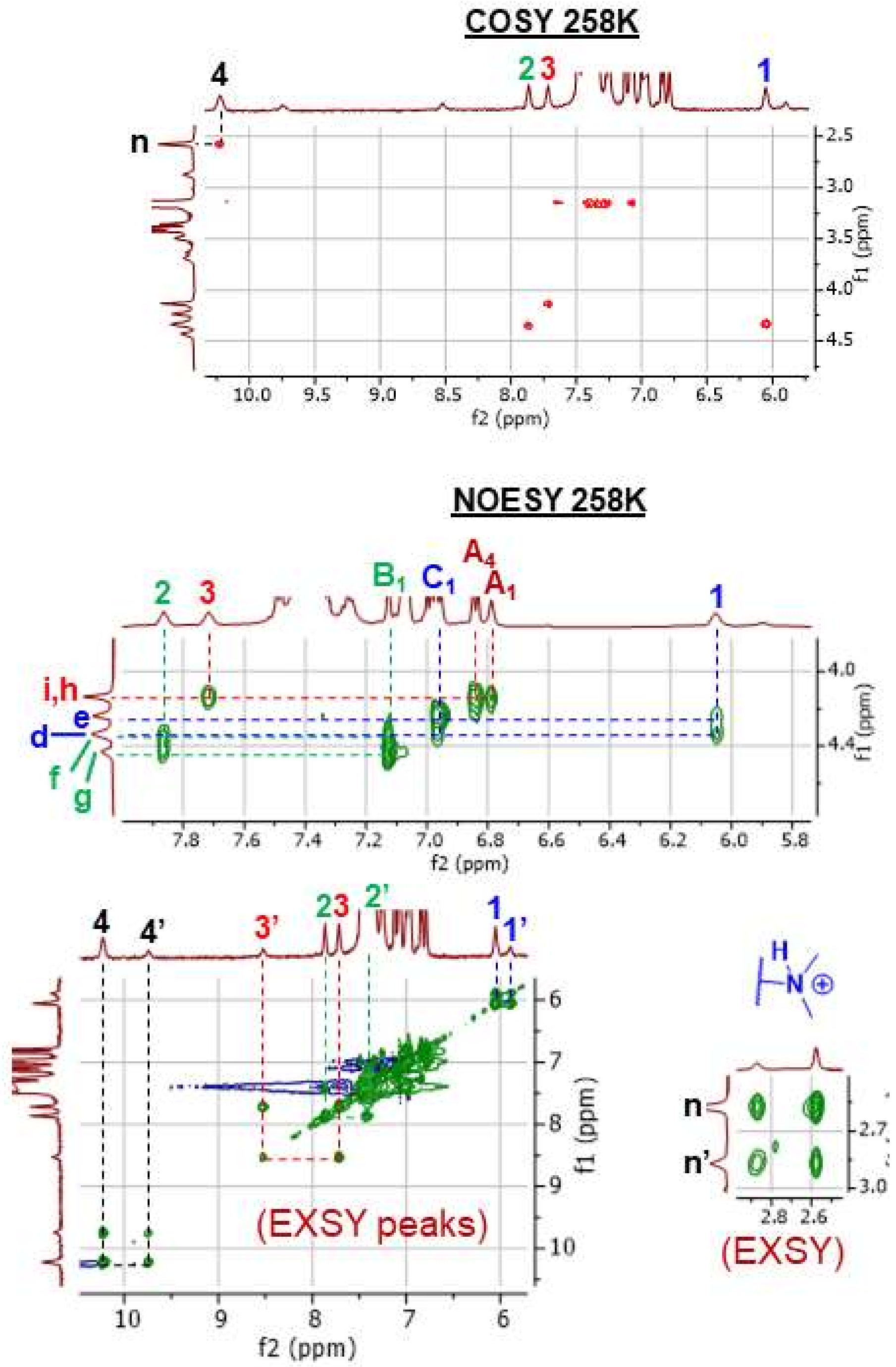

Figure S26. a. Selected region of the ^{1}H NMR spectra (500 MHz, CD_2Cl_2) of compound **RD4-H⁺** (1 mM) at different temperatures after four HBF_4-TEA cycles (solution in 0.6 mL, see Figure S25), and proposed representation of the two species observed at 258 K. The conformers proposed would explain the similar chemical shift of signals 1/1' (NH1, major/minor species) and 2/2' (NH2, major/minor species) and a much larger downfield change in the position of 3' (NH3, minor species) compared to 3 (NH3, major species). b. Selected region of the COSY and NOESY spectra (500 MHz, CD_2Cl_2, 258 K) used to assign the 1D spectrum at 258 K.

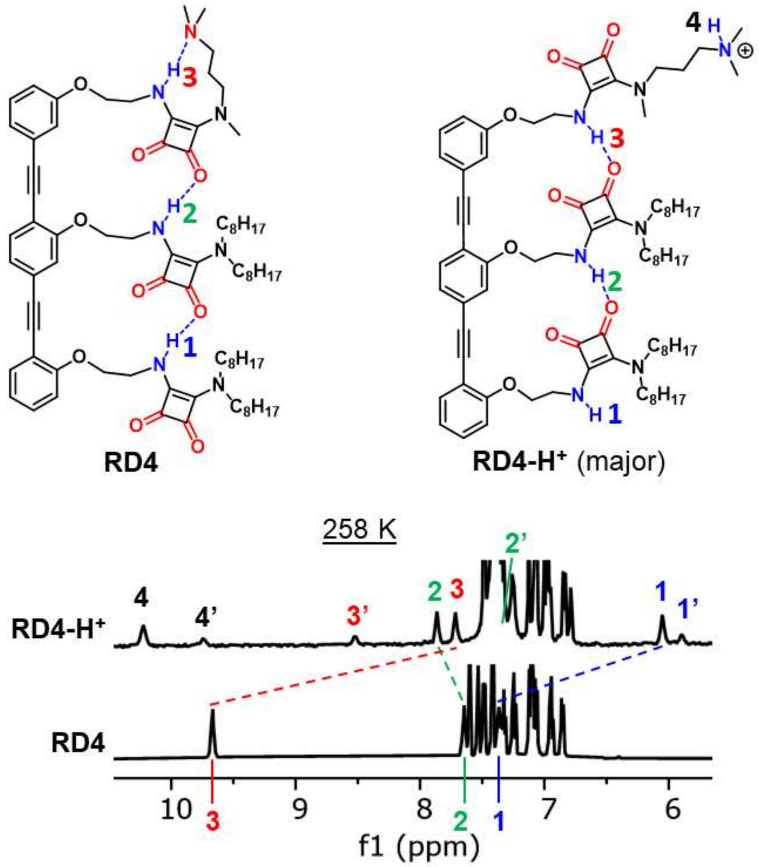

Figure S27. a. Selected region of the ^{1}H NMR spectra (500 MHz, CD_2Cl_2) of the neutral and protonated forms of compound **RD4** (1 mM) at 258 K. The comparison shows large differences for NH3 and NH1, in agreement with the different environments (exposed or internally hydrogen bonded) experienced by those NHs in the two proposed conformers, and a little difference for NH2, which is internally hydrogen bonded in both **RD4** and **RD4-H^{+}**.

6. Addition of CCl_3COOH to RD4

The effect of CCl_3COOH on compound **RD4** was monitored by adding CCl_3COOH (1.04 equivalents, 12.5 µL of a stock solution) to a solution of **RD4** (1 mM, 600 µL CD_2Cl_2), in an NMR tube. The ^1H NMR spectrum of the sample was recorded before the addition of the acid, and at different times after the addition of the acid (0 – 54 h) (Figure S30, Figures 5 and 6 in the main manuscript). The final spectrum was identical to that before the addition of the CCl_3COOH, but with an additional peak of $CHCl_3$ (Figure S28). The progressive appearance of $CHCl_3$ was quantified by integration (Figure S29). During the experiment, CD_2Cl_2 was added to the sample when necessary, to compensate solvent evaporation over time. Slow evaporation may also lead to some loss of the volatile $CHCl_3$ waste generated; only 86 % of $CHCl_3$ (relative to the amount of **RD4**) is measured after complete consumption of 1 equivalent of fuel (Figure S29 and Figure 6b of the main manuscript).

- Stock solution (20 mM CCl_3COOH in CD_2Cl_2): the acid (1.93 mg) was dissolved in CD_2Cl_2 (236 µL). The solution was always used during the day of preparation.

In a preliminary experiment, the addition of a further 0.25 additional equivalents of CCl_3COOH confirmed that 1 equivalent of this acid was sufficient to fully protonate **RD4**. However the extra 0.25 eq. of acid makes the recovery time longer (Figure S33). A second addition of CCl_3COOH after recovery of the initial state of **RD4** proved that the operation cycle can be repeated.

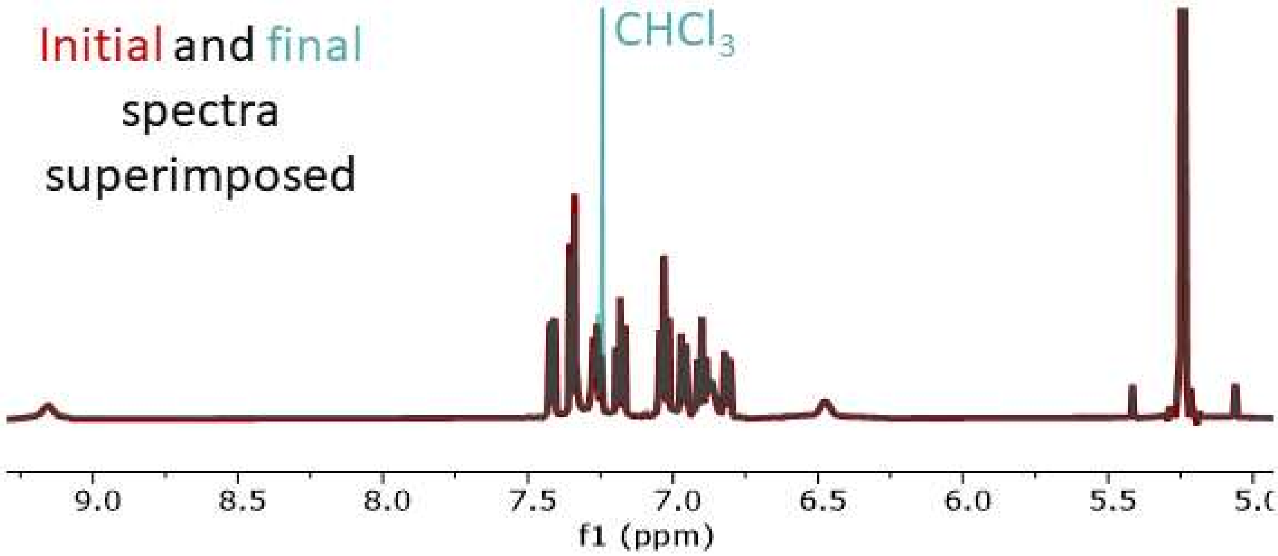

Figure S28. Superimposed selected regions of the ^1H NMR spectra (500 MHz, 298 K, CD_2Cl_2) of compound **RD4** (1 mM) before addition of 1 equivalent of CCl_3COOH and after complete consumption of this acid (initial and final spectra in Figure 6 of the main manuscript).

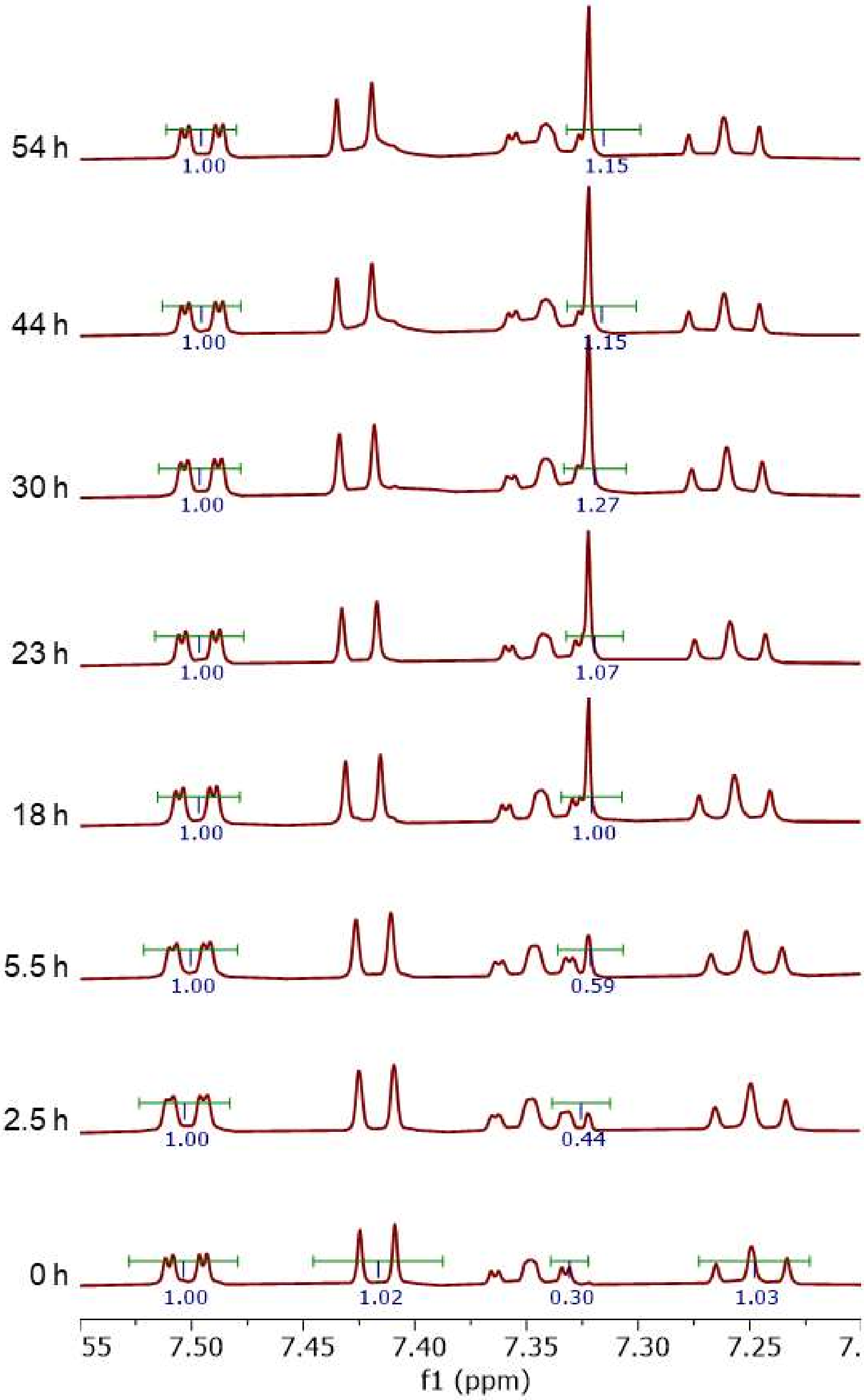

Figure S29. Selected region of the ^{1}H NMR spectra (500 MHz, 298 K, CD_2Cl_2) of **RD4** (1 mM) at different times after the addition of 1.04 equivalents of CCl_3COOH, showing the appearance of $CHCl_3$ at 7.32 ppm. The amount of $CHCl_3$ is quantified by integration, using the doublet at 7.50 ppm as reference and subtracting the section of the triplet at 7.35 ppm that overlaps with the singlet of $CHCl_3$ (*i.e.,* this starting value of 0.30 observed at 0 h is subtracted from the subsequent values obtained).

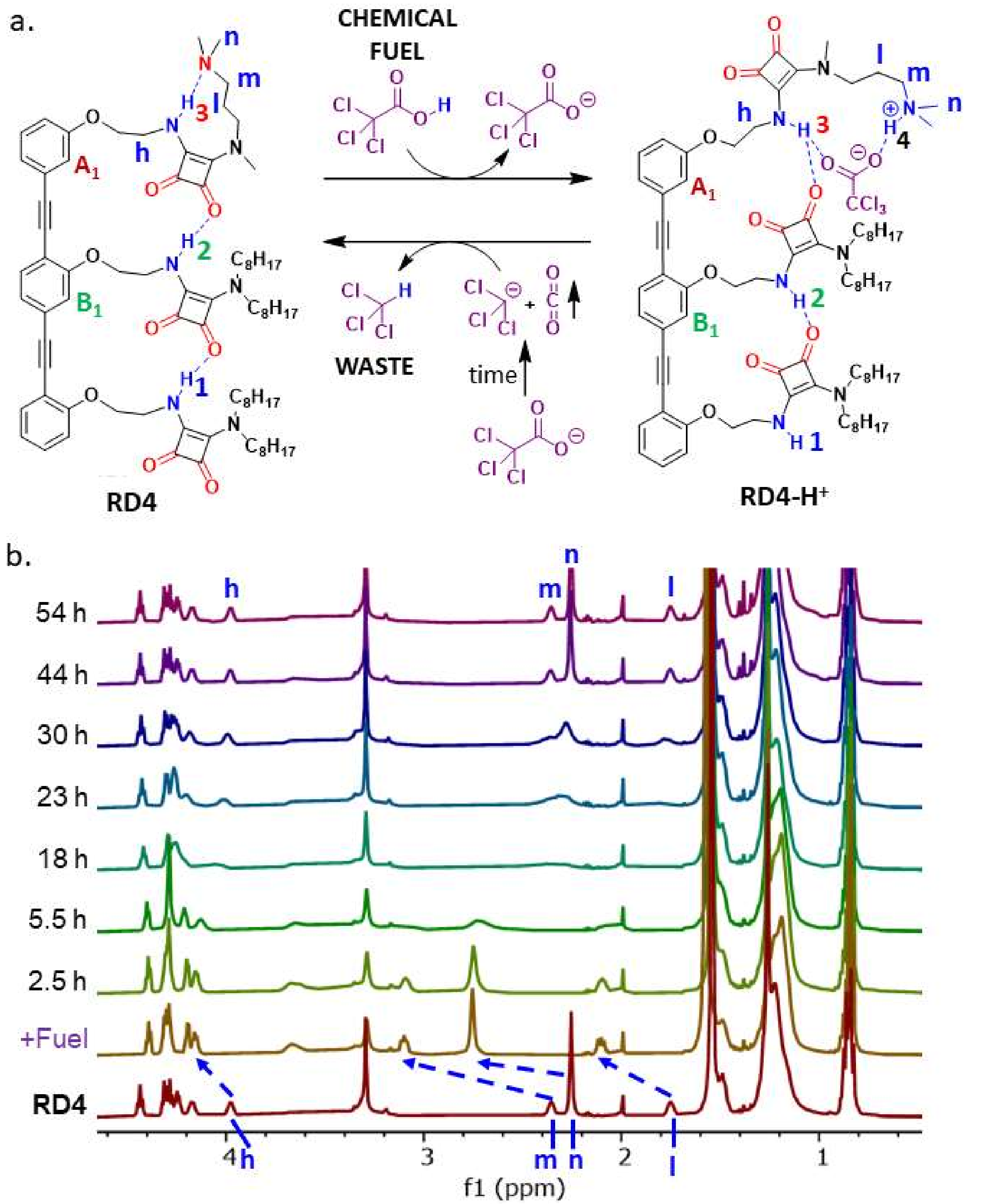

Figure S30. a. Representation of the conformational changes induced on **RD4** by the addition of CCl_3COOH and its subsequent decarboxylation over time. The formation of a proposed **RD4-H**$^+$$\cdots$$CCl_3COO^-$ complex is analogous to the proposed complex with BF_4^-, and explains why NH3 in **RD4-H**$^+$ is further downfield than NH2. It is consistent with the similar chemical shift for NH2 in both **RD4** and **RD4-H**$^+$ (NH2 remains internally hydrogen bonded in both). b. Upfield region of the spectra corresponding to the experiment shown in Figure 6 of the main manuscript. Signals m, n, l report the protonation and deprotonation of the amino group, and signal h was used to monitor the conformational change over time (other signals more affected by the change in protonation state become too broad during the recovery process).

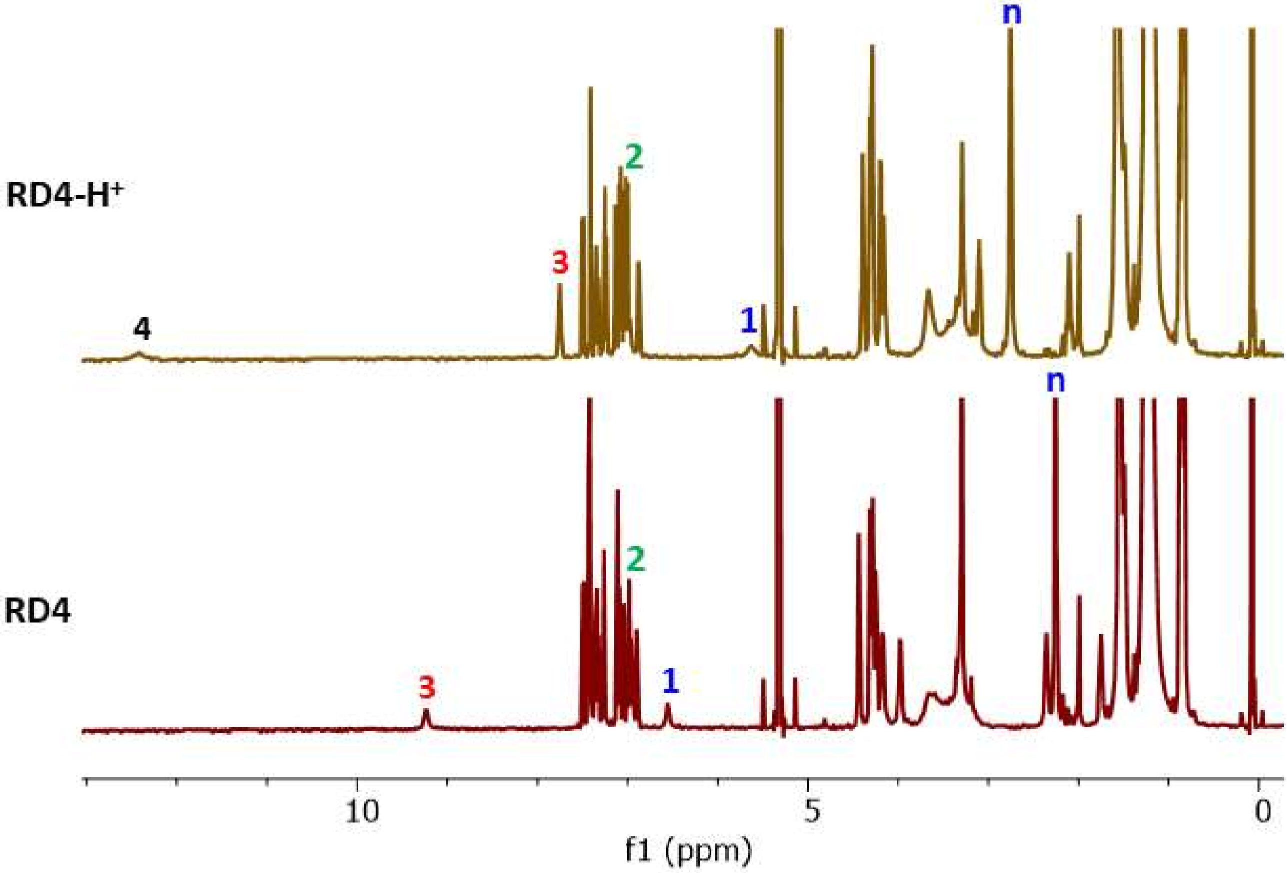

Figure S31. ^1H NMR spectra (500 MHz, 298 K, CD_2Cl_2) of compound **RD4** (1 mM) before and after the addition of 1 equivalent of CCl_3COOH.

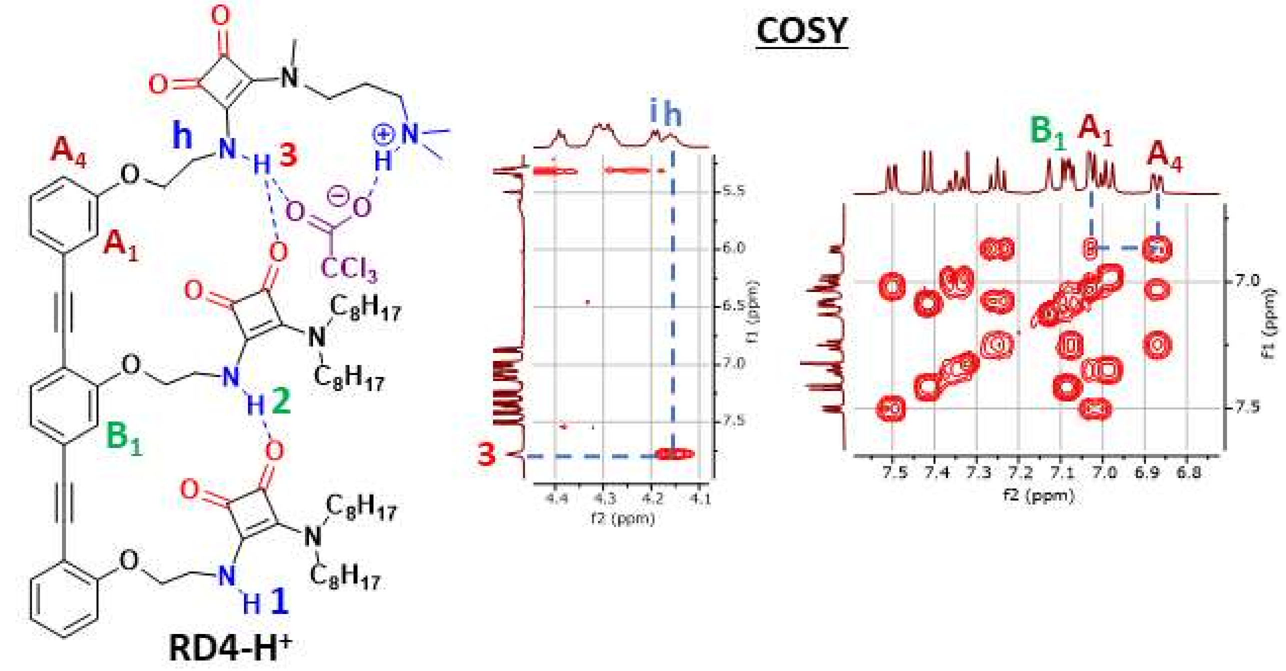

Figure S32. Selected region of the COSY spectrum (500 MHz, CD_2Cl_2) of compound **RD4** (1 mM) in presence of 1.25 equivalents of CCl_3COOH.

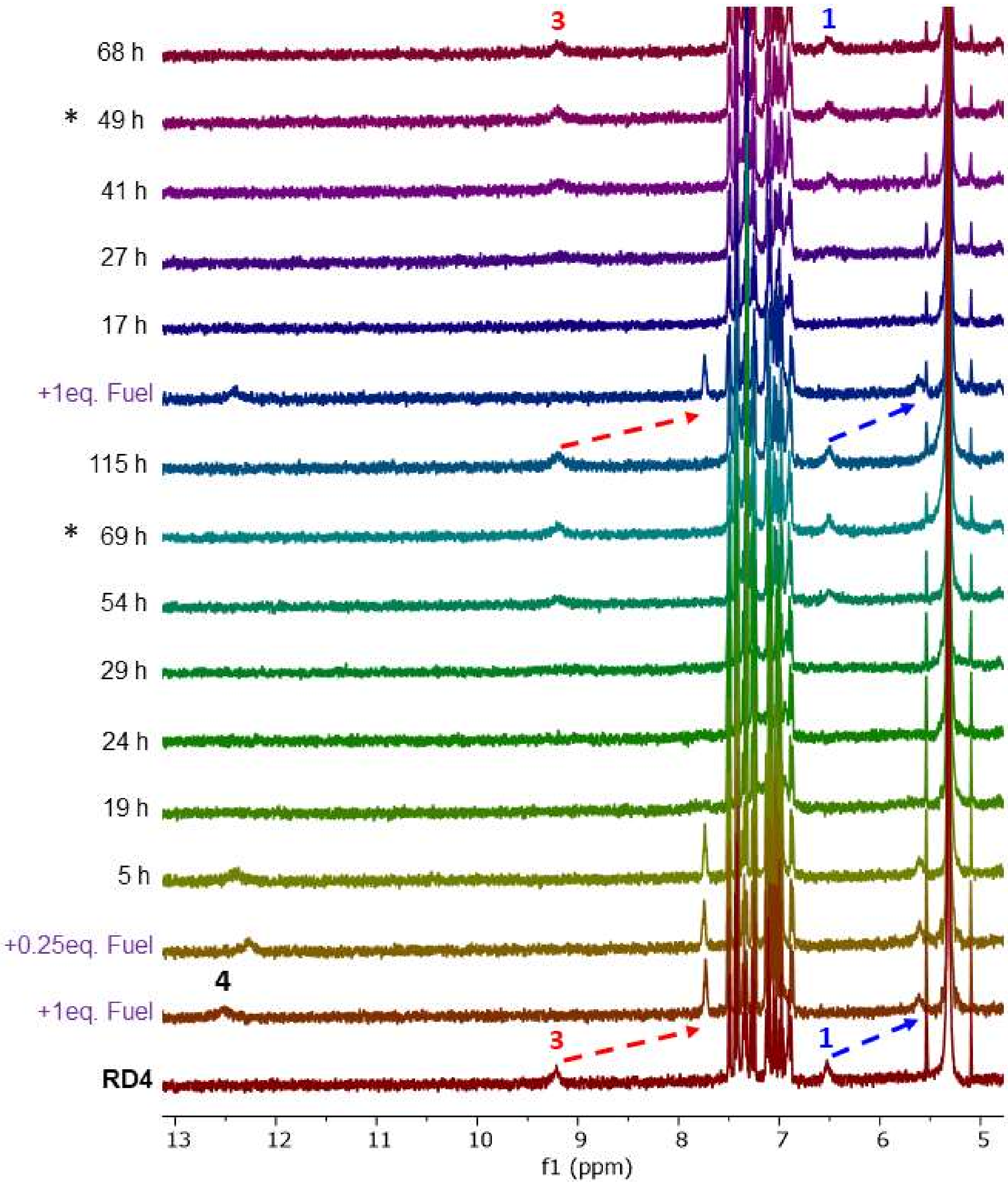

Figure S33. Selected region of the ^{1}H NMR spectra (CD_2Cl_2, 400 MHz, 298 K) of compound **RD4** (1 mM), showing two cycles of chemically fueled conformational inversion. First, 1 equivalent of CCl_3COOH (fuel) caused the conformational switch with further addition producing negligible changes. After complete recovery of the initial state of **RD4** (69 h) the addition of one further equivalent of CCl_3COOH triggered a second cycle. The stars indicate the time of full recovery of the non-protonated state of **RD4** (longer for the first cycle, where 1.25 eq. of fuel had been added).

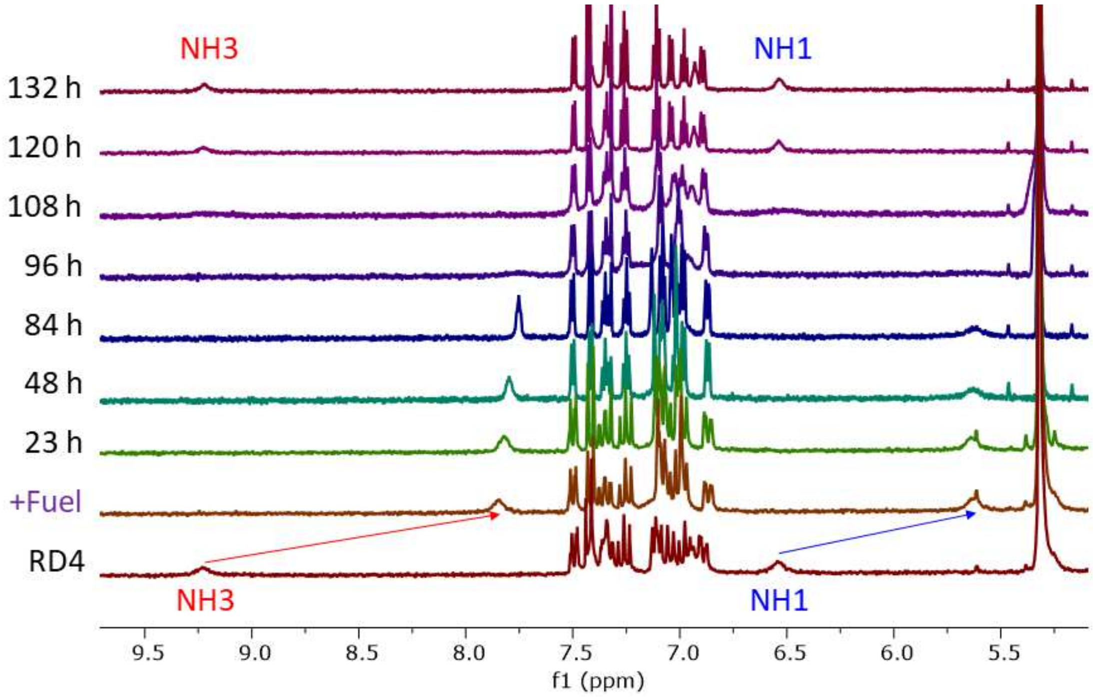

Figure S34. Selected region of the ^{1}H NMR spectra (CD_2Cl_2, 600 MHz, 298 K) of compound **RD4** (1 mM) before and after the addition of 2 equivalent of CCl_3COOH (fuel). The spectra recorded over time show that the parallel conformer **RD4-H$^+$** is maintained for at least 84 h and that 120 h are required for **RD4** to recover its original state. This experiment confirms that the amount of fuel added can determine the time that the device spends as a fully protonated parallel species, and therefore the time required to complete a full communication cycle, as suggested by the experiment in Figure S33.

7. Dilution experiment with RD4.

To rule out the presence of aggregates in solution to levels that could interfere on the conformational characterization of **RD4** in CD_2Cl_2 or on the results of the switching experiments, we have registered the ^{1}H NMR spectra of **RD4** at different concentrations (Figure S35). This experiment shows little effect of concentration on the spectra, since the changes in chemical shift observed are in a much lower range than the difference in chemical shift between parallel and antiparallel orientations.

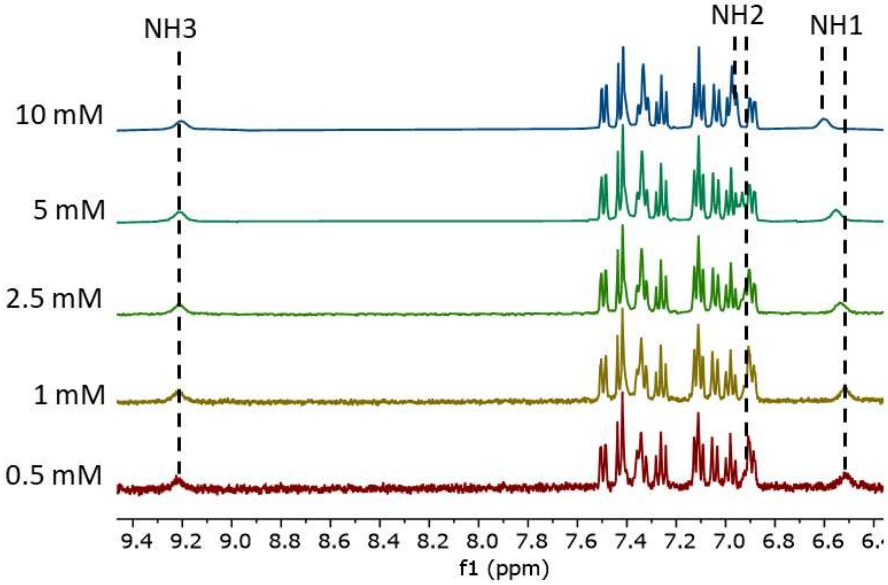

Figure S35. Selected region of the ^{1}H NMR spectra (400 MHz, 298 K, CD_2Cl_2) of compound **RD4** at different concentrations.

8. ¹H, ¹³C and selected 2D NMR spectra of 1-19 and RD1-RD4.

***tert*-Butyl (2-(2-iodophenoxy)ethyl)carbamate (1)**

¹H NMR:

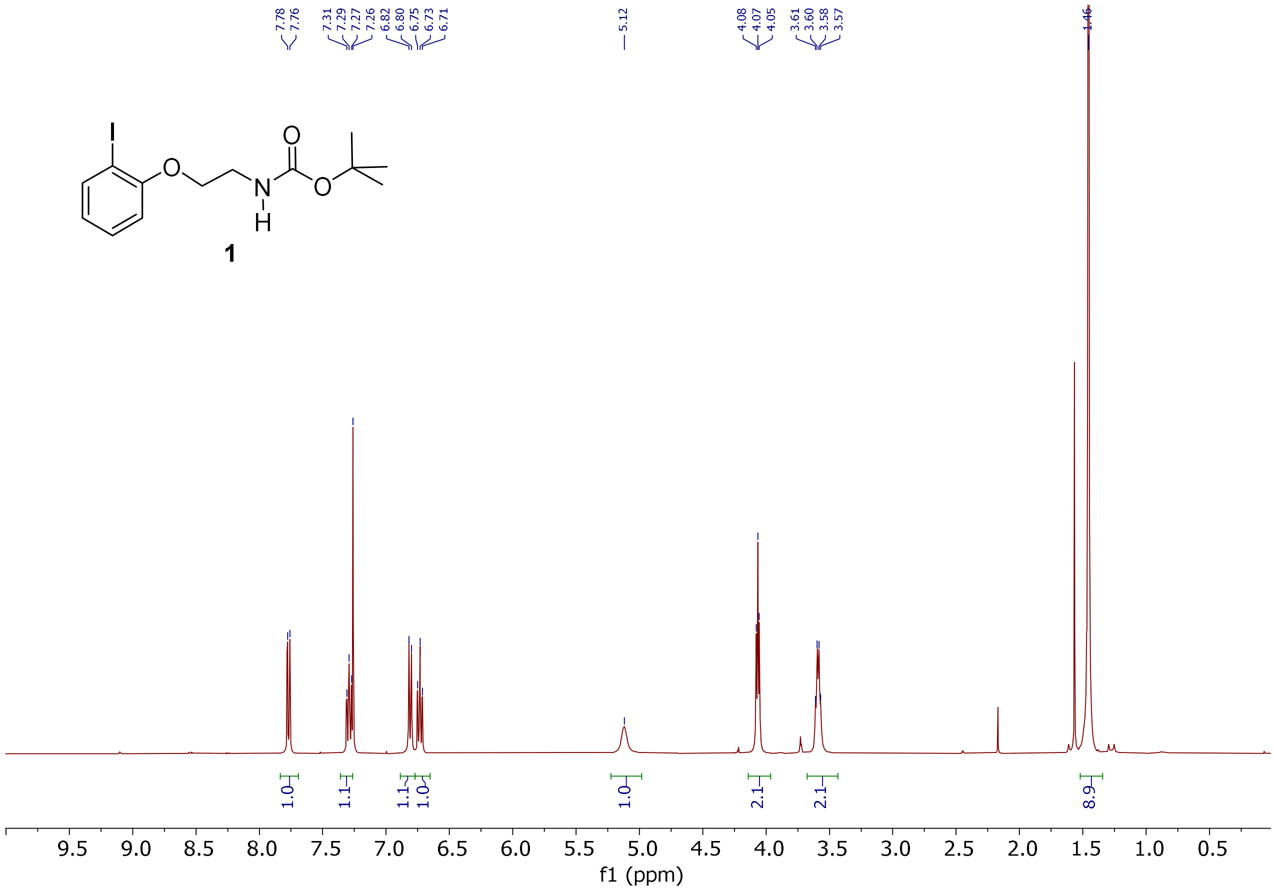

¹³C NMR:

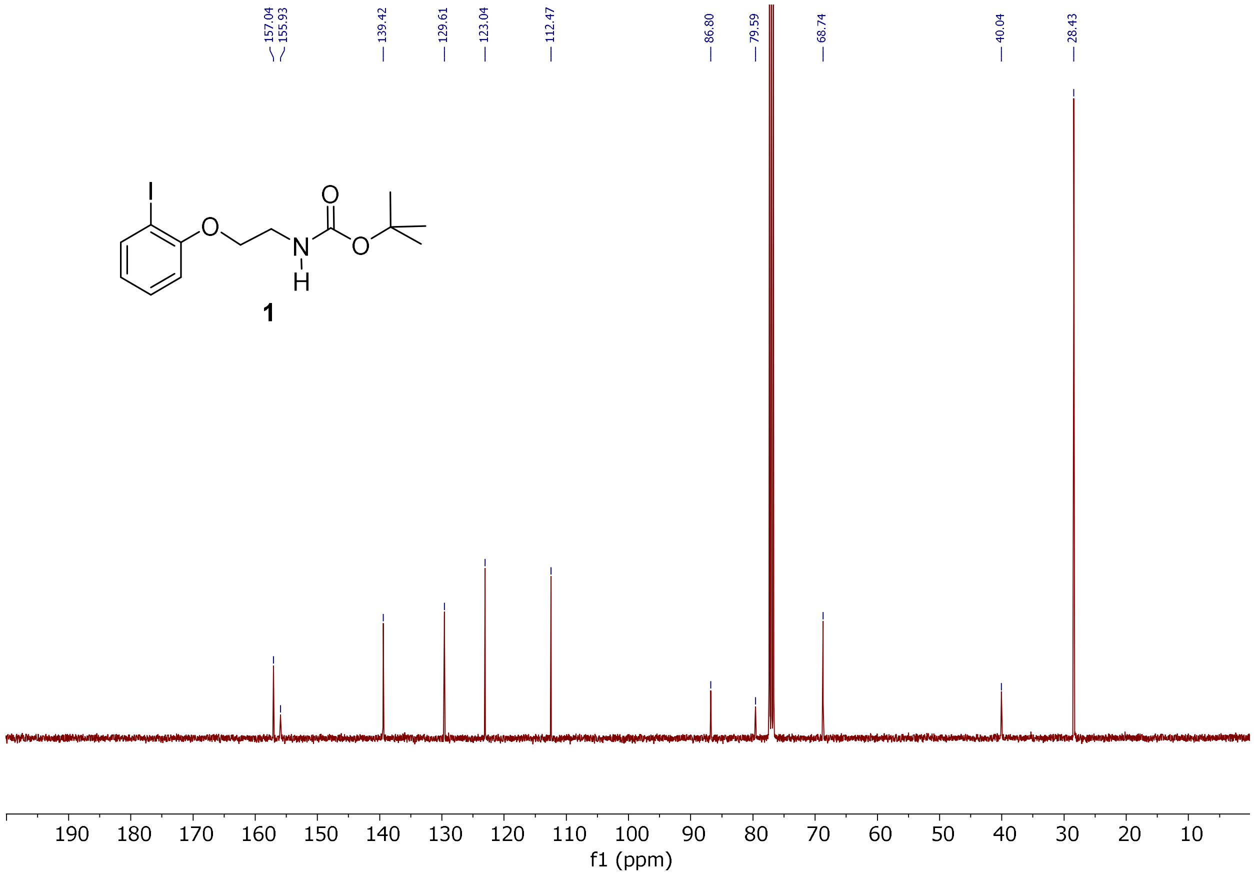

***tert*-Butyl (2-(3-iodophenoxy)ethyl)carbamate (2)**

^{1}H NMR:

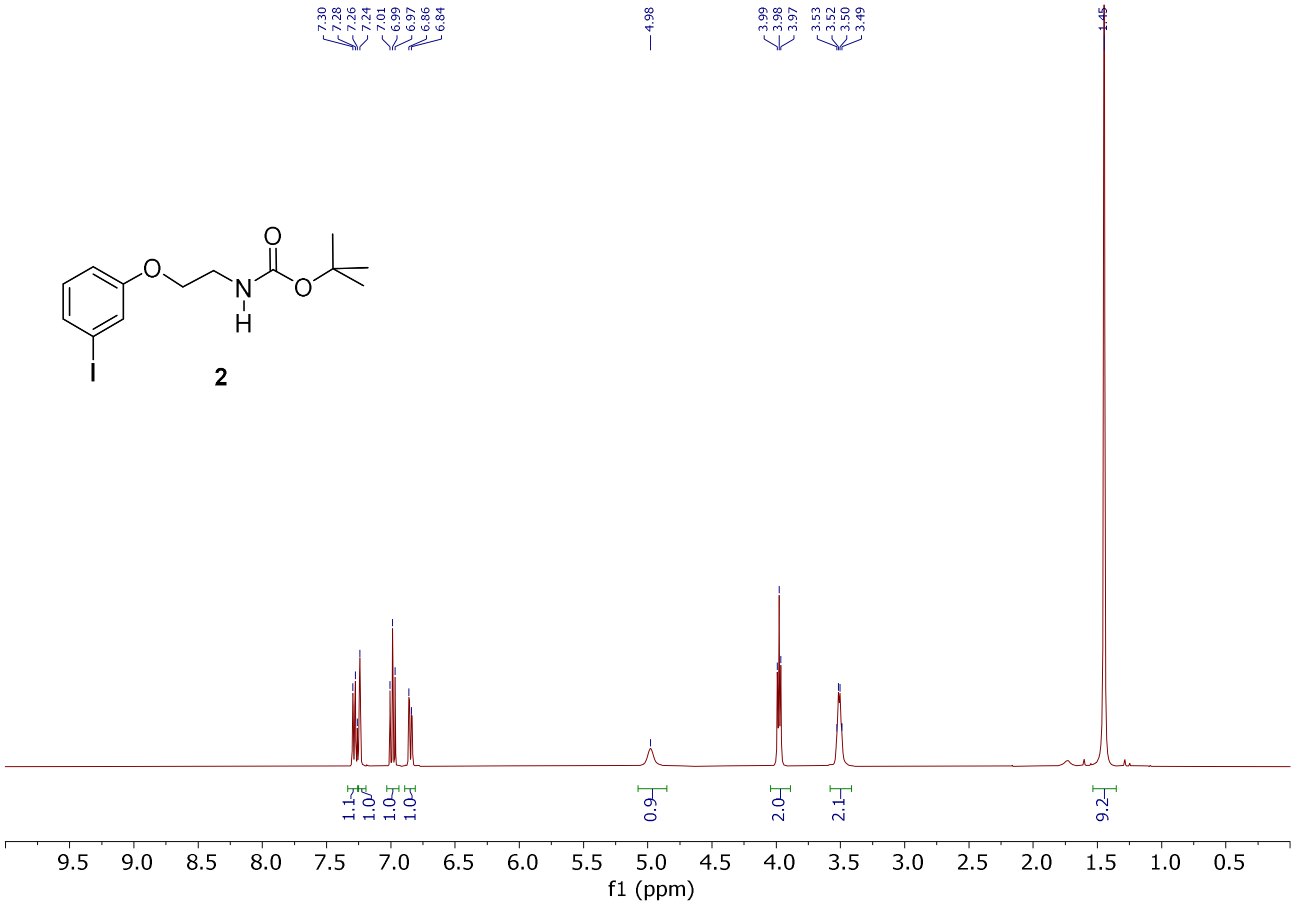

^{13}C NMR:

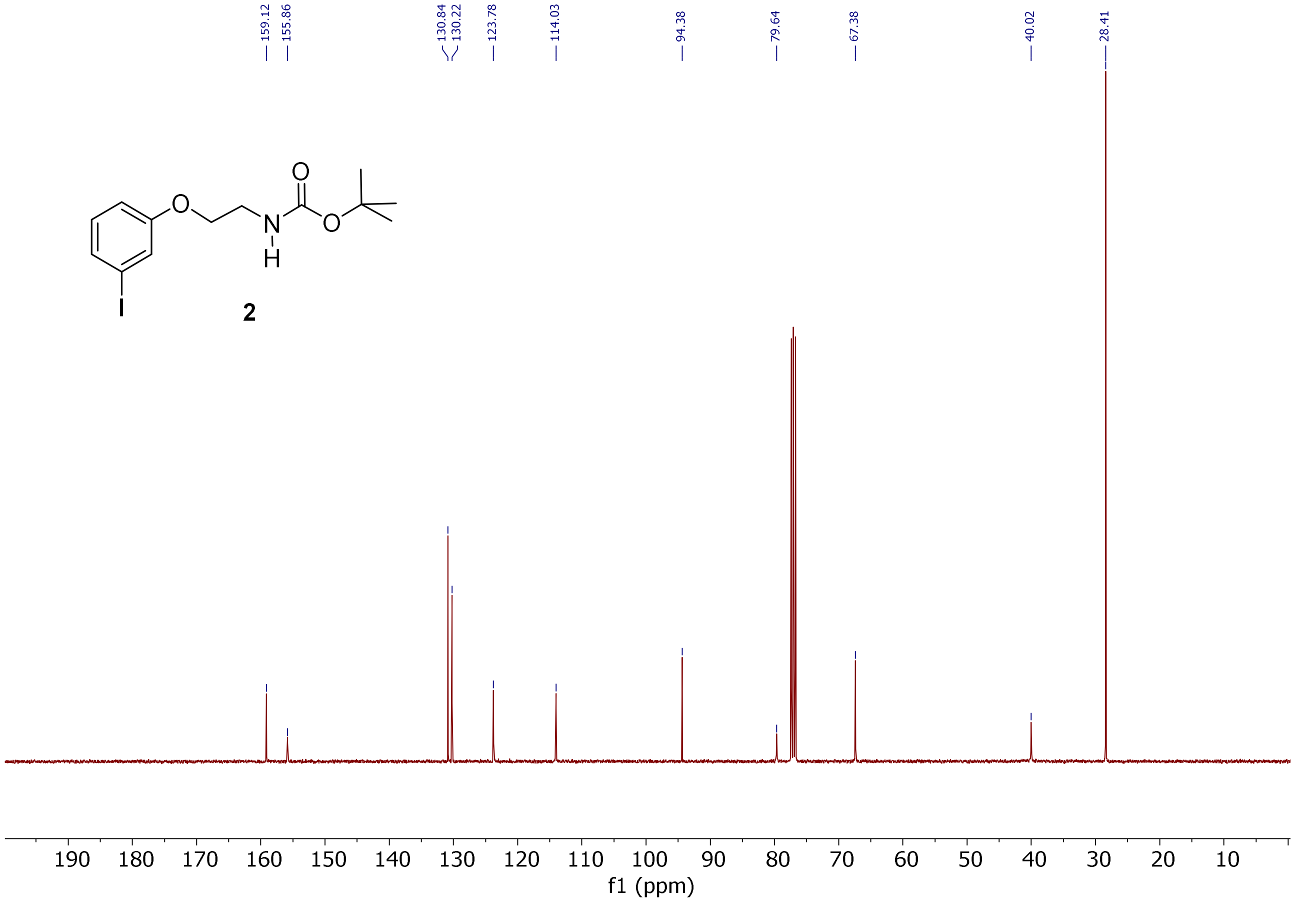

2-Bromo-5-iodophenol (3)[S2]

^{1}H NMR:

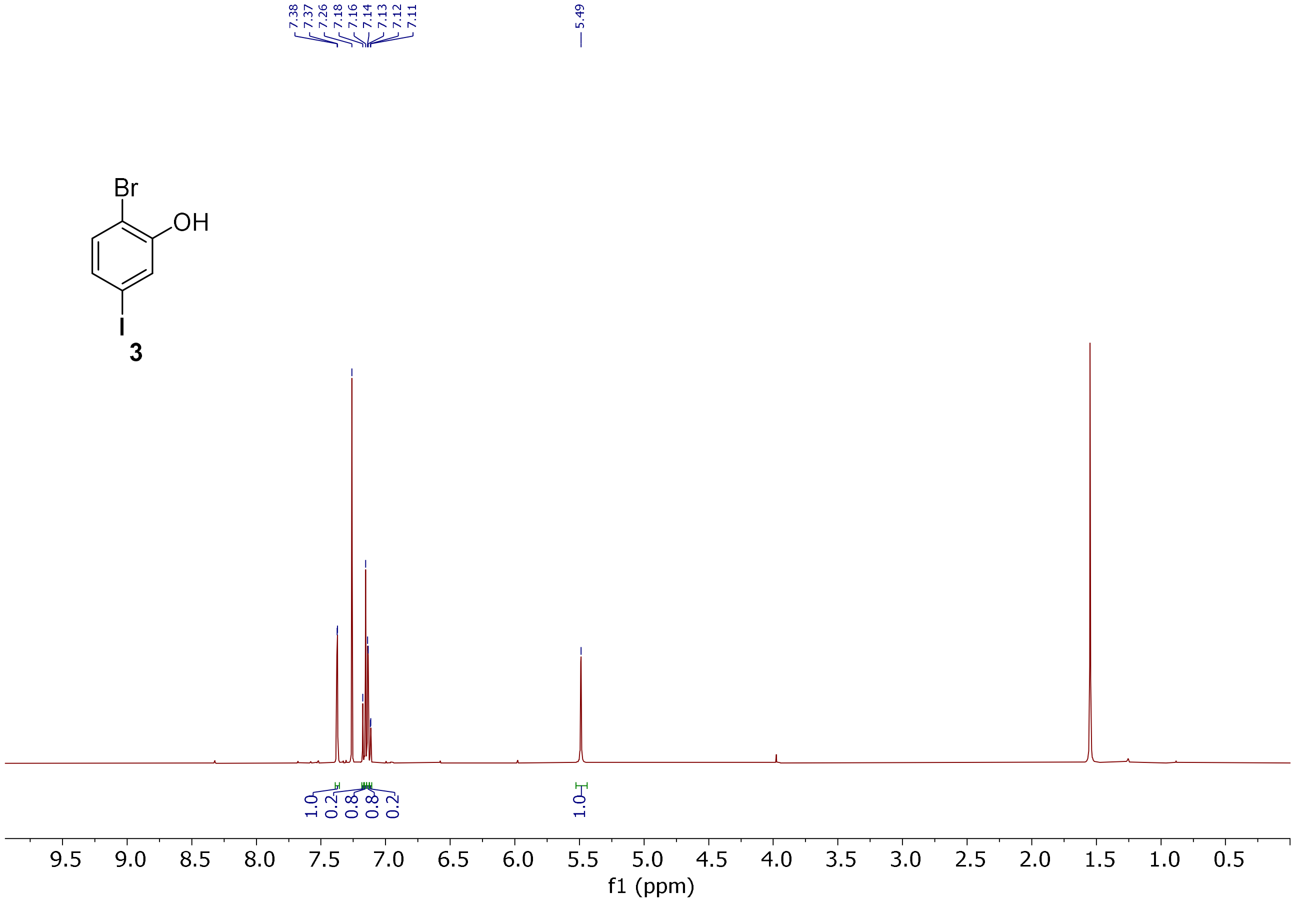

***tert*-Butyl (2-(2-bromo-5-iodophenoxy)ethyl)carbamate (4)**

^{1}H NMR:

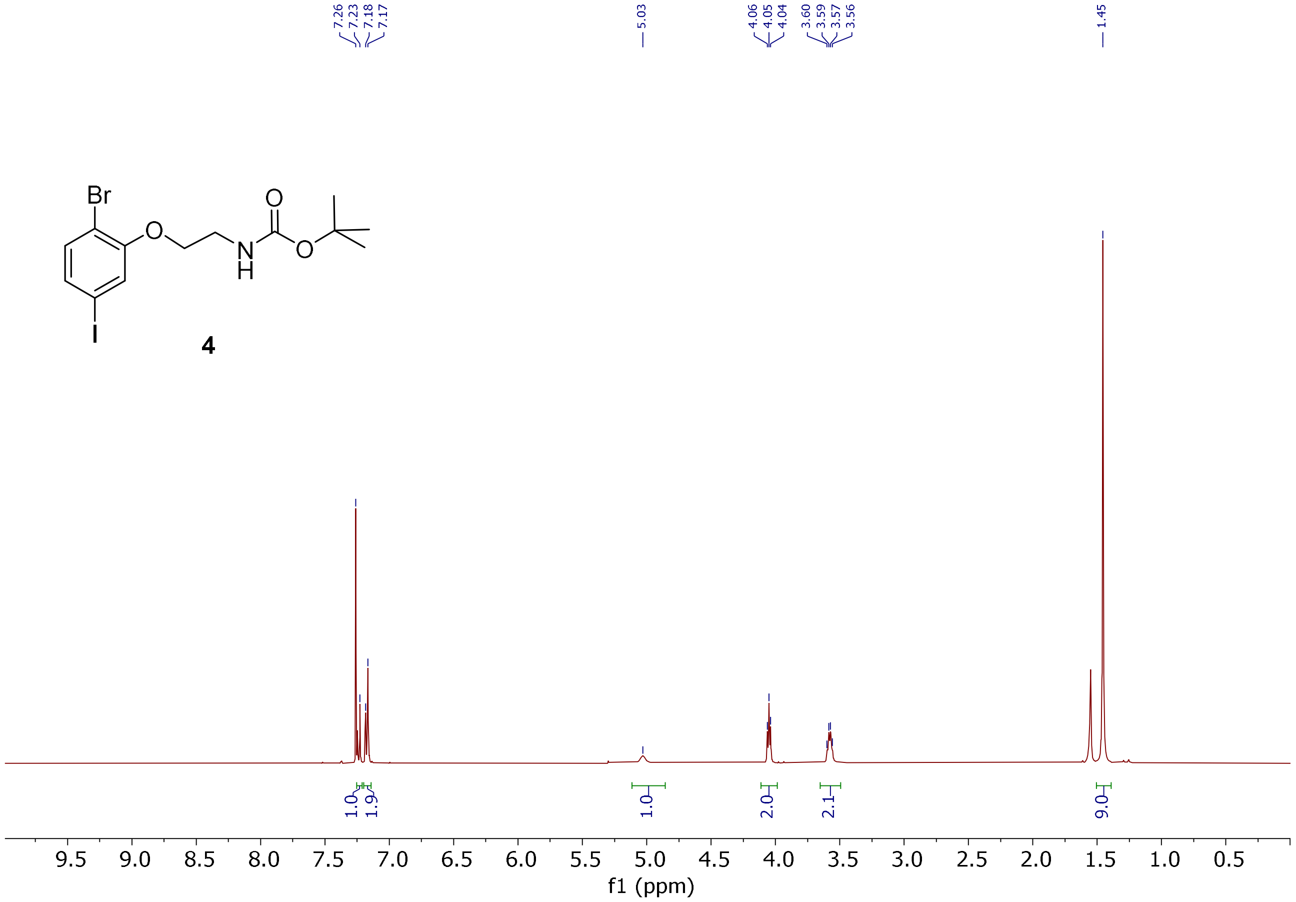

^{13}C NMR:

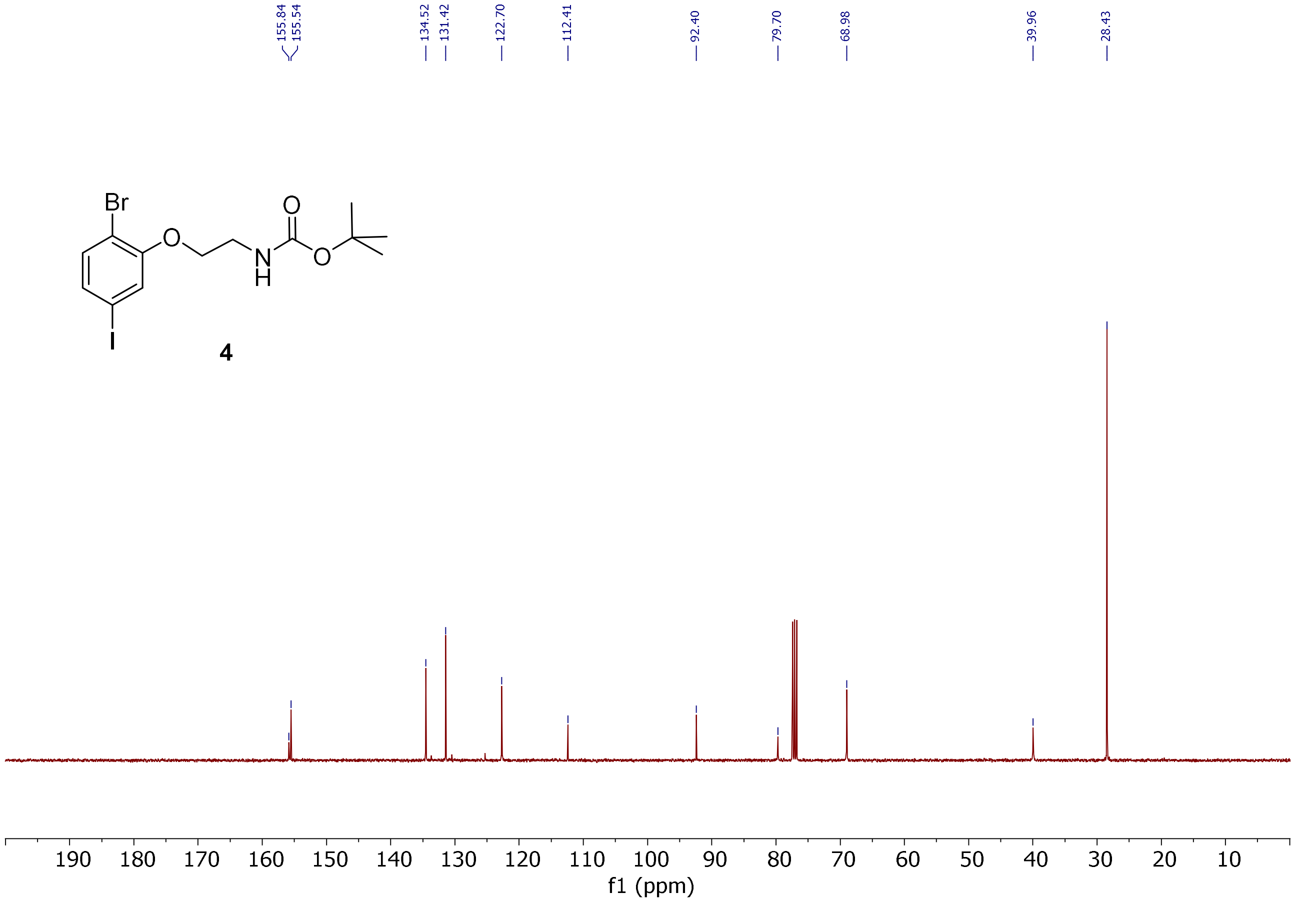

***tert*-Butyl (2-(3-ethynylphenoxy)ethyl)carbamate (5)**

^{1}H NMR:

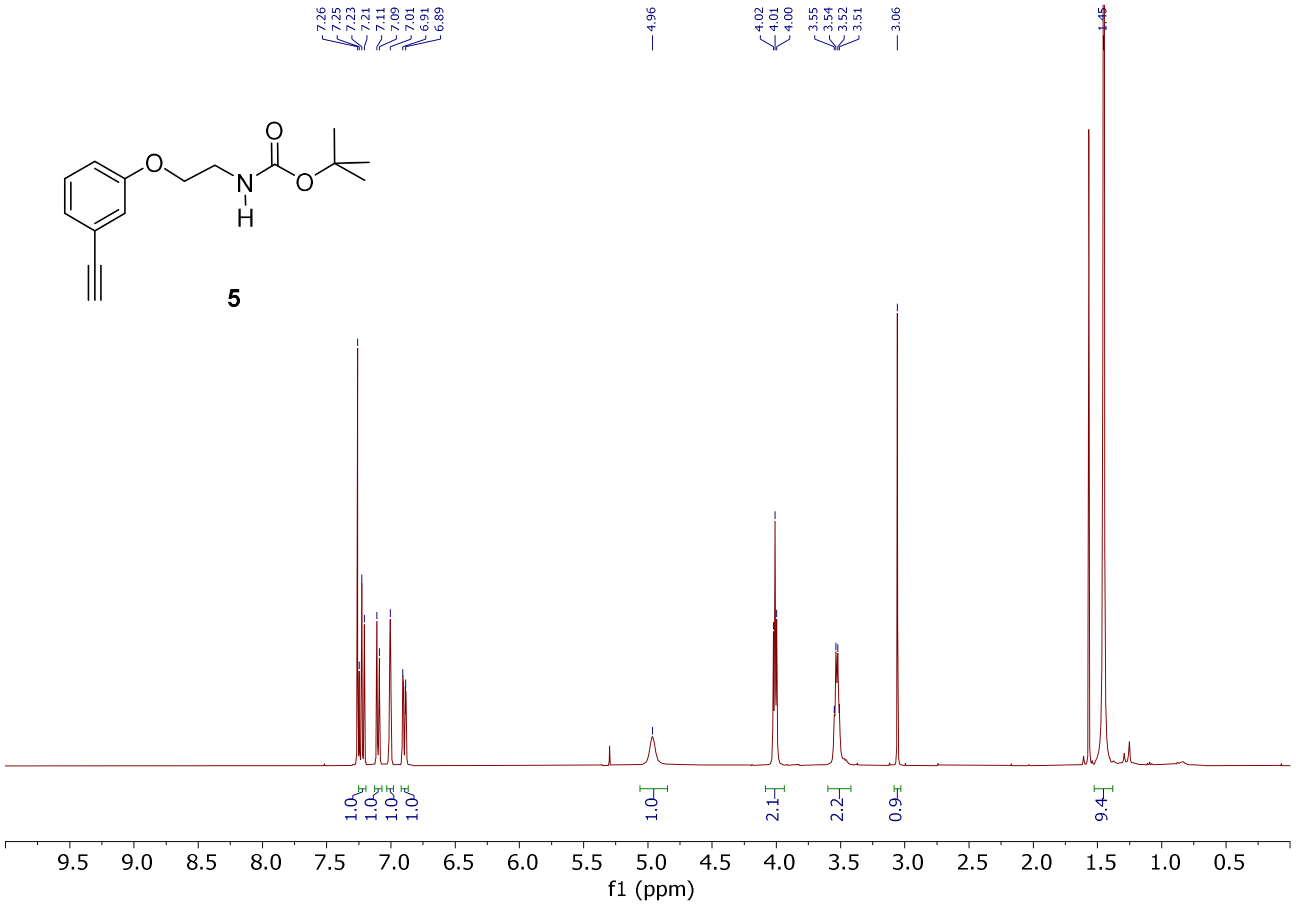

^{13}C NMR:

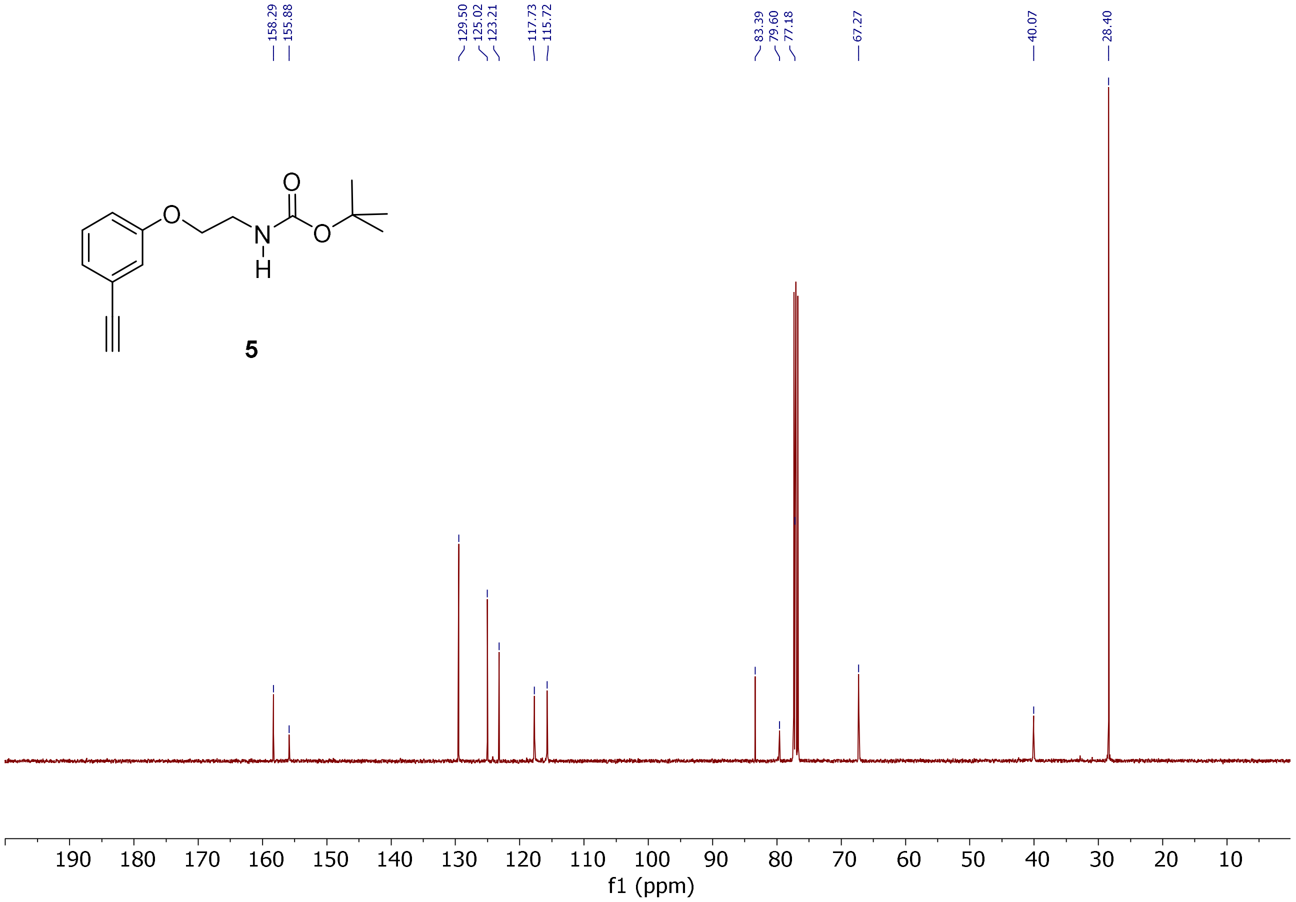

3-(Di(*n*-octyl)amino)-4-methoxycyclobut-3-ene-1,2-dione (6)

^{1}H NMR:

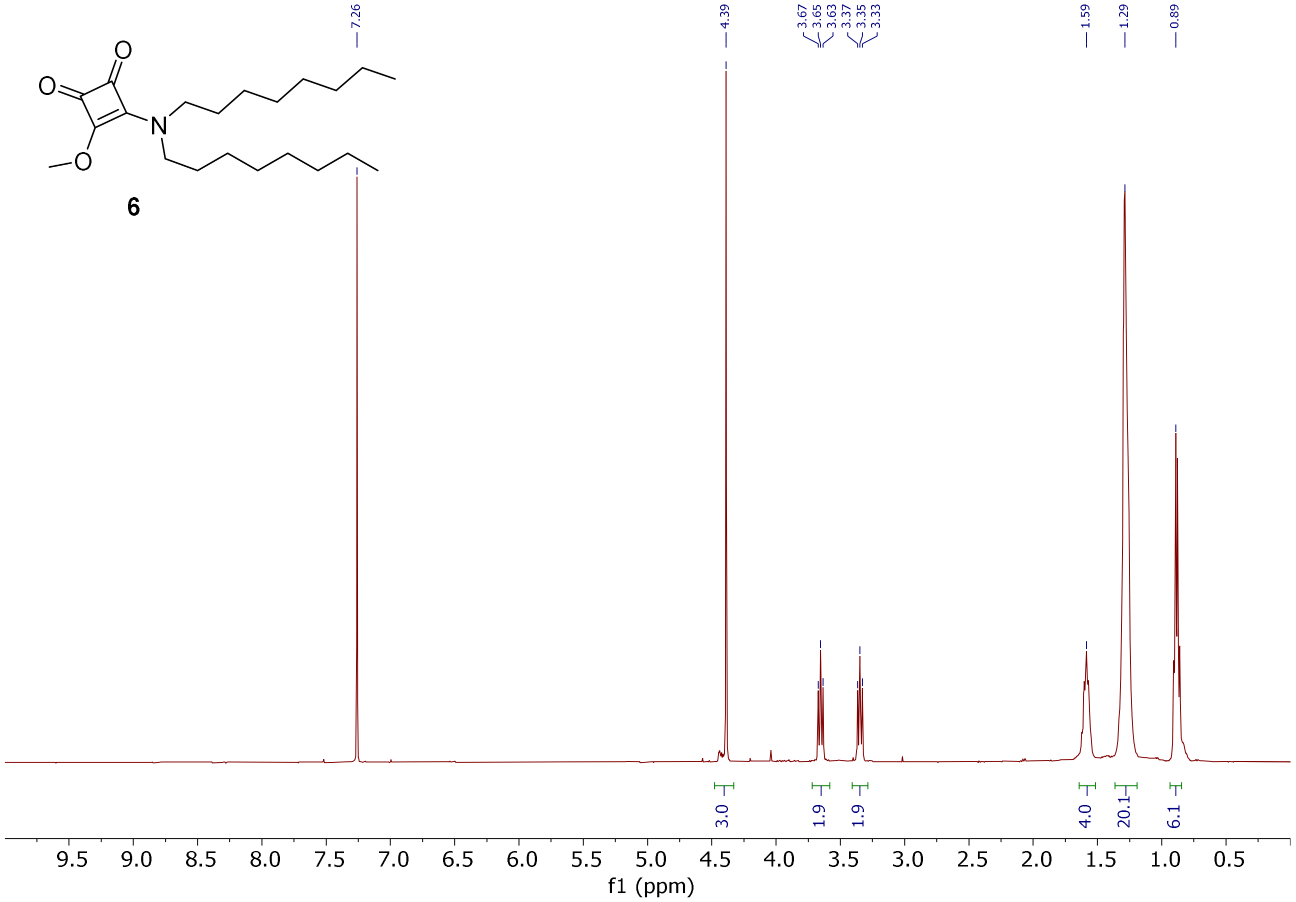

^{13}C NMR:

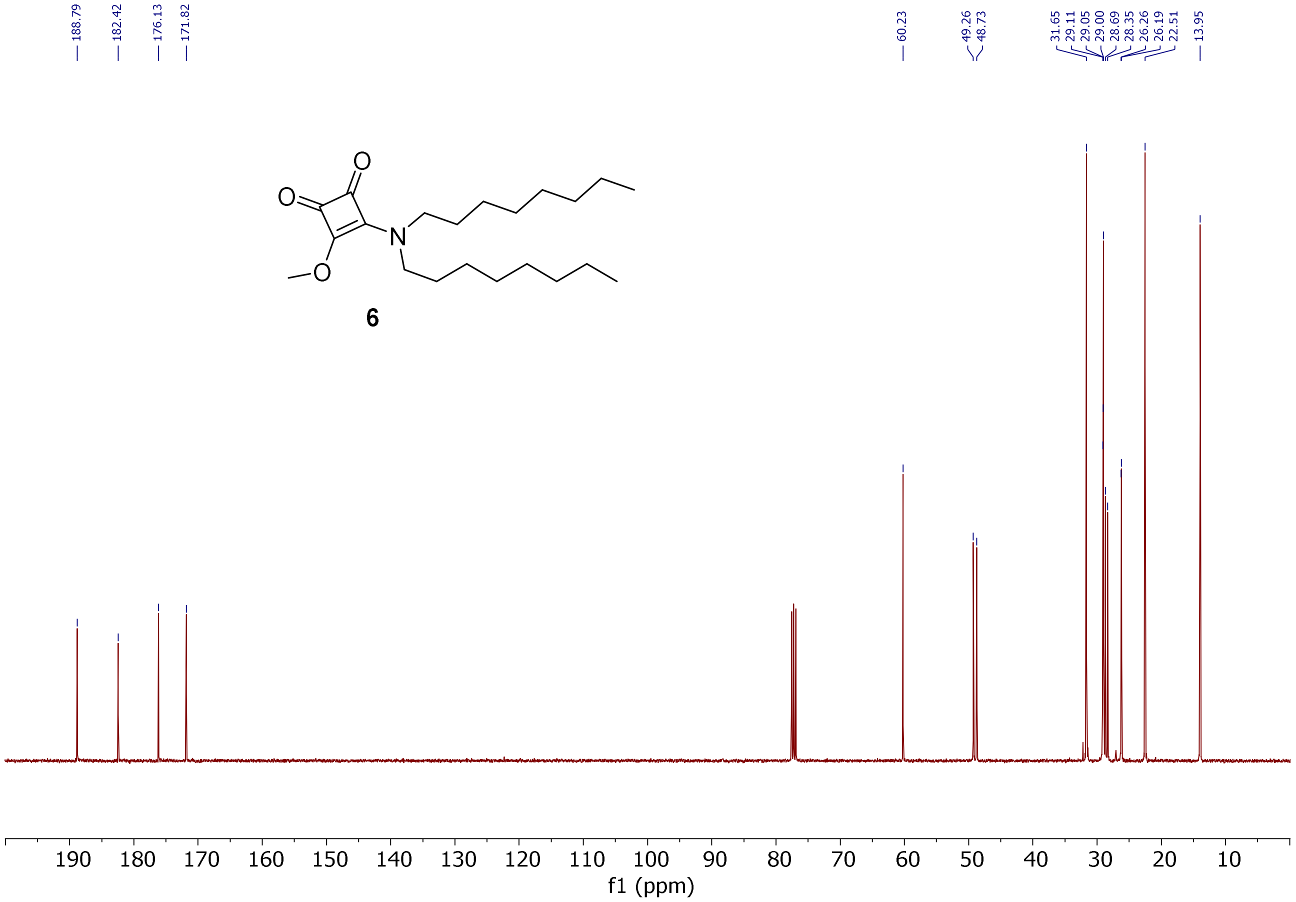

3-(Di(n-octyl)amino)-4-ethoxycyclobut-3-ene-1,2-dione (7)

^{1}H NMR:

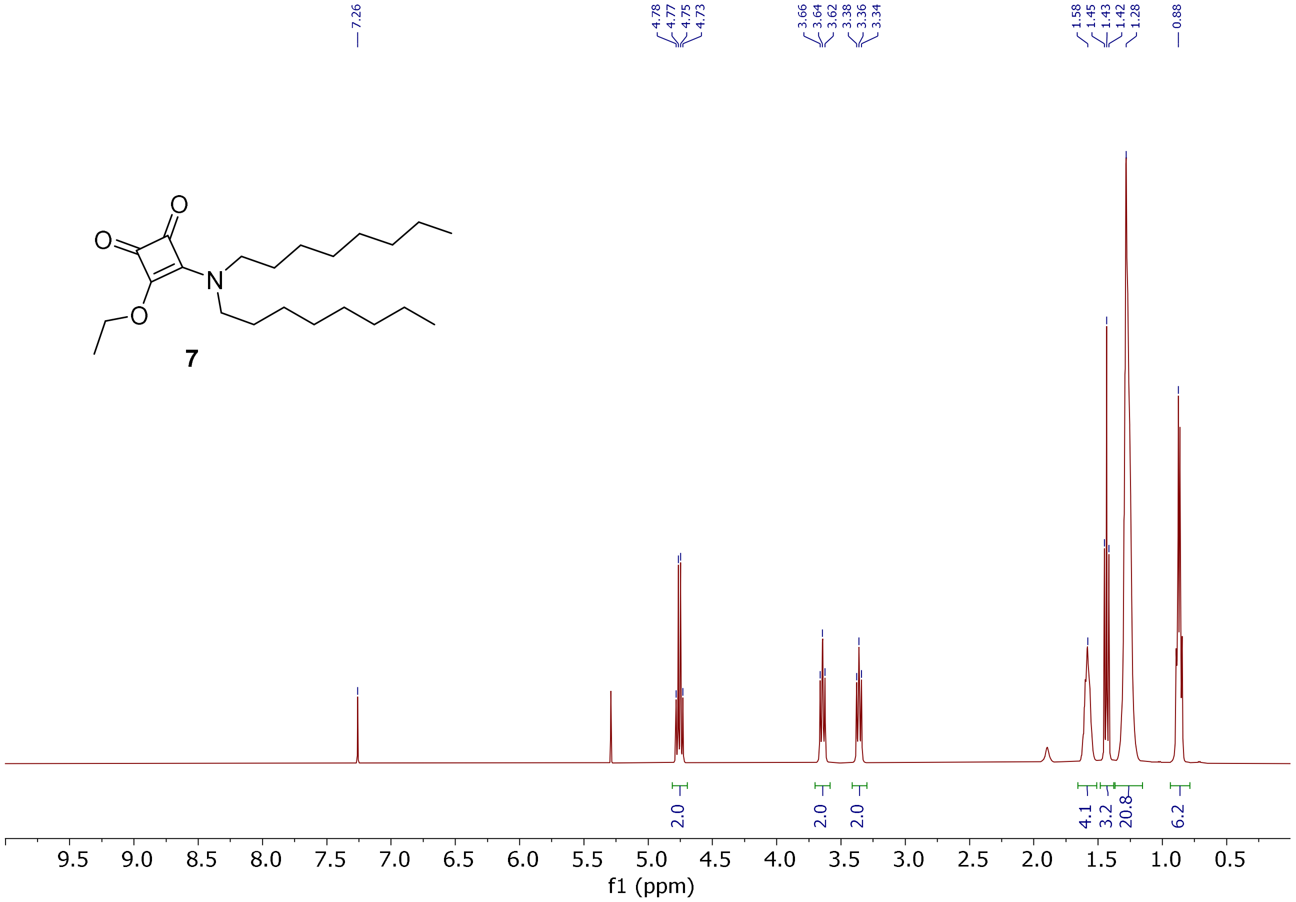

^{13}C NMR:

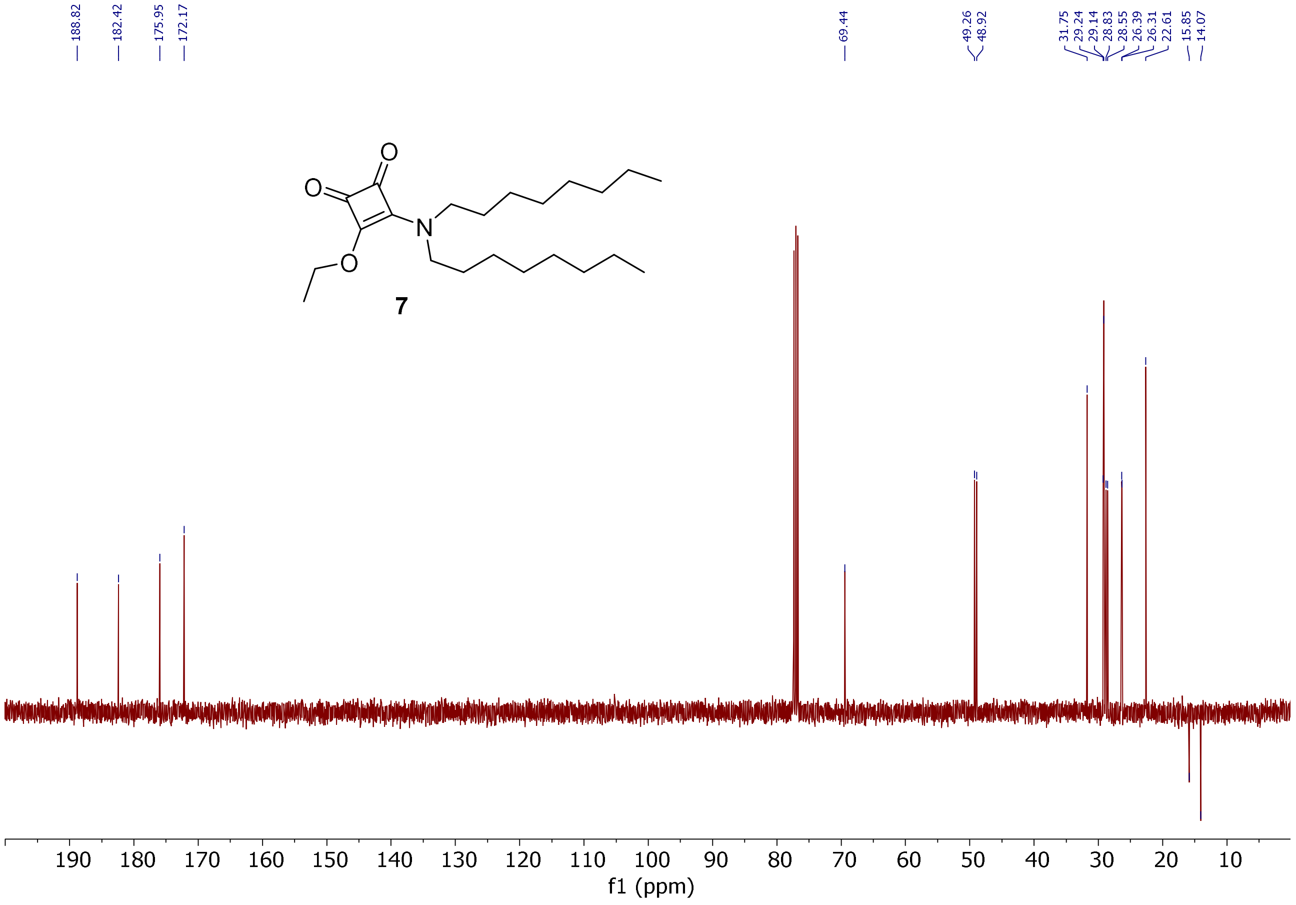

3-(Diethylamino)-4-ethoxycyclobut-3-ene-1,2-dione (8)[S3]

^{1}H NMR:

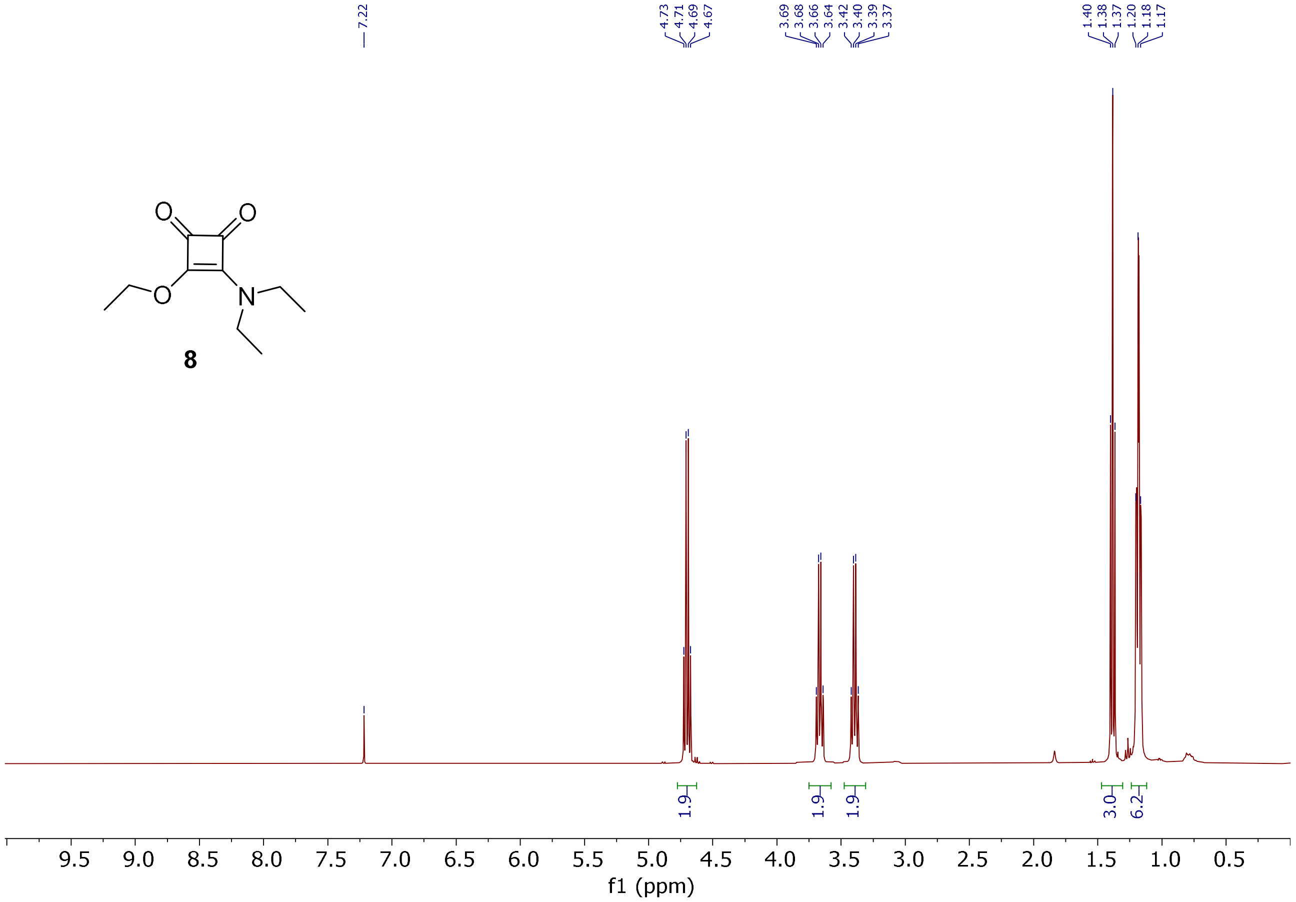

^{13}C NMR:

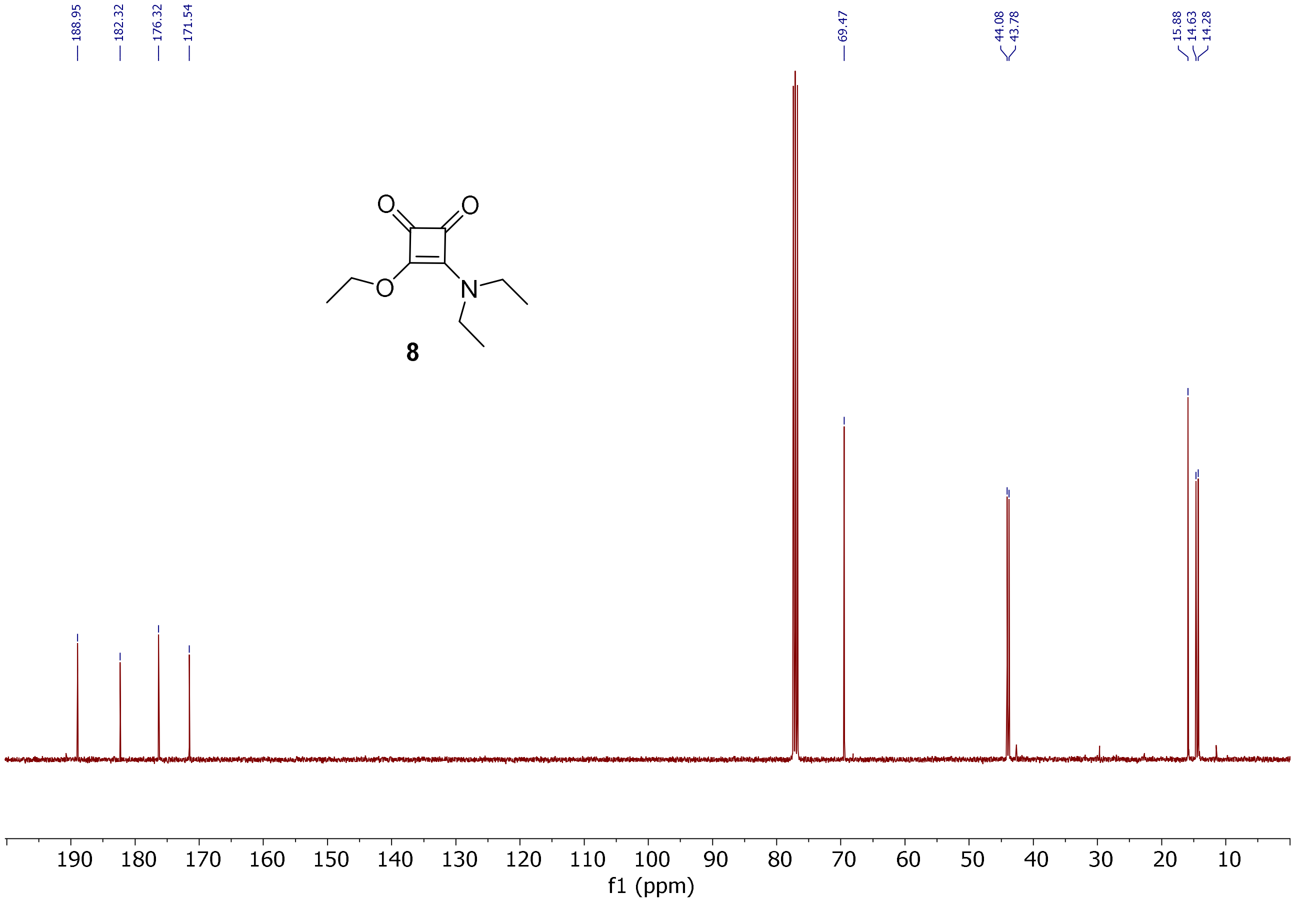

3-((3-(Dimethylamino)propyl)(methyl)amino)-4-ethoxycyclobut-3-ene-1,2-dione (9)[S4]

^{1}H NMR:

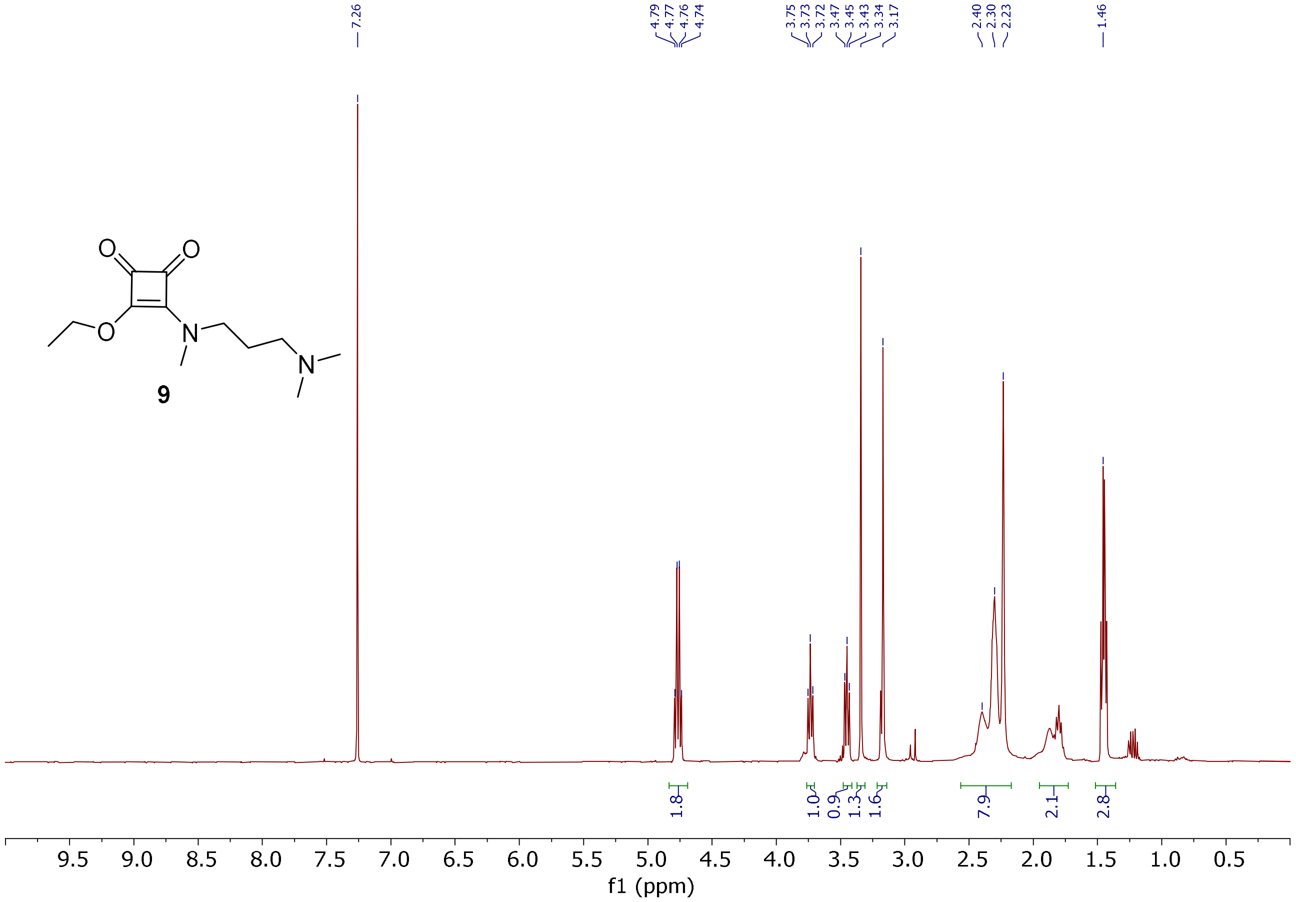

3-(Di(*n*-octyl)amino)-4-((2-(3-ethynylphenoxy)ethyl)amino)cyclobut-3-ene-1,2-dione (RD1)

^{1}H NMR:

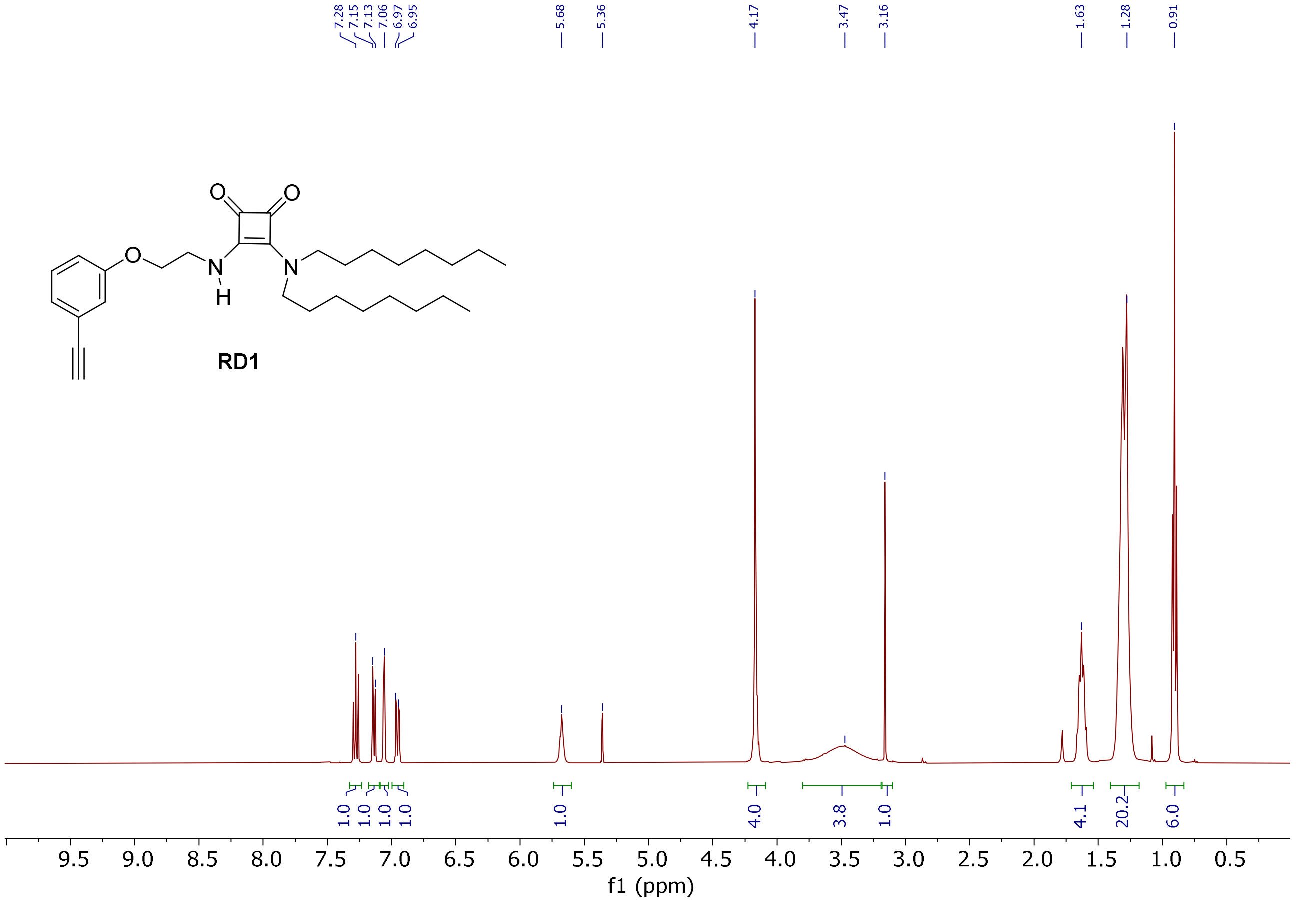

^{13}C NMR:

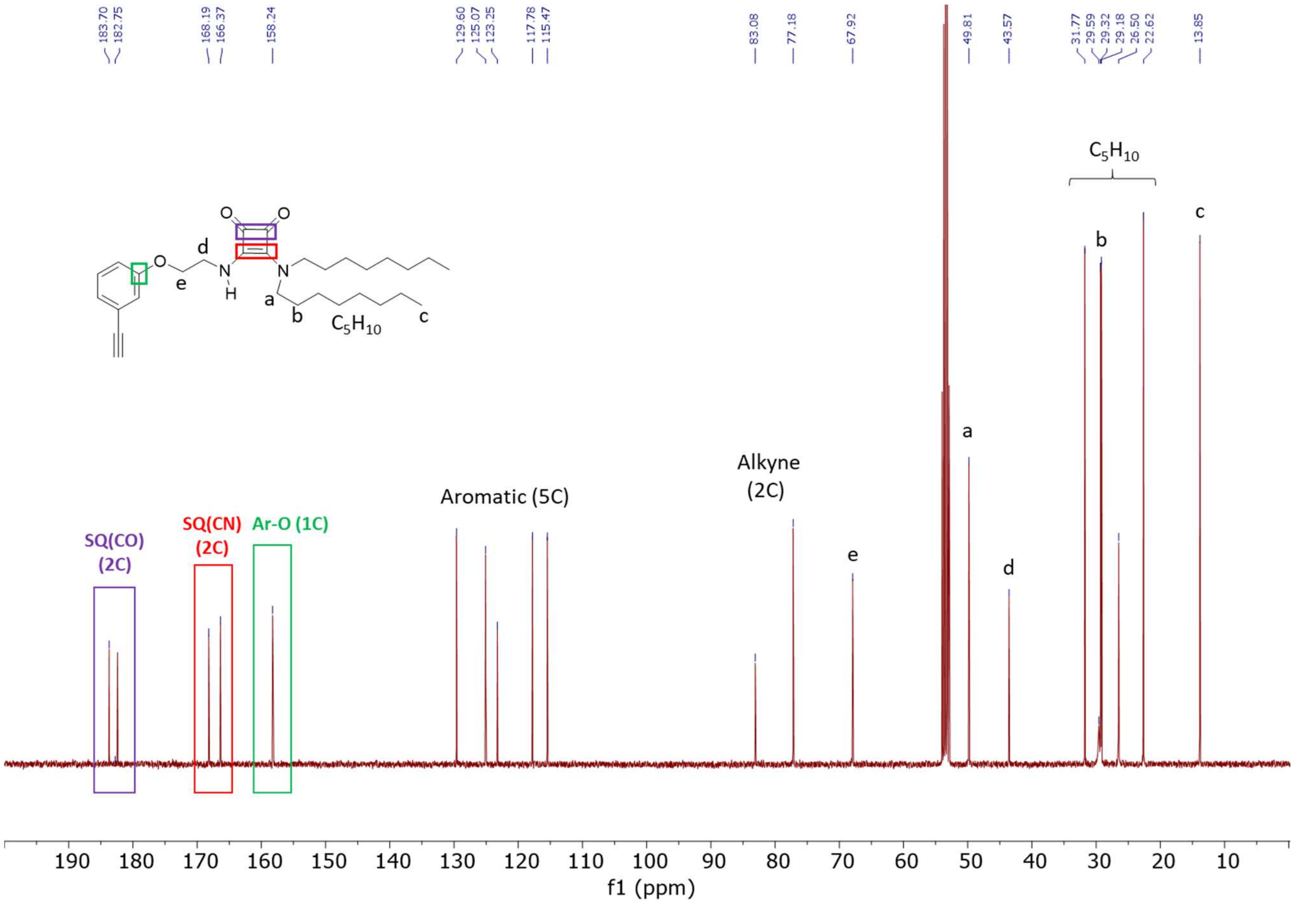

2D NMR:

(COSY)

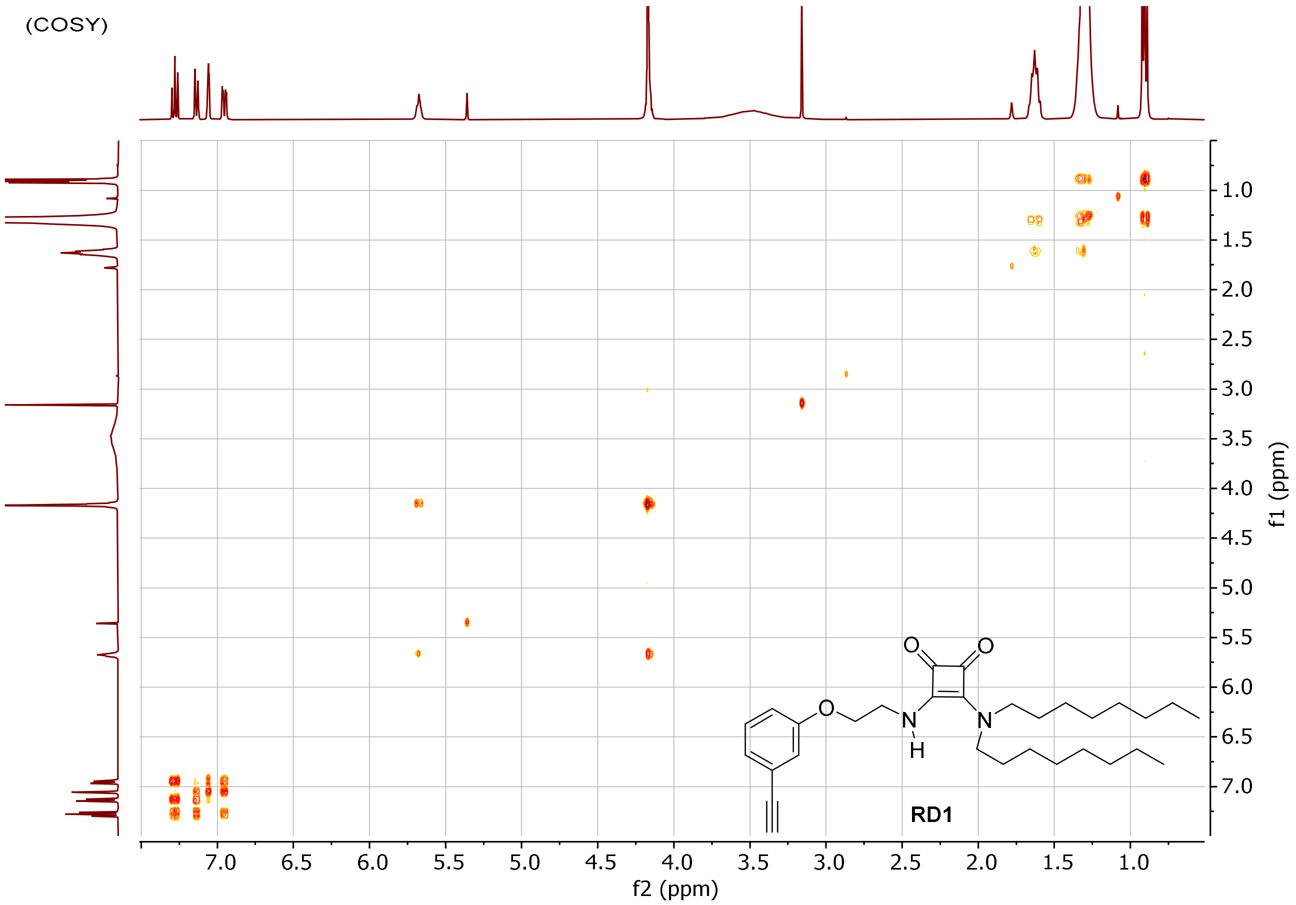

(HSQC)

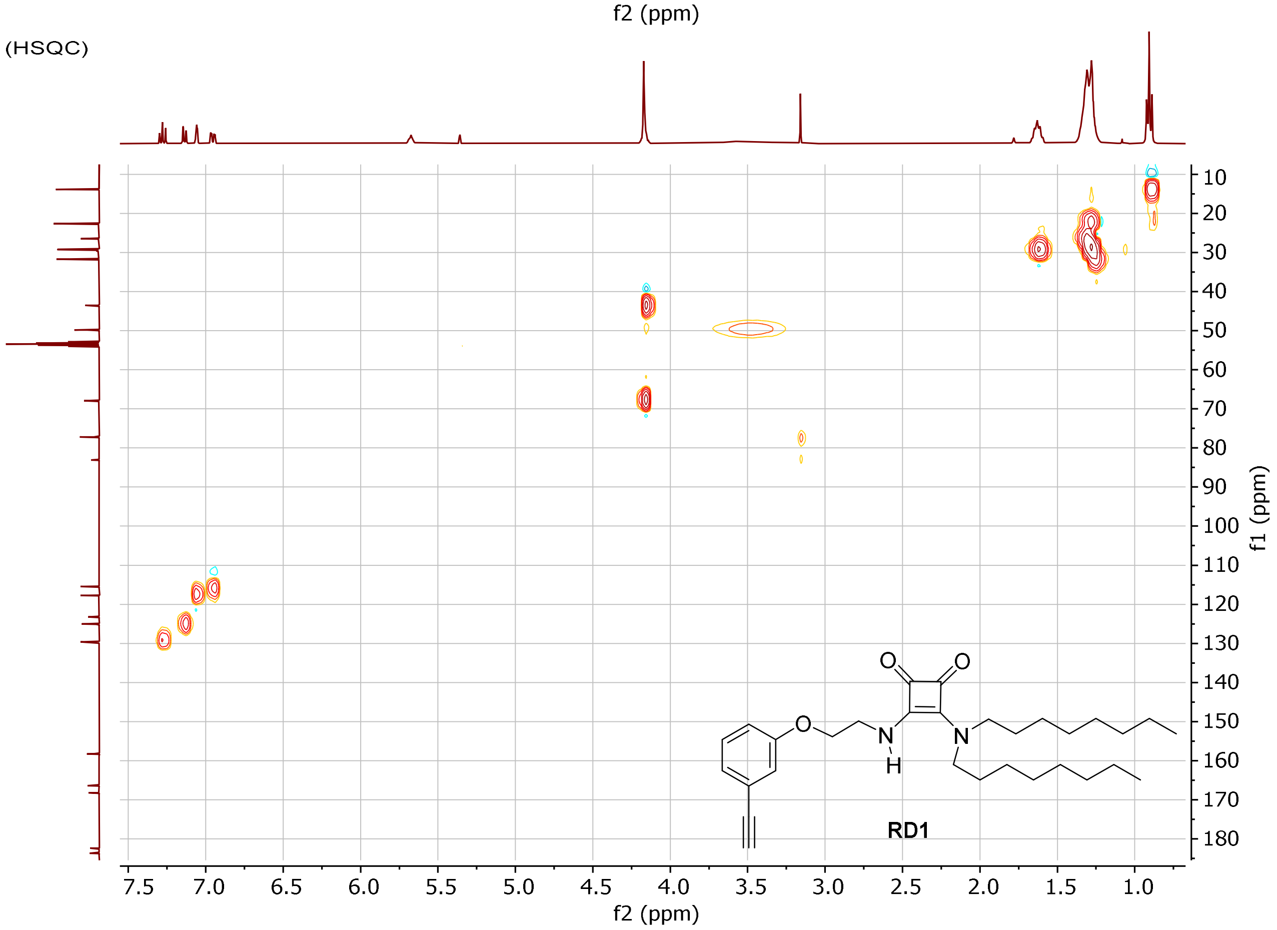

(HMBC)

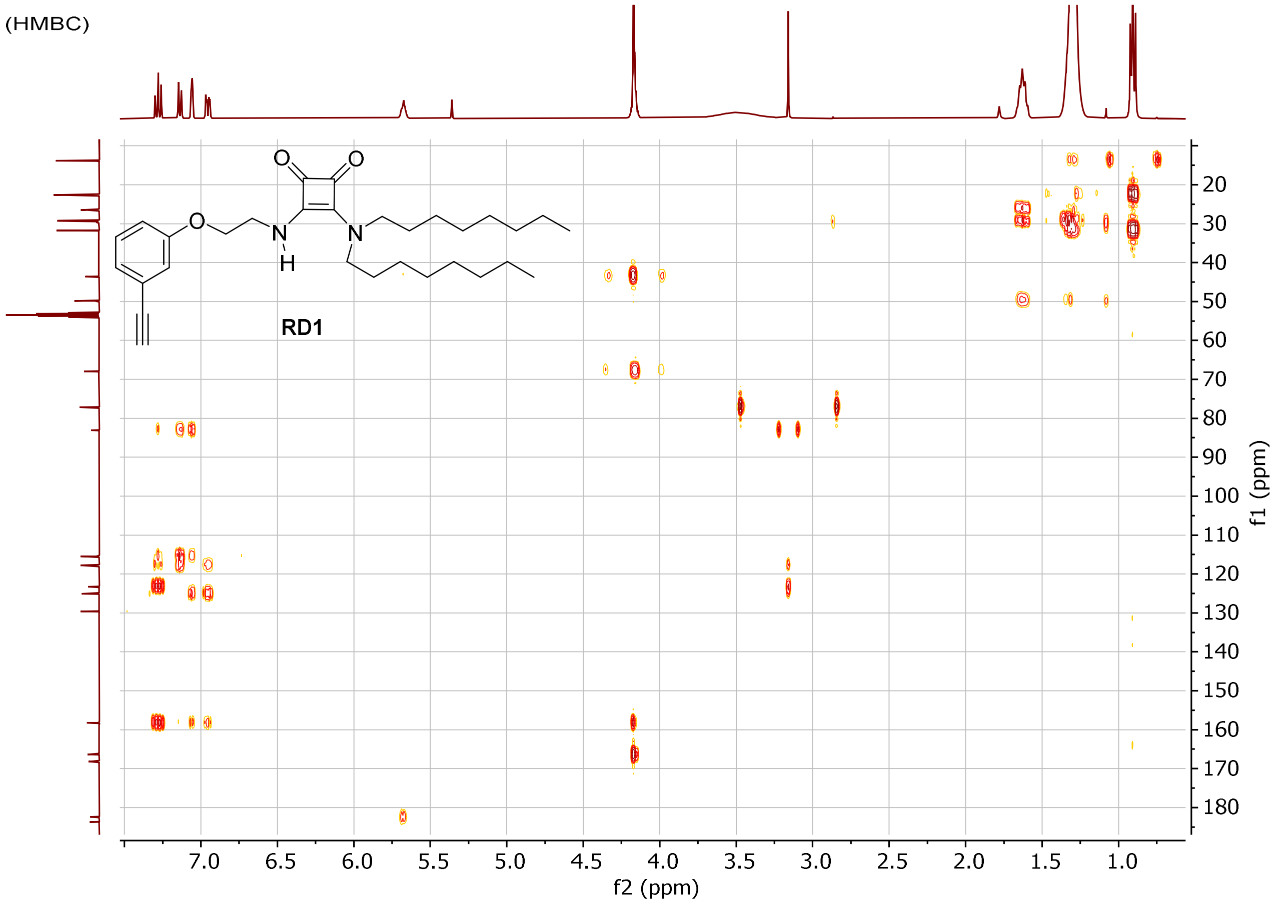

RD1
7.0
6.5
6.0
5.5
5.0
4.5
4.0
3.5
3.0
2.5
2.0
1.5
1.0
f2 (ppm)
20
30
40
50
60
70
80
90
100
110
120
130
140
150
160
170
180
f1 (ppm)

***tert*-Butyl (2-(2-((4-bromo-3-(2-((*tert*-butoxycarbonyl)amino)ethoxy)phenyl)ethynyl)phenoxy)ethyl)-carbamate (11)**

^{1}H NMR:

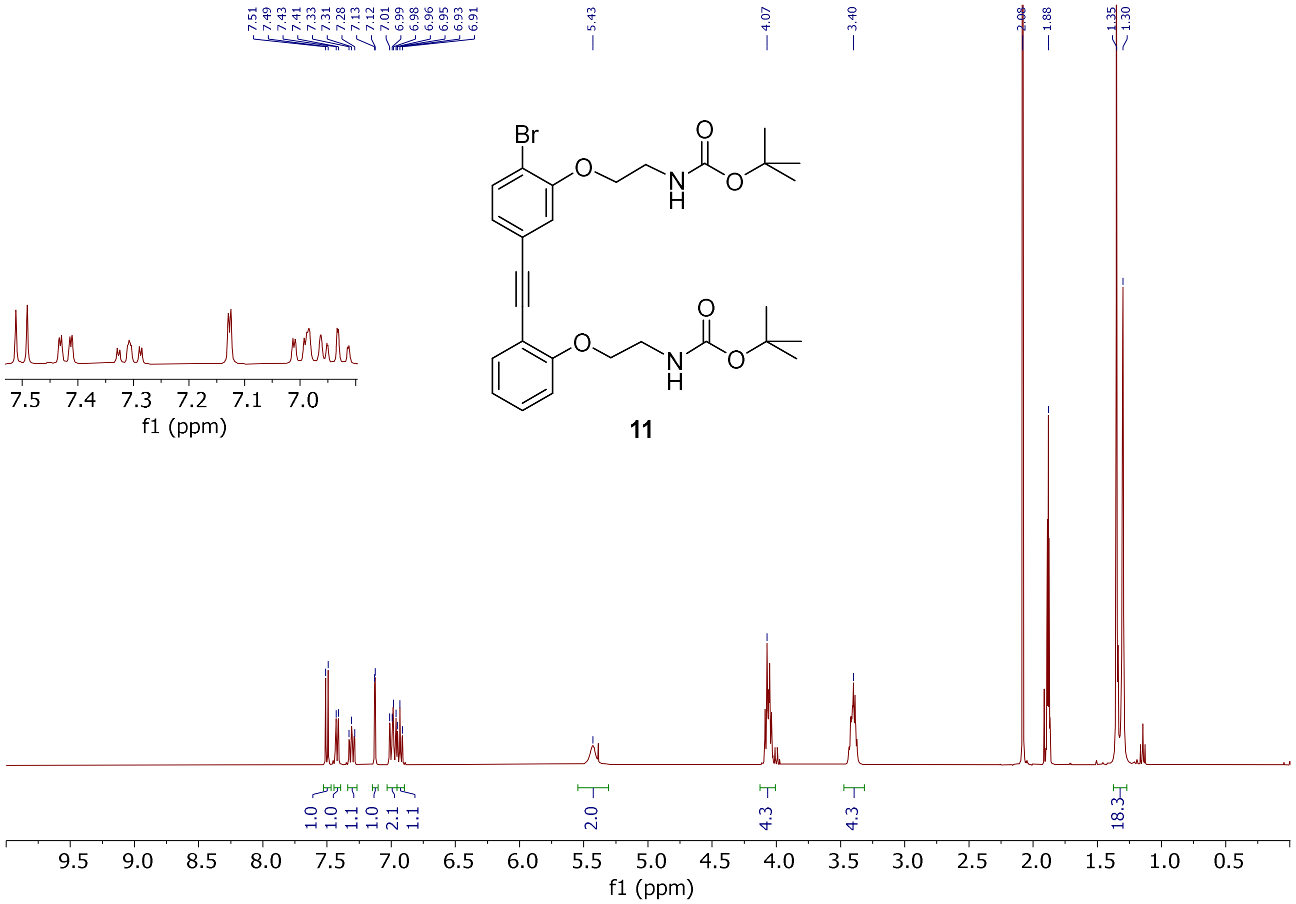

^{13}C NMR:

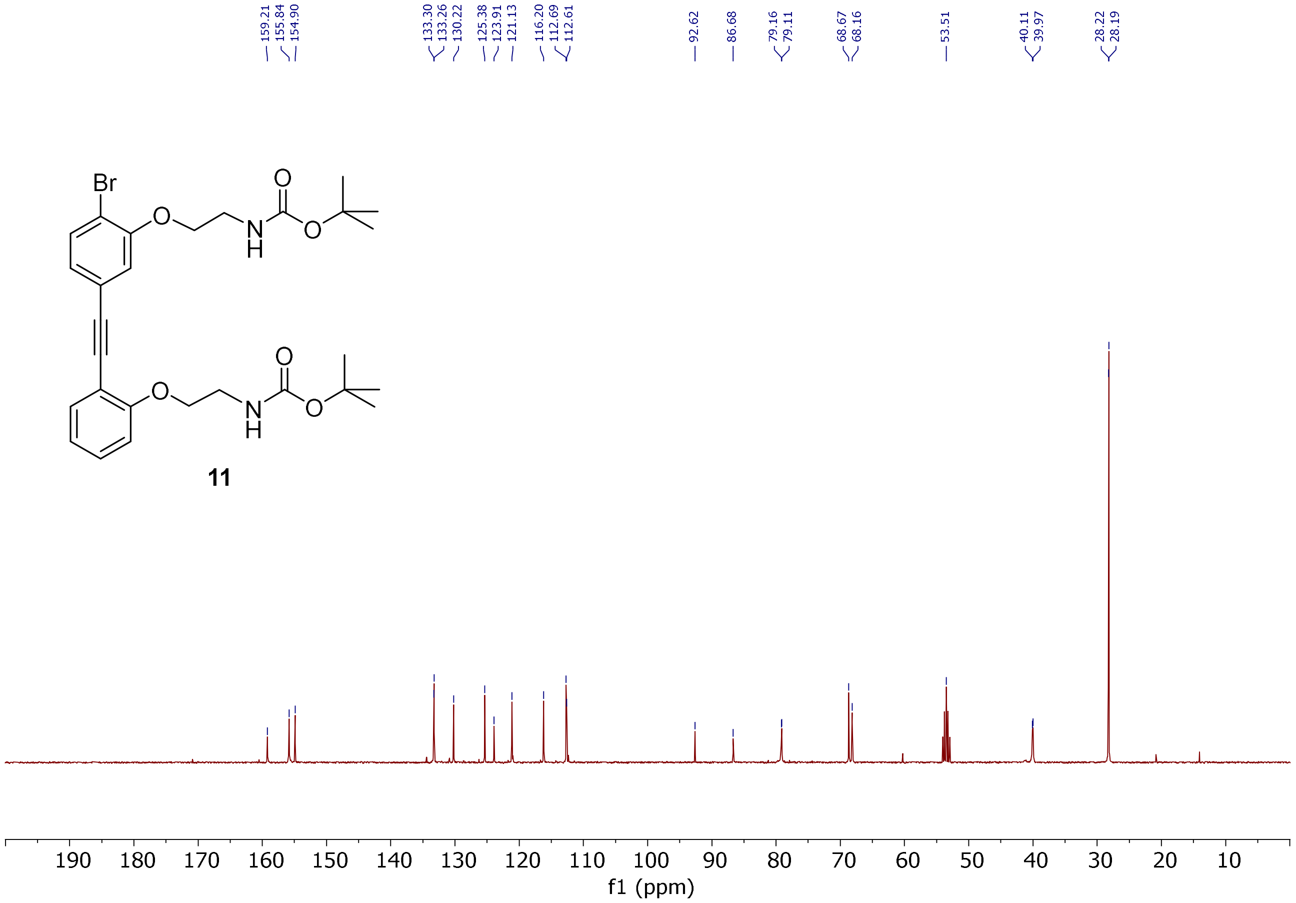

***tert*-Butyl (2-(2-((3-(2-((*tert*-butoxycarbonyl)amino)ethoxy)-4-((trimethylsilyl)ethynyl)phenyl)ethynyl)-phenoxy)ethyl)carbamate (12)**

^{1}H NMR:

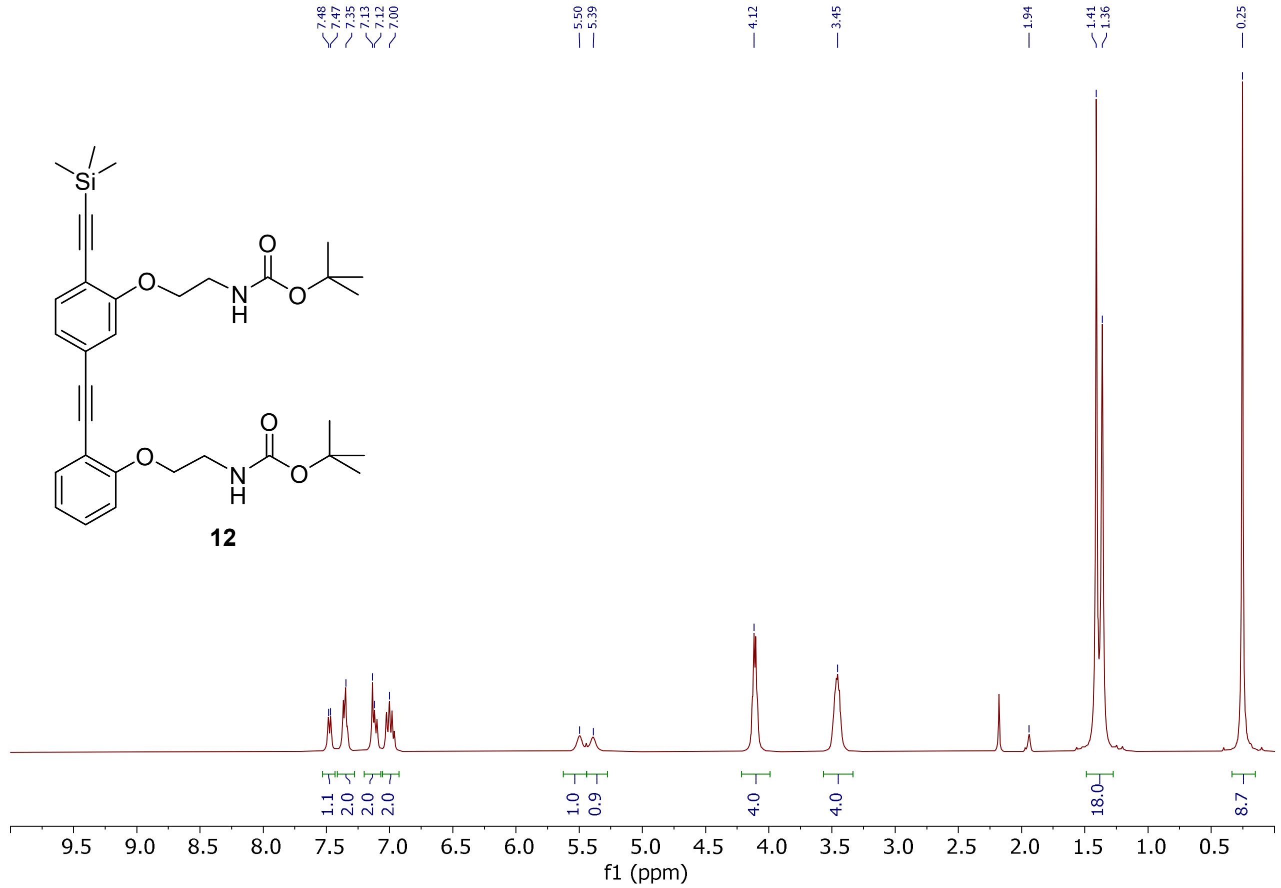

^{13}C NMR:

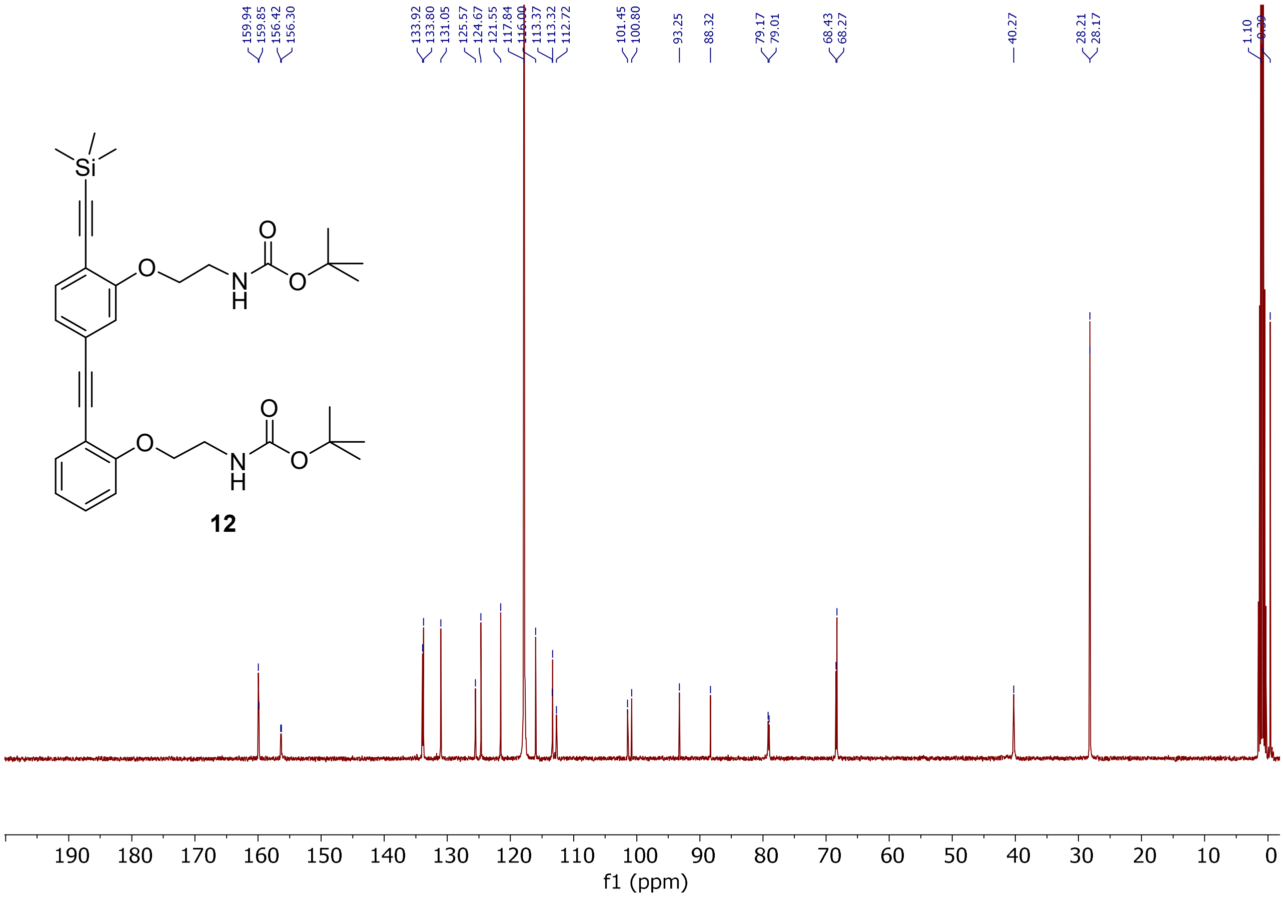

3-(Diethylamino)-4-((2-(3-iodophenoxy)ethyl)amino)cyclobut-3-ene-1,2-dione (13)

^{1}H NMR:

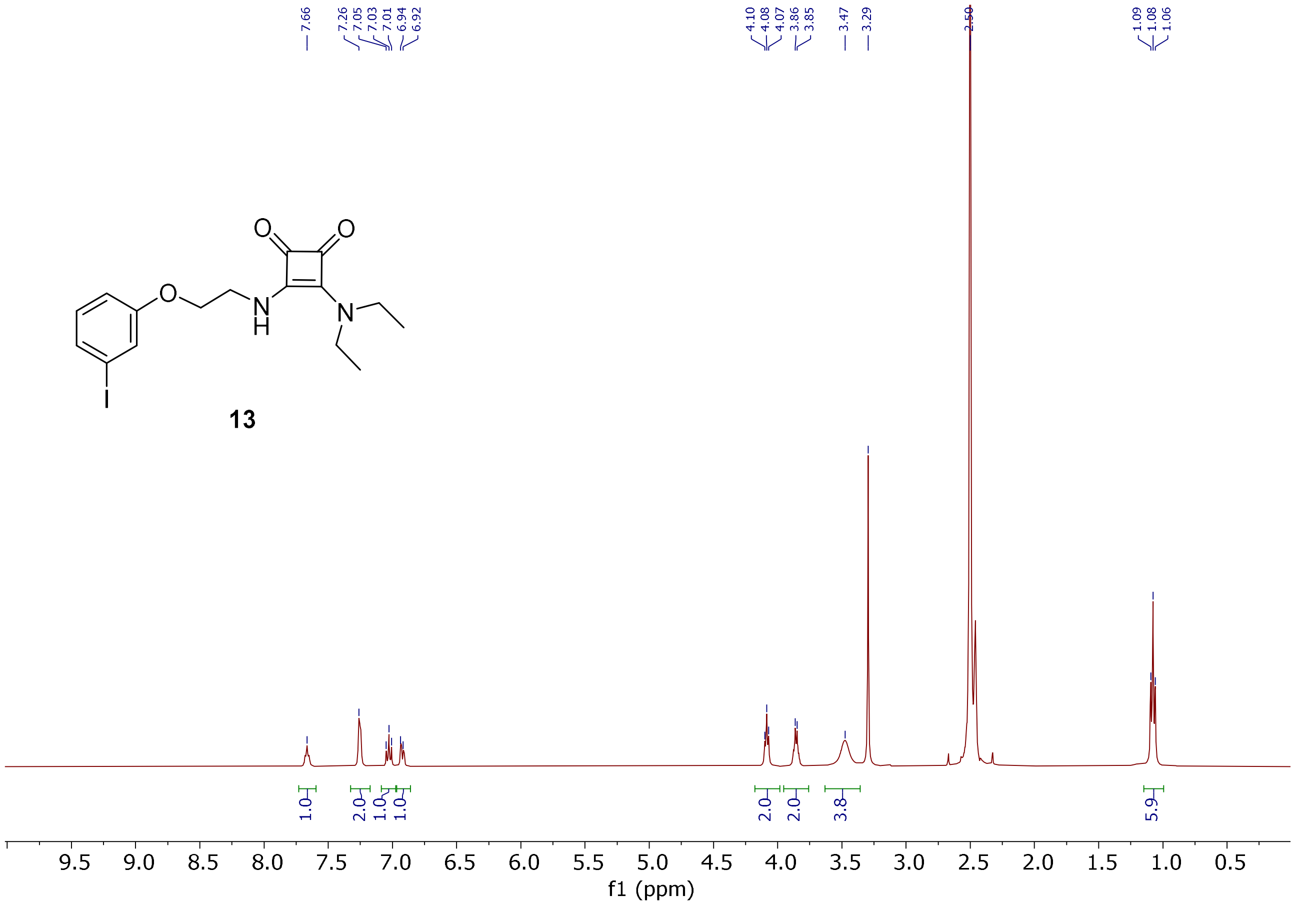

^{13}C NMR:

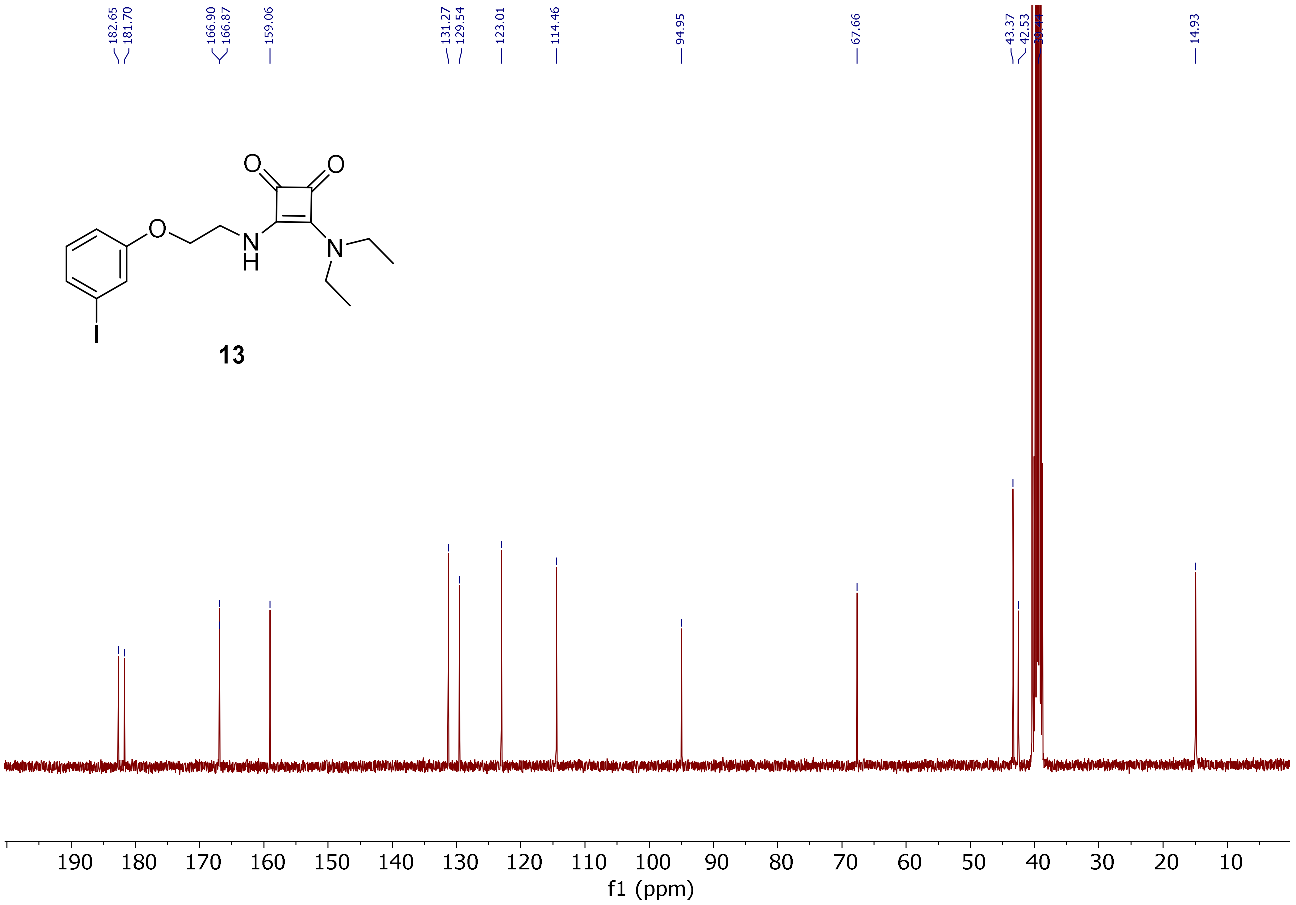

3-(Diethylamino)-4-((2-(3-iodophenoxy)ethyl)(methyl)amino)cyclobut-3-ene-1,2-dione (14)

^{1}H NMR:

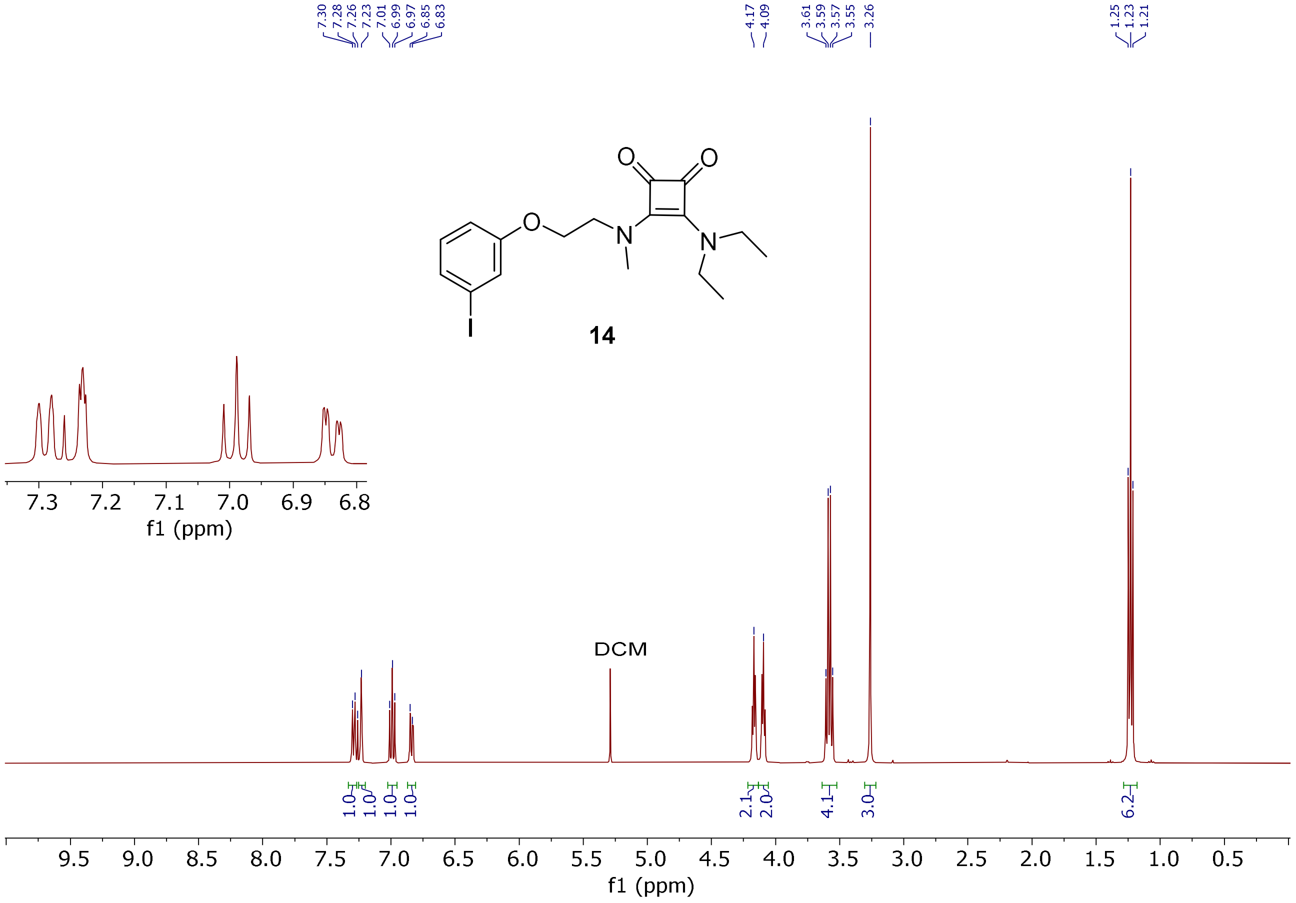

^{13}C NMR:

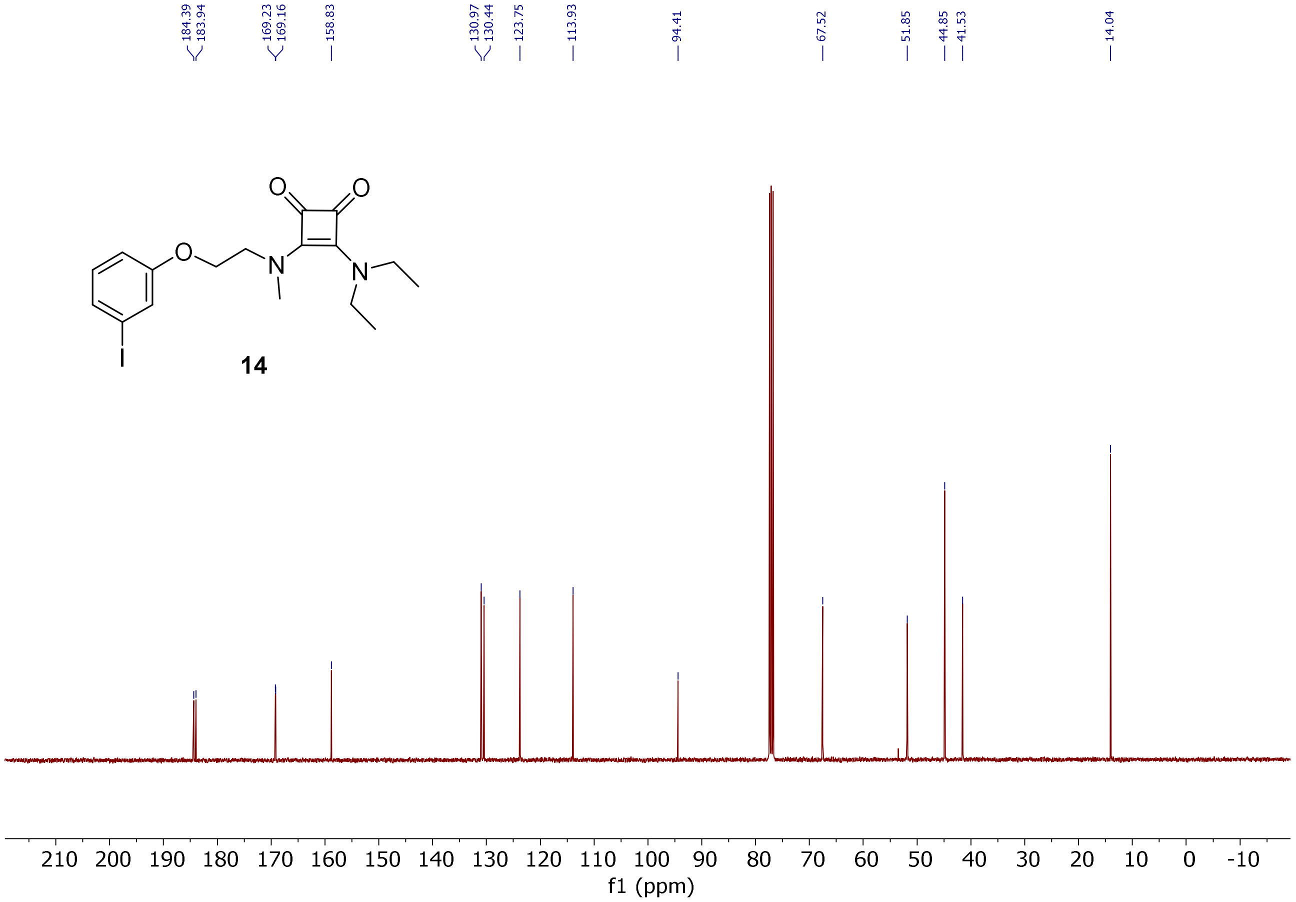

***tert*-Butyl (2-(2-((3-(2-((*tert*-butoxycarbonyl)amino)ethoxy)-4-((3-(2-((2-(diethylamino)-3,4-dioxocyclobut-1-en-1-yl)(methyl)amino)ethoxy)phenyl)ethynyl)phenyl)ethynyl)phenoxy)ethyl)carbamate (15)**

^{1}H NMR:

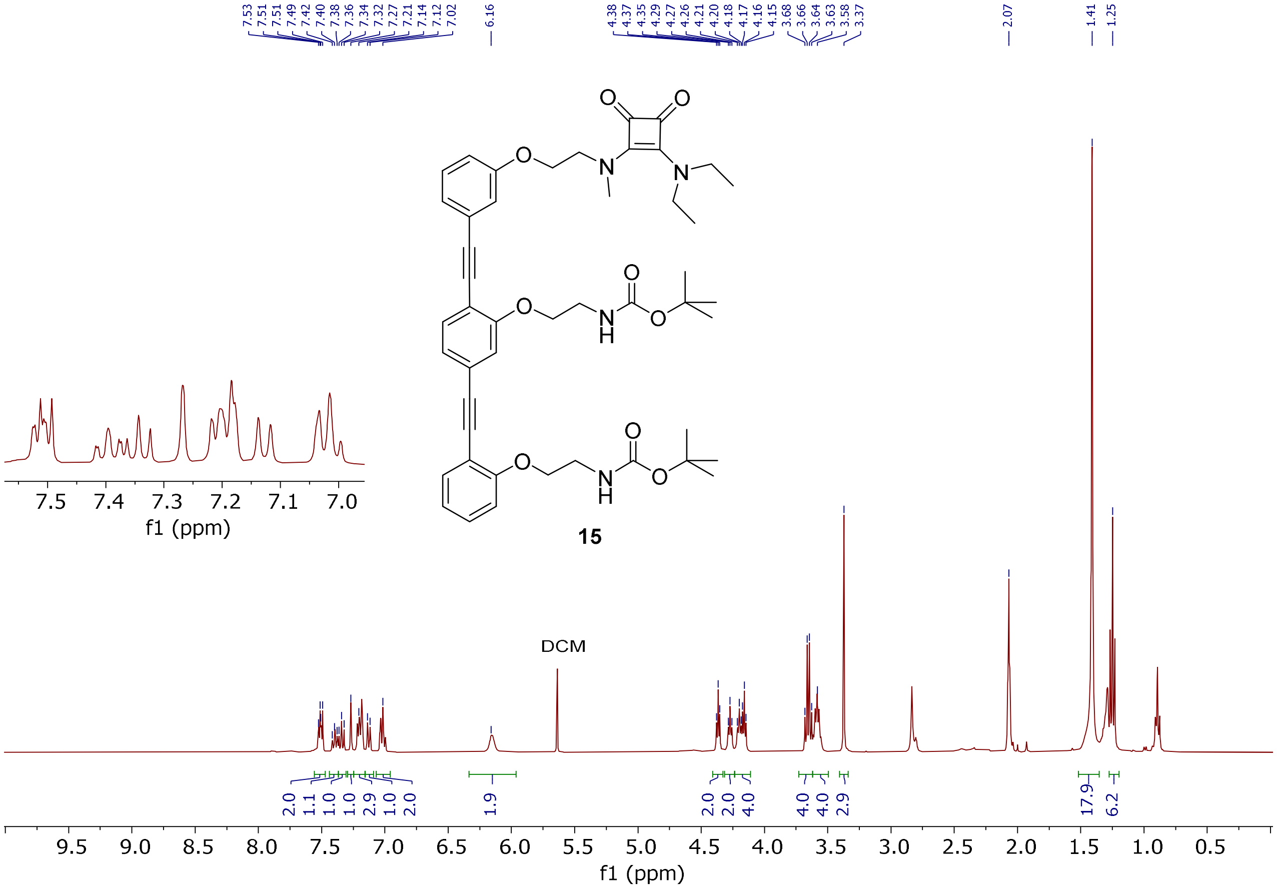

^{13}C NMR:

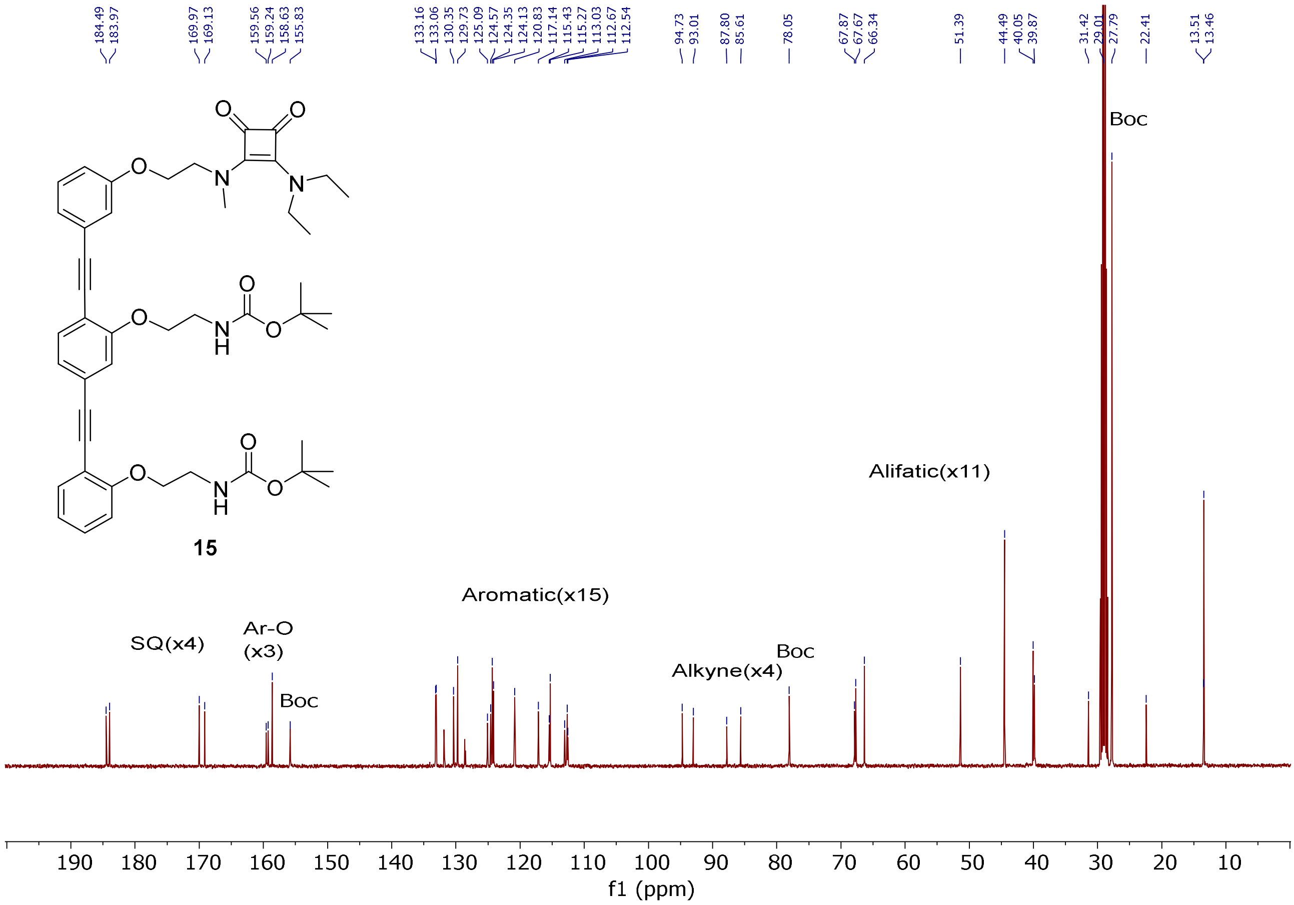

2D NMR:

(HSQC)

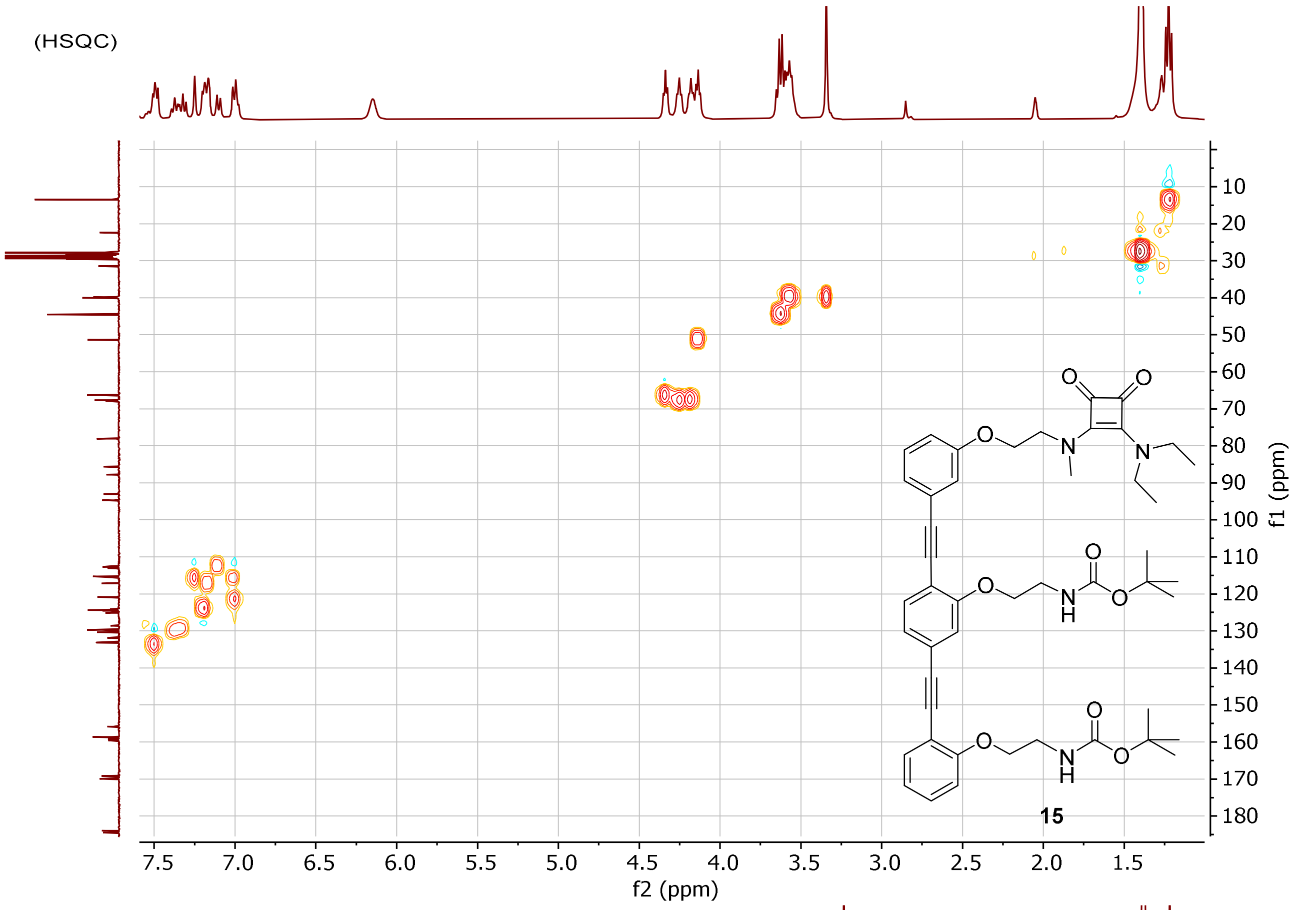

(HMBC)

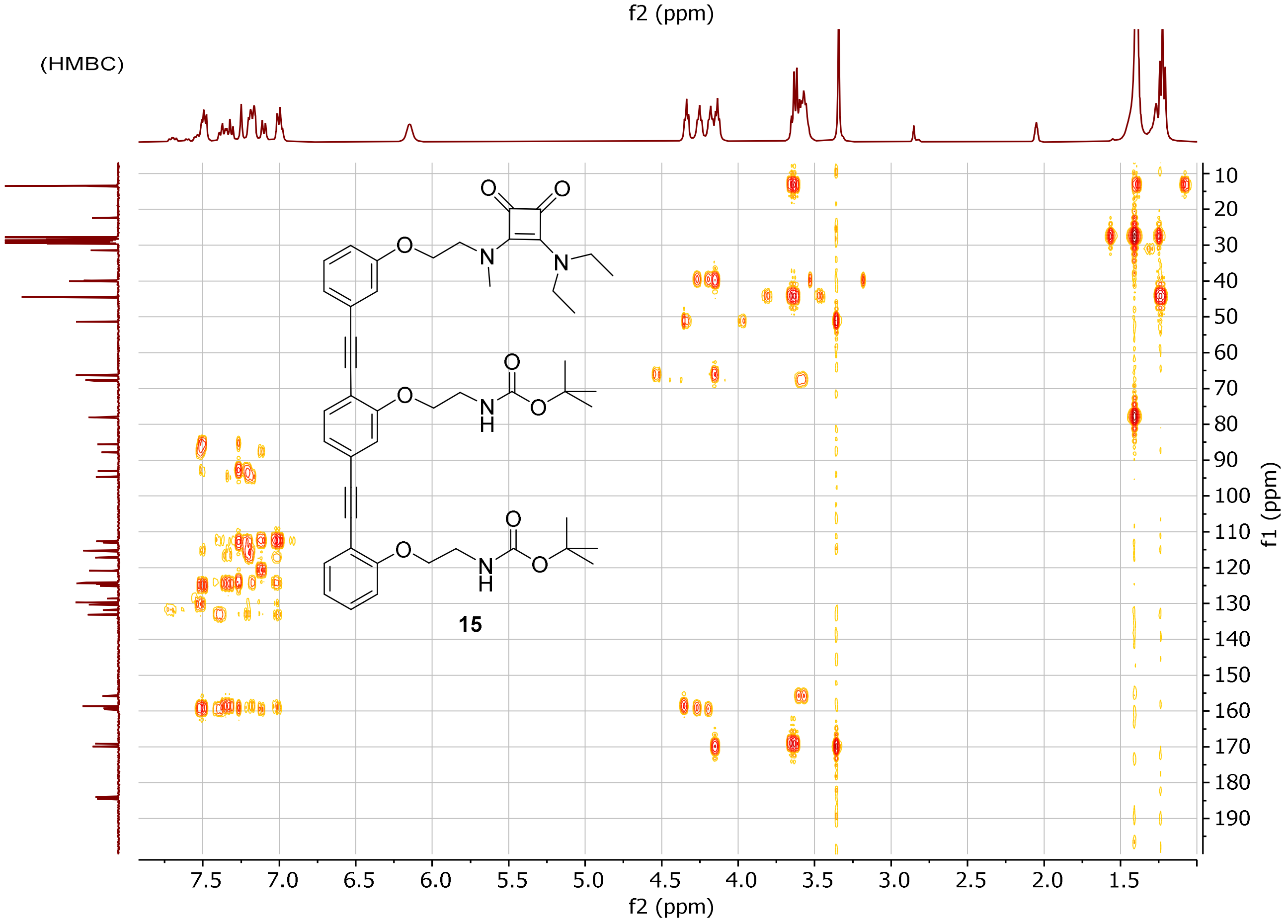

3-(Diethylamino)-4-((2-(3-((2-(2-((2-(di(*n*-octyl)amino)-3,4-dioxocyclobut-1-en-1-yl)amino)ethoxy)-4-((2-(2-((2-(di(*n*-octyl)amino)-3,4-dioxocyclobut-1-en-1-yl)amino)ethoxy)phenyl)ethynyl)phenyl)ethynyl)phenoxy)ethyl)-(methyl)amino)cyclobut-3-ene-1,2-dione (**RD2**)

^{1}H NMR:

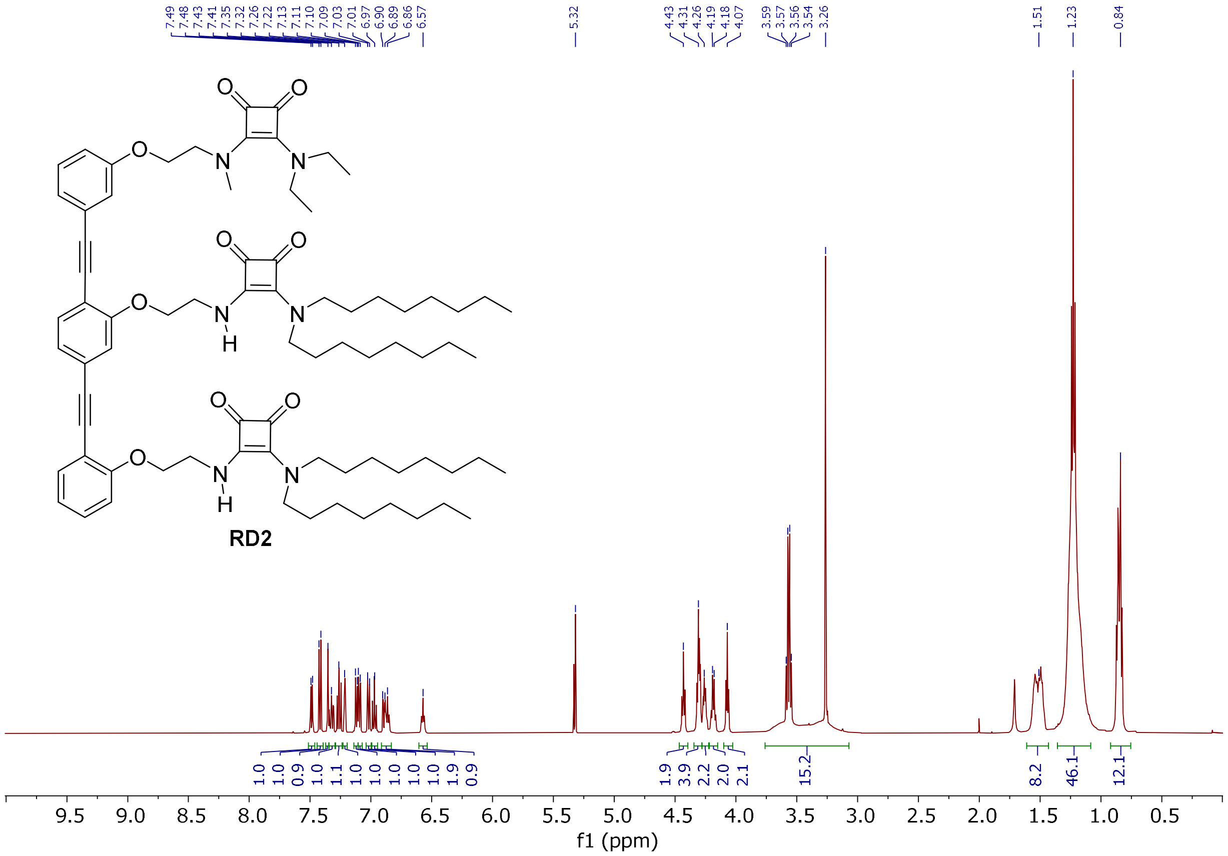

^{13}C NMR:

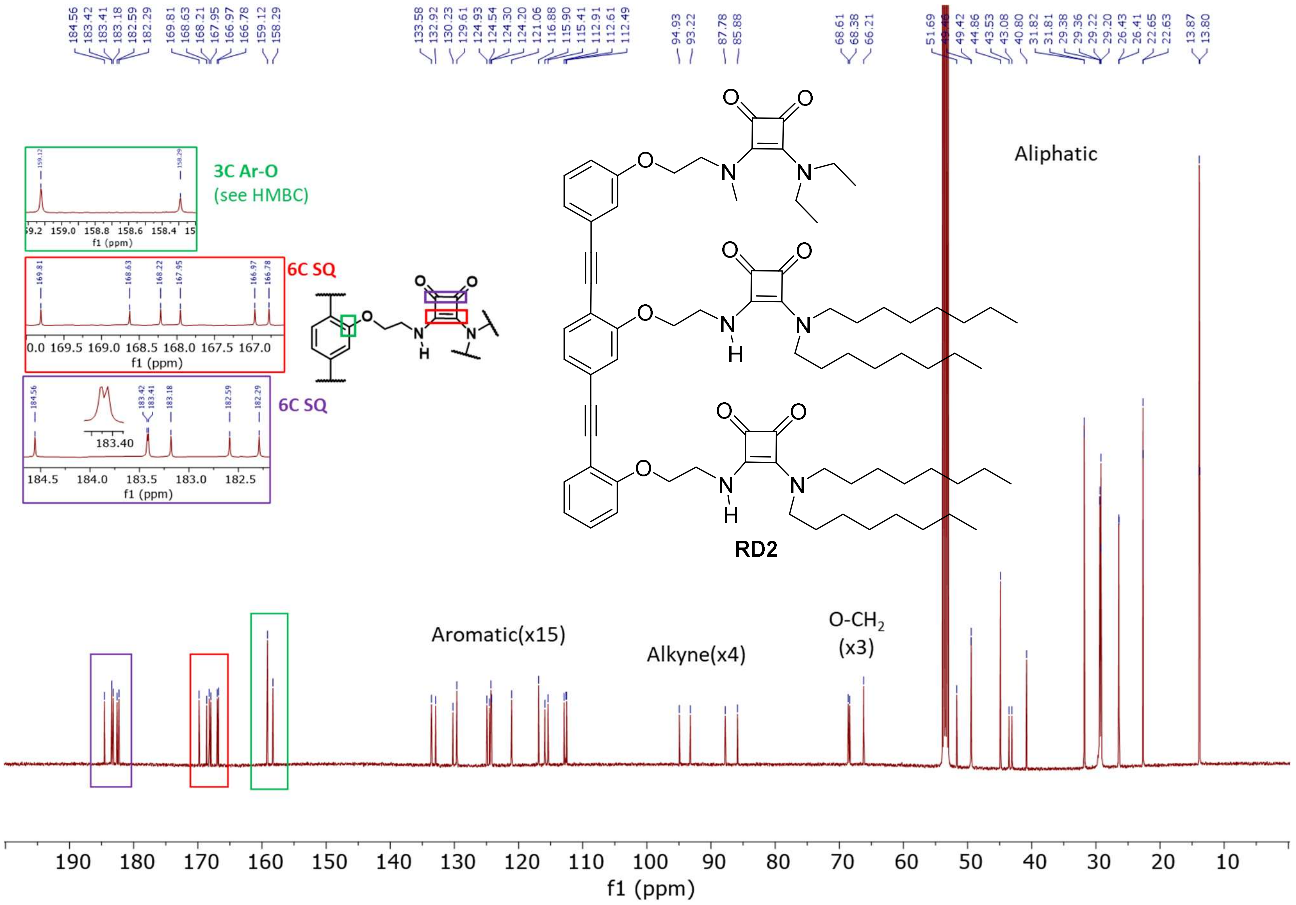

2D NMR:

(HSQC)

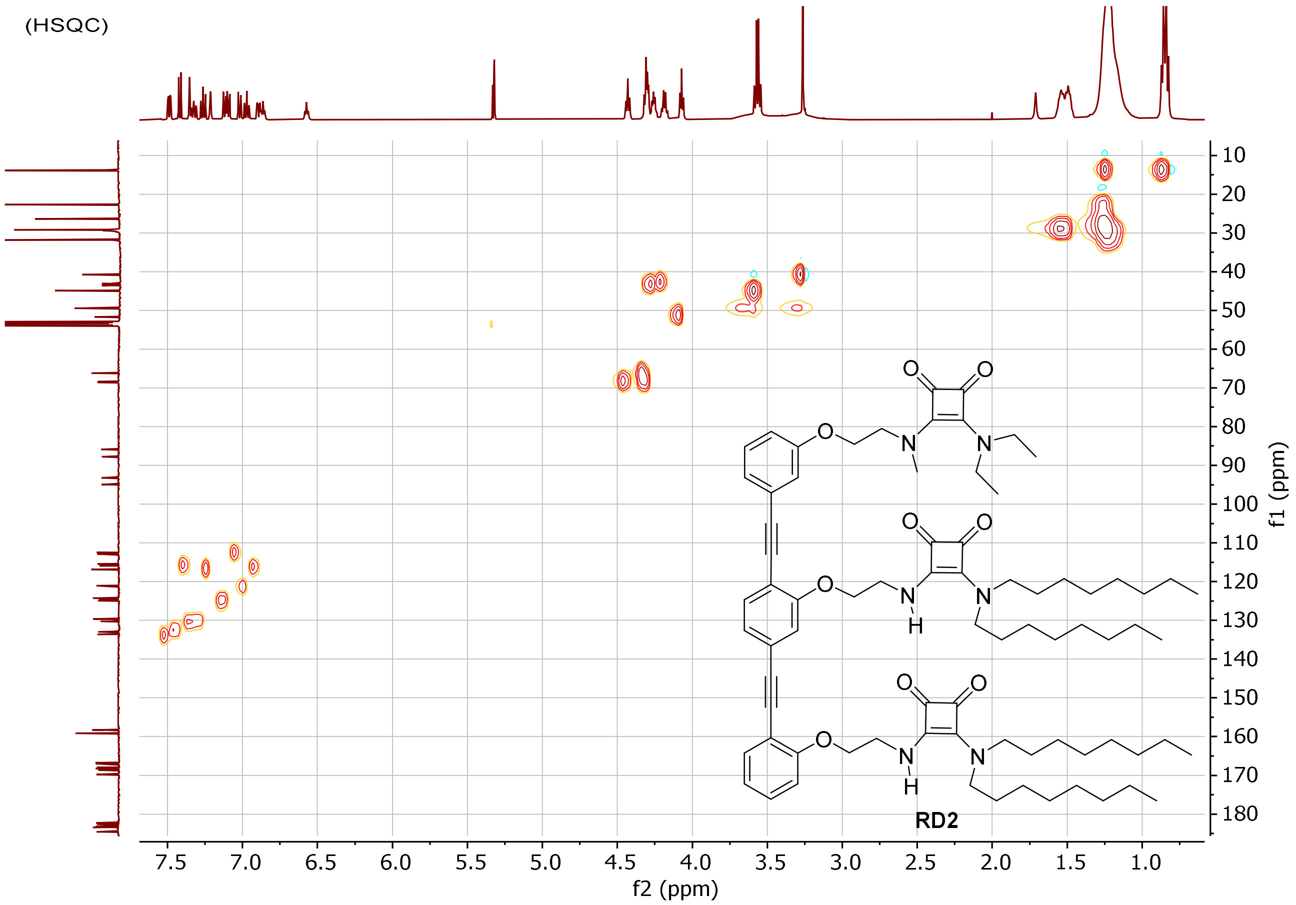

(HMBC)

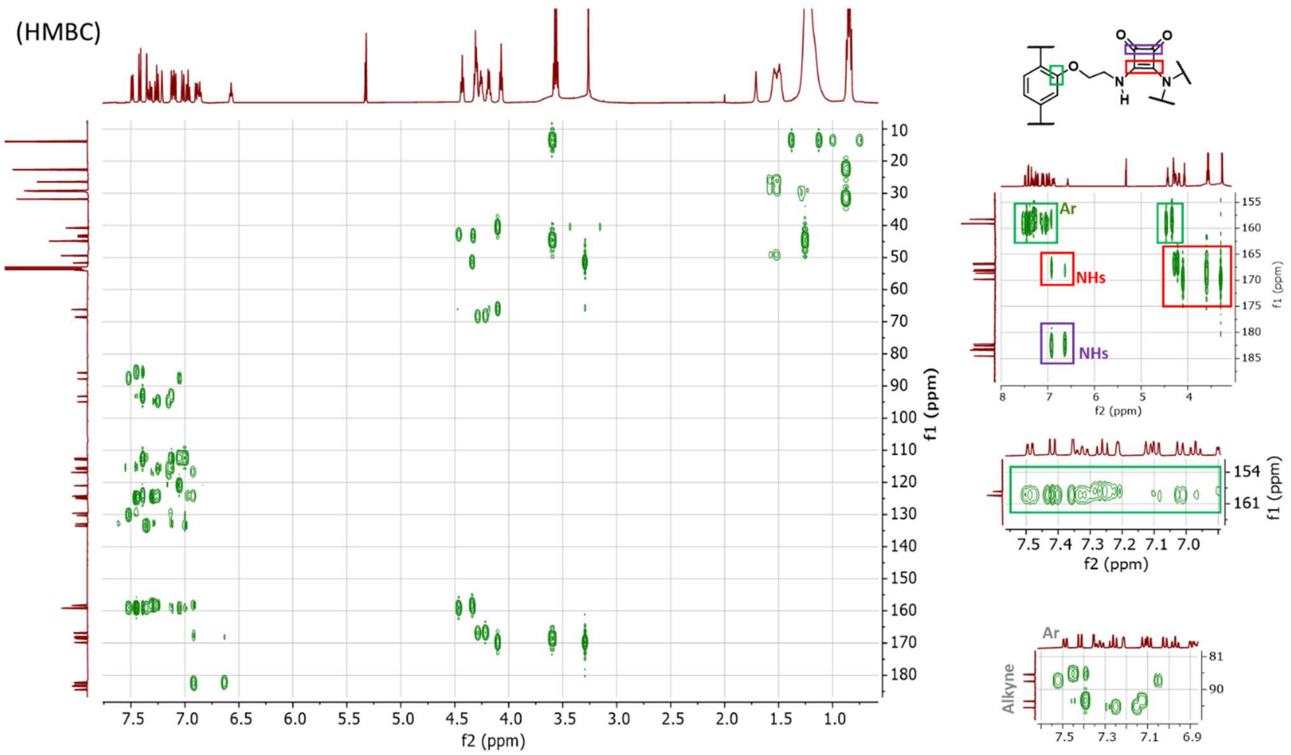

(COSY)

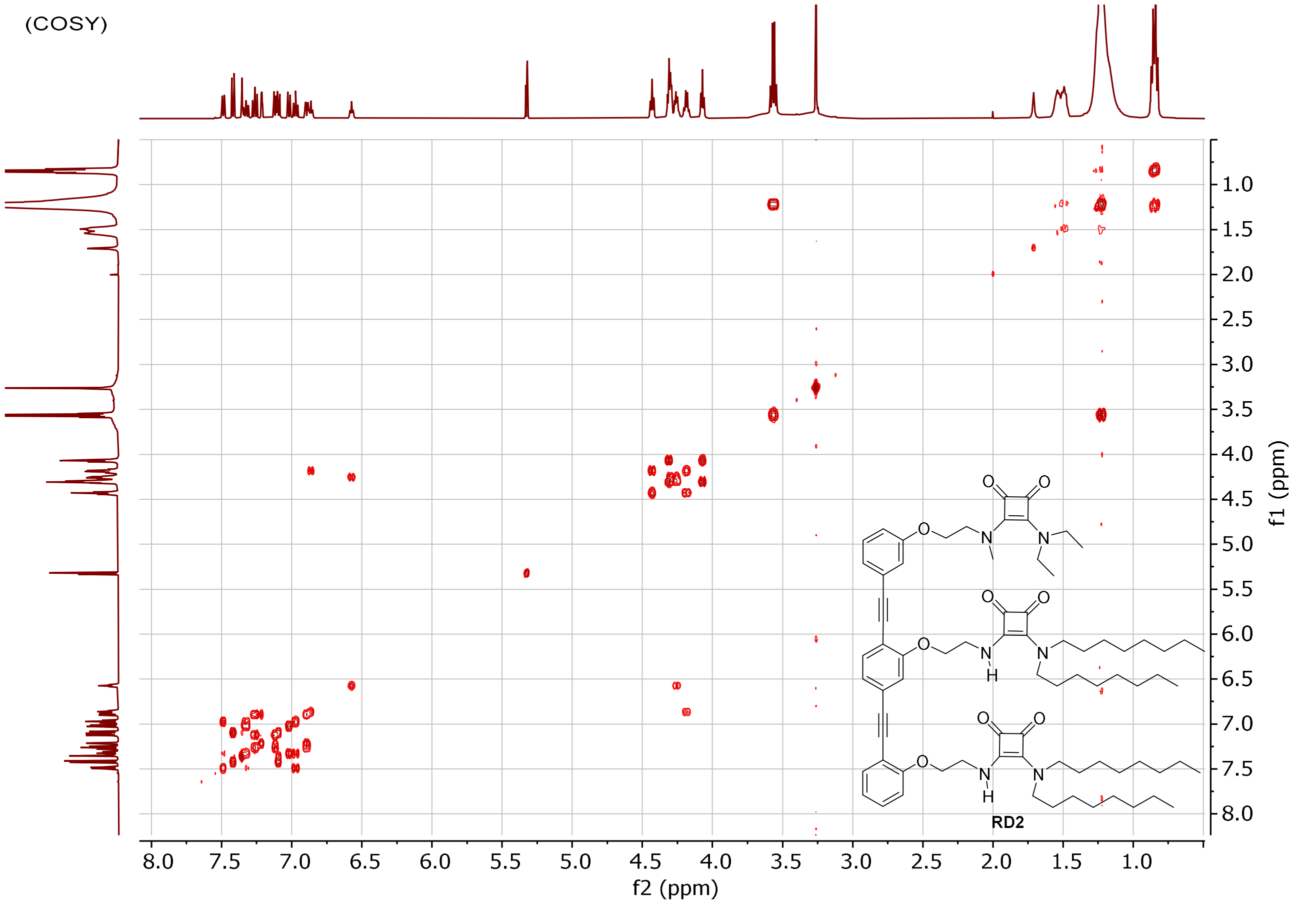

(NOESY)

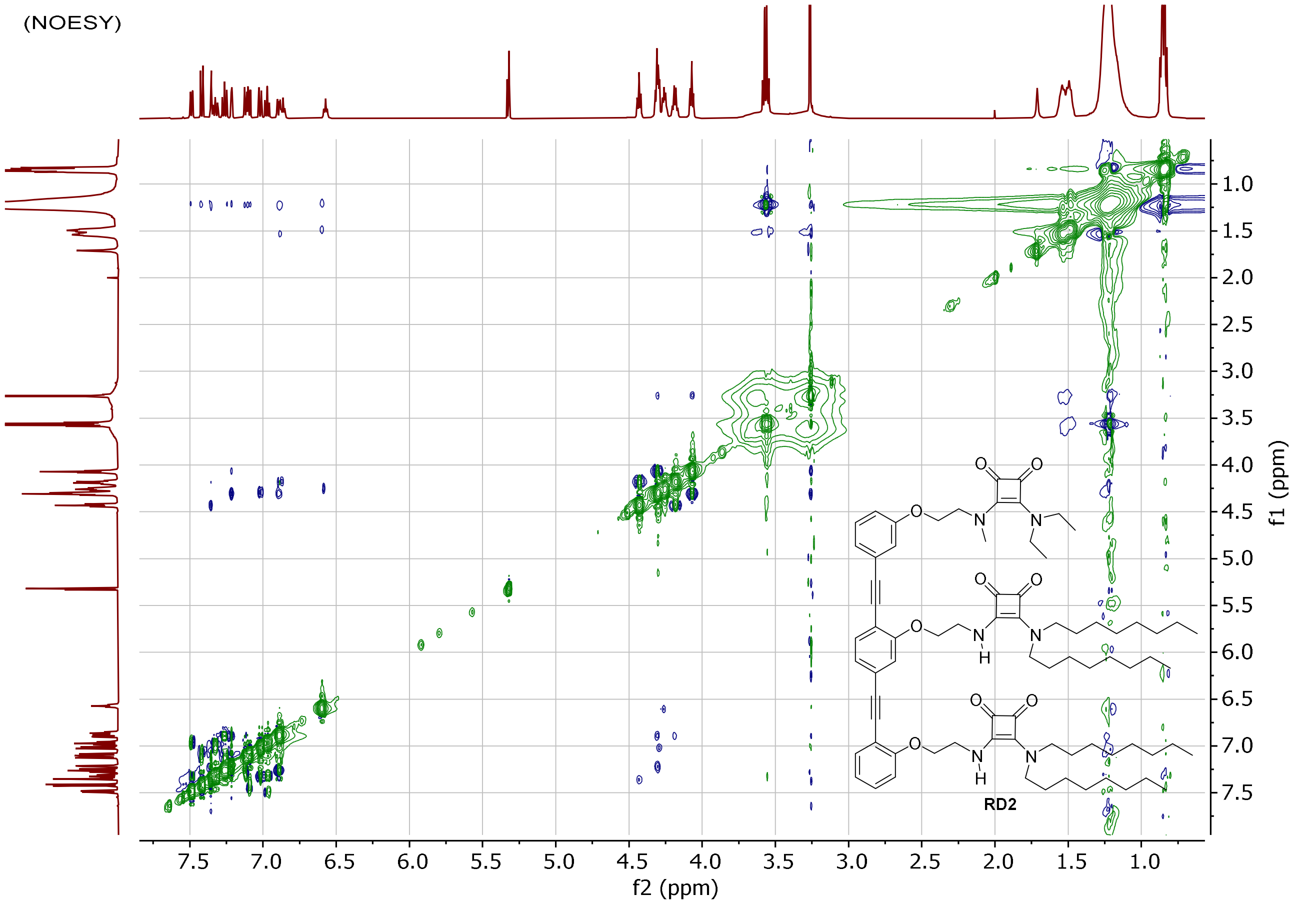

1-(3,5-Bis(trifluoromethyl)phenyl)-3-(2-(3-ethynylphenoxy)ethyl)thiourea (16)

^{1}H NMR:

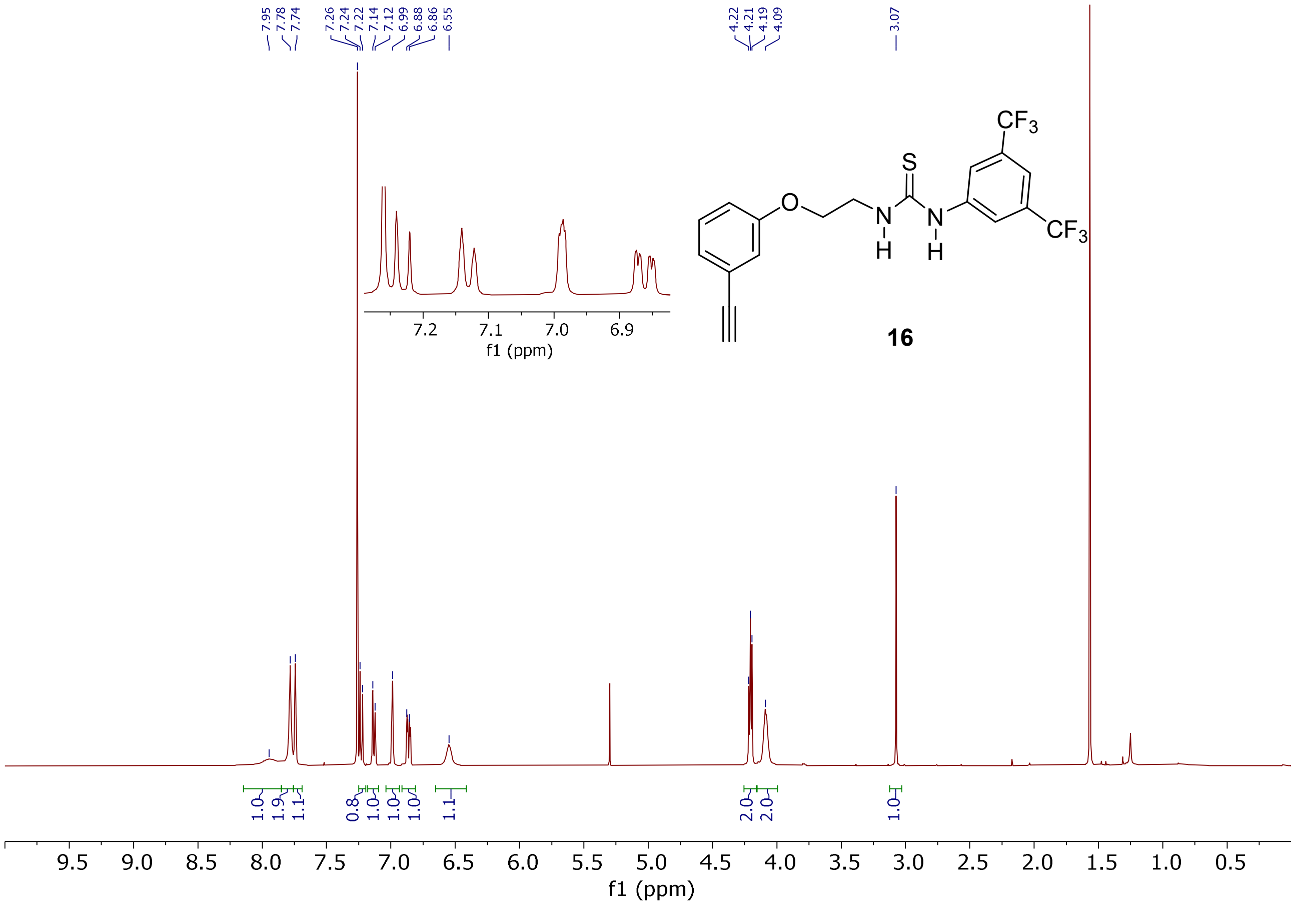

^{13}C NMR:

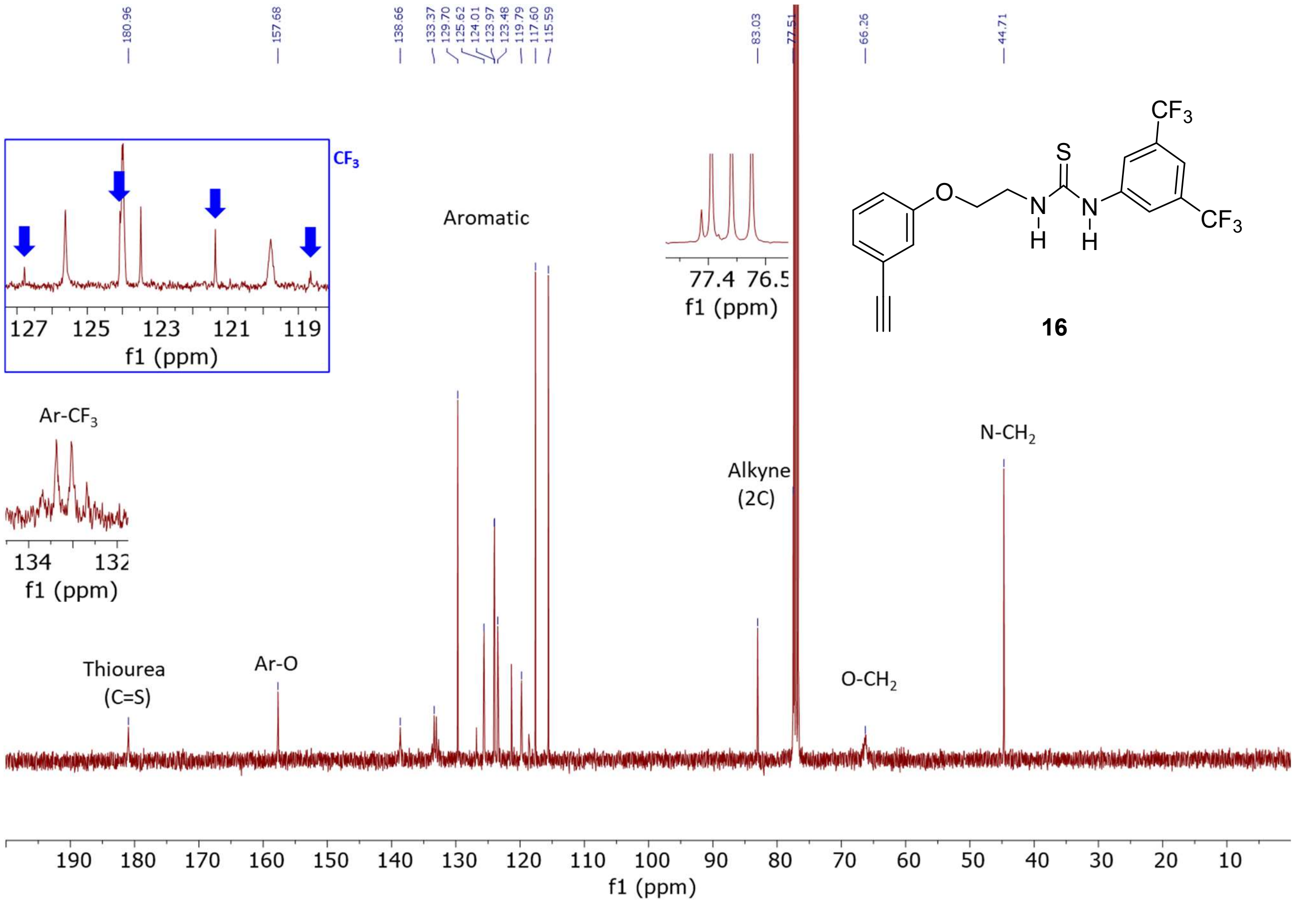

***tert*-Butyl (2-(2-((4-((3-(2-aminoethoxy)phenyl)ethynyl)-3-(2-((*tert*-butoxycarbonyl)amino)ethoxy)-phenyl)ethynyl)phenoxy)ethyl)carbamate (17)**

^{1}H NMR:

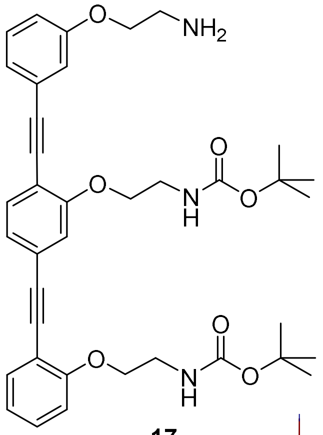

17

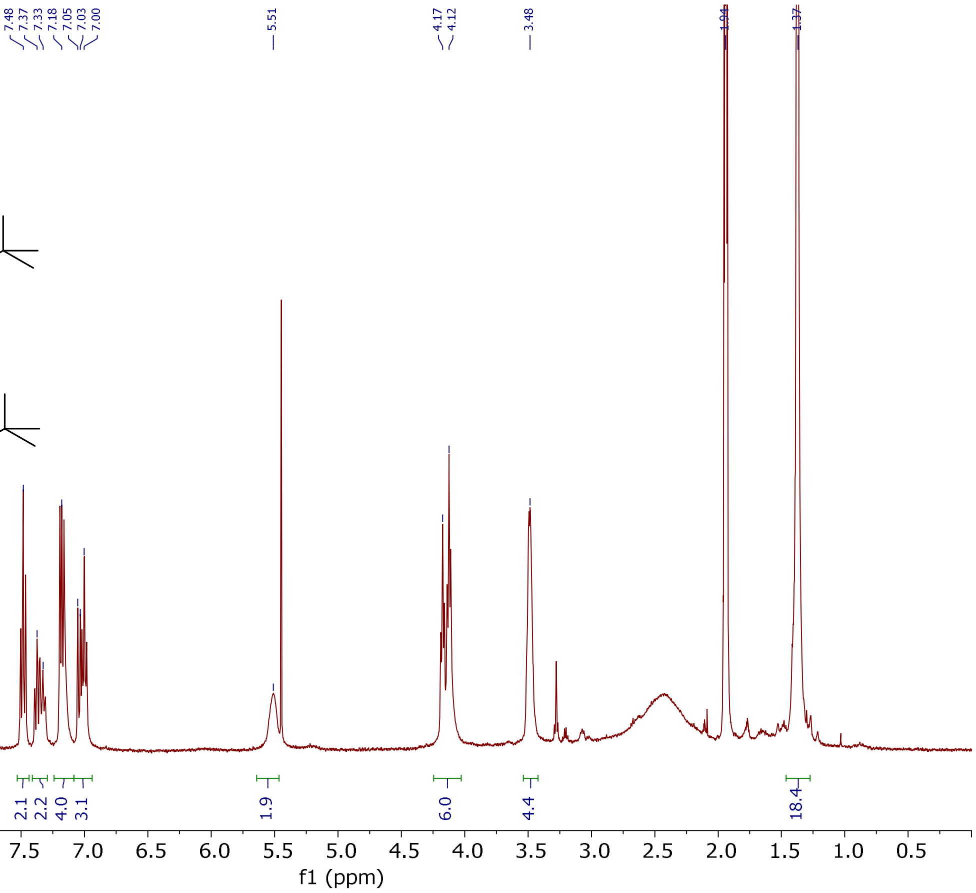

^{13}C NMR:

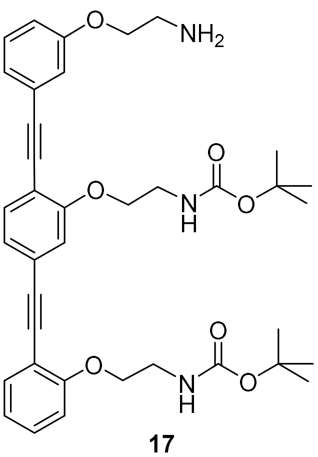

17

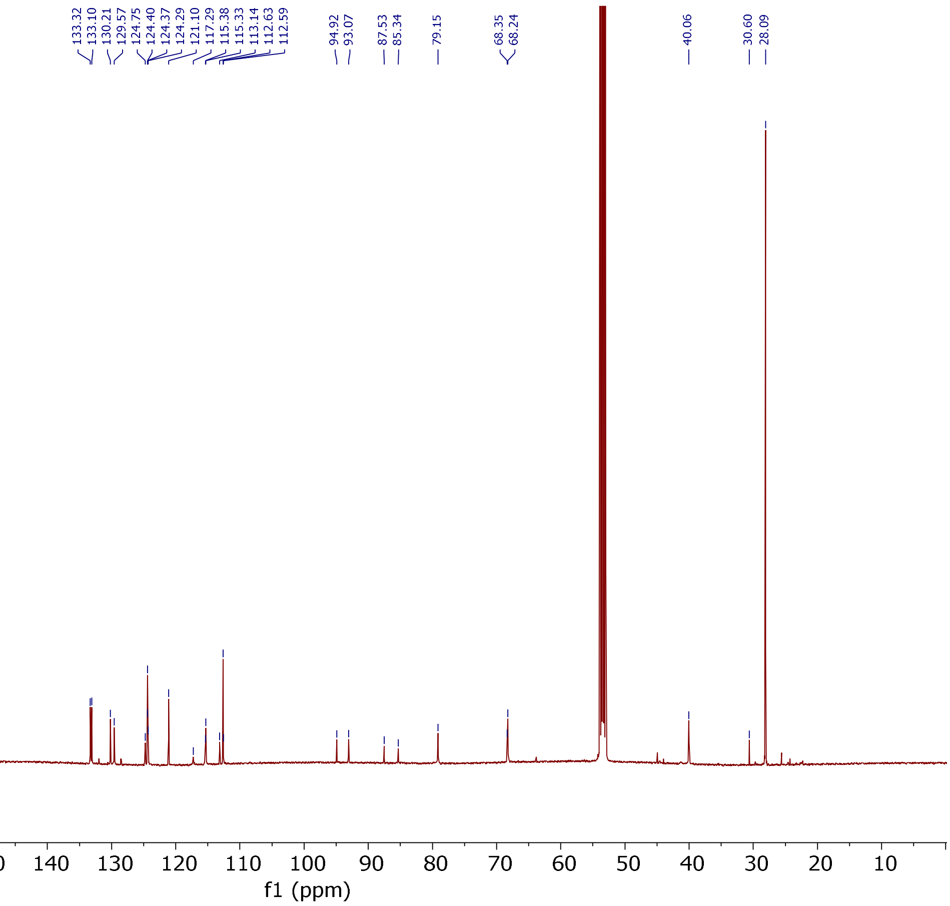

***tert*-Butyl (2-(2-((4-((3-(2-(3-(3,5-bis(trifluoromethyl)phenyl)thioureido)ethoxy)phenyl)ethynyl)-3-(2-((*tert*-butoxycarbonyl)amino)ethoxy)phenyl)ethynyl)phenoxy)ethyl)carbamate (18)**

^{1}H NMR:

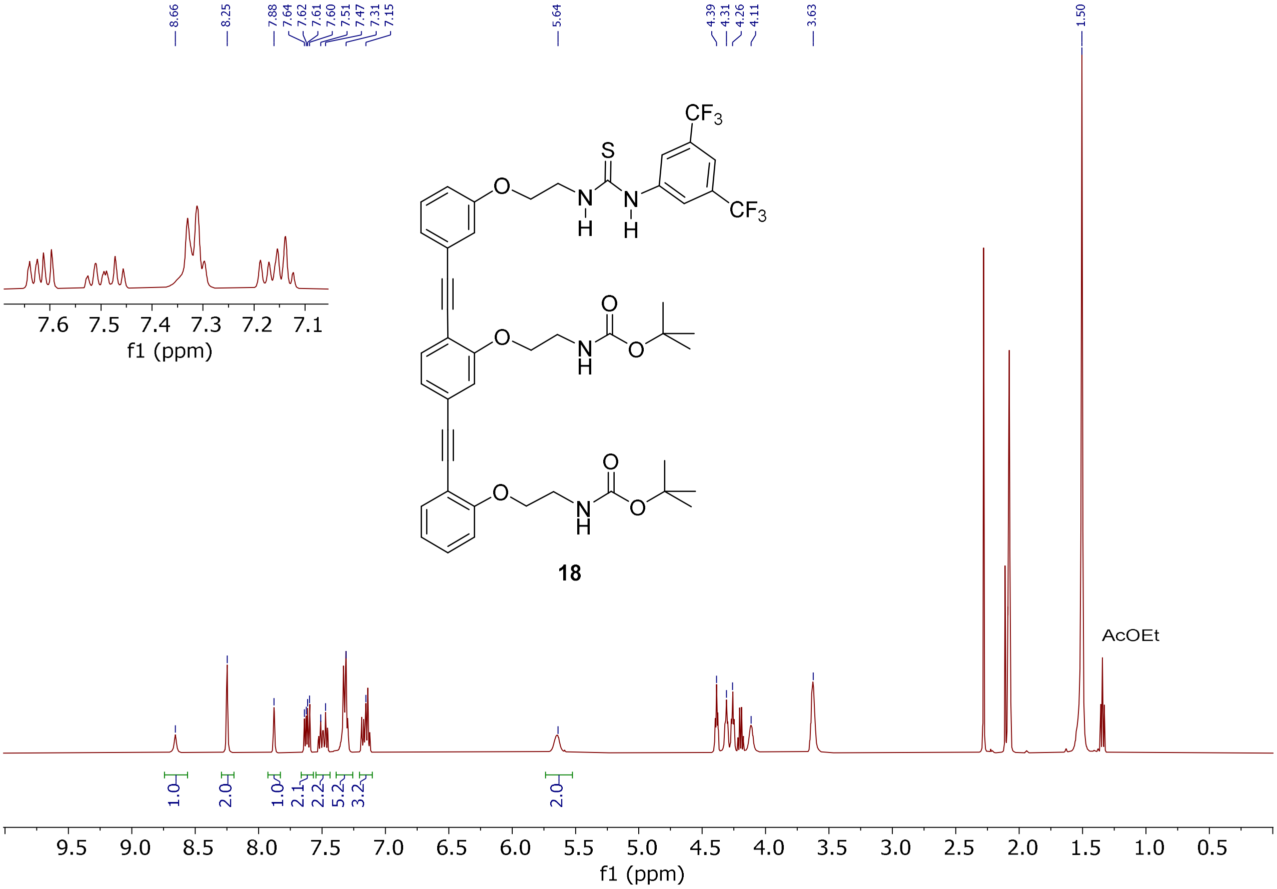

^{13}C NMR:

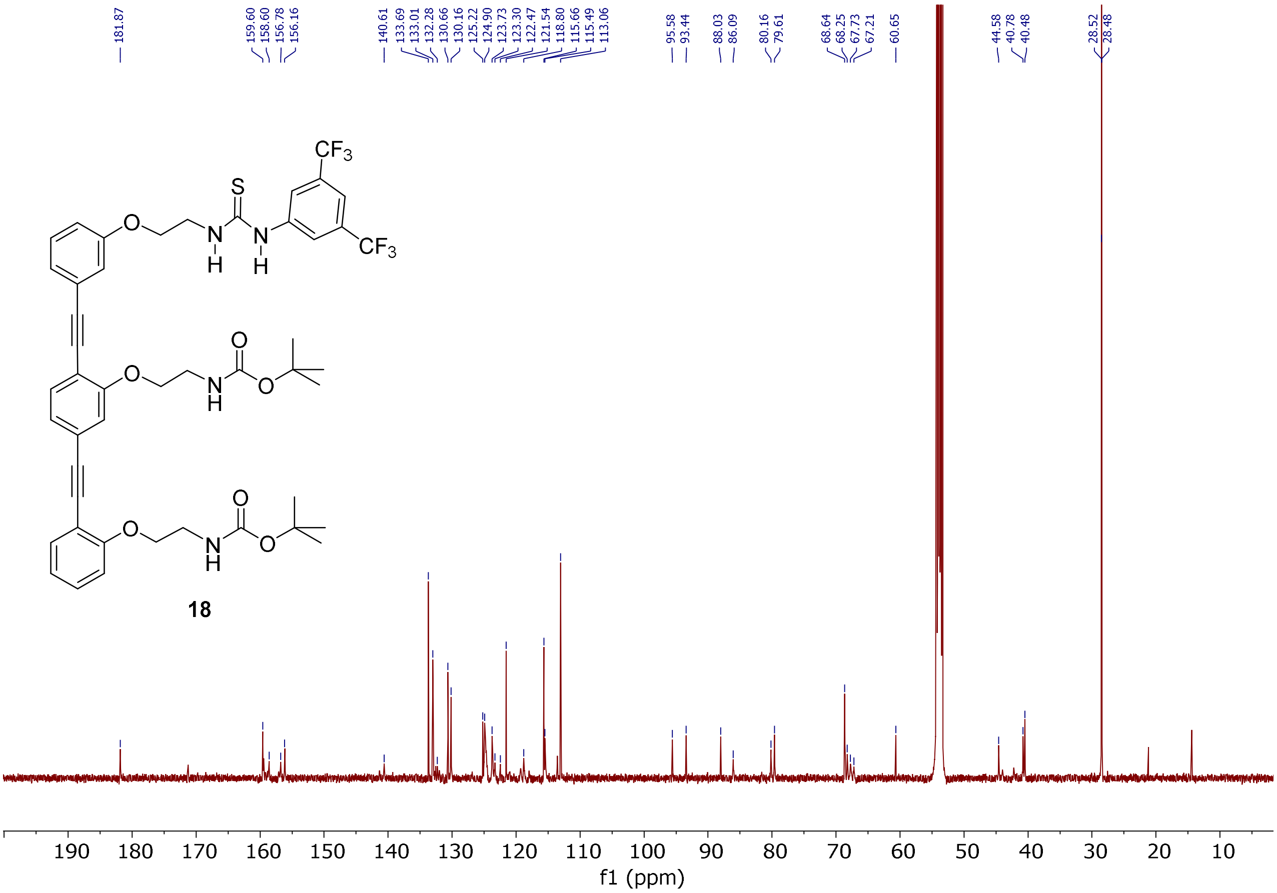

1-(3,5-Bis(trifluoromethyl)phenyl)-3-(2-(3-((2-(2-((2-(di(*n*-octyl)amino)-3,4-dioxocyclobut-1-en-1-yl)amino)-ethoxy)-4-((2-(2-((2-(di(*n*-octyl)amino)-3,4-dioxocyclobut-1-en-1-yl)amino)ethoxy)phenyl)ethynyl)phenyl)ethynyl)phenoxy)ethyl)-thiourea (RD3)

^{1}H NMR:

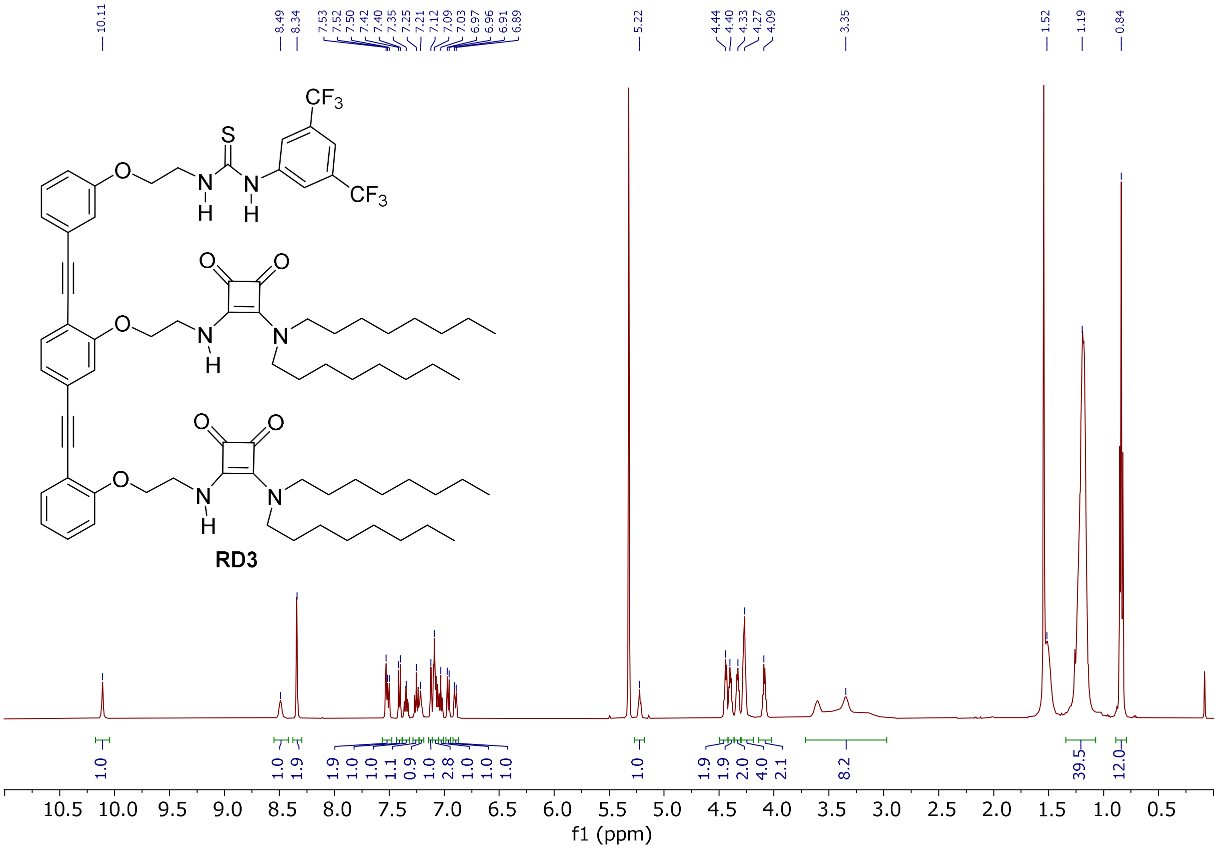

^{13}C NMR:

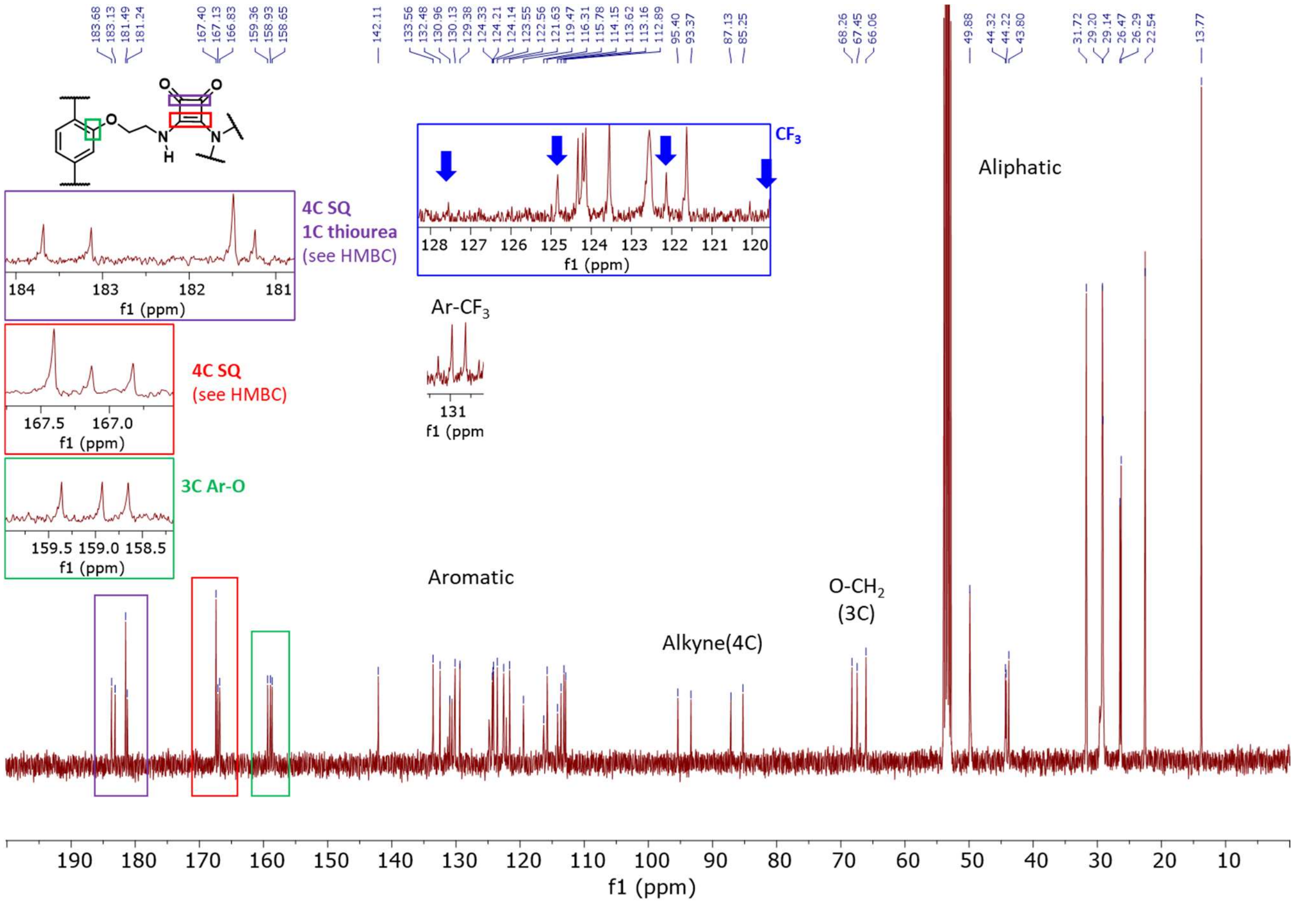

2D NMR:

(HSQC)

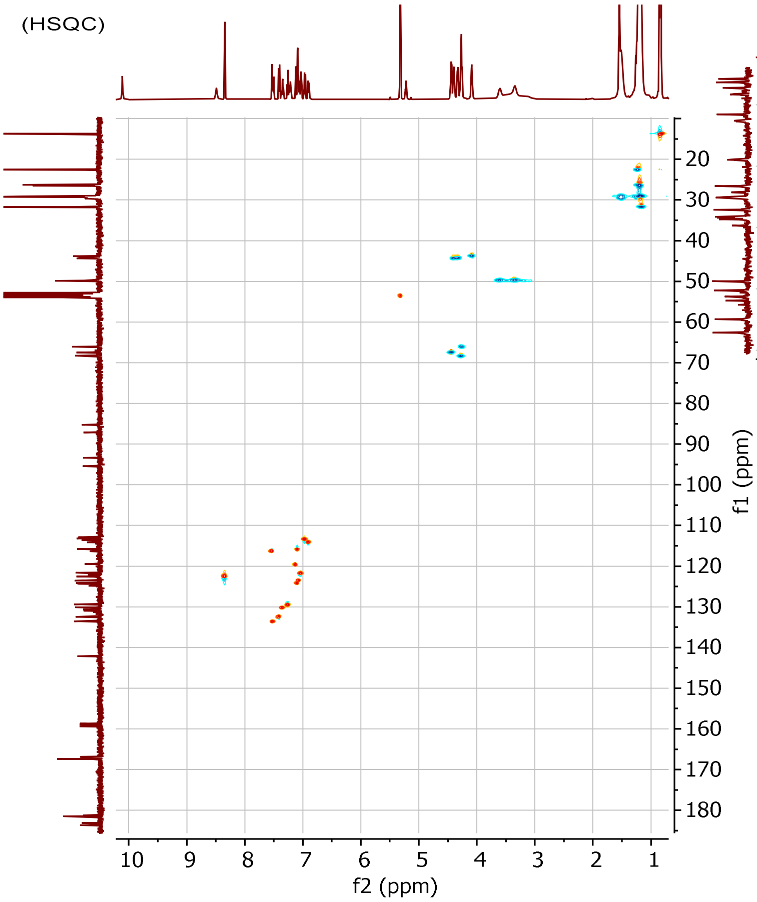

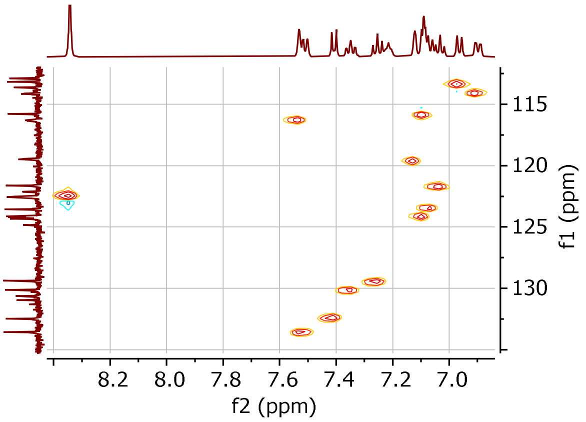

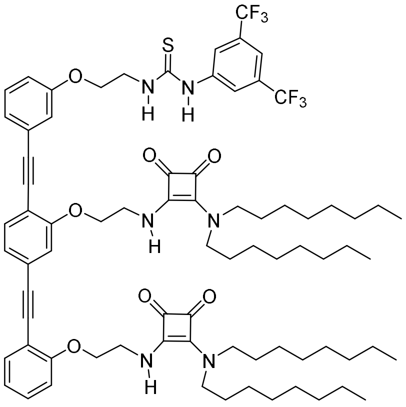

RD3

(HMBC)

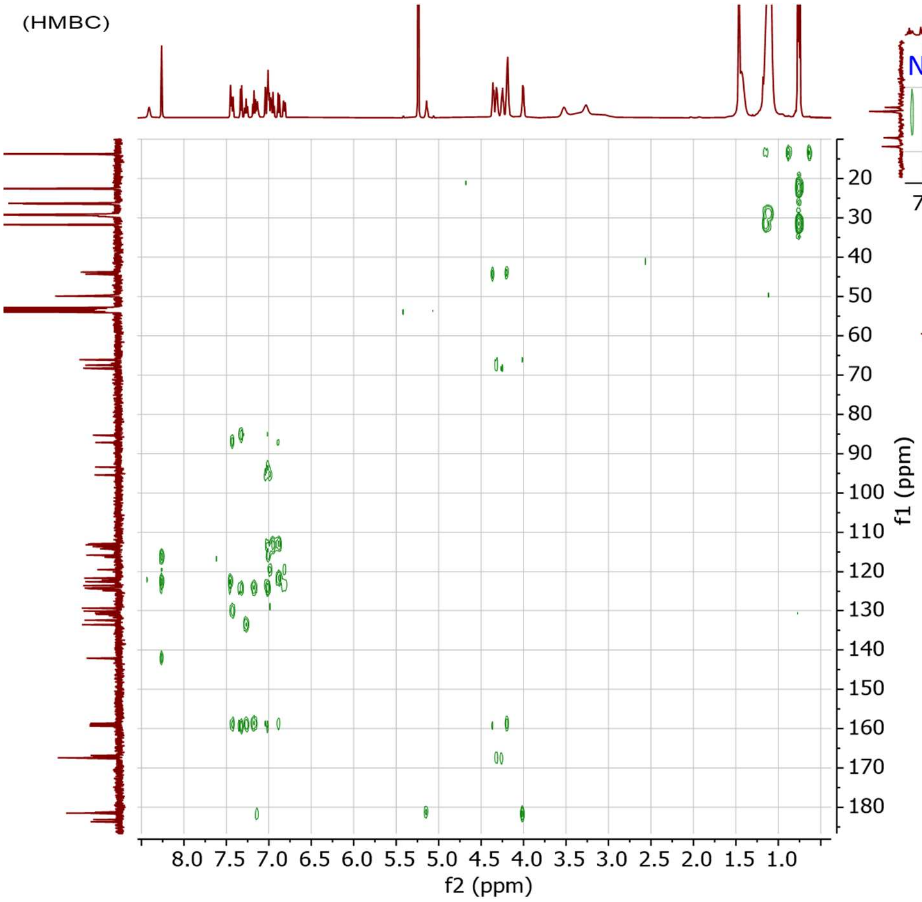

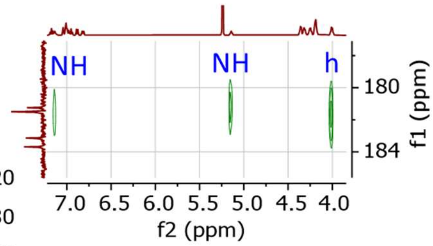

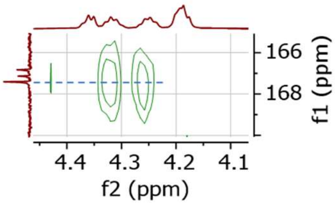

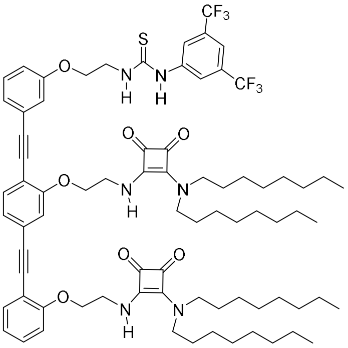

RD3

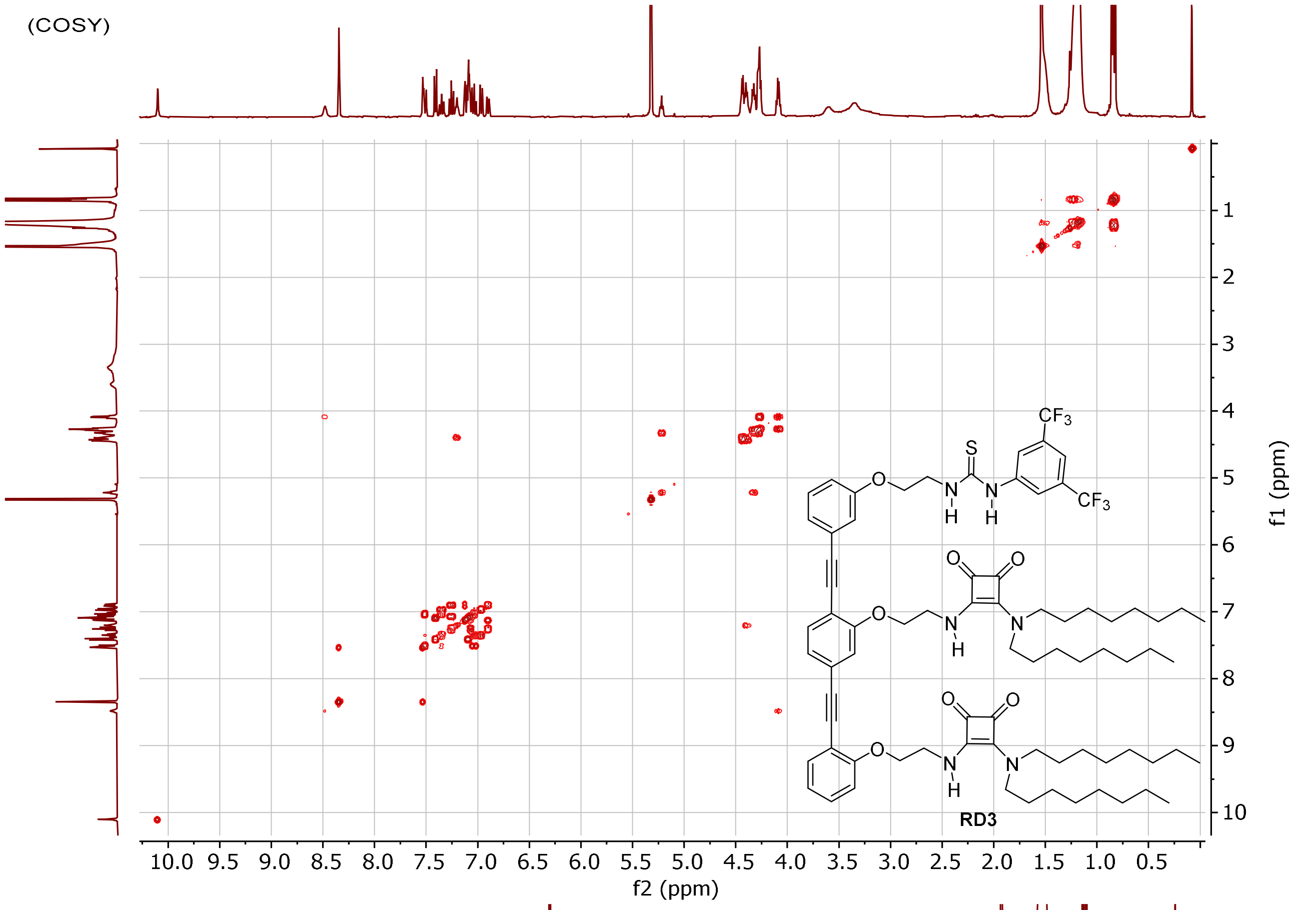
(COSY)
RD3
f2 (ppm)
f1 (ppm)

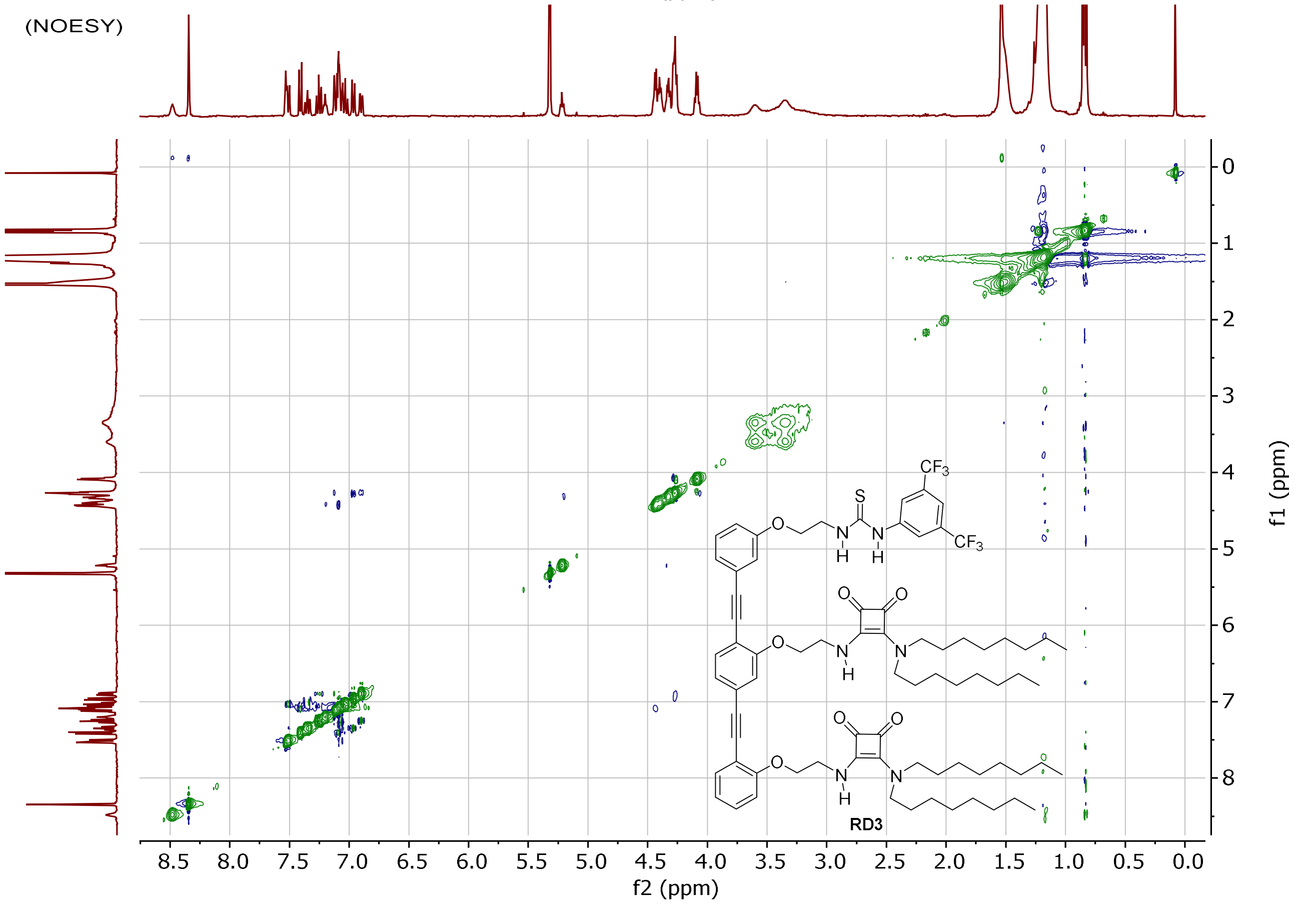
(NOESY)
RD3
f2 (ppm)
f1 (ppm)

***tert*-Butyl (2-(2-((3-(2-((*tert*-butoxycarbonyl)amino)ethoxy)-4-((3-(2-((2-((3-(dimethylamino)propyl)-(methyl)amino)-3,4-dioxocyclobut-1-en-1-yl)amino)ethoxy)phenyl)ethynyl)phenyl)ethynyl)phenoxy)-ethyl)carbamate** (**19**)

^{1}H NMR:

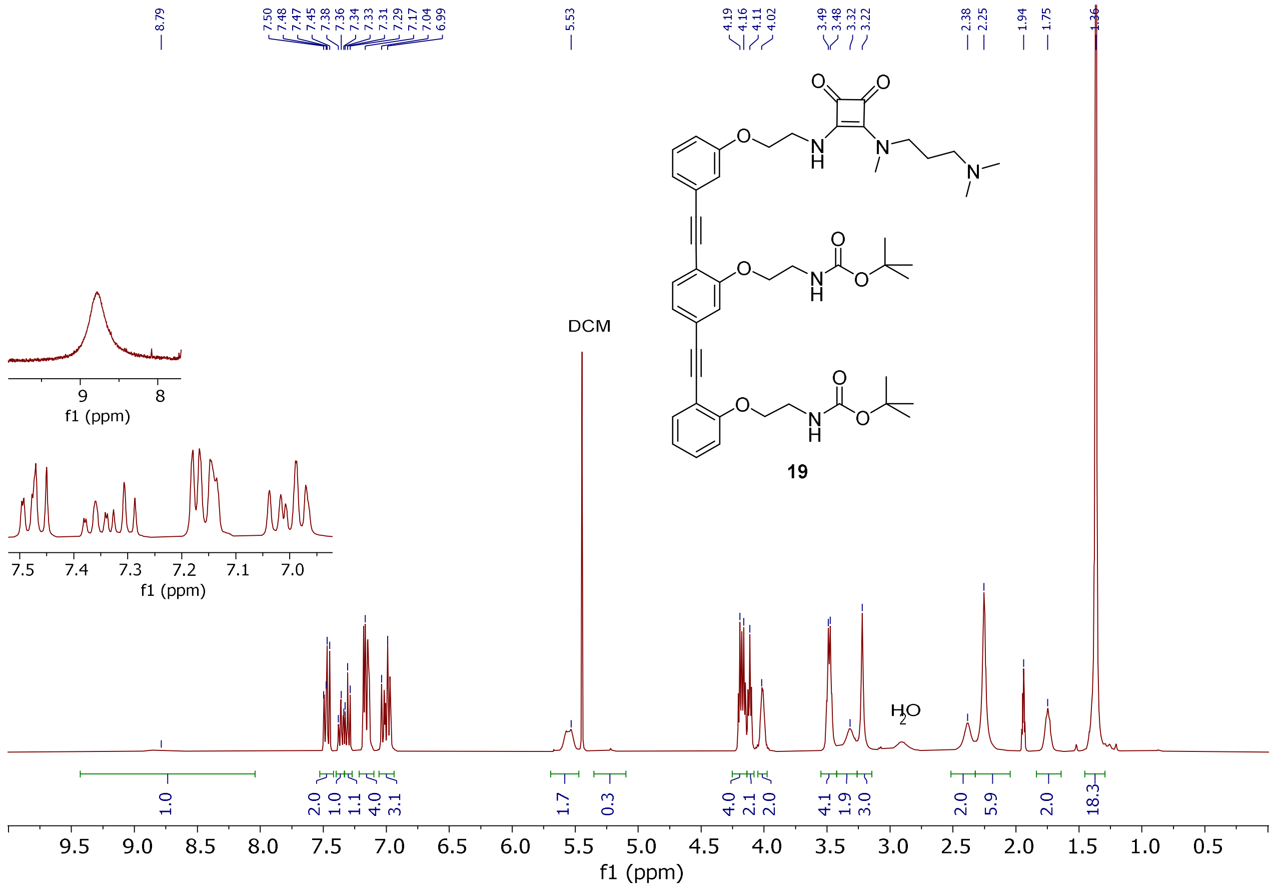

^{13}C NMR:

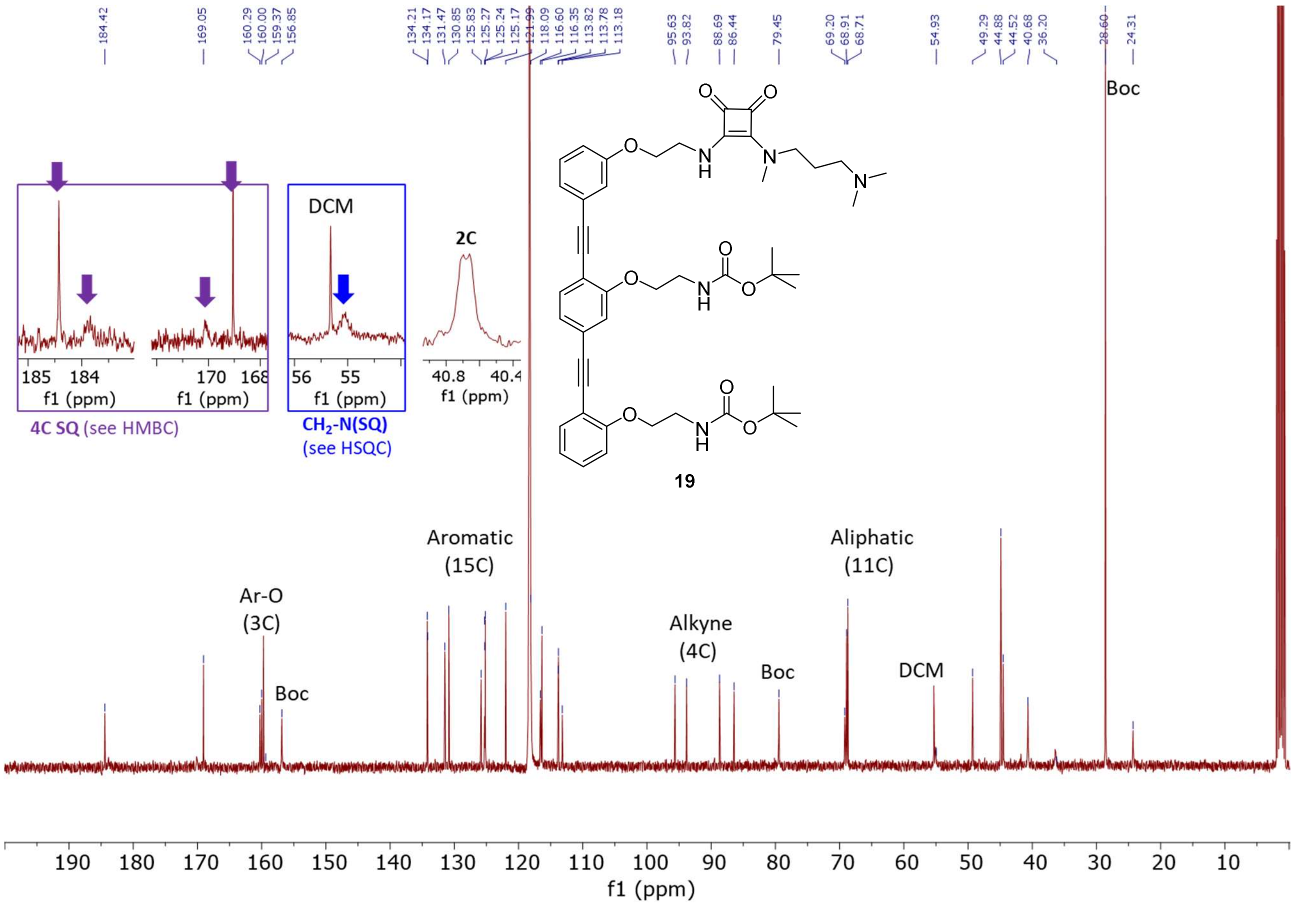

2D NMR:

(HSQC)

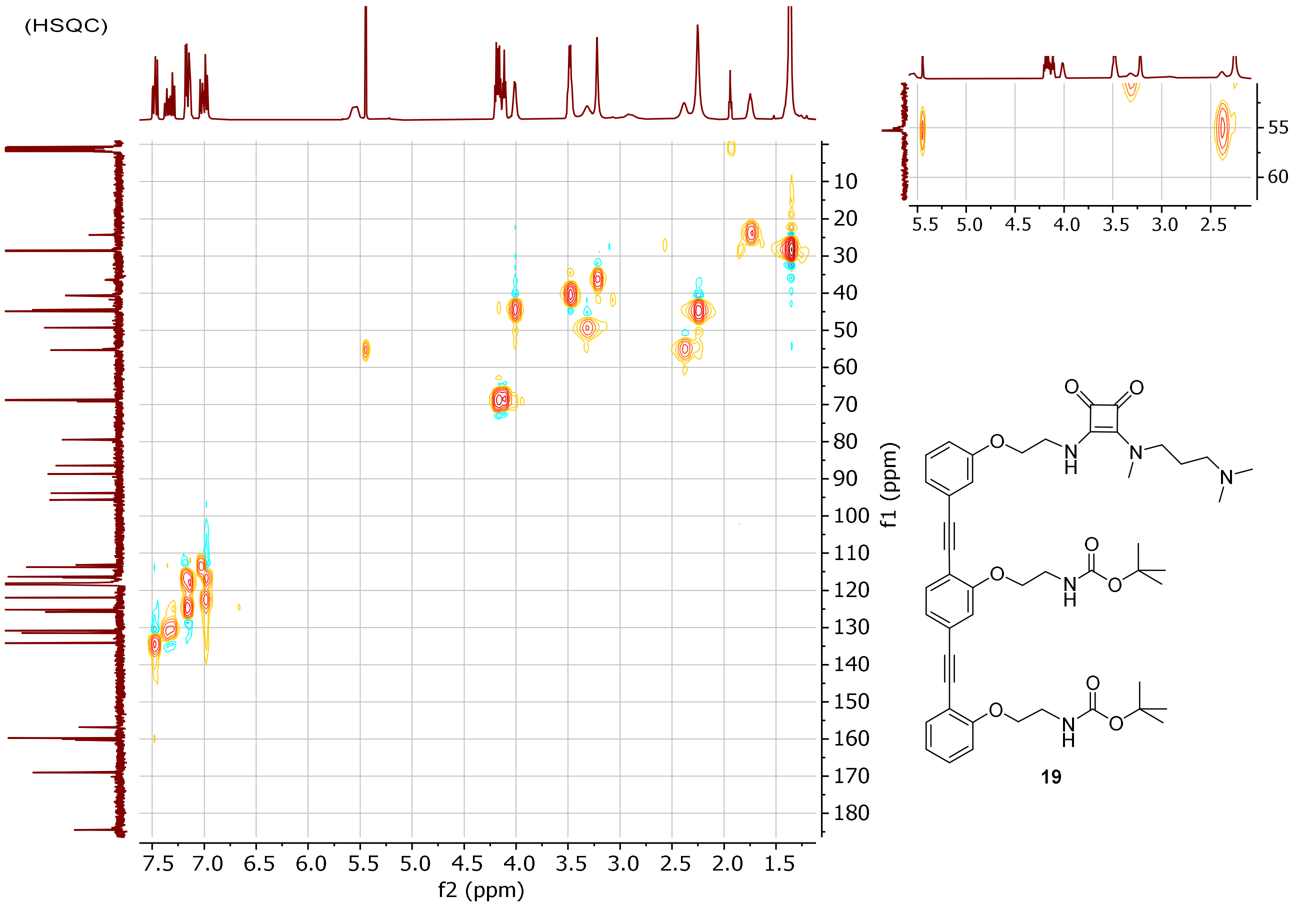

(HMBC)

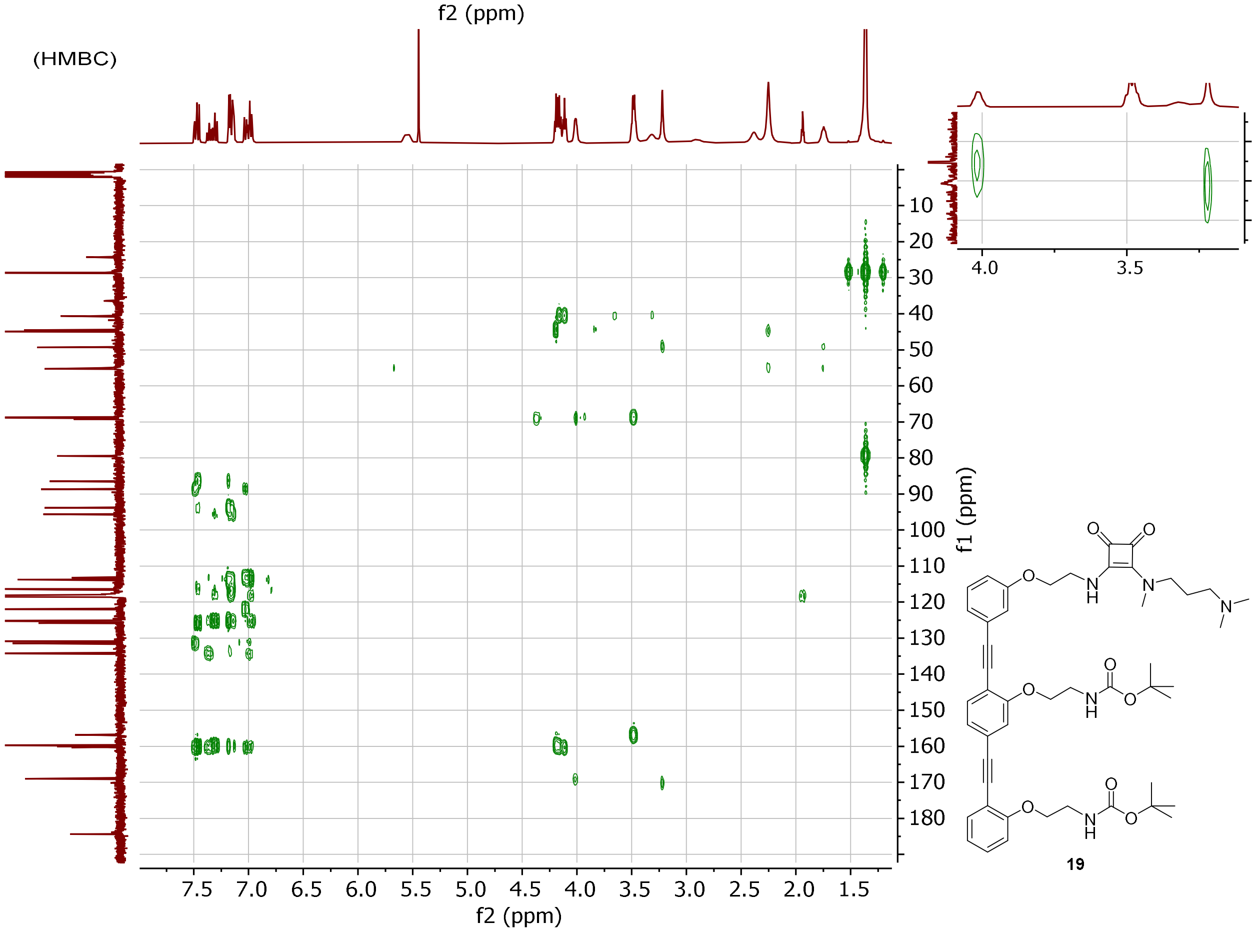

3-((3-(Dimethylamino)propyl)(methyl)amino)-4-((2-(3-((2-(2-((2-(di(*n*-octyl)amino)-3,4-dioxocyclobut-1-en-1-yl)amino)ethoxy)-4-((2-(2-((2-(di(*n*-octyl)amino)-3,4-dioxocyclobut-1-en-1-yl)amino)ethoxy)phenyl)ethynyl)-phenyl)ethynyl)phenoxy)ethyl)amino)cyclobut-3-ene-1,2-dione (**RD4**)

^{1}H NMR:

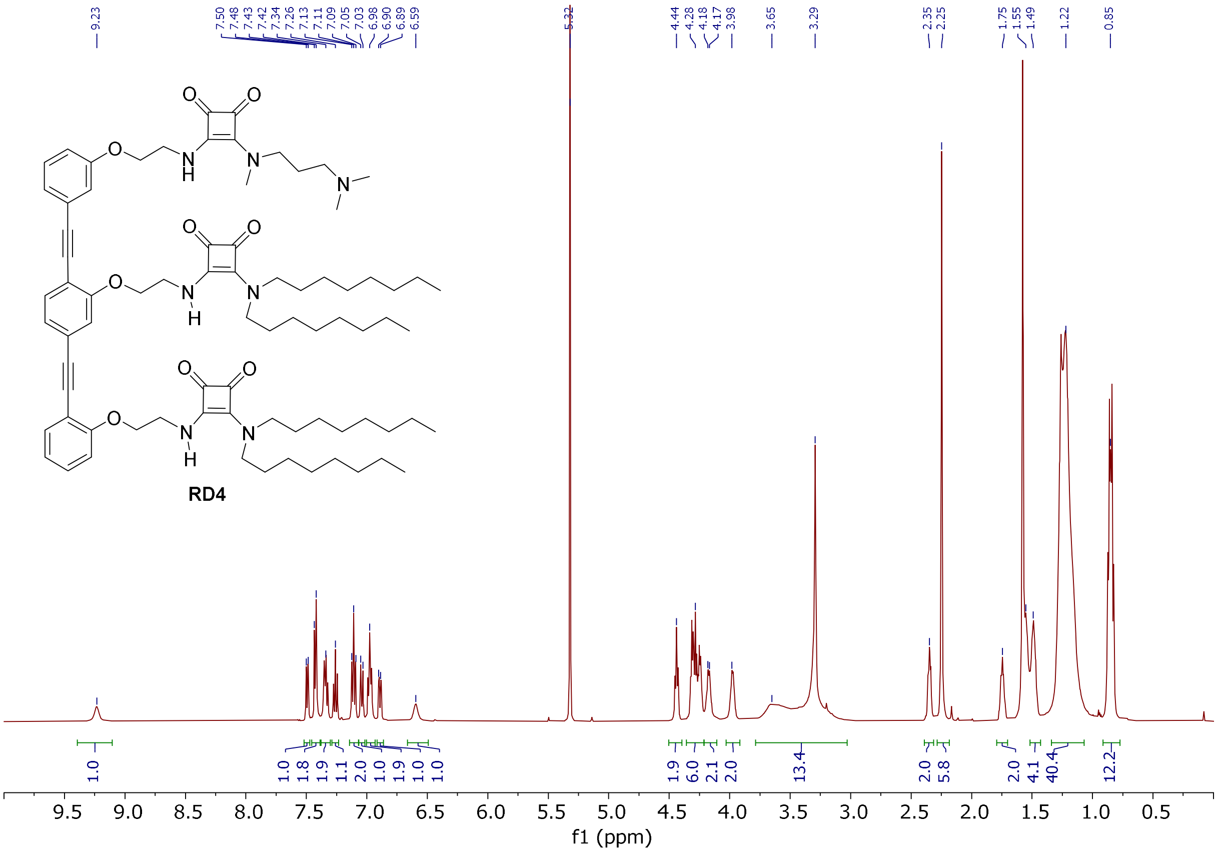

^{13}C NMR:

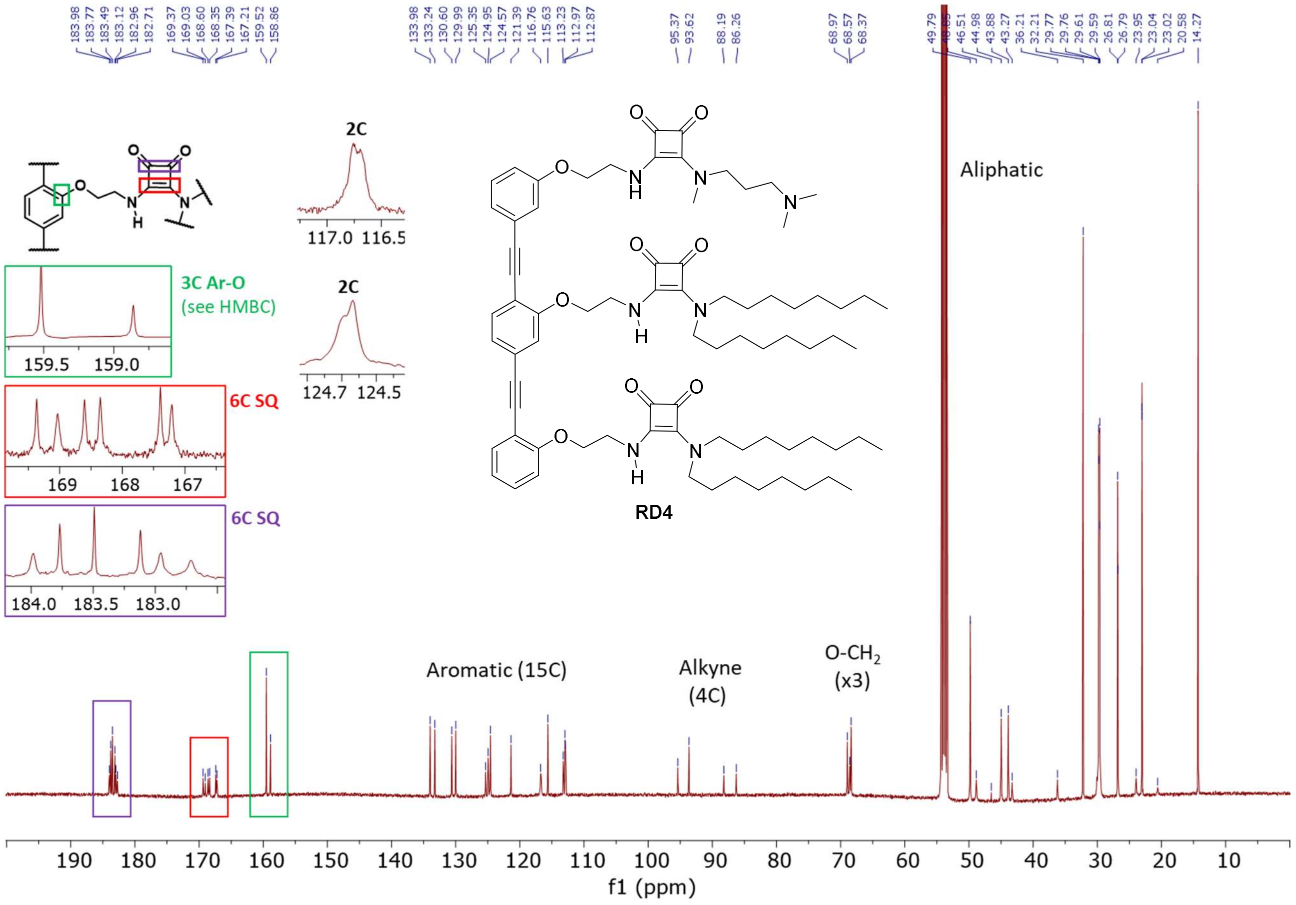

2D NMR:

(HSQC)

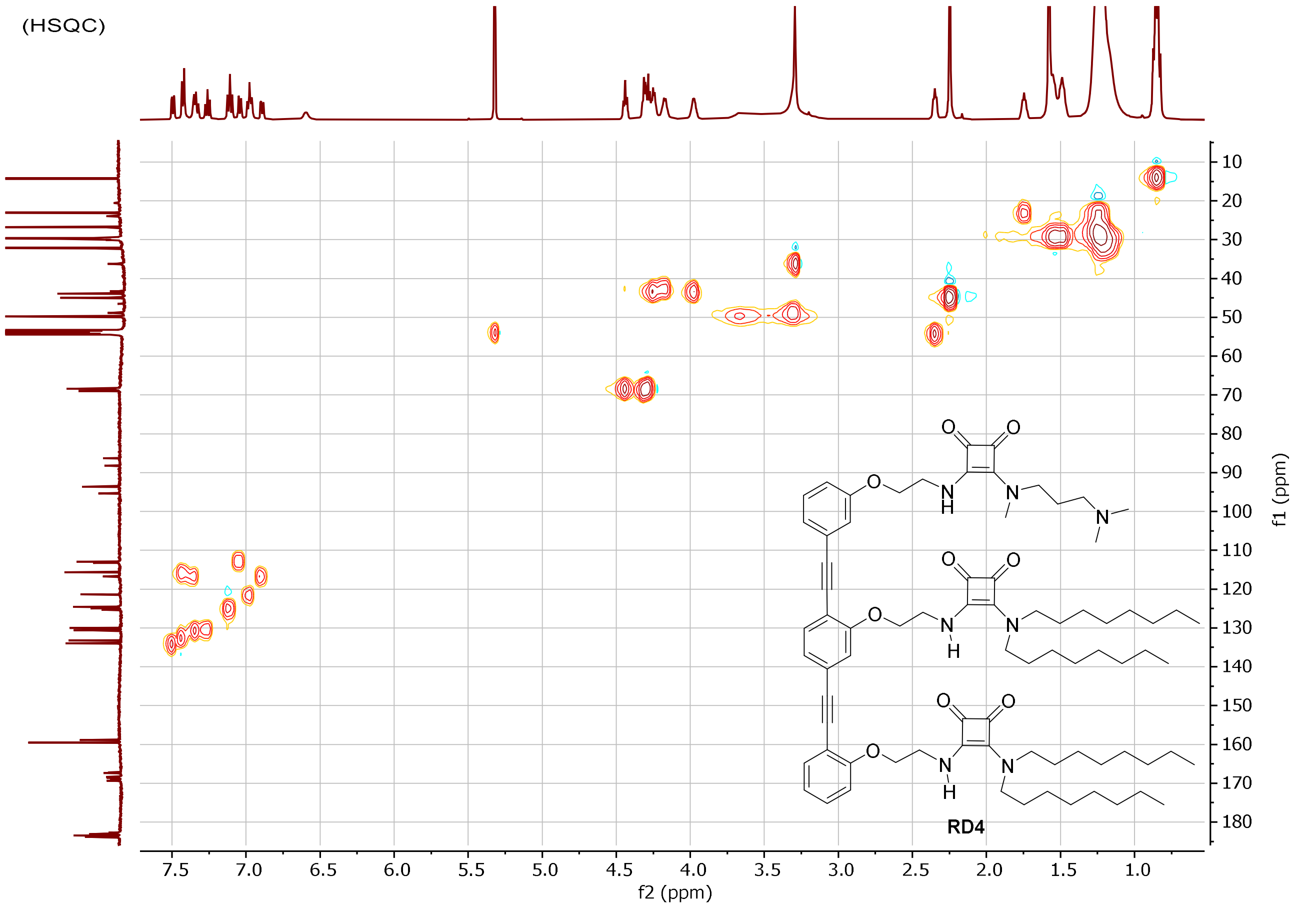

(HMCB)

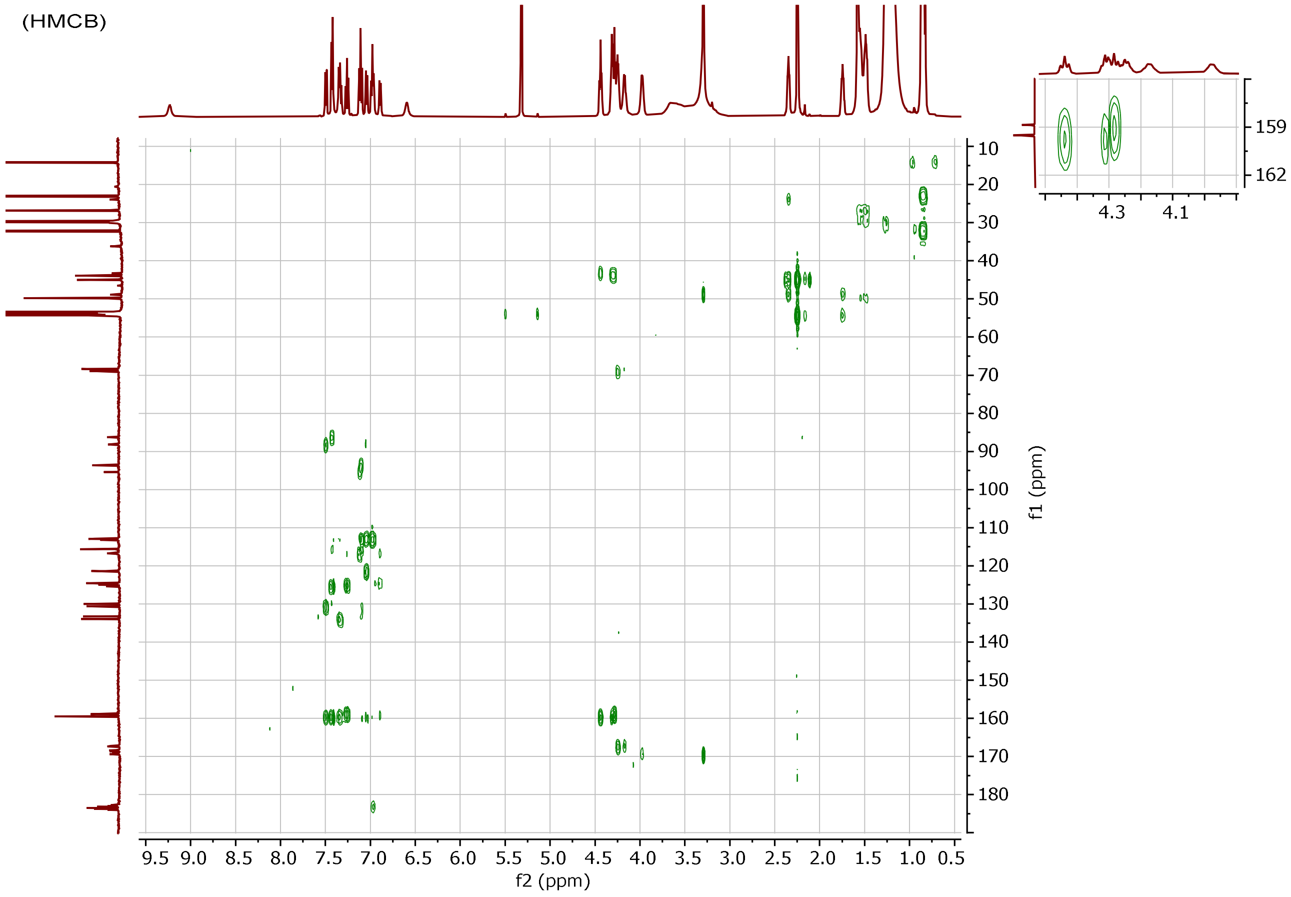

(COSY)

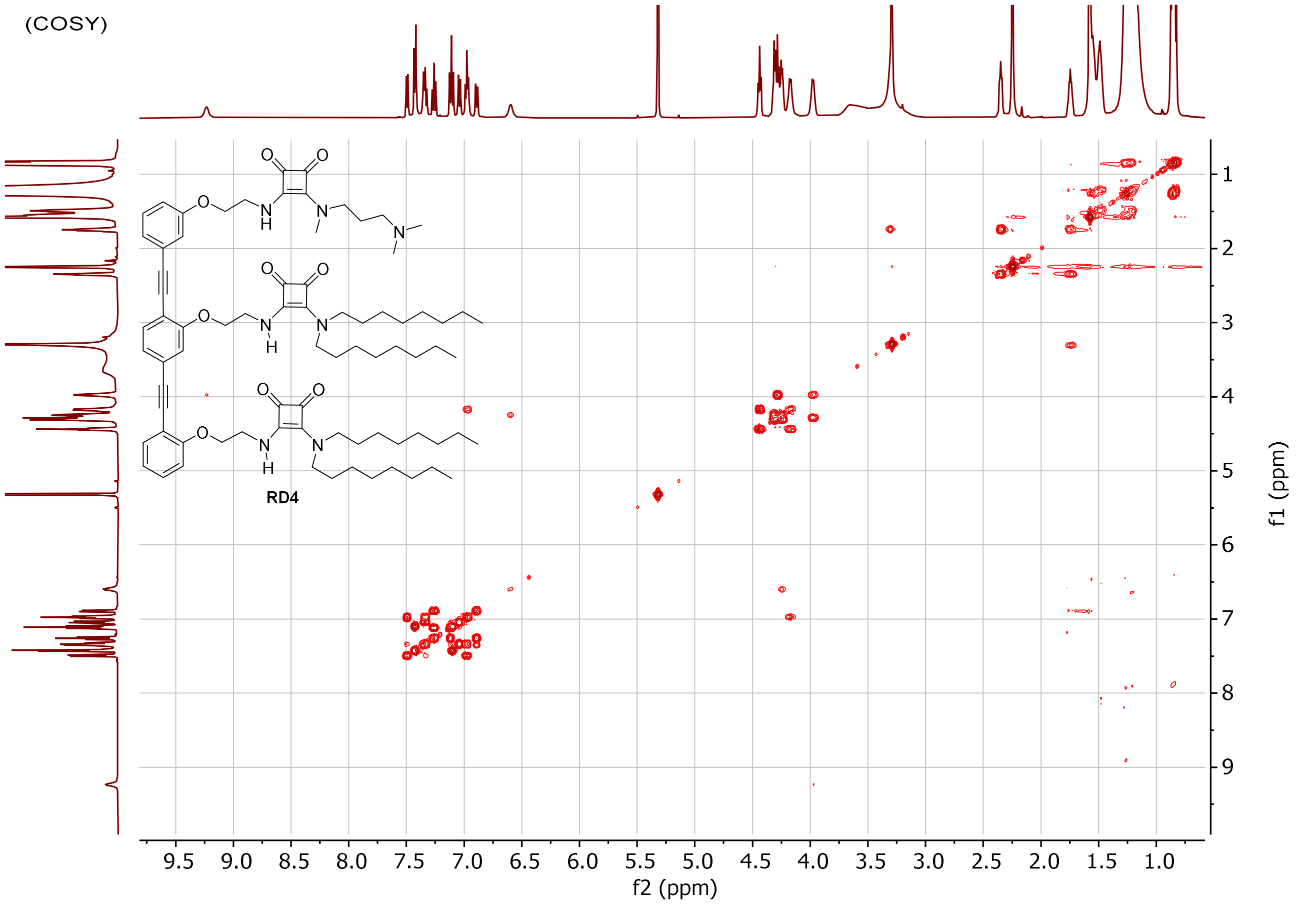

(NOESY)

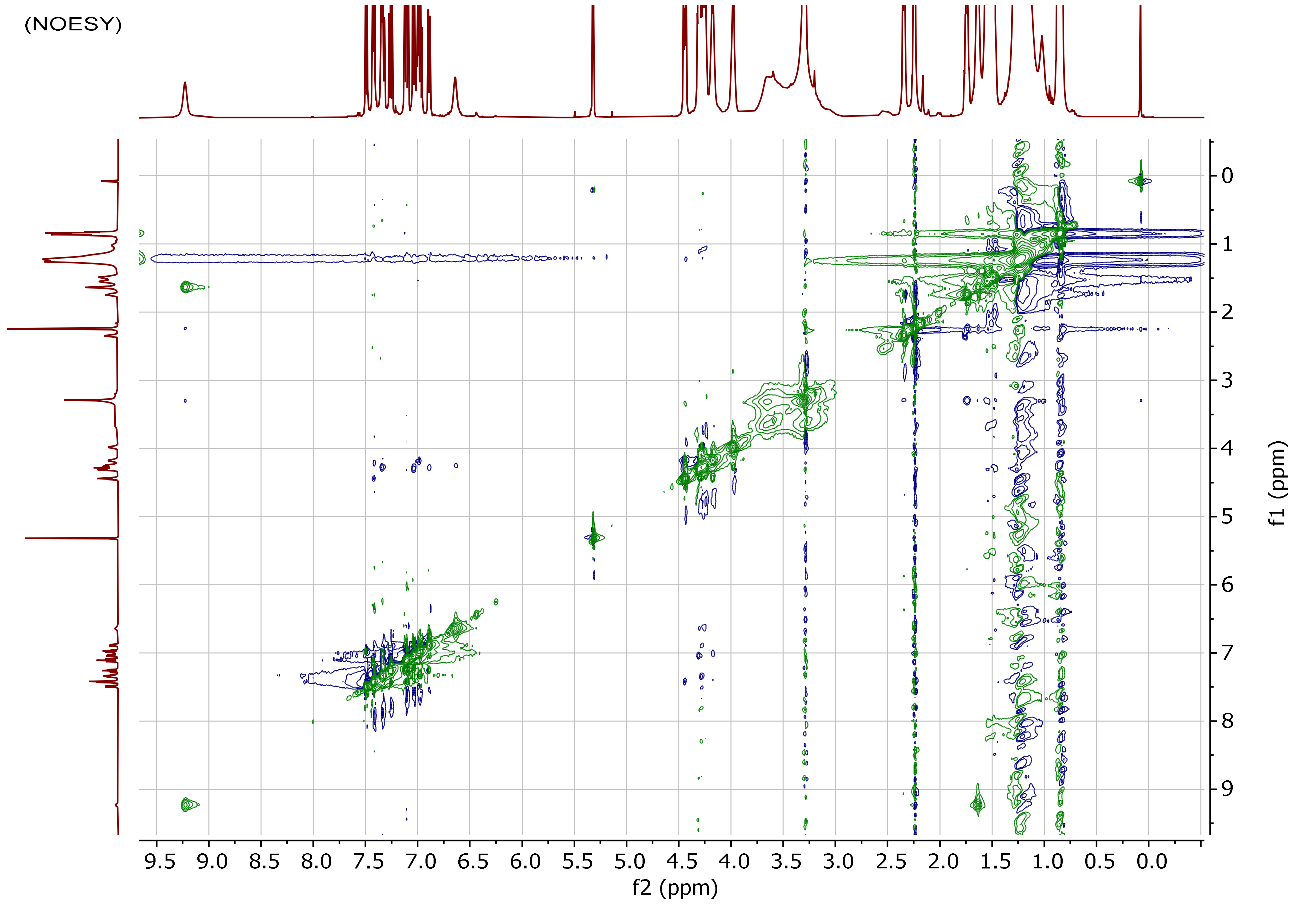

9. References

S1. Fulmer, G.R., Miller, A.J.M., Sherden, N.H., Gottlieb, H.E., Nudelman, A., Stoltz, B.M., Bercaw, J.E., and Goldberg, K. I.. NMR Chemical Shifts of Trace Impurities: Common Laboratory Solvents, Organics, and Gases in Deuterated Solvents Relevant to the Organometallic Chemist. *Organometallics*, **2010**, *29*, 2176–2179. 10.1021/om100106e.

S2. Jester, S.-S.; Sigmund, E.; and Höger. S. Nanopatterning by Molecular Polygons. *J. Am. Chem. Soc.*, **2011**, *133*, 11062–11065. 10.1021/ja203536t.

S3. Prohens, R., Portell, A., Font-Bardia, M., Bauzá, A., and Frontera, A. (2016) Experimental and theoretical study of weak intermolecular interactions in crystalline tertiary squaramides. *CrystEngComm*, *18*, 6437–6443. 10.1039/C6CE01299J.

S4. Olmo, F., Rotger, C., Ramírez-Macías, I., Martínez, L., Marín, C., Carreras, L., Urbanová, K., Vega, M., Chaves-Lemaur, G., Sampedro, A., Rosales, M.J., Sánchez-Moreno, M., and Costa A. Synthesis and Biological Evaluation of *N*,*N*′-Squaramides with High in Vivo Efficacy and Low Toxicity: Toward a Low-Cost Drug against Chagas Disease. *J. Med. Chem.*, **2014**, *57*, 987–999. 10.1021/jm4017015.

S5. Morris, D.T.J., Wales, S.M., Tilly, D.P., Farrar, E.H.E., Grayson, M.N., Ward, J.W., and Clayden, J. A molecular communication channel consisting of a single reversible chain of hydrogen bonds in a conformationally flexible oligomer. *Chem.*, **2021**, *7*, 2460-2472. 10.1016/j.chempr.2021.06.022.

S6. Sheldrick, G. M. Crystal Structure Refinement with SHELXL. *Acta Crystallogr. Sect. C Struct. Chem.,* **2015**, *71* (Md), 3–8. 10.1107/S2053229614024218.

S7. Dolomanov, O. V., Bourhis, L. J., Gildea, R. J., Howard, J. A. K., and Puschmann, H. OLEX2: A Complete Structure Solution, Refinement and Analysis Program. *J. Appl. Crystallogr.,* **2009**, *42*, 339–341. 10.1107/S0021889808042726.
